# Seed Menus: An integrated decision‐support framework for native plant restoration in the Mojave Desert

**DOI:** 10.1002/ece3.8805

**Published:** 2022-04-12

**Authors:** Daniel F. Shryock, Lesley A. DeFalco, Todd C. Esque

**Affiliations:** ^1^ U.S. Geological Survey Western Ecological Research Center Boulder City Nevada USA

**Keywords:** decision support tool, Mojave Desert, native plants, priority species list, restoration, seed mix, Shiny application, species distribution model

## Abstract

The combination of ecosystem stressors, rapid climate change, and increasing landscape‐scale development has necessitated active restoration across large tracts of disturbed habitats in the arid southwestern United States. In this context, programmatic directives such as the National Seed Strategy for Rehabilitation and Restoration have increasingly emphasized improved restoration practices that promote resilient, diverse plant communities, and enhance native seed reserves. While decision‐support tools have been implemented to support genetic diversity by guiding seed transfer decisions based on patterns in local adaptation, less emphasis has been placed on identifying priority seed mixes composed of native species assemblages. Well‐designed seed mixes can provide foundational ecosystem services including resilience to disturbance, resistance to invasive species, plant canopy structure to facilitate natural seedling recruitment, and habitat to support wildlife and pollinator communities. Drawing from a newly developed dataset of species distribution models for priority native plant taxa in the Mojave Desert, we created a novel decision support tool by pairing spatial predictions of species habitat with a database of key species traits including life history, flowering characteristics, pollinator relationships, and propagation methods. This publicly available web application, Mojave Seed Menus, helps restoration practitioners generate customized seed mixes for native plant restoration in the Mojave Desert based on project locations. Our application forms part of an integrated Mojave Desert restoration program designed to help practitioners identify species to include in local seed mixes and nursery stock development while accounting for local adaptation by identifying appropriate seed source locations from key restoration species.

## Graphical abstract

We describe an integrated decision‐support framework for native plant restoration in the Mojave Desert, including online applications available through the USGS and BLM Mojave Desert Native Plant Program. With this framework, restoration practitioners and resource managers can create seed mixes for restoration sites and identify appropriate seed sources using two complimentary web applications, Mojave Seed Menus and Climate Distance Mapper.

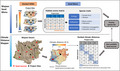

## 1 INTRODUCTION

Restoring degraded environments to diverse and resilient ecosystems is a fundamental conservation goal, but one that is increasingly challenging due to accelerating human development and rapid climate change across much of the globe. Evidence suggests that diversity is key to ecosystem stability and ability to withstand novel stressors (Isbell et al., 2015; Tilman & Downing, 1994). This pattern holds at multiple levels of organization from the regional to the plant community and genotypic levels (Oliver et al., 2015). Awareness of the fundamental role of diverse, connected ecosystems has resulted in a paradigm shift in restoration ecology, from previous efforts tailored for rapid soil stability and erosion control (e.g., use of cultivars or soil‐stabilizing species from outside regions) to native plant materials development programs aimed at increasing local seed reserves, promoting genetic diversity, and minimizing risk from long‐distance seed transfer (Kettenring et al., 2014; Oldfield & Olwell, 2015; Olwell & Riibe, 2016). Substantial challenges remain for restoration practitioners seeking to apply these principles across a variety of disturbed environments.

The desert ecoregions of the southwestern United States are particularly challenging environments to restore (Lovich & Bainbridge, 1999). These ecoregions are increasingly threatened by stressors such as climate change, which is creating a hotter and drier climate and may shift seasonal precipitation patterns (Dai, 2013; IPCC, 2013), putting local ecotypes at a phenological disadvantage (Kimball et al., 2010). Moreover, widespread invasions of annual grass species (e.g., *Bromus tectorum* and *B*. *madritensis*) have altered shrubland communities across the southwestern United States and contributed to wildfires unprecedented in size and frequency (Brooks et al., 2004; D’Antonio & Vitousek, 1992). Disturbance impacts in deserts are also compounded by the notoriously slow pace of plant community recovery (Engel & Abella, 2011; Webb & Newman, 1982). Recruitment and establishment of desert shrubland plants occurs largely during infrequent resource pulses, with little regeneration outside of these periods (Chesson et al., 2004). Moreover, many desert woody species do not readily resprout after wildfire or surface disturbance (Abella, 2009), and resprouting does not guarantee survival following disturbance (DeFalco et al., 2010). Instead, replenishment of soil seed banks by seeds dispersing from intact areas depends largely on seasonal precipitation pulses that favor reproduction (Bamberg et al., 1976; Meyer & Pendleton, 2015). Persistent soil seed banks have evolved bet‐hedging strategies to circumvent reproductive failure (Angert et al., 2009), yet seedling recruitment often fails because disturbance to the soil surface diminishes seed banks (DeFalco et al., 2009; Esque, Young, et al., 2010) and reduces shrub cover for wildlife and nurse plants that facilitate establishment of native seedlings (Brown & Minnich, 1986; Cave & Patten, 1984), particularly in the presence of invasive species (Esque, Kaye, et al., 2010). In coming decades, the footprint of landscape‐scale disturbance is likely to increase across the southwestern United States, in part due to planned utility‐scale renewable energy development (Bureau of Land Management & U.S. Department of Energy, 2015; Hernandez et al., 2014). Hence, there is a clear need for effective restoration strategies that overcome ecosystem stressors in this region and promote healthy, diverse, and resilient landscapes.

Seeding efforts in the desert southwest have often had limited success (Knutson et al., 2014), even while the frequency and scale of such treatments have increased concomitantly with a shift toward the use of native species (Copeland et al., 2018). Recently, national programs such as the National Seed Strategy (Olwell & Riibe, 2016), Seeds of Success (Haidet & Olwell, 2015), and the National Strategy to Promote the Health of Honeybees and Other Pollinators (Vilsack & McCarthy, 2015) have funded efforts to put “the right seed in the right place at the right time” and supported the development of diverse native seed reserves, along with improved restoration techniques. For example, the Bureau of Land Management (BLM) Mojave Desert Native Plant Program has taken a multi‐faceted approach that uses science to discriminate among best restoration techniques, identify priority restoration species (Esque et al., 2021) and plant functional groups (Shryock et al., 2014), and develop seed transfer zones using landscape genomics and common garden studies (Shryock et al., 2017). However, a topic that has received less emphasis, but has a large potential impact, is the development of geographically appropriate seed mixes that promote diverse native species assemblages. Well‐designed seed mixes can promote community resilience by restoring diversity (Isbell et al., 2015) and functional traits (Balazs et al., 2020), resisting competitive pressure from invasive species (Abella et al., 2012), and providing essential cover and forage for wildlife (Esque et al., 2021). Moreover, custom seed mixes can be tailored toward restoring plant‐pollinator associations in denuded areas, as these relationships are critical to ecosystem function (Bucharova et al., 2021) and support biodiversity across trophic levels (Burghardt & Tallamy, 2013).

Thus far, a key element missing from the restoration practitioner's toolbox is an accessible decision‐support tool that incorporates species trait information and habitat requirements in a geographical context, such that restoration practitioners can easily create species lists – or “seed menus” – based on restoration project locations (but see M’Gonigle et al., 2017). A well‐crafted seed menu can predict suitable species based on their habitat distribution and the environmental characteristics of a restoration site, while also providing species attribute information so that practitioners can emphasize functional traits, pollinator diversity, rapid growth, or other species characteristics in their restoration designs. Although seed menus help to identify suitable native species, one complication is that they do not account for local adaptation. Most desert species are adapted to a particular set of environmental conditions at the population level (Baughman et al., 2019) including climate, soil characteristics, and pollinator associations. Introduction of maladapted genotypes into local populations can have negative consequences such as outbreeding depression or reproductive failure (Hufford & Mazer, 2003; McKay et al., 2005). However, decision‐support tools exist to guide seed transfer decisions in the Mojave and elsewhere (Shryock et al., 2018). We propose an integrated workflow that includes tools to select and prioritize species for a given restoration site as well as to identify appropriate seed sources from each species to account for local adaptation or facilitate alternative seed sourcing designs such as genetic admixture (Broadhurst et al., 2008) or predictive seed sourcing/assisted migration (Breed et al., 2013).

Recently, in partnership with the Bureau of Land Management's Mojave Desert Native Plant Program, Esque et al. (2021) developed a priority native plant species list (hereafter “Mojave PSL”) for the Mojave Desert based on a variety of species traits and wildlife services. This list establishes species targets for large‐scale seed collection programs (e.g., the National Seed Strategy, Seeds of Success) to prioritize the development of native plant materials and seed reserves for future restoration needs in the Mojave. Here, we extend the utility of the Mojave PSL by providing a spatially explicit decision support tool that generates seed menus for project sites in the Mojave Desert. Our new application, Mojave Seed Menus, draws from presence‐only species distribution models (hereafter SDMs) to predict suitable habitat for 49 species from the Mojave PSL. These models predict where species are likely to occur based on climate, topography, or other natural features associated with species occurrence records. By spatially stacking SDMs, we generate interactive lists of priority plant species for any given location within the Mojave. Moreover, Mojave Seed Menus pairs habitat predictions with species attribute information from the Mojave PSL, including life‐history, bloom and flowering traits, pollinator associations, propagation techniques, importance as forage or cover for the Mojave desert tortoise (*Gopherus agassizii*), and response to disturbance (Esque et al., 2021). We describe how this novel spatial decision‐support tool can be used to create detailed seed menus for Mojave restoration projects, as well as integrate with existing tools for genetically informed seed transfer designs.

## 2 METHODS

### 2.1 Study site

The Mojave Desert spans approximately 150,000 km^2^ in the southwestern United States. This warm desert ecoregion is characterized by north to south trending mountain ranges and interlaying basins (MacMahon, 1988). Elevations range from below sea level in Death Valley to over 3000 m in the Panamint Range and Spring Mountains. Alluvial fans and washes form along mid to lower elevation slopes and contribute to the accumulation of fine particles and salinity in lower basins, forming playas in closed basins. Annual precipitation varies along elevational gradients but averages approximately 135 mm, with much of this occurring during the winter months (Hereford et al., 2006). However, summer precipitation increases along a longitudinal gradient, with higher quantities recorded in the eastern Mojave due to summer tropical storms. As with precipitation, temperatures vary along elevation gradients and range from <0°C in winter to over 50°C in summer at low elevations. Mean annual temperature is approximately 17°C.

### 2.2 Study species

We selected 49 species for SDMs (Appendix S1) based on their restoration importance and inclusion in the recent Mojave PSL. The species selected here for habitat modeling are a subset of those included in the full Mojave PSL, but include representatives from different growth forms and lifespans, as well as foundational species. Selected species promote overall community recovery from disturbance by providing favorable microsites and attracting animals to increase diversity, such as creosote bush (*Larrea tridentata*) and the Joshua tree (*Yucca brevifolia* and *Y*. *jaegeriana*) (Hurd & Linsley, 1975; Miller & Stebbins, 1975; respectively).

### 2.3 Environmental variables

We derived 14 environmental variables to serve as covariates in SDMs, which together characterize climate, topography, plant canopy, and soil surface properties for the Mojave Desert (Table 1). Precipitation and temperature were extracted at collection sites using ClimateNA v. 6.2 (Wang et al., 2016), which downscales PRISM data (Daly et al., 2008) and corrects for elevational variation. Satellite metrics incorporated plant canopy and soil surface data from the moderate‐resolution imaging spectroradiometer (MODIS) satellite averaged across a minimum of ten years (NDVI amplitude and maximum – USGS eMODIS Remote Sensing Phenology, https://doi.org/10.5066/F7PC30G1; other metrics – Inman et al., 2014). Topographic metrics were calculated by aggregating a 30 m digital elevation model at a 1 km^2^ resolution for modeling (National Elevation Dataset, https://www.usgs.gov/programs/national‐geospatial‐program/national‐map).

**TABLE 1:** Environmental covariates used to fit SDMs for plant species in the Mojave Desert

Environmental variable	Code	Definition
Climate		
Summer precipitation (mm)	SP	Average precipitation received from May to Oct
Winter precipitation (mm)	WP	Average precipitation received from Nov to April
Summer maximum temperature (°C)	*T*_max_	Maximum temperature of warmest month
Winter minimum temperature (°C)	*T*_min_	Minimum temperature of coldest month
Annual temperature range (°C)	*T*_range_	Average of the monthly temperature ranges (monthly maximum minus monthly minimum)
Annual heat/moisture index	AHM	(MAT + 10)/(MAP/1000)
Climatic moisture deficit	CMD	Difference between potential evapotranspiration (PET) and actual evapotranspiration (AET; Dobrowski et al., 2013)
Satellite metrics		
NDVI amplitude	AMP	Maximum increase in canopy photosynthetic activity above the baseline averaged for the period 2003–2017
NDVI maximum	MAXN	Maximum level of photosynthetic activity during the growing season averaged for the period 2003–2017
Soil water stress	SWS	Mean of the Shortwave and Infrared Water Stress Index (SIWSI; Fensholt & Sandholt, 2003) from 2001–2010 (Inman et al., 2014).
Surface texture	ATI	Difference in mean daytime and nighttime surface temperatures for 2001–2010 (Inman et al.,^2014^)
Topography		
Heat load index	HLI	Aspect/slope transformation index (McCune & Keon, 2002) representing the range in heat load from coolest (northeast slope) to warmest (southwest slope)
Slope (°)	Slope	Slope in degrees
Topographic position index	TPI	Steady‐state wetness index expressed as a function of slope and upstream contributing area

### 2.4 Species distribution modeling

We used an ensemble modeling approach to create SDMs for 49 native plant species throughout their Mojave Desert ranges. We used a custom R script to control pseudo‐absence selection and model evaluation and to implement parallel processing and model‐averaged response curves. As input data for the SDMs, we assembled species occurrence records from a variety of sources including public databases (Consortium of California Herbaria – http://ucjeps.berkeley.edu/consortium/; SEInet – https://swbiodiversity.org/seinet/), vegetation classification studies (National Park Service vegetation inventory products, https://www.nps.gov/im/vmi‐products.htm), U.S. Bureau of Land Management Seeds of Success collections, and U. S. Geological Survey datasets (Webb et al., 2003). Prior to modeling, all occurrences were visually assessed for georeferencing errors and masked from water bodies. Additionally, we excluded occurrences with positional uncertainty larger than 1 km when noted in the metadata. Occurrences for each species are mapped in Appendix S1.

Our ensemble modeling approach included three algorithms: generalized additive models (R package “mgcv” version 1.8–22; Wood, 2017), random forests (R package “randomForest” version 4.6–12; Liaw & Wiener, 2002), and MaxEnt version 3.3.3k (as implemented in R package “dismo” version 1.1–4; Hijmans et al., 2017; Phillips et al., 2006). We chose to average predictions across different types of algorithms because the choice of algorithm is the largest source of variability in SDM predictions (Watling et al., 2015) and because multi‐model ensembles broaden the types of response functions that can be identified (Araújo & New, 2007). For each individual algorithm, we generated models reflecting all combinations of the 14 environmental variables (Table 1) while restricting the total number of terms within any one model to six to avoid overfitting. Correlated variables (*r* > |0.7|) were not included in the same models. Due to the lack of surveyed absence points, we created random selections of pseudo‐absences following the recommendations in Barbet‐Massin et al. (2012) for each algorithm. To account for patterns of spatial aggregation/unequal sampling effort in the presence points, which can bias model predictions (Veloz, 2009), we first rasterized presences to the modeling resolution (1 km^2^) and subsequently applied a spatial thinning procedure (grid sampling) in which a maximum of three points could be sampled from any 10 km^2^ area (Fourcade et al., 2014). Each model was fit across a series of 50 cross‐validation runs, with each run consisting of a random sample of pseudo‐absences and spatially thinned presence points. For each cross‐validation, a random 20% sample of points was withheld for model evaluation. All GAM models were fit with restricted maximum likelihood (REML) and an extra penalty allowing smooth terms to be penalized to zero (“gam” option select=TRUE in “mgcv” package) to aid model selection. Random forest models were fit with 1000 random trees. MaxEnt models were fit with 10,000 pseudo absences and program defaults.

We considered several metrics of model prediction accuracy to select a candidate list of approximately 10 well‐performing models for each algorithm (30 total candidate models): AUC (i.e., the area under the receiver operating characteristic; Fielding & Bell, 1997), the Boyce Index (Hirzel et al., 2006), and the True Skill Statistic (TSS; Allouche et al., 2006). For GAM and MaxEnt models, we also calculated each model's average AIC (with each model being fit to the same subsets of data) to help identify well‐performing, parsimonious models. AIC values for Maxent models were calculated using the “ENMeval” package in R (Muscarella et al., 2014), which follows the approach developed by Warren and Seifert (2011). To aid model interpretation, we derived relative importance values for each predictor present in the candidate models for each algorithm (Appendix S1). Maxent relative importance values were based on the default permutation importance output for each predictor (Phillips et al., 2006). Relative importance for predictors in random forest models was based on the mean decrease in accuracy from permutations leaving out each term (“importance” function in the R package randomForest; Liaw & Wiener, 2002). For GAM, we used the predictor's average expected degrees of freedom (edf) across all candidate GAM models in which the predictor appeared as the measure of relative importance. We also derived partial variable response curves for each of the top nine predictors present in the candidate models for each species. These curves indicate the shape and direction of relationships between predictors and habitat probability values. For GAM and Maxent models, response curve functions for predictors were averaged across all of the models in which each predictor occurred: in Appendix S1, these model‐averaged curves are overlaid on the individual response curves from candidate models including each predictor. For random forest models, we used the default response curves (“partialPlot” function) fitted to a model with the top nine predictors.

Raster surfaces representing SDM predictions from each model were generated by averaging model predictions across the 50 cross‐validation runs (all surfaces were generated with the “predict” function of the R package “raster”). Next, ensemble predictions for individual algorithms were generated by taking the weighted average among candidate model predictions for each algorithm based on TSS scores, resulting in three ensemble algorithm predictions. For each species, we also calculated a standard error layer based on variation across all candidate models included in the ensemble. Finally, an overall ensemble SDM prediction was generated by taking the average of the three individual algorithm ensembles.

#### 2.4.1 Evaluation of systematic model bias

In largely unpopulated regions of the Mojave Desert, species occurrence records may be biased toward areas with easier human access (e.g., near roads or other developed features), and a pattern of unequal sampling could bias SDM model performance and evaluation (Fourcade et al., 2014; Veloz, 2009). Although our use of occurrence records from vegetation classification and other research studies may partially alleviate this issue, we sought to evaluate systematic spatial sampling bias. To do so, we used a 1 km^2^ resolution terrestrial development index created for the Western United States (Carr & Leinwand, 2020; Carter et al., 2017) to derive a spatial layer reflecting distance from roads and other developed features. Next, we created spatial layers reflecting the overall mean of the habitat probabilities across all 49 individual species SDMs, as well as the mean of the standard error layers for each species (hereafter referred to as “aggregated habitat probabilities” and “aggregated model standard errors”, respectively). We then assessed whether the aggregated spatial patterns in SDM habitat probabilities and/or model standard errors were associated with distances to developed features, as might occur if there were strong systematic bias in the model suite. To allow for non‐linear associations, we fit generalized additive models in the R package “mgcv” (Wood, 2017) with the default thinplate splines and evaluated models based on these models’ coefficients of determination.

### 2.5 Mojave Seed Menus application

We developed an interactive spatial decision support tool, Mojave Seed Menus, as a “shiny application” coded using the R package “shiny” v.1.5.0, which generates interactive web pages or dashboards paired with the analytical capabilities of R (Chang et al., 2020). Our application also supports an interactive online map generated using the leaflet package (Cheng et al., 2019) for dynamic user input. The core function of Mojave Seed Menus is to overlay SDMs for species of restoration importance and extract their habitat probability values (probabilities range from 0 to 1, with higher values indicating higher probability of occurrence) based on user input coordinates provided as spreadsheets, shapefiles, or map clicks. Species habitat values for each potential project site are paired with species attribute values from the Mojave PSL, including life‐history, disturbance ecology, pollinator interactions, and propagation techniques. The application outputs a downloadable “seed menu” table with species predicted to have suitable habitat at a given restoration site(s), along with each species’ attribute information. The application also makes available the entire species guide presented in Esque et al. (2021). Used in combination with other restoration tools, e.g., provisional seed transfer zones or climate distance projections (Shryock et al., 2018), the Mojave Seed Menus application presents a powerful new tool for restoration practitioners.

## 3 RESULTS

### 3.1 Species distribution models

Our ensemble modeling approach produced SDMs that performed well on average, with AUC ranging from a low of 0.82 for *Ambrosia dumosa* to a high of 0.97 for *Lupinus odoratus*, and averaging 0.88 across all species (Table 2). Somewhat counterintuitively, we obtained lower AUC scores for several of the most common species including *A*. *dumosa* and *Larrea tridentata*. However, this is likely due to these species having particularly broad ranges within the Mojave Desert mapping extent, such that random pseudoabsences would more frequently fall within suitable habitat than for species inhabiting a narrower range of conditions. A complete set of species maps is available in Appendix S1, while habitat layers are provided both within Mojave Seed Menus and as a separate U.S. Geological Survey data release (Shryock et al., 2022b).

**TABLE 2:** SDM model performance and relative importance of environmental predictors for Mojave Desert native plant species

Species	*n*	Model performance		Relative importance of model terms												
		AUC	TSS	AHM	CMD	WP	SP	*T*_max_	*T*_min_	*T*_range_	HLI	Slope	TPI	Text	AMP/MAXN^b^	SWS
Cacti																
*Echinocereus engelmannii*	505	0.901	0.699	13.68	3.71	7.13	7.60	7.69	5.53	14.24	0.00	0.91	9.92	14.60	10.80	4.17
*Opuntia basilaris*	967	0.852	0.571	5.82	11.81	9.25	7.14	5.56	8.60	12.28	0.00	8.96	10.25	14.66	2.17	3.52
Forbs^a^																
*Acmispon humistratus*	196	0.888	0.678	6.95	7.04	2.22	2.08	12.99	13.60	11.50	0.48	6.55	9.00	0.46	27.14	0.00
*Acmispon strigosus*	352	0.902	0.677	7.72	3.70	14.98	14.82	8.09	10.58	18.85	0.00	6.95	5.50	3.60	5.21	0.00
*Amsinckia tessellata*	889	0.828	0.538	9.82	0.00	7.21	17.46	9.68	12.69	10.35	0.94	3.78	7.13	4.65	14.95	1.33
***Asclepias erosa***	108	0.894	0.733	8.26	2.62	15.25	7.85	5.79	16.67	14.05	6.93	11.43	2.16	7.56	0.81	0.64
*Astragalus didymocarpus*	152	0.884	0.694	9.48	0.96	6.79	22.75	17.84	12.29	7.65	3.93	0.75	5.29	8.86	2.69	0.71
***Astragalus layneae***	168	0.922	0.76	18.14	3.20	4.96	16.89	22.43	1.07	3.54	0.64	3.89	9.66	6.78	4.35	4.45
***Baileya multiradiata***	242	0.902	0.688	8.40	7.58	5.64	29.67	8.11	12.56	6.78	1.67	3.19	9.29	4.92	2.19	0.00
*Chylismia brevipes*	806	0.858	0.611	14.38	4.99	10.89	21.51	6.00	7.00	5.18	0.48	6.27	4.91	13.35	3.09	1.94
*Cryptantha micrantha*	513	0.857	0.594	8.87	3.39	16.92	10.92	8.45	12.80	13.95	0.55	3.23	6.97	6.17	7.79	0.00
*Cryptantha nevadensis*	880	0.837	0.549	8.24	6.12	13.61	22.87	4.55	10.99	7.58	1.09	9.67	7.35	0.00	6.12	1.81
*Descurainia pinnata*	740	0.86	0.602	5.91	4.92	13.29	8.08	6.53	11.00	16.63	0.19	7.62	6.49	5.28	14.08	0.00
***Eriogonum inflatum***	1395	0.858	0.584	6.51	7.45	7.71	10.35	6.50	6.27	5.79	1.61	21.25	6.28	6.88	4.99	8.40
***Euphorbia albomarginata***	484	0.886	0.659	8.83	4.09	14.55	8.76	9.70	14.06	19.35	4.15	2.98	3.94	3.67	3.27	2.65
*Lepidium lasiocarpum*	1210	0.863	0.606	3.11	11.16	5.87	19.39	8.81	11.87	13.00	2.79	3.48	2.86	7.96	9.70	0.00
*Lupinus odoratus*	45	0.97	0.919	9.97	5.68	13.09	22.51	14.41	0.00	0.00	0.00	7.93	9.52	16.88	0.00	0.00
*Malacothrix glabrata*	744	0.837	0.554	14.33	3.47	14.26	13.97	5.72	7.97	11.64	1.73	2.87	10.59	3.88	5.89	3.67
***Mirabilis laevis***	341	0.893	0.669	6.93	10.60	3.57	13.25	11.70	0.48	18.76	0.00	10.92	1.82	14.05	5.09	2.83
***Oenothera cespitosa***	94	0.944	0.814	6.59	1.75	14.37	10.15	27.20	8.74	6.74	1.32	4.12	3.58	15.43	0.00	0.00
*Oenothera deltoides*	299	0.916	0.759	11.81	12.21	5.02	11.51	5.35	18.49	3.07	12.84	7.71	4.91	1.39	4.89	0.80
*Oenothera primiveris*	88	0.898	0.701	2.96	20.82	4.50	9.05	8.20	12.68	2.09	3.21	13.77	15.97	2.98	3.09	0.69
***Penstemon palmeri***	203	0.922	0.775	5.84	3.46	7.77	30.51	10.27	5.51	0.00	1.59	6.39	9.91	16.49	2.27	0.00
*Plantago ovata*	1208	0.862	0.588	10.68	6.81	6.34	7.59	22.63	10.58	4.39	2.06	5.70	9.93	6.70	2.36	4.23
*Salvia columbariae*	687	0.864	0.596	7.31	3.59	5.38	14.98	11.18	10.62	12.13	1.67	10.80	3.73	7.94	8.55	2.12
***Sphaeralcea ambigua***	1458	0.831	0.528	16.55	3.30	5.23	13.02	12.92	3.76	12.18	1.44	0.63	8.12	12.55	2.82	7.48
*Stephanomeria exigua*	222	0.899	0.712	1.32	4.81	16.84	10.56	5.41	6.57	28.60	0.88	3.32	15.71	1.76	3.48	0.74
Grasses																
*Achnatherum hymenoides*	530	0.836	0.57	8.76	4.19	5.13	4.38	10.80	16.22	17.96	4.05	5.65	11.21	6.32	3.99	1.34
*Hilaria rigida*	658	0.871	0.611	5.22	11.10	7.86	14.76	7.19	11.47	15.20	1.77	6.22	2.57	4.93	9.89	1.82
*Muhlenbergia porteri*	418	0.915	0.733	11.46	7.01	5.96	23.61	7.65	10.56	16.40	0.00	0.79	0.32	7.19	8.25	0.78
*Vulpia octoflora*	532	0.868	0.605	3.35	8.94	9.35	9.21	9.90	5.30	12.15	4.54	16.65	13.49	1.43	0.00	5.68
Shrubs																
*Ambrosia dumosa*	2524	0.822	0.496	21.45	6.53	7.71	15.43	5.98	4.60	8.27	2.61	7.62	9.30	4.44	6.07	0.00
*Ambrosia salsola*	1280	0.831	0.531	16.83	6.40	11.18	13.55	9.85	4.54	10.11	0.27	3.43	10.90	4.85	8.09	0.00
*Atriplex hymenelytra*	305	0.92	0.742	0.00	5.51	0.83	19.42	17.60	10.90	12.11	0.77	13.77	2.92	4.86	7.26	4.05
*Encelia farinosa*	892	0.896	0.659	0.00	14.74	3.56	9.66	5.97	9.23	6.93	2.49	11.54	6.00	22.34	4.87	2.66
*Ephedra nevadensis*	1972	0.84	0.557	15.78	5.72	9.41	9.26	13.06	7.21	13.40	1.16	7.51	6.10	3.83	5.54	2.02
*Ericameria cooperi*	433	0.921	0.727	10.48	0.00	7.19	14.46	24.13	7.02	10.56	1.45	4.79	4.76	3.59	8.96	2.62
*Eriogonum fasciculatum*	1098	0.872	0.6	14.07	4.52	9.87	9.59	10.14	3.36	10.40	4.62	8.85	3.28	10.31	9.33	1.66
*Krameria bicolor*	272	0.92	0.732	3.75	13.60	9.45	8.08	7.26	12.26	12.89	1.10	9.29	3.39	18.93	0.00	0.00
*Krameria erecta*	798	0.885	0.637	8.25	12.89	5.38	14.47	6.84	11.69	8.83	0.00	8.17	3.86	8.76	10.86	0.00
*Larrea tridentata*	2337	0.833	0.521	17.52	8.65	11.66	14.03	7.49	4.01	3.10	1.82	7.85	10.97	4.78	8.12	0.00
*Lycium andersonii*	653	0.861	0.583	4.76	6.20	11.35	17.20	11.12	9.70	19.77	1.41	0.84	1.49	4.12	6.28	5.77
*Lycium cooperi*	306	0.88	0.638	10.85	12.39	1.83	14.40	19.09	11.44	14.18	0.51	5.41	6.01	0.00	3.89	0.00
*Lycium pallidum*	131	0.902	0.698	1.30	24.08	11.61	14.27	1.06	14.66	10.34	1.37	4.01	2.23	7.16	0.00	7.91
*Psorothamnus fremontii*	444	0.894	0.672	9.24	4.83	10.46	16.23	12.72	11.90	9.40	0.75	12.51	6.26	3.39	0.00	2.31
*Stephanomeria parryi*	100	0.926	0.815	8.29	3.34	5.07	19.68	21.26	0.00	20.69	0.00	8.14	2.14	5.65	2.31	3.42
Trees/Arborescents																
*Chilopsis linearis*	330	0.9	0.688	0.00	20.18	19.65	4.70	5.16	4.60	4.47	1.72	0.72	8.84	19.62	6.20	4.13
*Yucca brevifolia* ^c^	1633	0.875	0.622	10.11	3.70	5.09	10.52	8.11	12.64	15.24	0.16	6.97	9.27	4.10	4.61	9.49
*Yucca schidigera*	1123	0.884	0.668	10.26	7.38	5.82	15.88	6.16	7.76	11.81	0.00	11.91	3.79	13.13	6.09	0.00
**Average**		0.881	0.652	8.78	7.17	8.74	13.84	10.46	9.06	11.11	1.73	6.89	6.73	7.62	5.68	2.20

^a^ Perennial forbs are in bold.

^b^ MAXN was used to represent canopy photosynthetic activity in models including perennial species, while AMP was used to represent vegetation green‐up potential in models including annual species.

^c^ This species has two recognized varieties in the ITIS, var. *brevifolia* and var. *jaegeriana*, of which the former occurs predominantly in the western Mojave Desert and the latter in the eastern Mojave, with limited transitional areas in southern NV. However, Lenz (2007) reassessed *Y*. *brevifolia* and *Y*. *jaegeriana* as distinct species.

In terms of environmental variable relative importance, we found that temperature generally outweighed precipitation, with the temperature variables (*T*_max_, *T*_min_, and *T*_range_) showing higher relative importance in aggregate than the precipitation variables (WP and SP) for 39 of 49 species (Table 2). However, given that temperature and precipitation interact to determine the overall aridity of a site, it may be difficult to disentangle these effects. Among the individual climate variables, the amount of summer precipitation (SP) had the greatest relative importance across species (13.84), followed by annual temperature range (11.11). Soil surface texture had the highest average relative importance (7.61) among variables representing topographic and surface characteristics, followed by slope (6.89). We did not observe obvious differences in the relative importance of environmental variables among different growth forms or lifespans, although uneven representation from these groups (e.g., we only considered a low number of cacti and grasses) likely reduces our ability to detect such differences. Response curves for all species are provided in Appendix S1.

#### 3.1.1 Evaluation of systematic model bias

**FIGURE 1:**
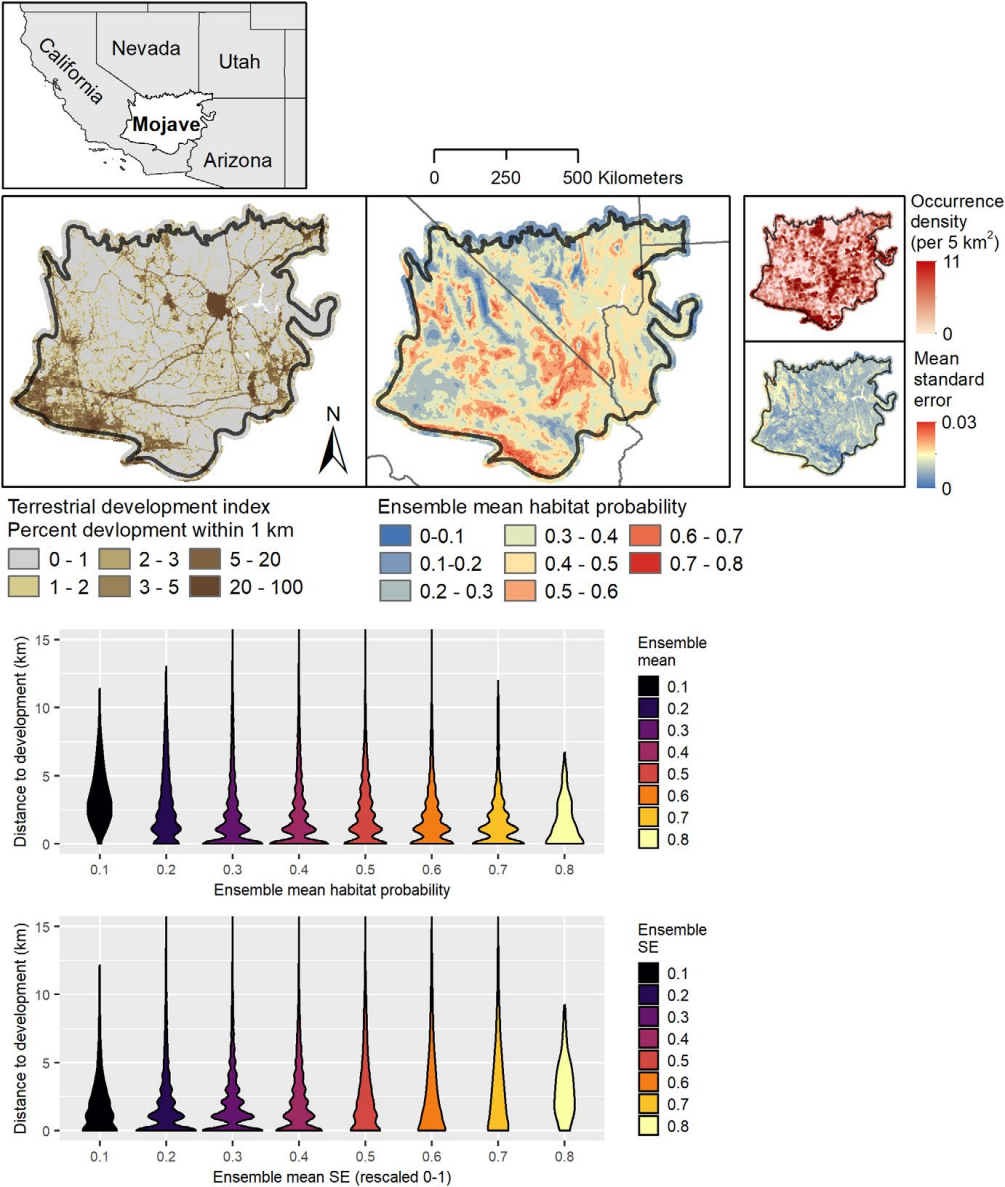
Evaluation of systematic model bias across SDMs of Mojave Desert plants. We compared a distance‐to‐development layer calculated from the terrestrial development index (Carr & Leinwand, 2020) with aggregated habitat probabilities and aggregated model standard errors for 49 SDMs. Violin plots display the association between distance‐to‐development and aggregated habitat probabilities/standard errors (binned into eight classes). The overall density of occurrence records per square km is also displayed (top right)

We did not find strong evidence for systematic model bias across the SDMs based on associations between aggregated habitat probabilities, aggregated standard errors, or distance to development. Graphs of the distance to development among binned habitat probability values indicated that habitat probabilities were slightly lower farther from developed areas (Figure 1). However, in GAM models, distance to development explained less than one percent of the variation in aggregated habitat probabilities. For aggregated model standard errors, somewhat higher values were associated with larger distances to development (Figure 1). However, this pattern was again not strong enough to explain more than one percent of the variation in aggregated standard errors in GAM models.

### 3.2 Seed Menu application

**FIGURE 2:**
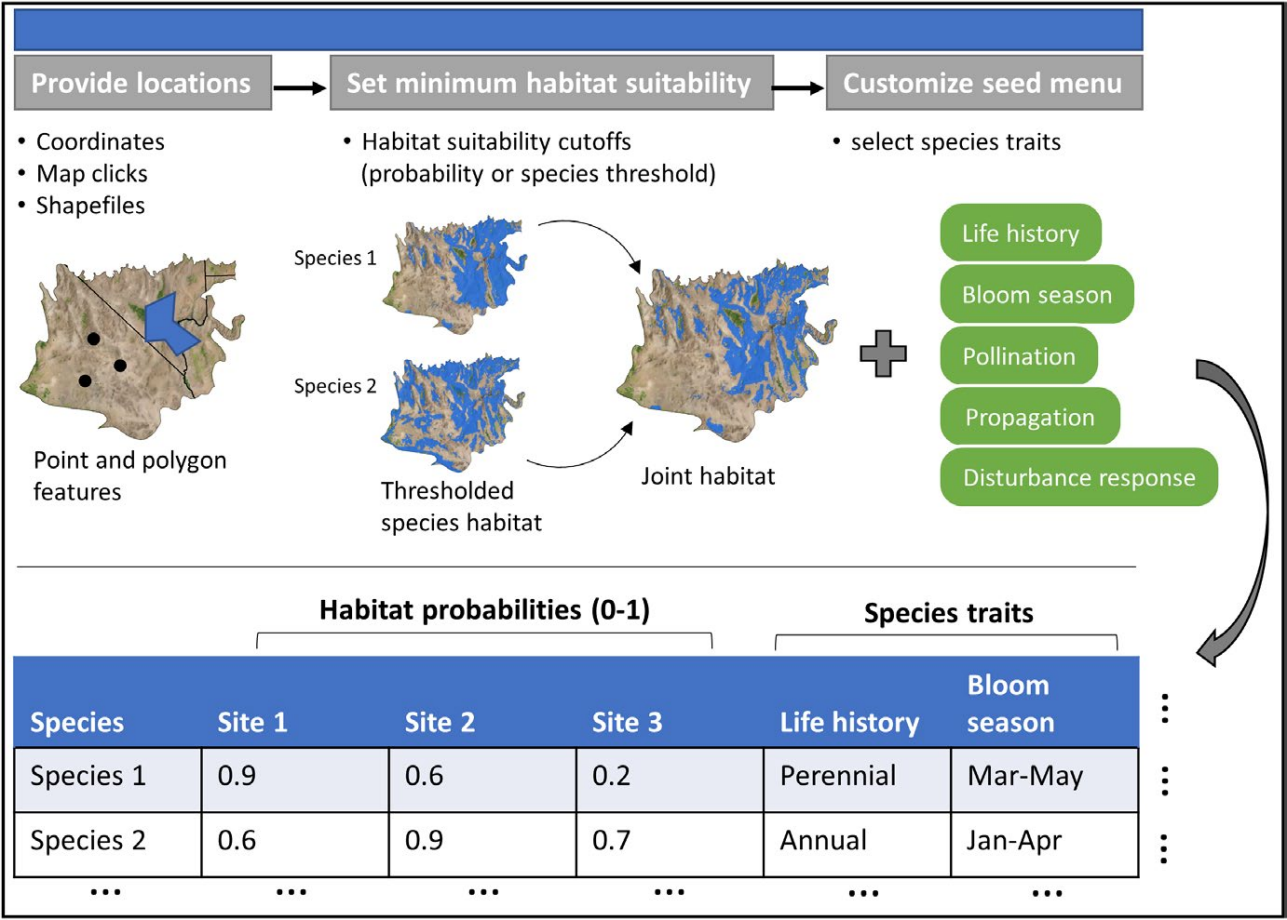
Flowchart of operations used by Mojave Seed Menus to create species lists for restoration sites. Users can supply restoration site locations as coordinates, map clicks in a browser, or point/polygon shapefiles. Next, users can specify a habitat suitability threshold if desired. This value determines the minimum probability of occurrence necessary for a species to be recommended at an input site (e.g., if the threshold is set at 0.4, all species will have an SDM occurrence probability of 0.4 or higher at an input site in the final seed menu). The application will then extract habitat probabilities (accounting for user‐specified thresholds) from spatially stacked SDMs and pair these habitat probabilities with user‐selected species traits in a downloadable seed menu table

We developed an interactive web application to aid restoration practitioners in creating seed menus for restoration sites. The application, “Mojave Seed Menus”, pairs predicted habitat suitability values for priority native plant species with species attribute information useful for restoration planning at user‐defined locations (Figure 2). Results from the application are provided to users in downloadable table format. Mojave Seed Menus will be freely available over the web (https://rconnect.usgs.gov/MojaveSeedMenu/) and will not require users to install special software or create a user account. The application will also be available as stand‐alone software for users who wish to run Mojave Seed Menus locally through RStudio (Shryock et al., 2022a; https://doi.org/10.5066/P94A2QLK). A list of dependencies for the stand‐alone software version is provided in the linked repository.

**FIGURE 3:**
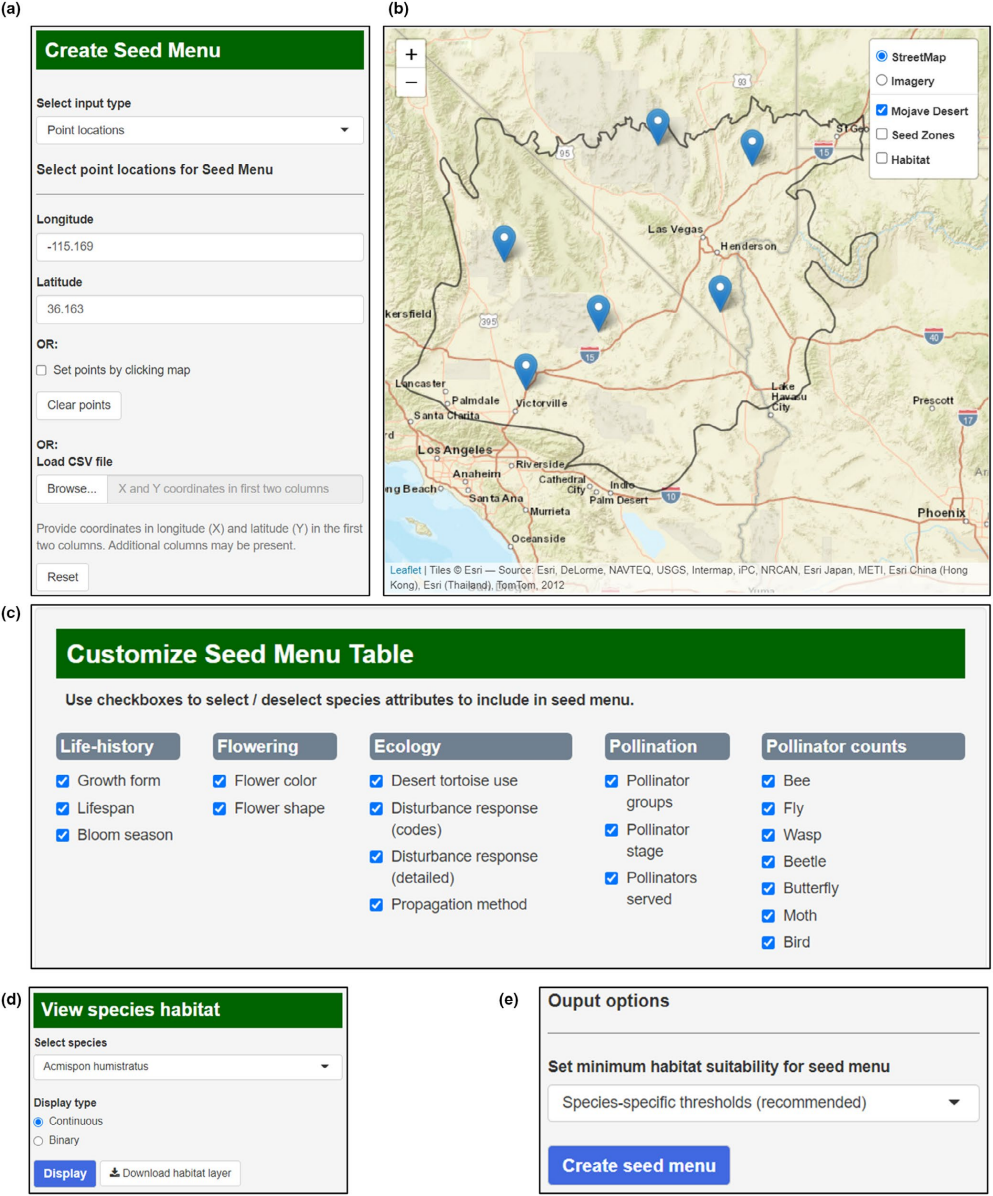
User interface for the Mojave Seed Menus Shiny application. (a) The “create seed menus” dialogue box directs users to upload restoration site locations as coordinates, map clicks, or shapefiles. (b) The online map displays current locations input and can be used to create input sites via map clicks. (c) The “Customize seed menu table” dialogue lets users select which species traits to include in outputs. (d) Users can also display individual species SDMs on the map through the “View species habitat” dialogue. (e) In the output options dialogue, users can control the habitat probability level needed for a species to be recommended at input sites

We illustrate several key features of Mojave Seed Menu's user interface (Figure 3a–e), including the location input menu, interactive online map, seed menu customization menu, and options for viewing species habitat models and setting habitat probability thresholds for species inclusion in seed menus. Users have several options for selecting the locations (e.g., restoration project sites) for generating seed menus, including uploads from coordinates tables, point or polygon shapefiles, or by zooming in and clicking the online map (Figure 3a, b). Users can also select which species attributes they would like to appear in the table (Figure 3c) and display individual species habitat models in the online map viewer (Figure 3d).

The minimum habitat suitability threshold parameter allows users to select the minimum probability of occurrence allowable in order for a species to be recommended for an input site (Figure 3e), based on the SDMs for each species. For example, if the user selects 0.4 as the threshold, then all species with an SDM occurrence probability value ≥0.4 will be included in the Seed Menu table for that site. When multiple sites are input, setting the threshold parameter to 0.4 would require that all species included have an occurrence probability value ≥0.4 at all input sites. The dropdown menu also includes options for selecting species‐specific thresholds. In this case, habitat suitability value thresholds have been determined separately for each species based on their SDM model sensitivities (proportion of presences correctly predicted) and/or specificities (proportion of absences correctly predicted). For example, the “maximum (sensitivity + specificity)” option provides thresholds that maximize the sum of model sensitivity and specificity for each species.

**FIGURE 4:**
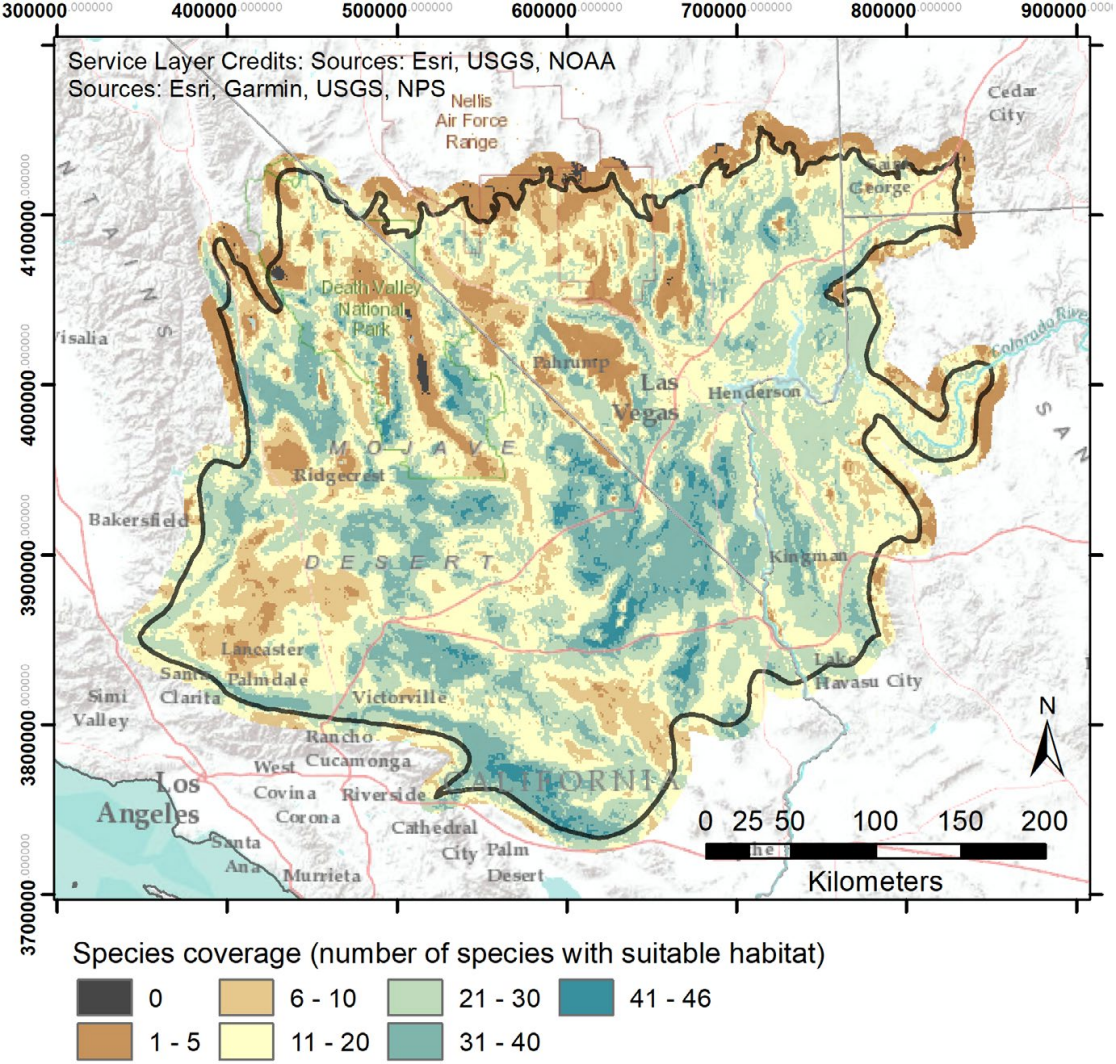
Total species coverage provided in the Mojave Seed Menus application, indicating how many species have suitable habitat in different parts of the Mojave based on the initial species list. Species with suitable habitat can be included in seed menus for a given location

Based on the initial group of 49 species, Mojave Seed Menus provides strong coverage throughout most of the Mojave Desert (Figure 4). The vast majority of the Mojave Desert is represented by more than 5 modeled priority plant species (i.e., a seed menu created anywhere in the Mojave would likely contain 5 or more recommended plant species). Only scattered and environmentally extreme areas (e.g., lower Death Valley and the highest mountain areas) provide coverage for fewer species.

Detailed online instructions are included with the Mojave Seed Menus application to facilitate proper use. Mojave Seed Menus also provides a web version of the Mojave Desert priority species guide developed in Esque et al. (2021). This guide contains a wealth of information for restoration practitioners, including detail on species propagation, production, cultivation, and recoverability, as well as species importance for the Mojave desert tortoise (*Gopherus agassizii*). Further, Mojave Seed Menus will display the provisional seed transfer zones for the Mojave Desert developed in Shryock et al. (2018) as a guide for identifying genetically appropriate seed sources.

## 4 DISCUSSION

Faced with increased development and unprecedented ecosystem stressors, restoration practitioners in the Desert Southwest must balance a need to act with the often‐limited commercial supply of native seeds (Johnson et al., 2010; Peppin et al., 2010) until regionally adapted germplasm is developed for seed increase by commercial growers in the ecoregion. Programmatic directives, national policy, and restoration science all point toward the fundamental importance of promoting native species assemblages, providing habitat for pollinator communities, and maintaining genetic diversity (Olwell & Riibe, 2016; Vilsack & McCarthy, 2015). Meeting these objectives requires careful planning and prioritization among various stakeholders, including government agencies, non‐profits, universities, and commercial seed suppliers. Accessible decision support tools are pivotal to this effort and have already been deployed to guide seed transfer decisions, reducing the risks of maladaptation or loss of genetic diversity (Massatti et al., 2018; Shryock et al., 2017, 2018). However, restoration practitioners must also select a mix of species to seed in degraded areas, a choice that is not trivial given the fundamental role of community assembly on numerous ecological processes (Oliver et al., 2015). To support effective seed mix designs in the Mojave Desert, we developed Mojave Seed Menus, a spatial application that pairs species distribution models (SDMs) for priority native plant species with species trait data, giving restoration practitioners and resource managers an interactive platform to plan seed mixes that can be customized to match project objectives.

Mojave Seed Menus is currently based on a dataset of 49 SDMs for priority plant taxa identified in the Mojave PSL (Esque et al., 2021) using numerous criteria, including their importance as forage or cover for the Mojave desert tortoise (*Gopherus agassizii*), associations with various pollinators, ability to colonize disturbed areas and/or compete with invasive species, and other metrics. Although presence‐only SDMs have known biases, in particular spatial bias due to aggregation of occurrence records near more easily accessed areas (Fourcade et al., 2014; Veloz, 2009), we did not detect obvious patterns of systematic bias across our SDMs. One might expect habitat suitability predictions from SDMs to show a trend of increasing habitat probabilities near roads or other developmental features if occurrence records were aggregated near such areas rather than more remote locations. However, we did not detect a strong association (linear or non‐linear) between aggregated habitat probabilities and the layer representing distance to development, or between aggregated model standard errors and this layer (Figure 1). In part, our SDMs may have been strengthened by our use of species occurrences from vegetation studies in addition to herbarium records, as the former are likely to be less spatially biased. We also used a grid sampling procedure to disaggregate occurrence records prior to modeling, which reduces the impact of unequal sampling effort (Fourcade et al., 2014). Moreover, we used an ensemble SDM approach to increase accuracy by reducing dependence on individual algorithms (Araújo & New, 2007). Overall, our SDMs provide reasonable accuracy based on the model AUC and TSS scores (Table 2) and predict suitable areas for each species to establish given favorable climate conditions. However, as with all SDMs, we note that our models are subject to bias based on the availability of species occurrence records, which may be spatially incomplete or fail to reflect post‐observation temporal habitat changes.

Mojave Seed Menus provides a number of accessible options for users to create interactive seed mixes for restoration projects (Figure 3). To use the application, the only required input is one or more geographic locations (within the Mojave Desert) from which to derive seed menu(s). These can be provided in multiple ways: users can provide coordinates for a single location, upload a spreadsheet with coordinates and other attributes, click on the online interactive map, or upload a point or polygon shapefile (multiple points and polygons are supported, but we recommend against uploading “multipart” shapefiles, in which multiple spatially distinct polygons are treated as a single feature). Shapefiles can be uploaded in any coordinate system recognized by the “rgdal” library in R (Bivand et al., 2020). Once geographic locations are uploaded, users can customize which species traits to include in the seed menu and download the resulting table. In determining which species can be included, users can optionally set a cut‐off threshold to exclude species that do not meet a given habitat suitability threshold or use a species‐specific habitat cut‐off point already provided as a drop‐down menu in the Mojave Seed Menu program (described above in Methods). Currently, Mojave Seed Menus has a coverage of more than 5 species across the vast majority of the Mojave Desert, with many areas represented by over 10 species (Figure 4). This coverage enables restoration practitioners to devise seed mixes emphasizing a particular suite of functional plant traits, pollinator services, or other characteristics. For example, if pollinator services are a priority, practitioners can select species with the highest pollinator counts or that serve as both larval and adult pollinator hosts. For projects in highly denuded areas, species that are known colonizers may be preferable to establish rapid cover. The detailed species accounts provided in the Mojave PSL and through Mojave Seed Menus afford practitioners broad flexibility to set resource targets and project objectives. In future updates, we hope to expand Mojave Seed Menus to include SDMs and trait data for a larger proportion of species described in the full Mojave PSL (Esque et al., 2021).

**FIGURE 5:**
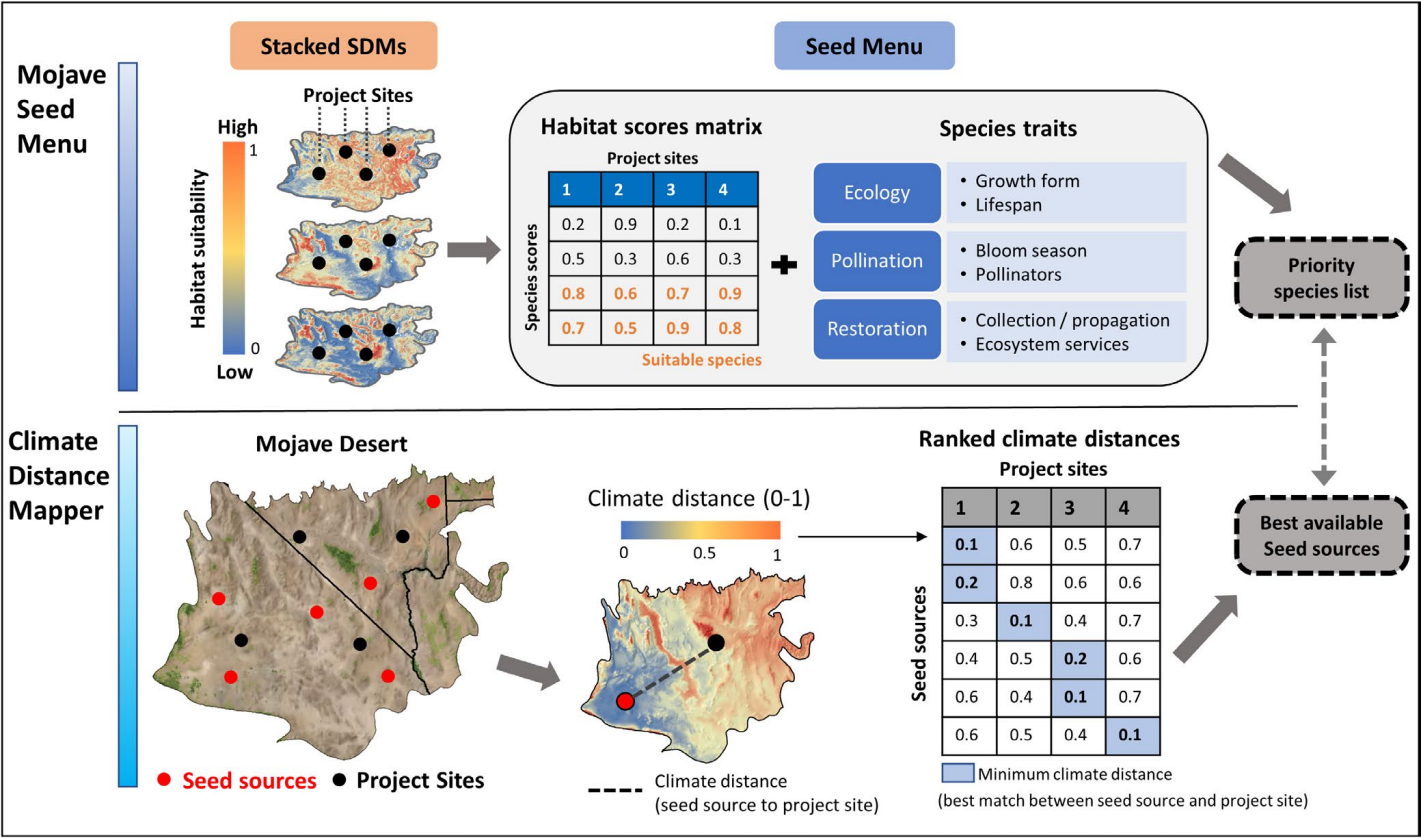
An integrated restoration decision‐support framework for the Mojave Desert, including online applications available through the USGS and BLM Mojave Desert Native Plant Program. In this framework, restoration practitioners and resource managers can first use *Mojave Seed Menus* to generate a list of native plant species given anticipated locations of restoration projects. By providing extensive species trait information, this application facilitates robust seed mix designs that can be customized according to project objectives (e.g., pollinator services, desert tortoise forage, rapid establishment). Once species are selected, *Climate Distance Mapper* can help practitioners identify suitable seed sources from existing stores, or areas to target for future seed collections. Climate Distance Mapper ranks seed sources based on the dissimilarity in climate (climate distance) between seed source and restoration sites and can incorporate future climate scenarios in these calculations. Together, *Mojave Seed Menus* and *Climate Distance Mapper* provide key decision support for prioritization and development of native plant resources to supply future restoration needs

Although species selection and seed mix design are important components of native plant restoration, we emphasize that Mojave Seed Menus is part of an integrated restoration program for the Mojave Desert (Figure 5). A second core component of this program aims to increase seeding effectiveness by accounting for within‐species variation. Local adaptation is widespread among plants in arid regions, leading to intraspecific variation in phenology, growth, emergence, and other traits expressed along gradients of climate and topography (Baughman et al., 2019). It is particularly important to account for local adaptation in heterogeneous regions such as the Mojave, which has both large elevational/climate gradients (Hereford et al., 2006) and an extreme climate that grants species narrow windows for regeneration (Reynolds et al., 2012). Seed transfer zones based on genetic studies (landscape genomics or common gardens) are still a primary approach for generating species‐specific guidelines (e.g., Shryock et al., 2017). When genetics studies are unavailable or pending, then climate distances between seed source and planting sites can serve as a generalized proxy for clines in local adaptation across many species (Shryock et al., 2017, 2018). In partnership with the Mojave Desert Native Plant Program, we previously made available a decision support tool (Climate Distance Mapper; https://rconnect.usgs.gov/Climate_Distance_Mapper) for the Mojave and other southwestern U.S. deserts that allows practitioners to rank seed sources for project sites in both current and future predicted climate (e.g., by minimizing the multivariate climate distance between sites; Shryock et al., 2018). By using this application in tandem with Mojave Seed Menus, practitioners can both create seed mixes for a restoration site and rank alternative seed sources for selected species, thereby decreasing potential for maladaptation in the current and future climates (Figure 5). Given the restoration site location and a table of available seed sources, the example workflow in Figure 5 can be rapidly accomplished. Moreover, if seed sources are unknown, Climate Distance Mapper allows users to create focal‐point seed zones surrounding restoration sites, designating areas to target for future seed collections. With such tools becoming widely accessible, restoration practitioners will have more time to focus on other challenges in desert restoration, including the timing of restoration projects to coincide with favorable conditions (Havrilla et al., 2020), strategies to cope with competition from invasive species that often dominate disturbed areas (Leger et al., 2021; Leger & Goergen, 2017), and propagating species for outplanting that may serve as “resource islands” to facilitate shrubland establishment (Badano et al., 2016; Hulvey et al., 2017). Collectively, the Mojave PSL (Esque et al., 2021), Mojave Seed Menus, and Climate Distance Mapper (Shryock et al., 2018) provide a robust and flexible decision support framework for restoration practitioners to create diverse, resilient, and sustainable native plant communities. In addition, these tools can help resource managers set priority targets for seed collection, production, and cultivation efforts that are necessary to sustain future restoration needs.

## CONFLICT OF INTEREST

The authors declare no conflict of interest.

## AUTHOR CONTRIBUTIONS

**Daniel F. Shryock:** Conceptualization (equal); Formal analysis (lead); Methodology (equal); Software (lead); Writing – original draft (lead); Writing – review & editing (equal). **Lesley A. DeFalco:** Conceptualization (lead); Funding acquisition (lead); Methodology (equal); Project administration (equal); Resources (equal); Supervision (equal); Writing – review & editing (equal). **Todd C. Esque:** Conceptualization (equal); Funding acquisition (equal); Methodology (equal); Project administration (equal); Resources (equal); Writing – review & editing (equal).

## Supporting information

Appendix S1

## Data Availability

Data associated with this manuscript will be available as a USGS data release product on ScienceBase (Shryock et al., 2022b; https://doi.org/10.5066/P9XQJFEL). Software code and documentation for Mojave Seed Menus will be available from the USGS official code repository (Shryock et al., 2022a; https://doi.org/10.5066/P94A2QLK).
