# Supplementary material for: Seed Menus: An integrated decision‐support framework for native plant restoration in the Mojave Desert
Source: Ecol Evol. 2022 Apr 12;12(4):e8805. doi: 10.1002/ece3.8805 (PMC9005930; doi:10.1002/ece3.8805)
Supplement: Supplementary file 1 — Appendix S1 [file ECE3-12-e8805-s001.pdf]

Supplemental information for:

Seed menus: an integrated decision-support framework for native plant restoration in the Mojave Desert.

Daniel F. Shryock, Todd C. Esque, and Lesley A. DeFalco

Appendix S1. Species distribution maps for priority native plant species of the Mojave Desert.

Description

This appendix contains species distribution maps along with response curves for environmental predictors used in models. Species distribution models (SDMs) characterize relationships between species occurrences and the physical environment (e.g., climate, soil, topographic relief) and provide a mechanism for assessing which species may successfully propagate at a restoration site. In conjunction with information on species attributes, SDMs facilitate holistic ecosystem restoration by enabling practitioners to identify diverse, resilient assemblages of native species. This project develops SDMs for native species of fundamental ecosystem importance in order to guide restoration of Mojave Desert landscapes. Each SDM represents a probability distribution of habitat suitability, where values range from 0 (very low probability of species occurrence) to 1 (very high probability of species occurrence). Predictions are based on known occurrence records for each species and are subject to model uncertainty. Hence, appropriate scrutiny should be taken when applying predictions to real-world assessments of habitat or other management applications.

Key to environmental predictors (see manuscript for details):
AHM: annual heat moisture index (includes MAT and MAP)
AMP: increase in photosynthetic activity (NDVI) above the baseline
CMD: climate moisture deficit
HLI: heat load index
MAXN: maximum annual NDVI (long-term MODIS satellite index)
Slope: slope in degrees
SP: summer precipitation (May-Oct)
SWS: shortwave infrared water stress index (MODIS satellite index)
Text: apparent thermal inertia (MODIS satellite index)
Tmax: Maximum summer temperature (Jun-Aug)
Tmin: Minimum winter temperature (Dec-Feb)
TPI: topographic position index
Trange: annual temperature range
WP: winter precipitation (Nov-Apr)
Response curves: each curve depicts the marginal influence of habitat predictors on habitat suitability for a given species. Black lines show the ensemble average, while gray lines show individual models. Histograms display background values taken from a large sample of grid cells in the Mojave Desert.

Contents

1. *Acmispon humistratus* SDM and response curves (GAM, Random Forest, Maxent) 4
2. *Achnatherum hymenoides* SDM and response curves (GAM, Random Forest, Maxent) 8
3. *Acmispon strigosus* SDM and response curves (GAM, Random Forest, Maxent) 12
4. *Ambrosia dumosa* SDM and response curves (GAM, Random Forest, Maxent) 16
5. *Ambrosia salsola* SDM and response curves (GAM, Random Forest, Maxent) 20
6. *Amsinckia tessellata* SDM and response curves (GAM, Random Forest, Maxent)......................... 24
7. *Astragalus didymocarpus* SDM and response curves (GAM, Random Forest, Maxent)................. 28
8. *Asclepias erosa* SDM and response curves (GAM, Random Forest, Maxent)................................ 32
9. *Astragalus layneae* SDM and response curves (GAM, Random Forest, Maxent)........................... 36
10. *Atriplex hymenelytra* SDM and response curves (GAM, Random Forest, Maxent)...................... 40
11. *Baileya multiradiata* SDM and response curves (GAM, Random Forest, Maxent)....................... 44
12. *Chylismia brevipes* SDM and response curves (GAM, Random Forest, Maxent).......................... 48
13. *Chilopsis linearis* SDM and response curves (GAM, Random Forest, Maxent)............................ 52
14. *Cryptantha micrantha* SDM and response curves (GAM, Random Forest, Maxent).................... 56
15. *Cryptantha nevadensis* SDM and response curves (GAM, Random Forest, Maxent)................... 60
16. *Descurainia pinnata* SDM and response curves (GAM, Random Forest, Maxent) 64
17. *Echinocereus engelmannii* SDM and response curves (GAM, Random Forest, Maxent).............. 68
18. *Encelia farinosa* SDM and response curves (GAM, Random Forest, Maxent) 72
19. *Ephedra nevadensis* SDM and response curves (GAM, Random Forest, Maxent) 76
20. *Ericameria cooperi* SDM and response curves (GAM, Random Forest, Maxent) 80
21. *Eriogonum fasciculatum* SDM and response curves (GAM, Random Forest, Maxent) 84
22. *Eriogonum inflatum* SDM and response curves (GAM, Random Forest, Maxent) 88
23. *Euphorbia albomarginata* SDM and response curves (GAM, Random Forest, Maxent) 92
24. *Hilaria rigida* SDM and response curves (GAM, Random Forest, Maxent) 96
25. *Krameria bicolor* SDM and response curves (GAM, Random Forest, Maxent)........................... 100
26. *Krameria erecta* SDM and response curves (GAM, Random Forest, Maxent) 104
27. *Larrea tridentata* SDM and response curves (GAM, Random Forest, Maxent).......................... 108
28. *Lepidium lasiocarpum* SDM and response curves (GAM, Random Forest, Maxent) 112
29. *Lupinus odoratus* SDM and response curves (Random Forest) 116
30. *Lycium andersonii* SDM and response curves (GAM, Random Forest, Maxent) 118
31. *Lycium cooperi* SDM and response curves (GAM, Random Forest, Maxent) 122
32. *Lycium pallidum* SDM and response curves (GAM, Random Forest, Maxent) 126

33. *Malacothrix glabrata* SDM and response curves (GAM, Random Forest, Maxent) 130
34. *Mirabilis laevis* SDM and response curves (GAM, Random Forest, Maxent) 134
35. *Muhlenbergia porteri* SDM and response curves (GAM, Random Forest, Maxent) 138
36. *Oenothera cespitosa* SDM and response curves (GAM, Random Forest, Maxent) 142
37. *Oenothera deltoides* SDM and response curves (GAM, Random Forest, Maxent) 146
38. *Oenothera primiveris* SDM and response curves (GAM, Random Forest, Maxent) 150
39. *Opuntia basilaris* SDM and response curves (GAM, Random Forest, Maxent) 154
40. *Penstemon palmeri* SDM and response curves (GAM, Random Forest, Maxent) 158
41. *Plantago ovata* SDM and response curves (GAM, Random Forest, Maxent) 162
42. *Psorothamnus fremontii* SDM and response curves (GAM, Random Forest, Maxent) 166
43. *Salvia columbariae* SDM and response curves (GAM, Random Forest, Maxent) 170
44. *Sphaeralcea ambigua* SDM and response curves (GAM, Random Forest, Maxent) 174
45. *Stephanomeria exigua* SDM and response curves (GAM, Random Forest, Maxent) 178
46. *Stephanomeria parryi* SDM and response curves (GAM, Random Forest, Maxent) 182
47. *Vulpia octoflora* SDM and response curves (GAM, Random Forest, Maxent) 186
48. *Yucca brevifolia* SDM and response curves (GAM, Random Forest, Maxent) 190
49. *Yucca schidigera* SDM and response curves (GAM, Random Forest, Maxent) 194
Disclaimer 198

1. *Acmispon humistratus* SDM and response curves (GAM, Random Forest, Maxent)

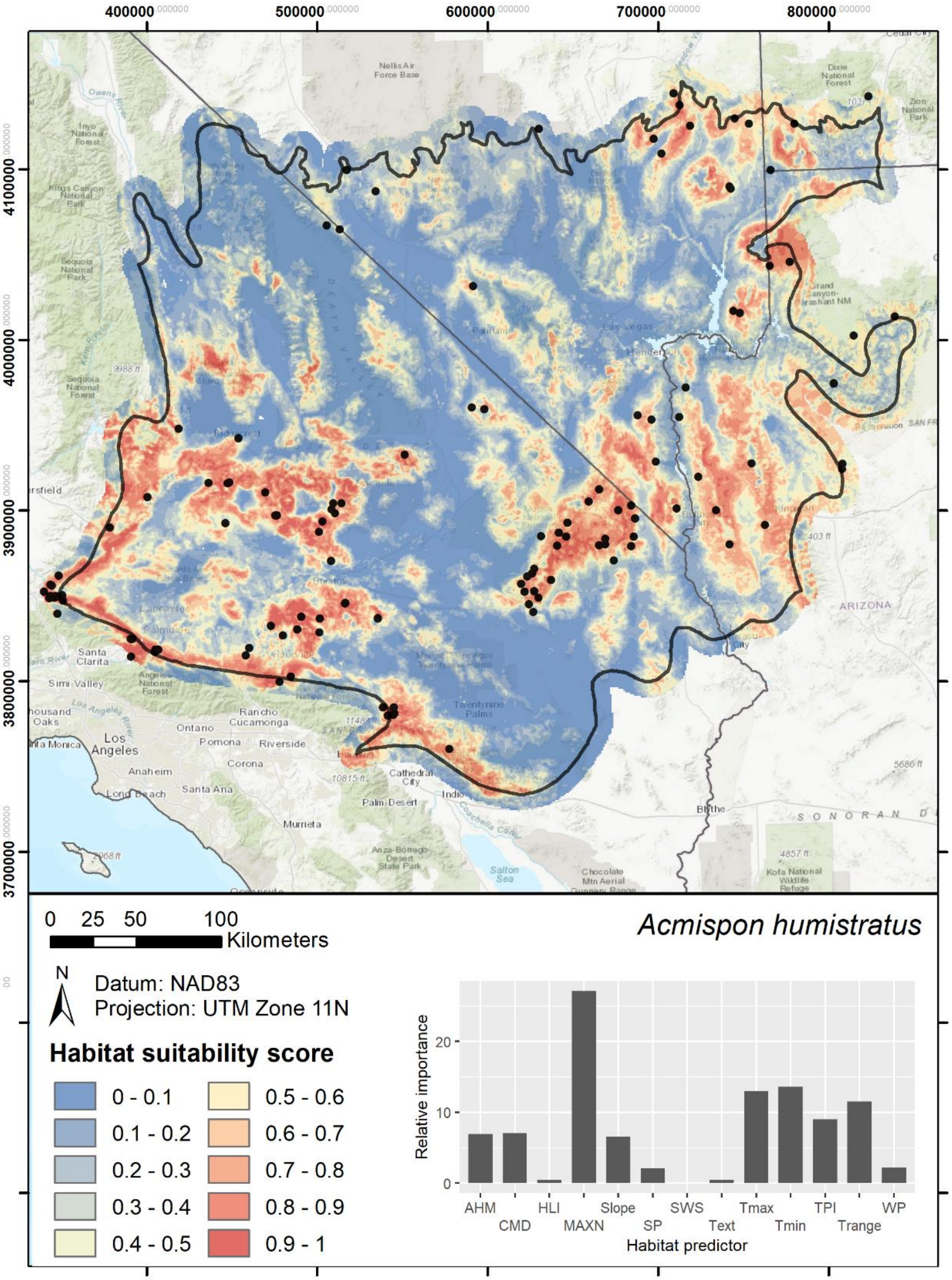

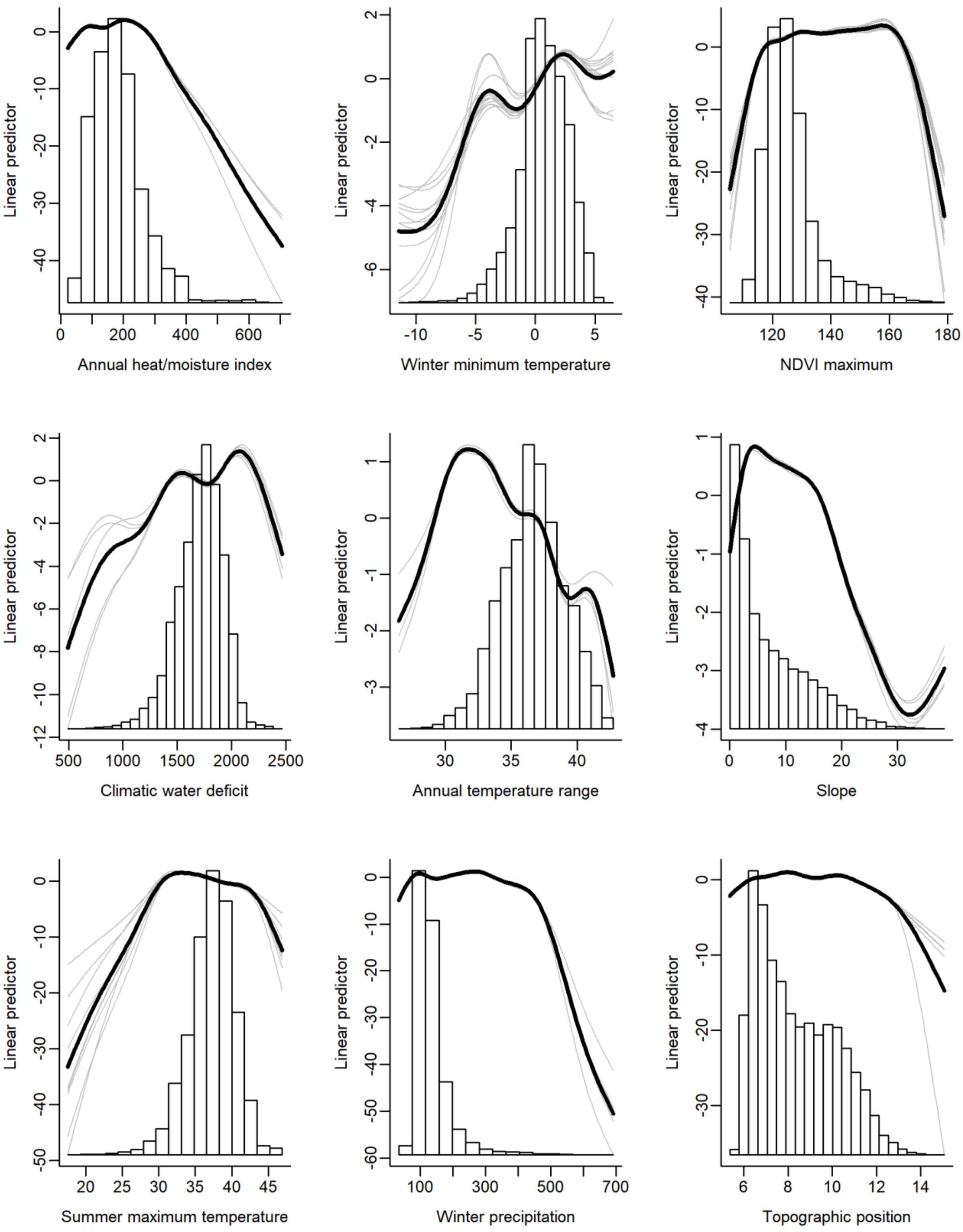

GAM response curves for *Acmispon humistratus*

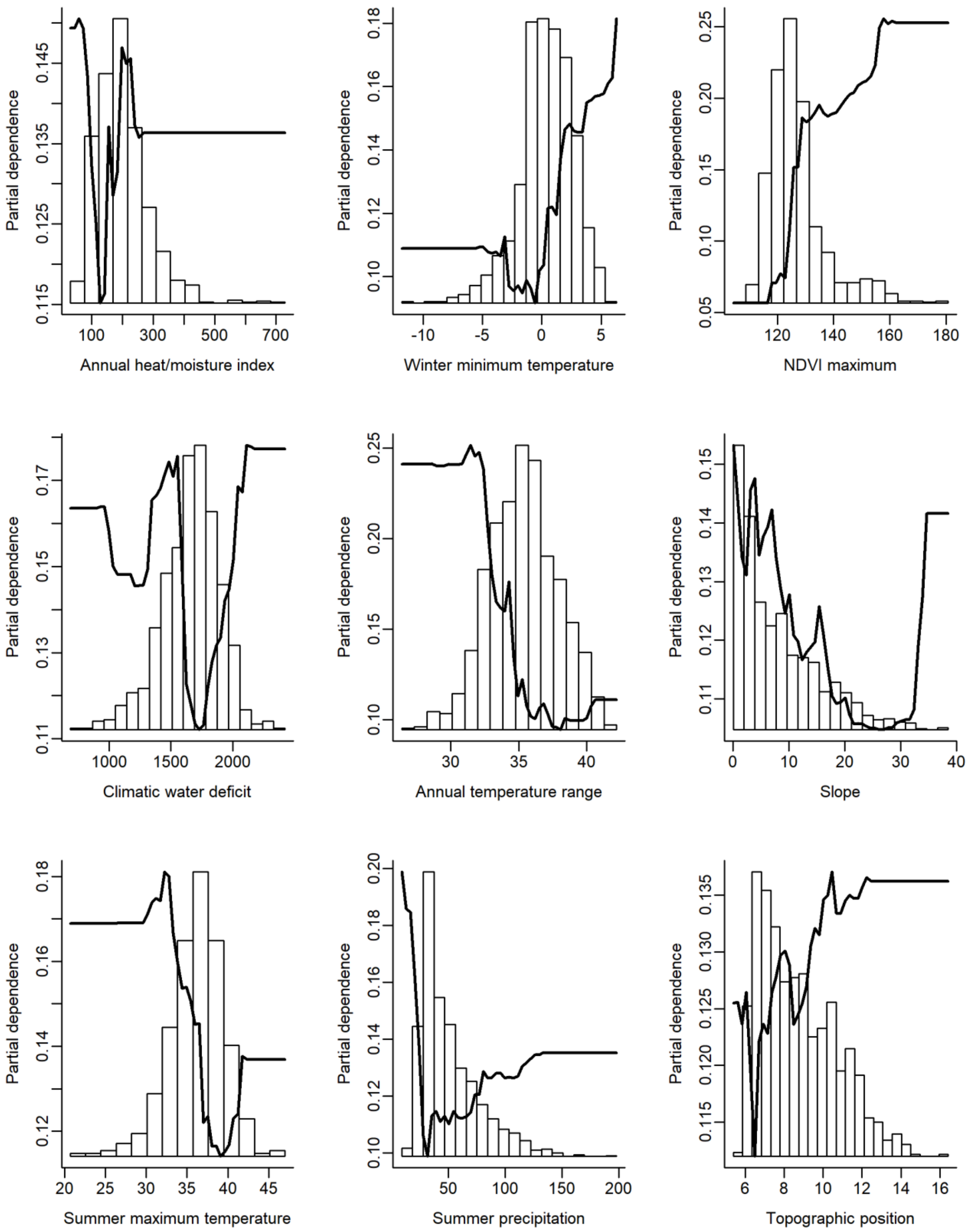

Random Forest response curves for *Acmispon humistratus*

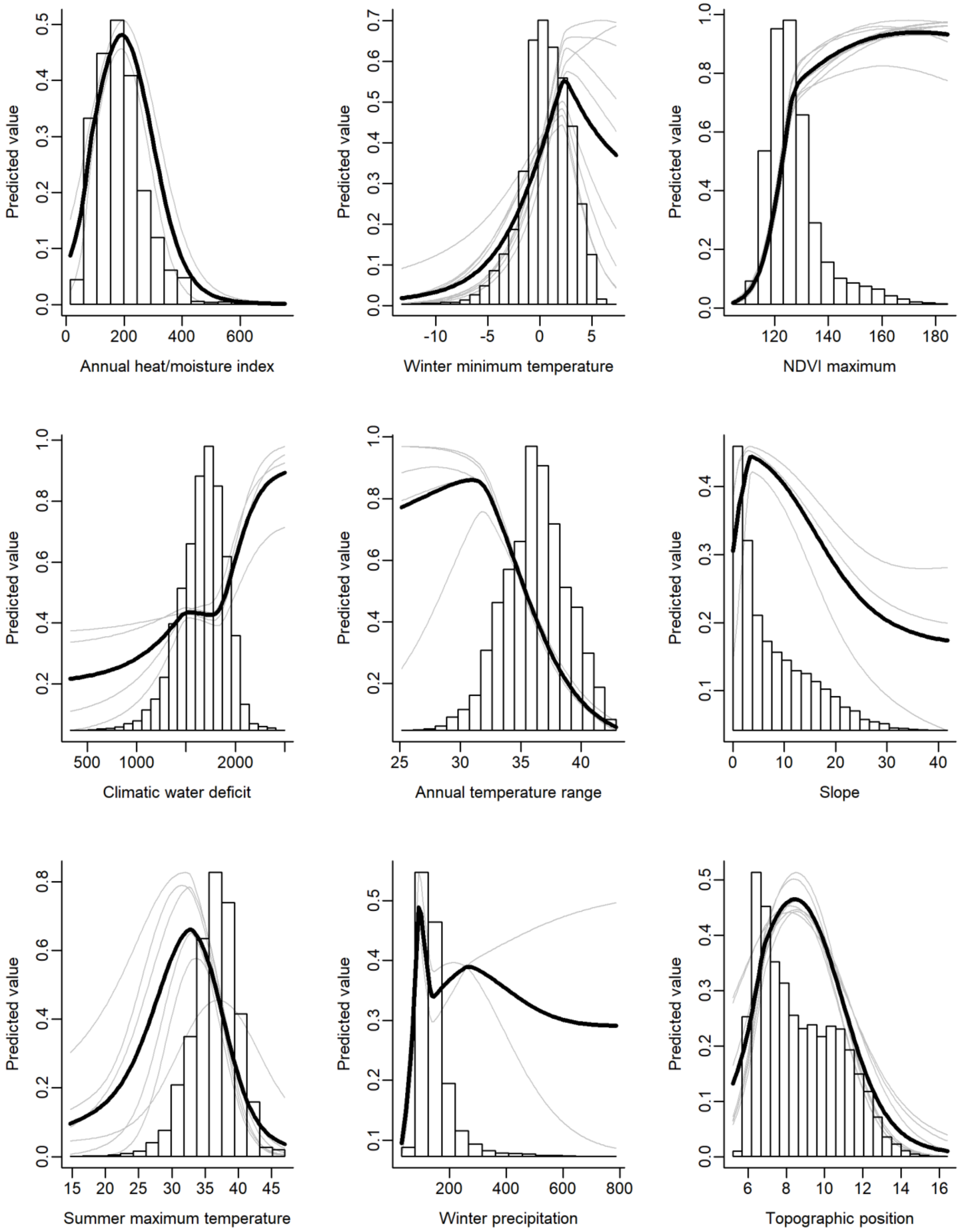

MaxEnt response curves for *Acmispon humistratus*

2. *Achnatherum hymenoides* SDM and response curves (GAM, Random Forest, Maxent)

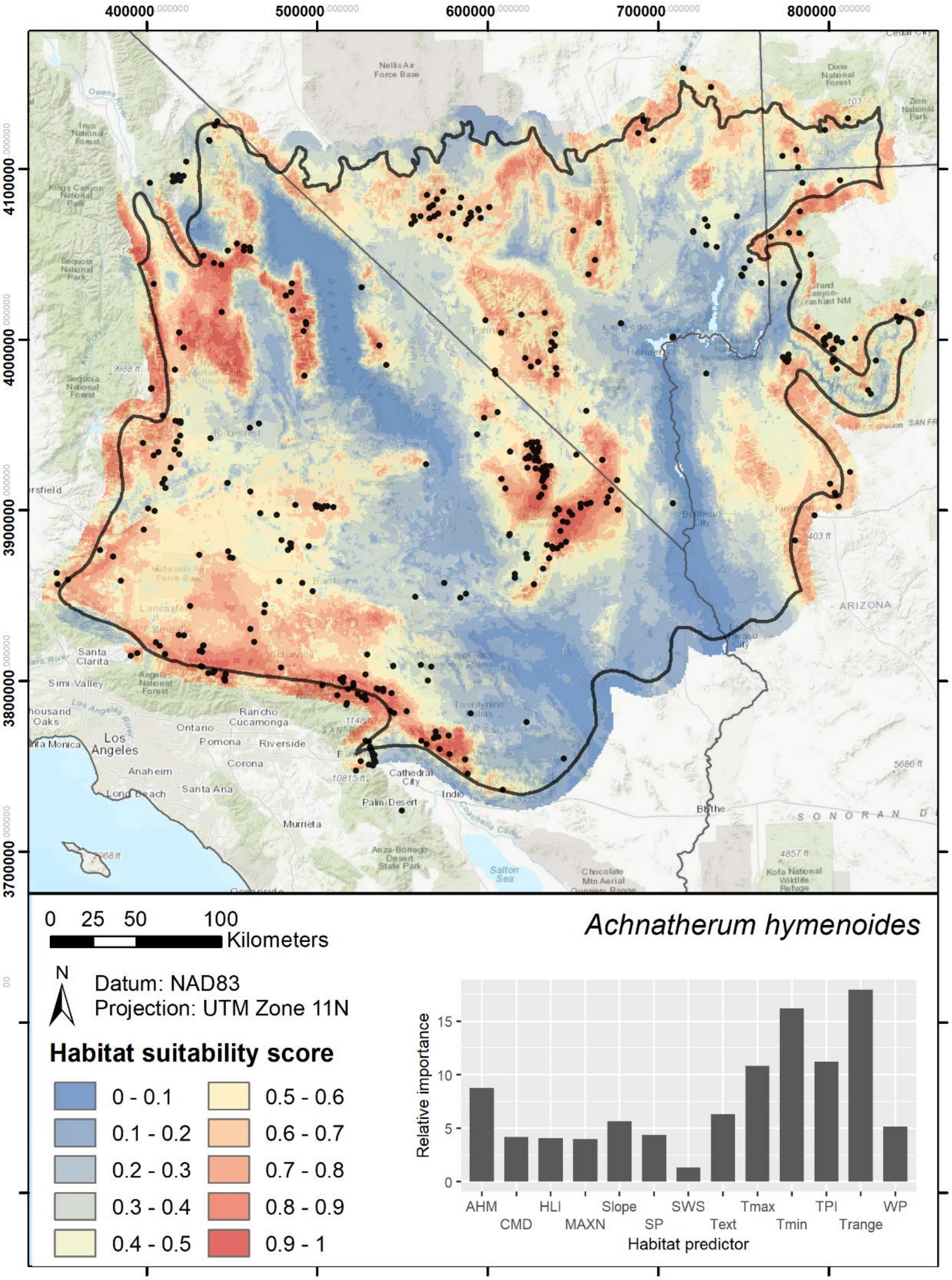

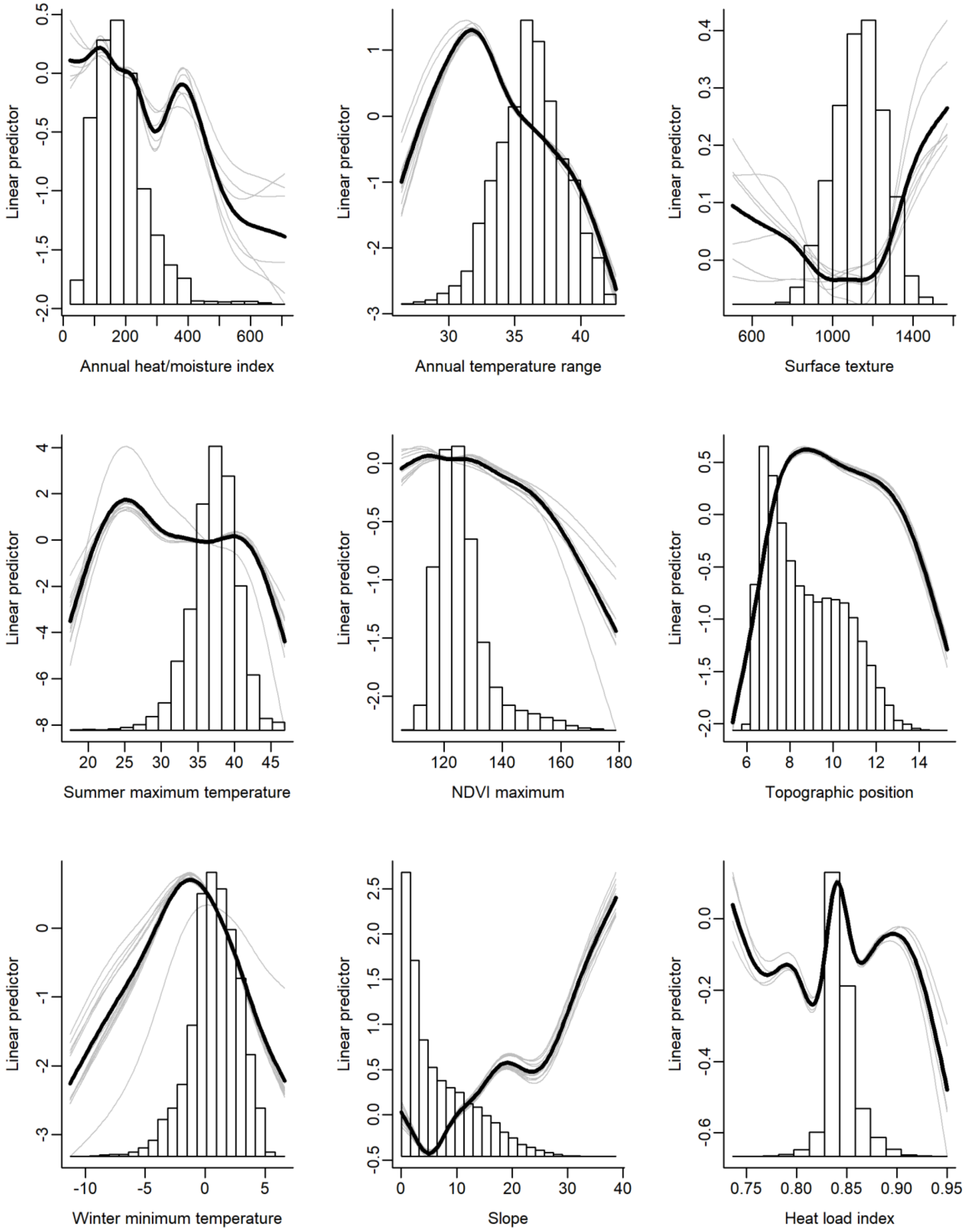

GAM response curves for *Achnatherum hymenoides*

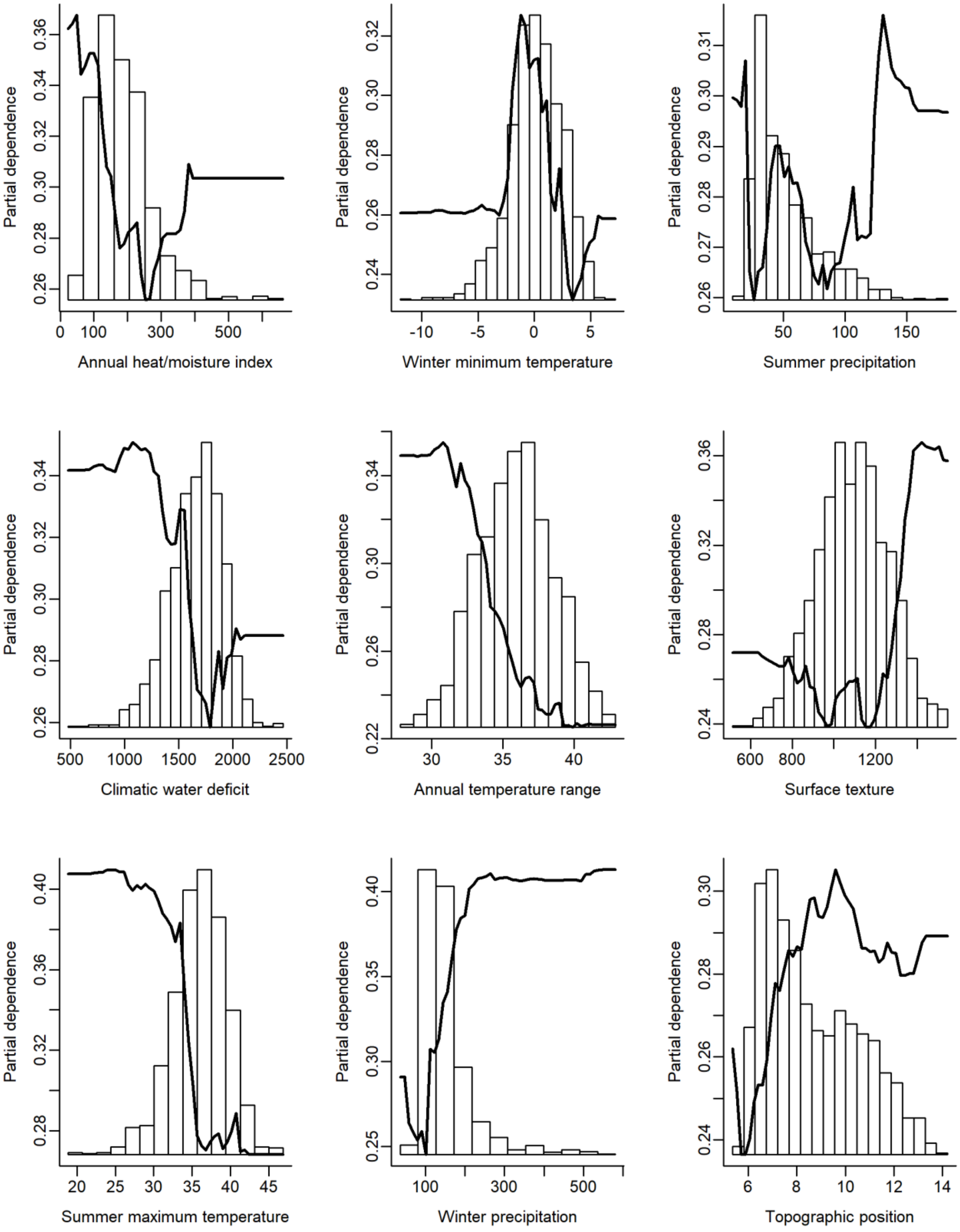

Random Forest response curves for *Achnatherum hymenoides*

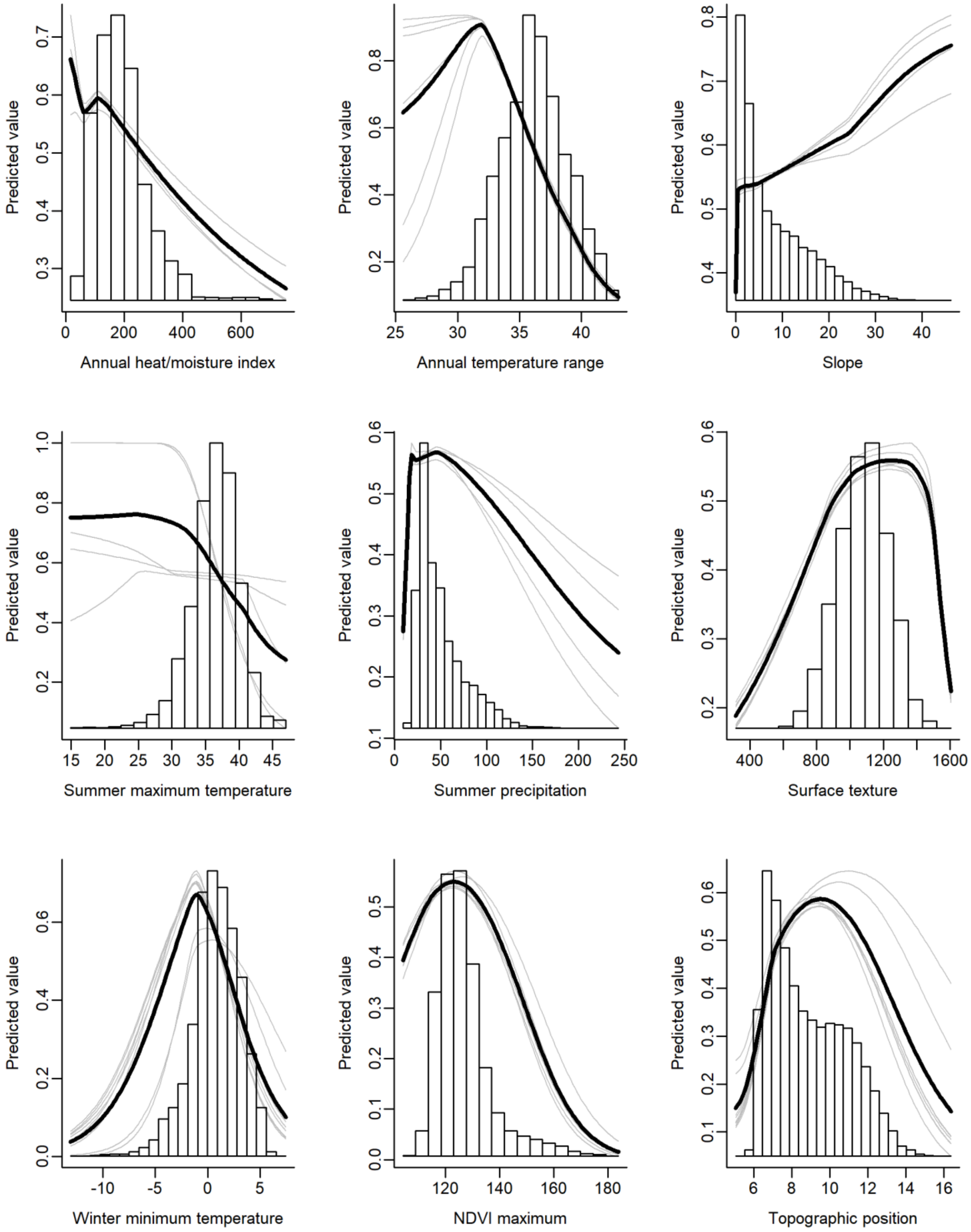

MaxEnt response curves for *Achnatherum hymenoides*

3. *Acmispon strigosus* SDM and response curves (GAM, Random Forest, Maxent)

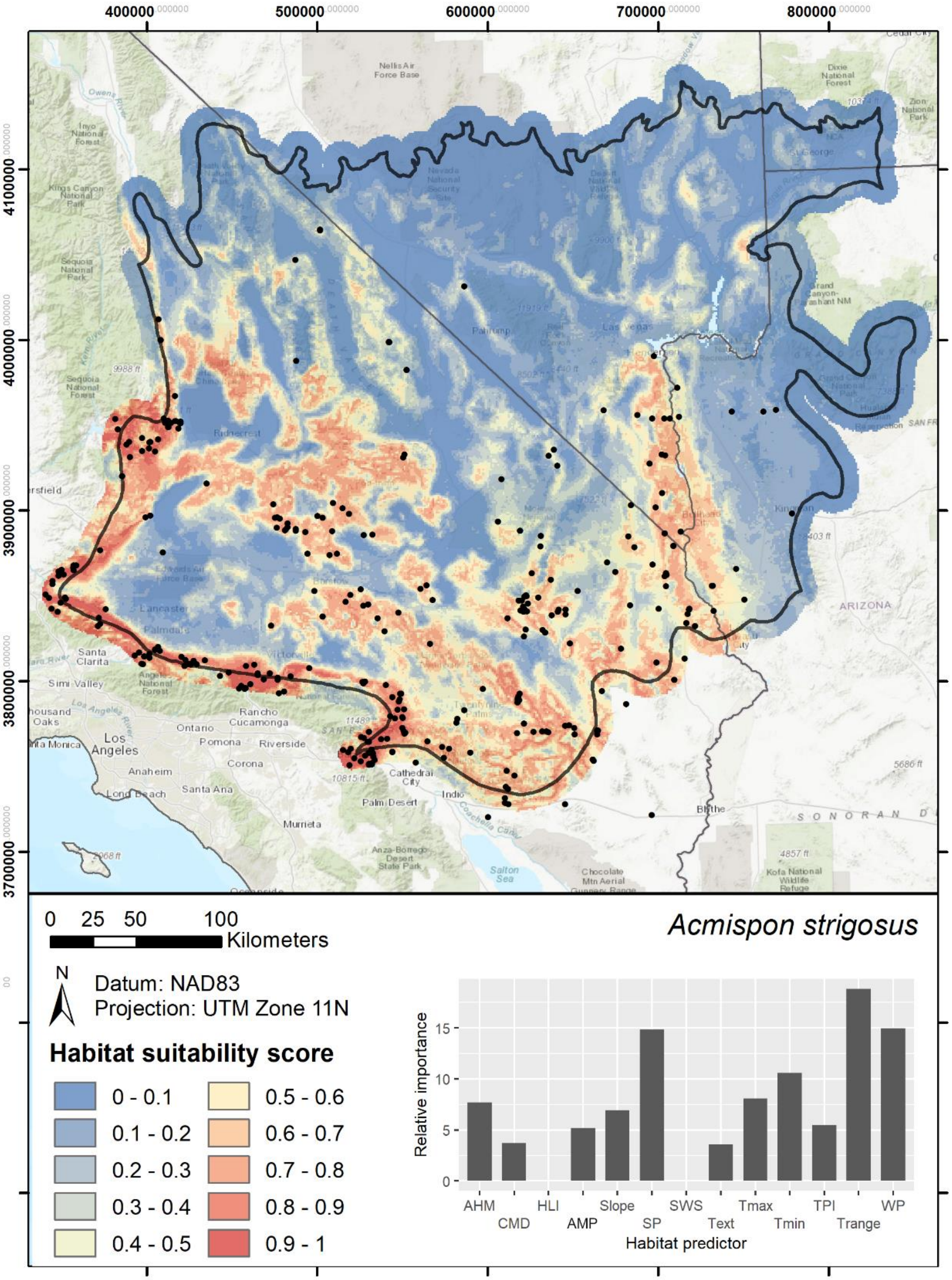

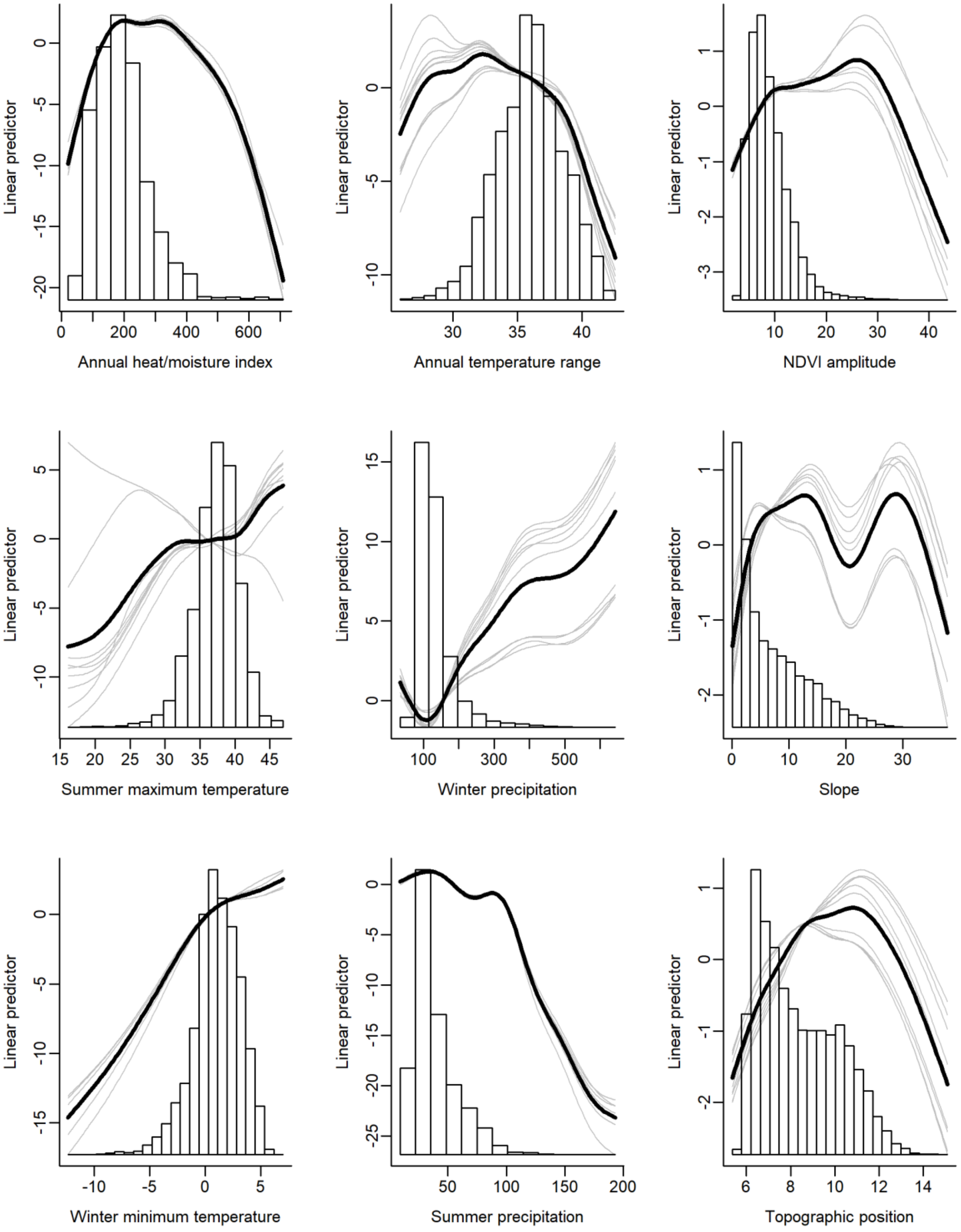

GAM response curves for *Acmispon strigosus*

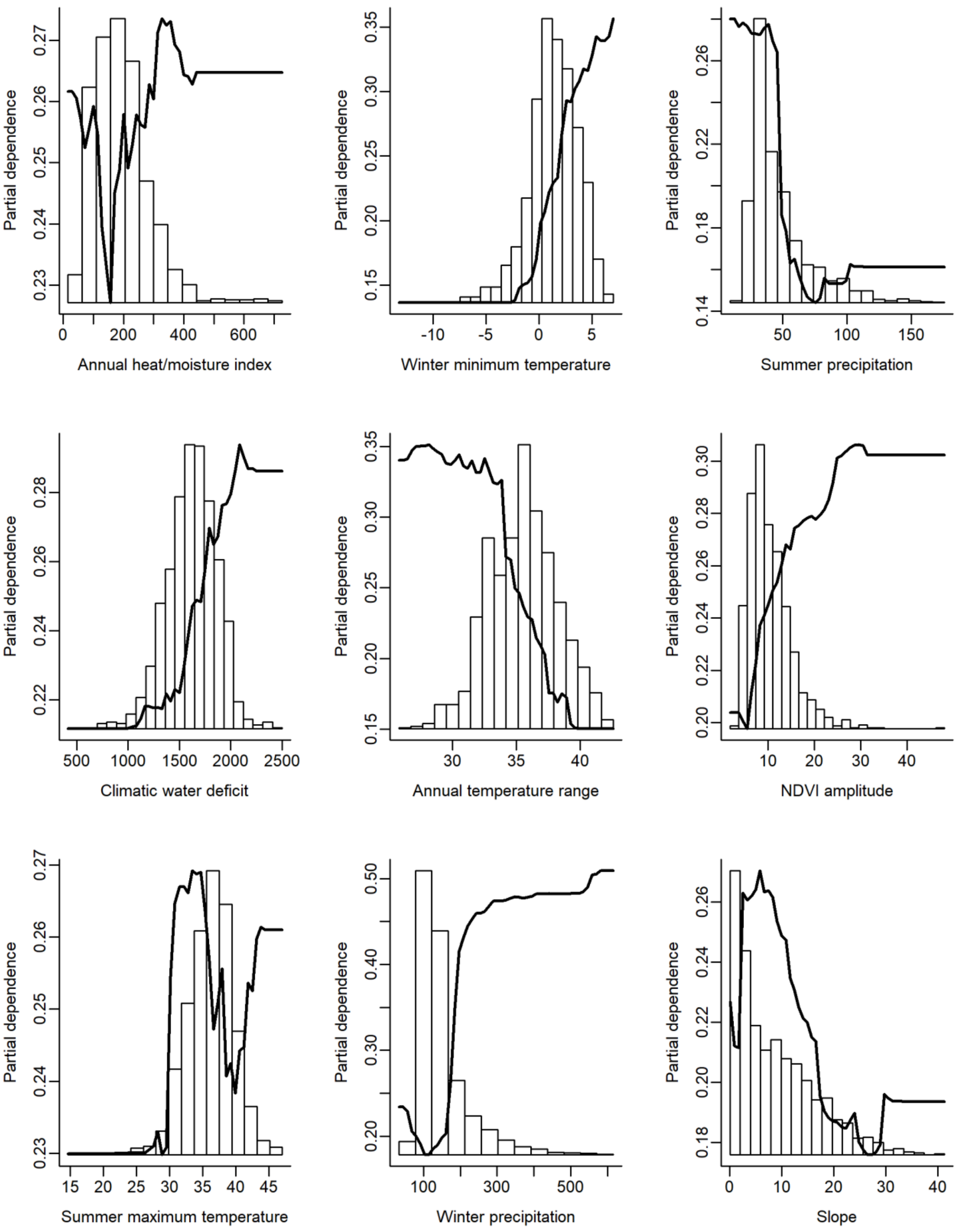

Random Forest response curves for *Acmispon strigosus*

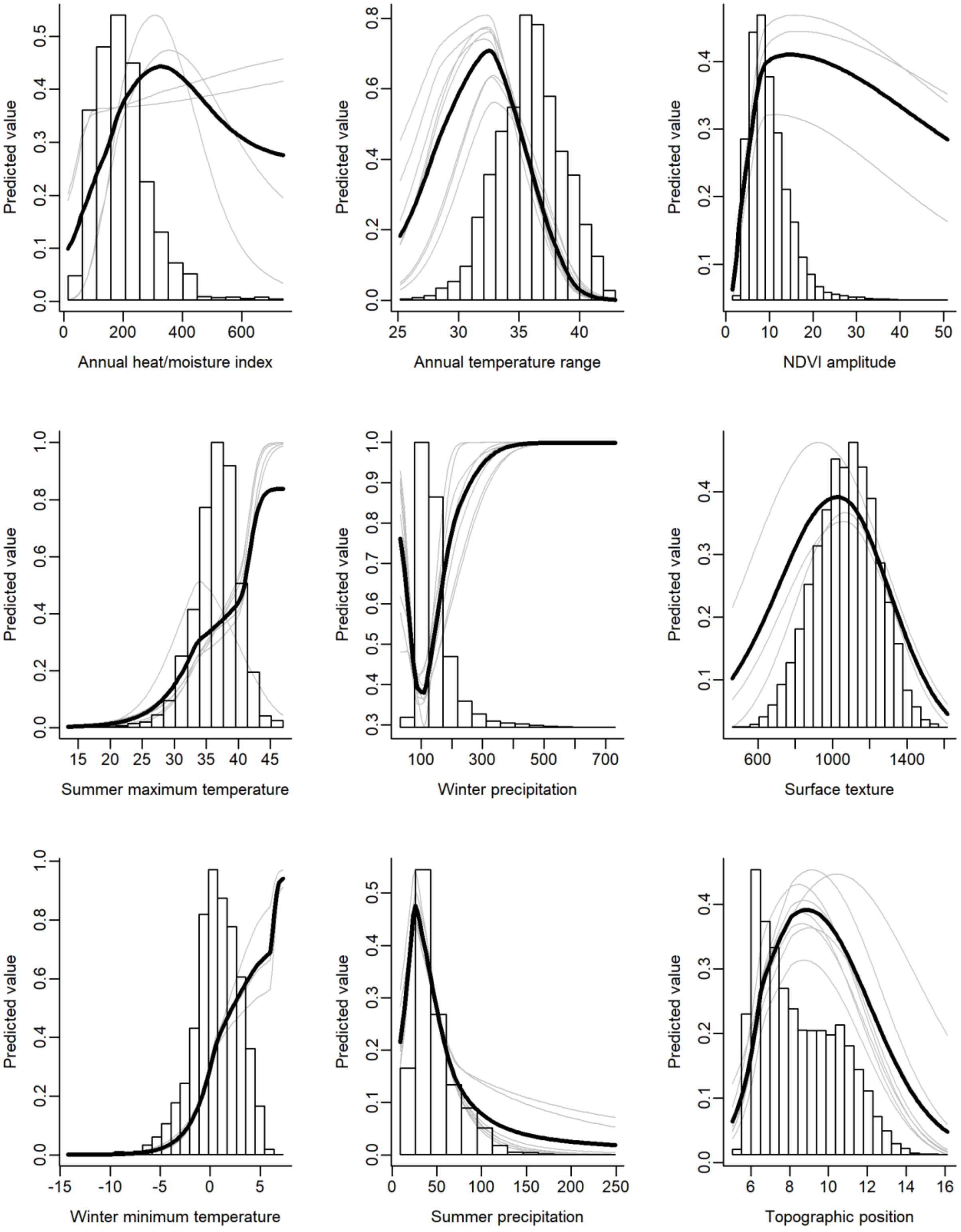

MaxEnt response curves for *Acmispon strigosus*

4. *Ambrosia dumosa* SDM and response curves (GAM, Random Forest, Maxent)

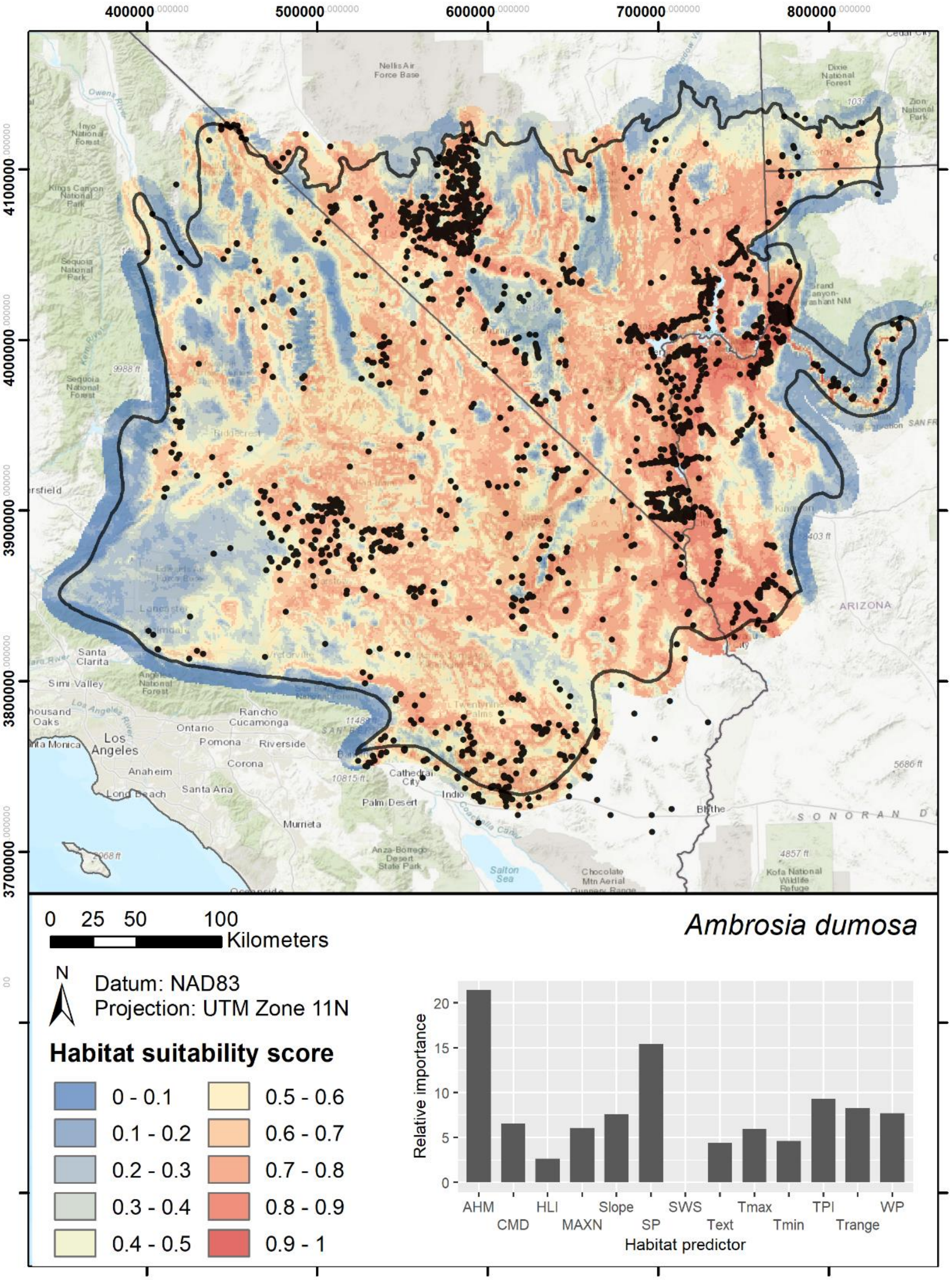

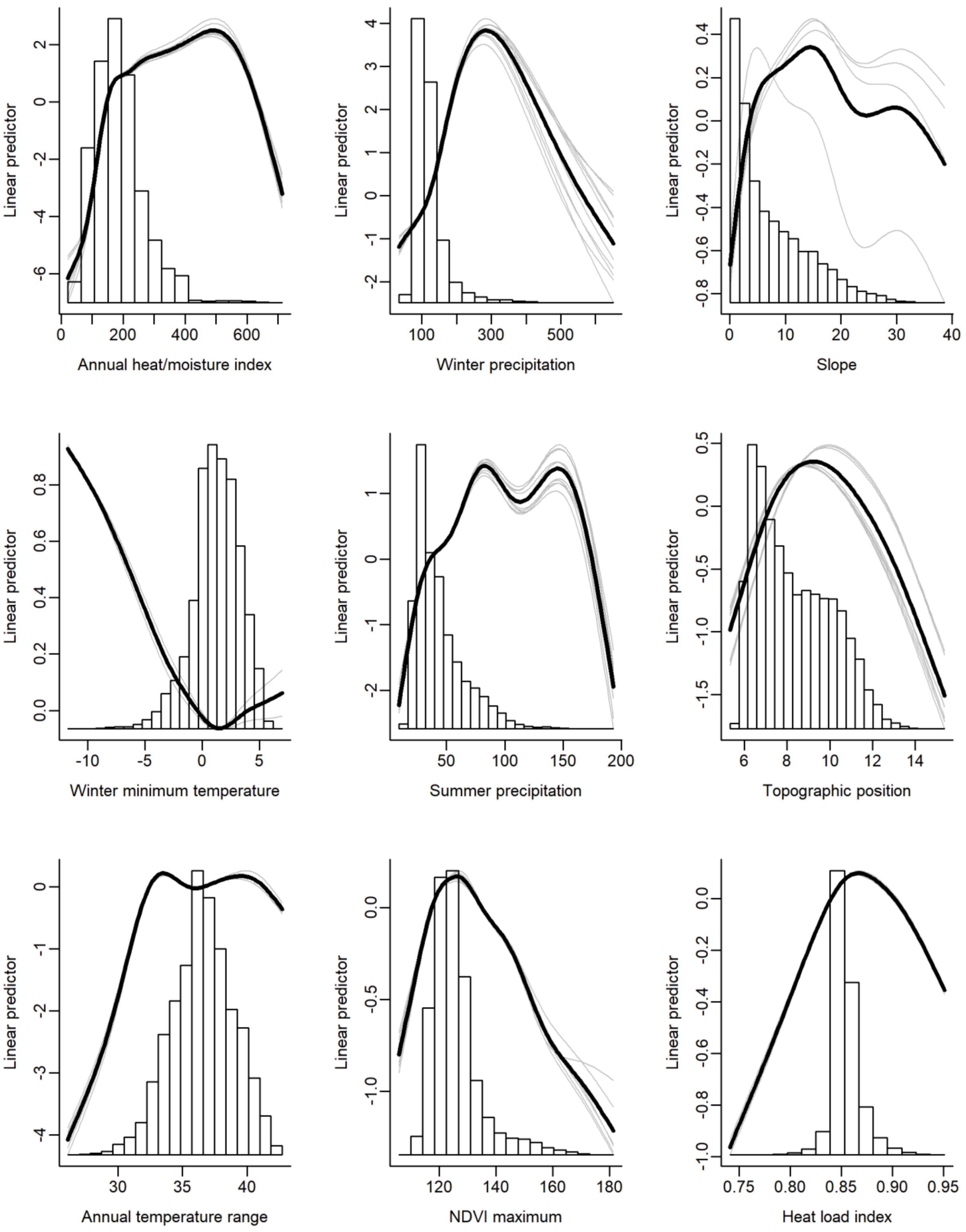

GAM response curves for *Ambrosia dumosa*

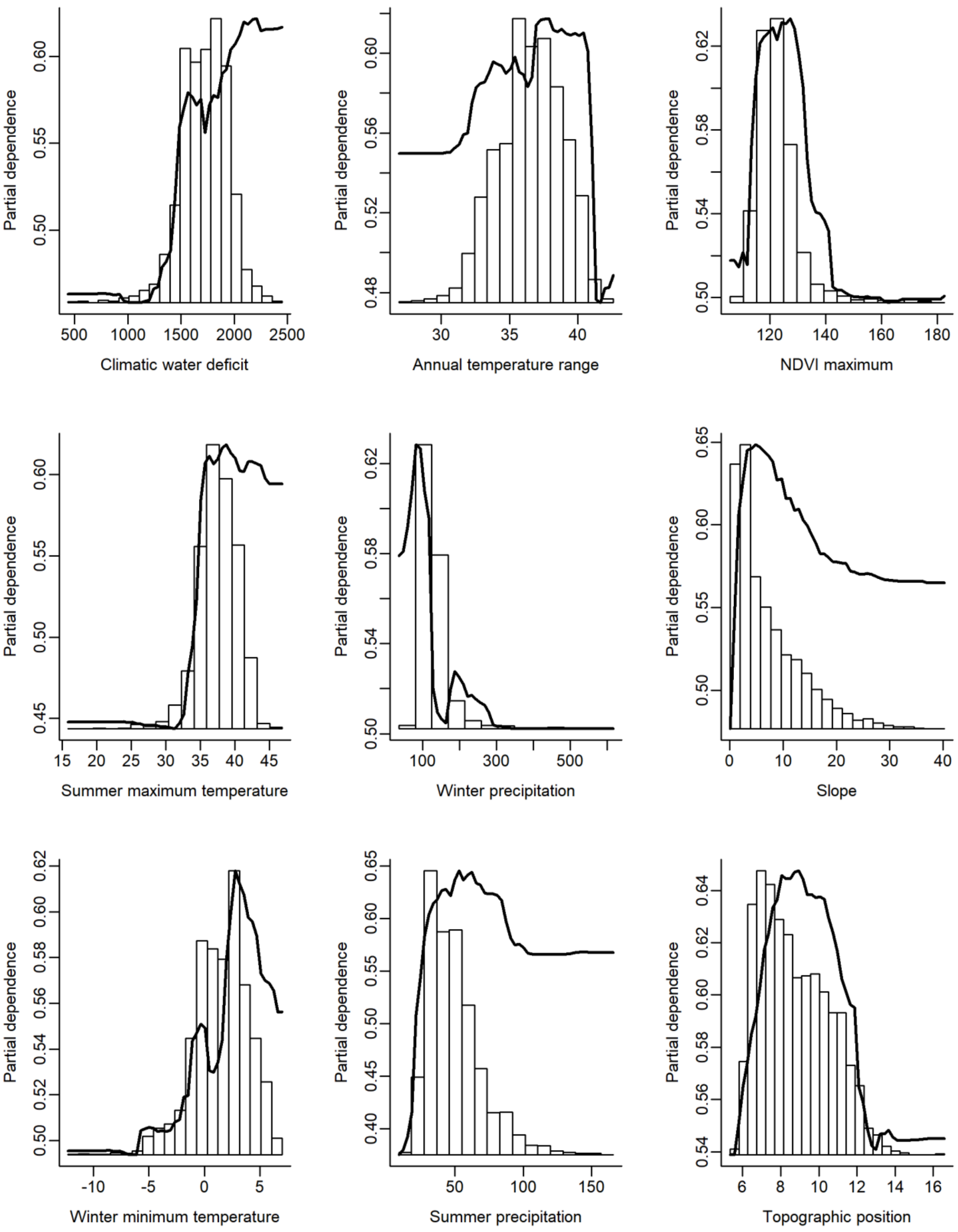

Random Forest response curves for *Ambrosia dumosa*

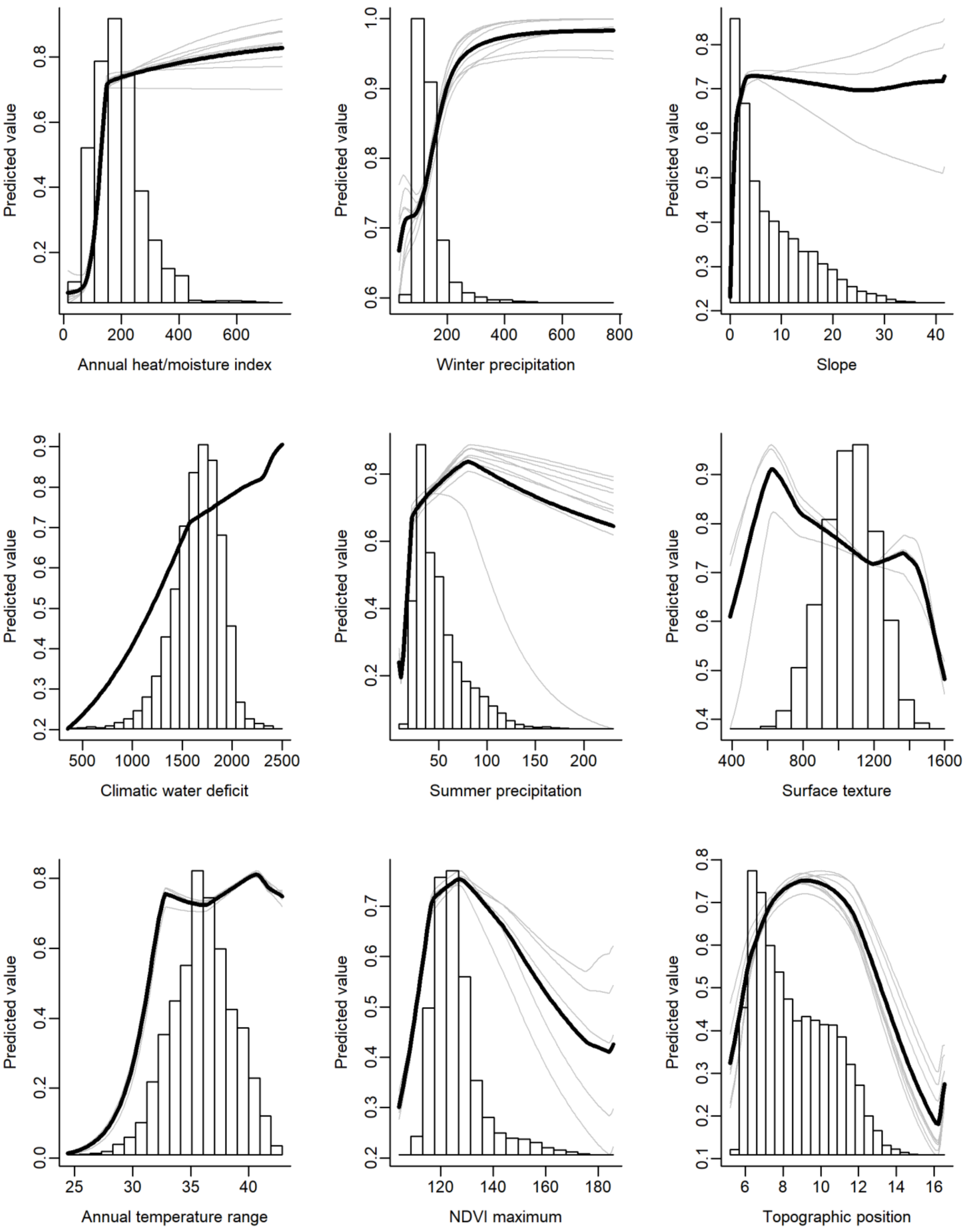

MaxEnt response curves for *Ambrosia Dumosa*

5. *Ambrosia salsola* SDM and response curves (GAM, Random Forest, Maxent)

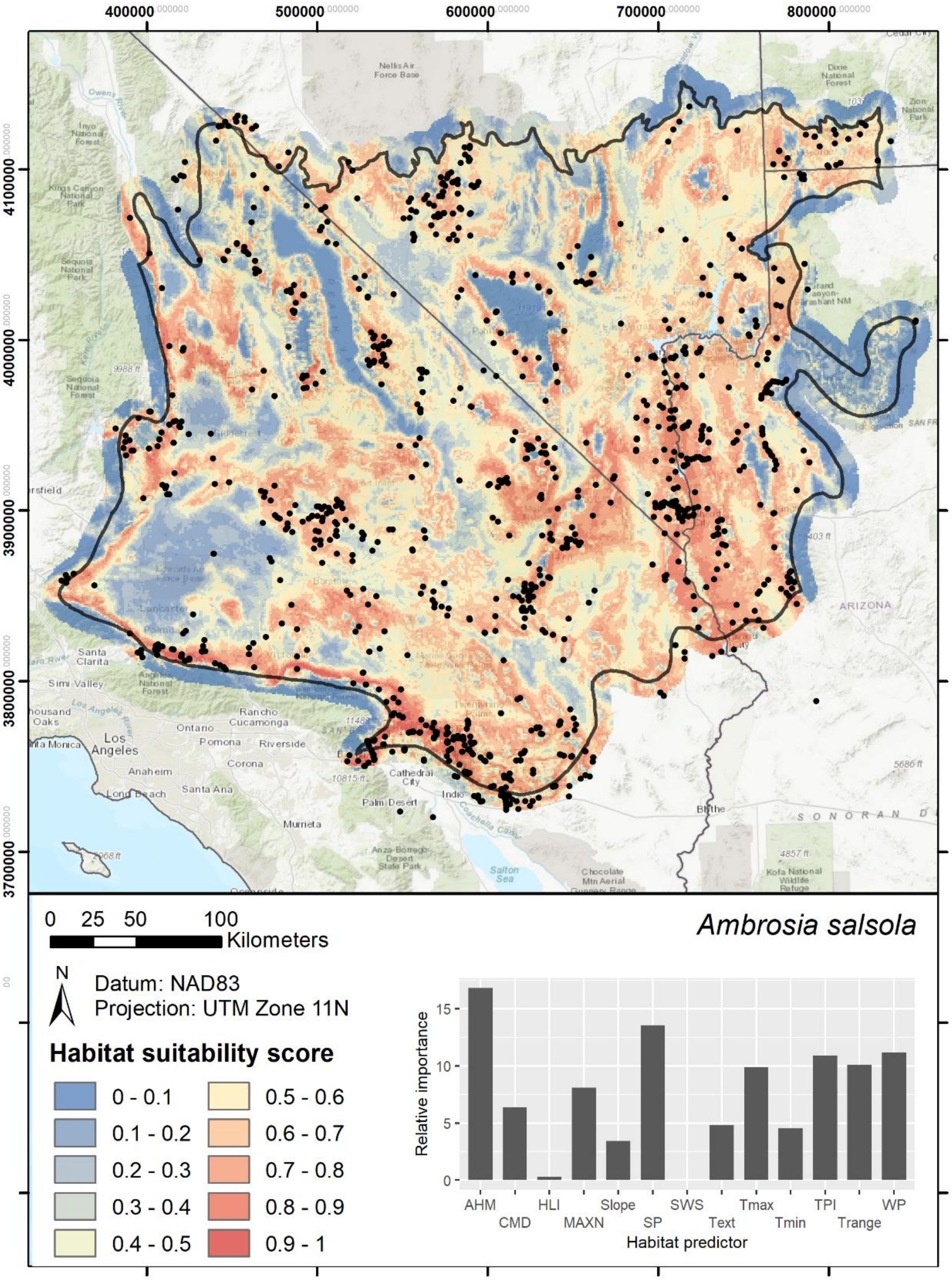

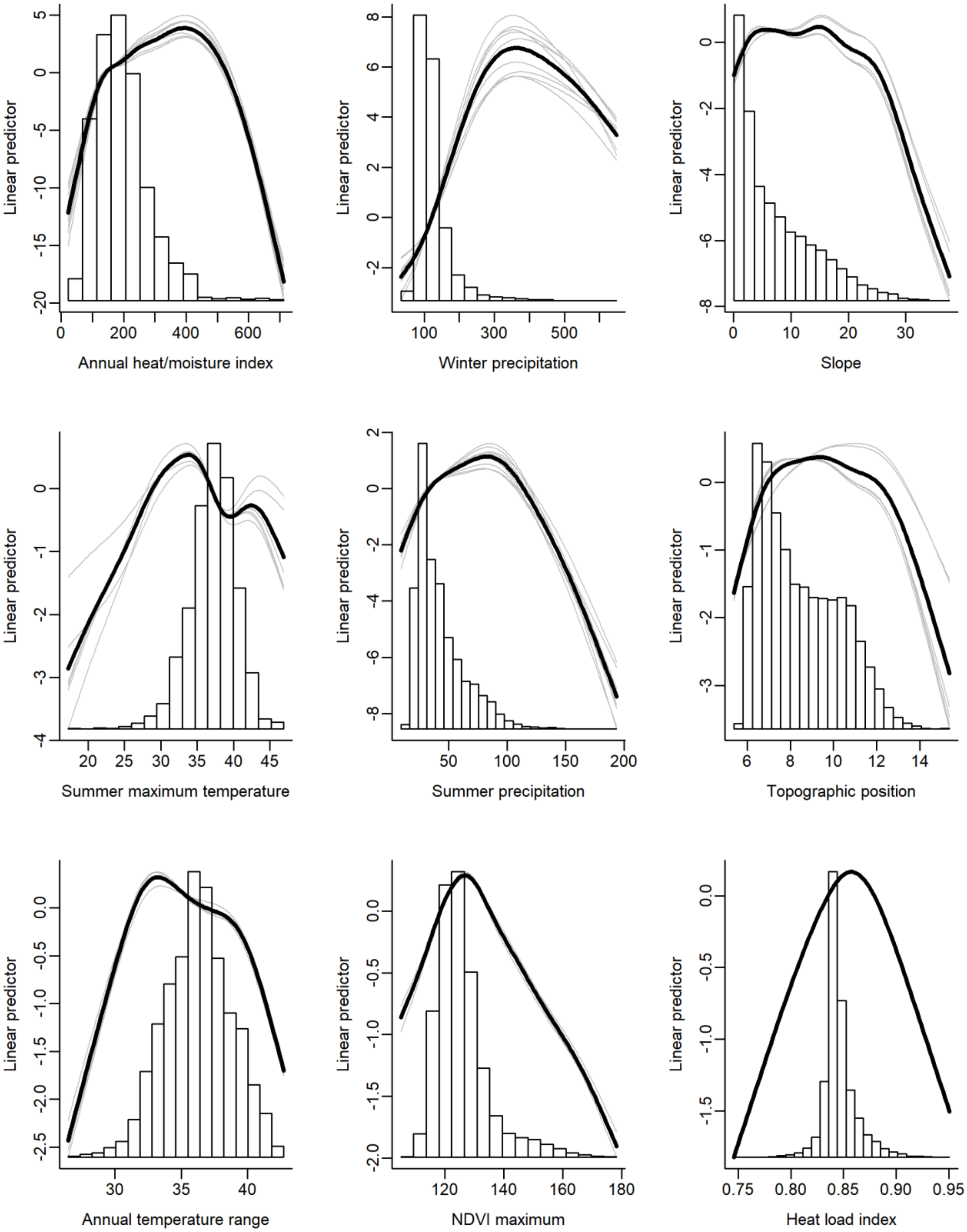

GAM response curves for *Ambrosia salsola*

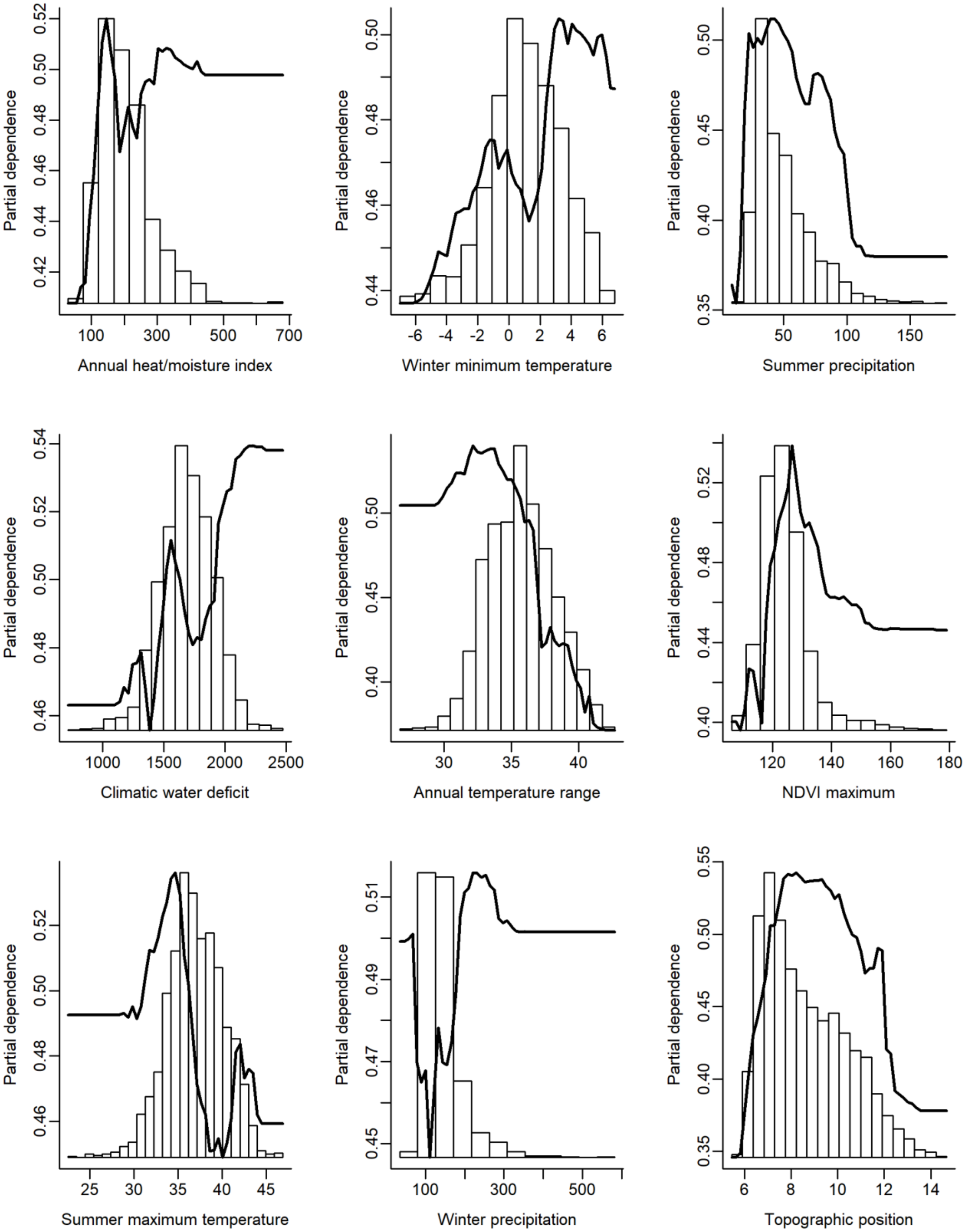

Random Forest response curves for *Ambrosia salsola*

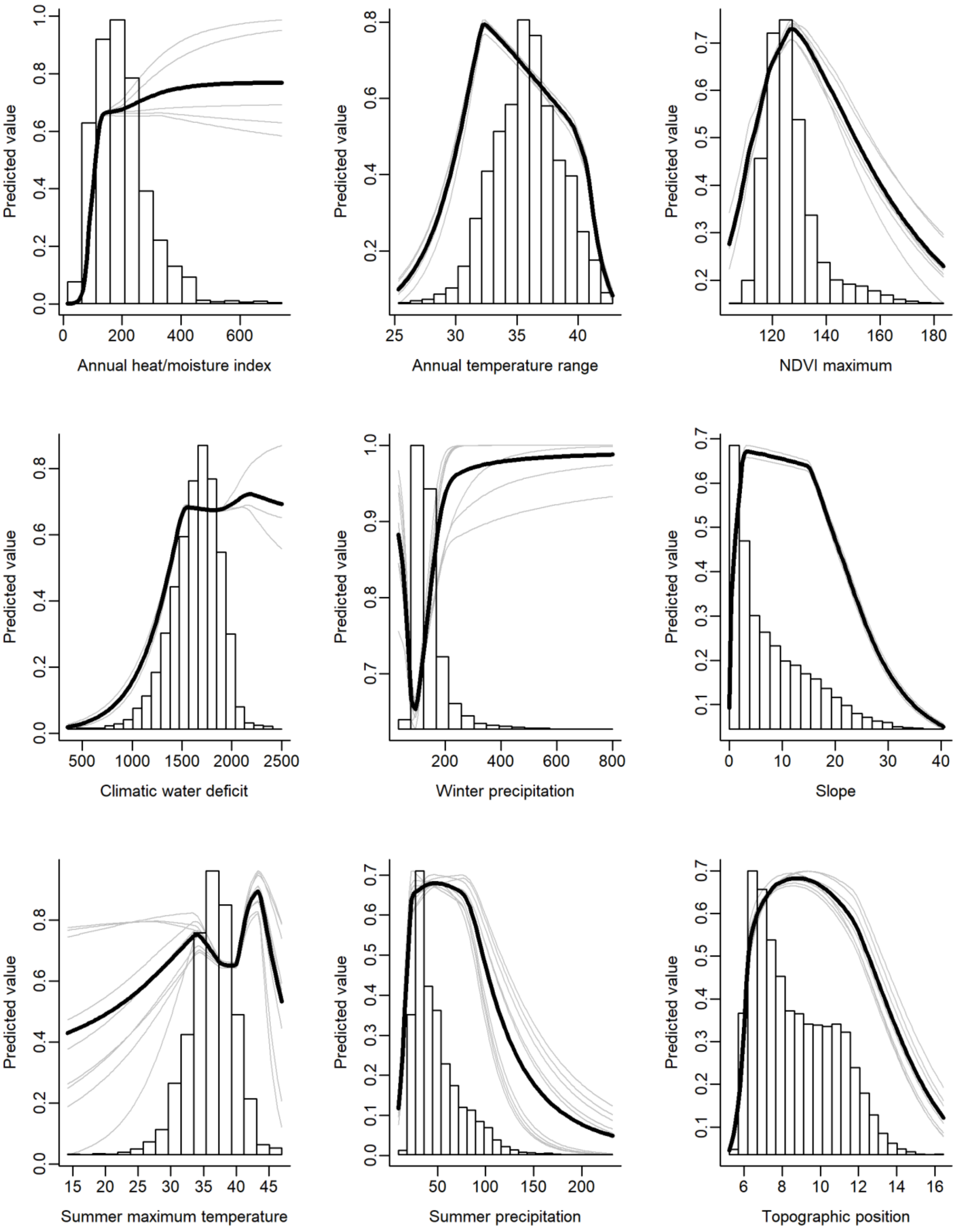

MaxEnt response curves for *Ambrosia salsola*

6. *Amsinckia tessellata* SDM and response curves (GAM, Random Forest, Maxent)

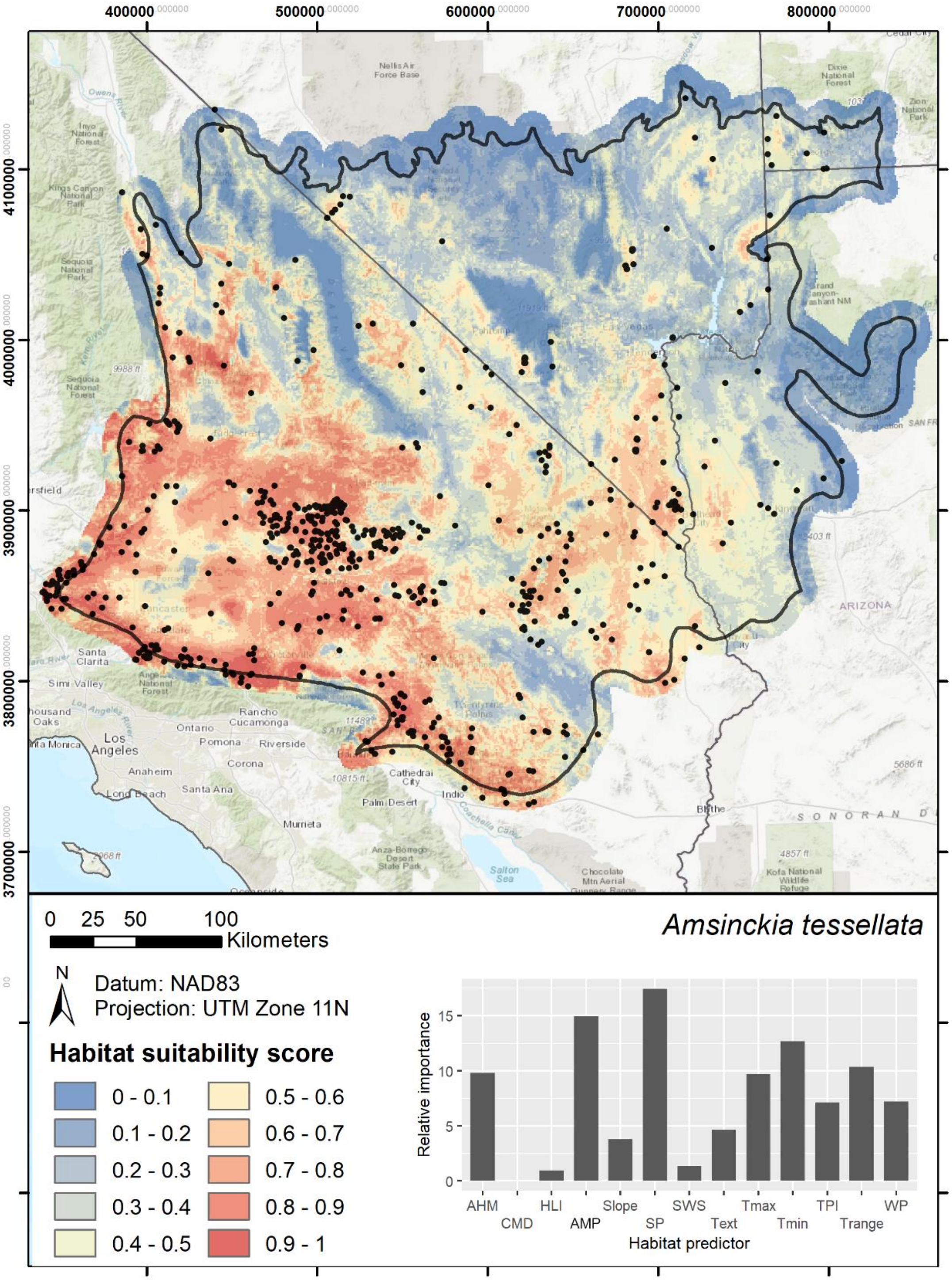

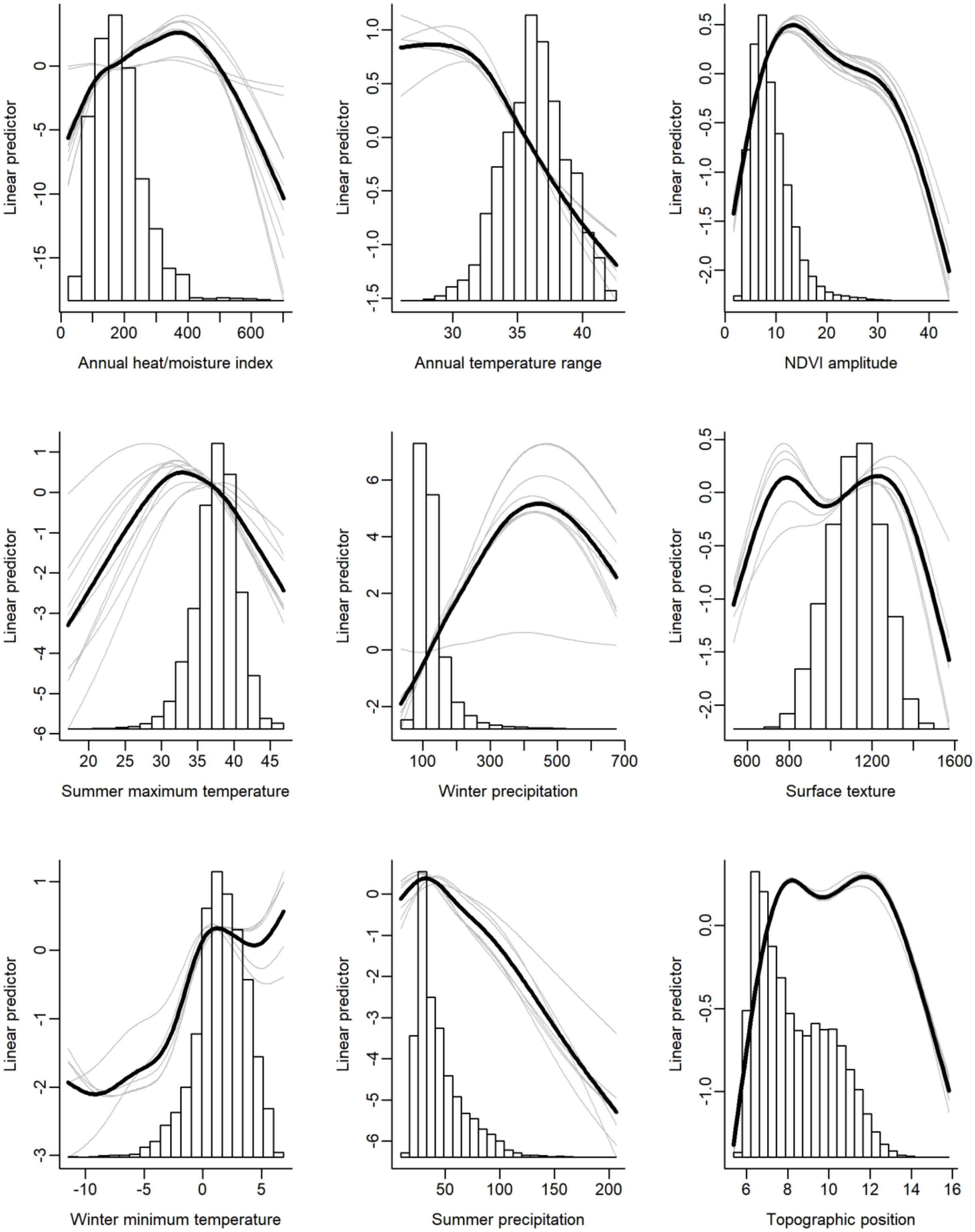

GAM response curves for *Amsinckia tessellata*

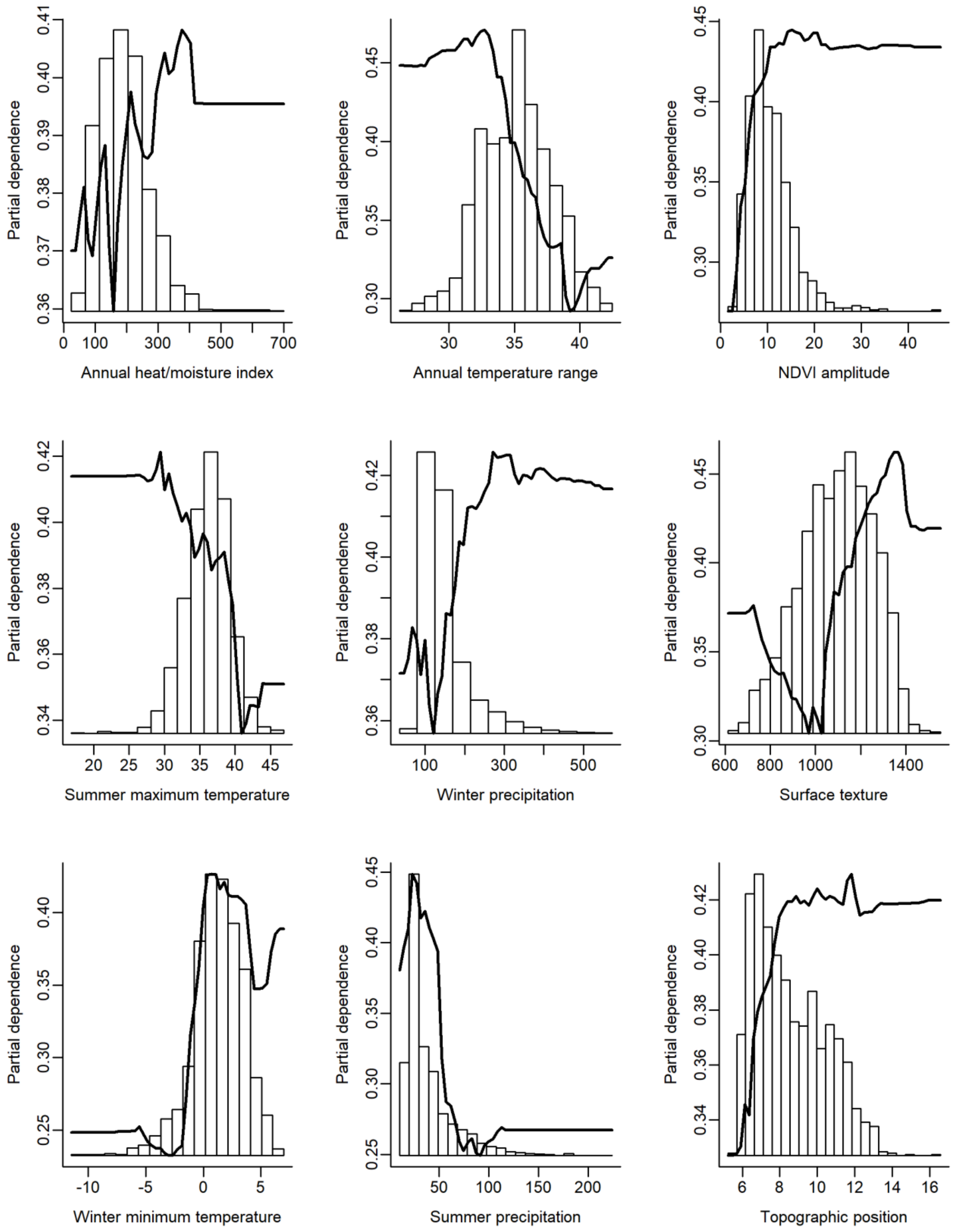

Random Forest response curves for *Amsinckia tessellata*

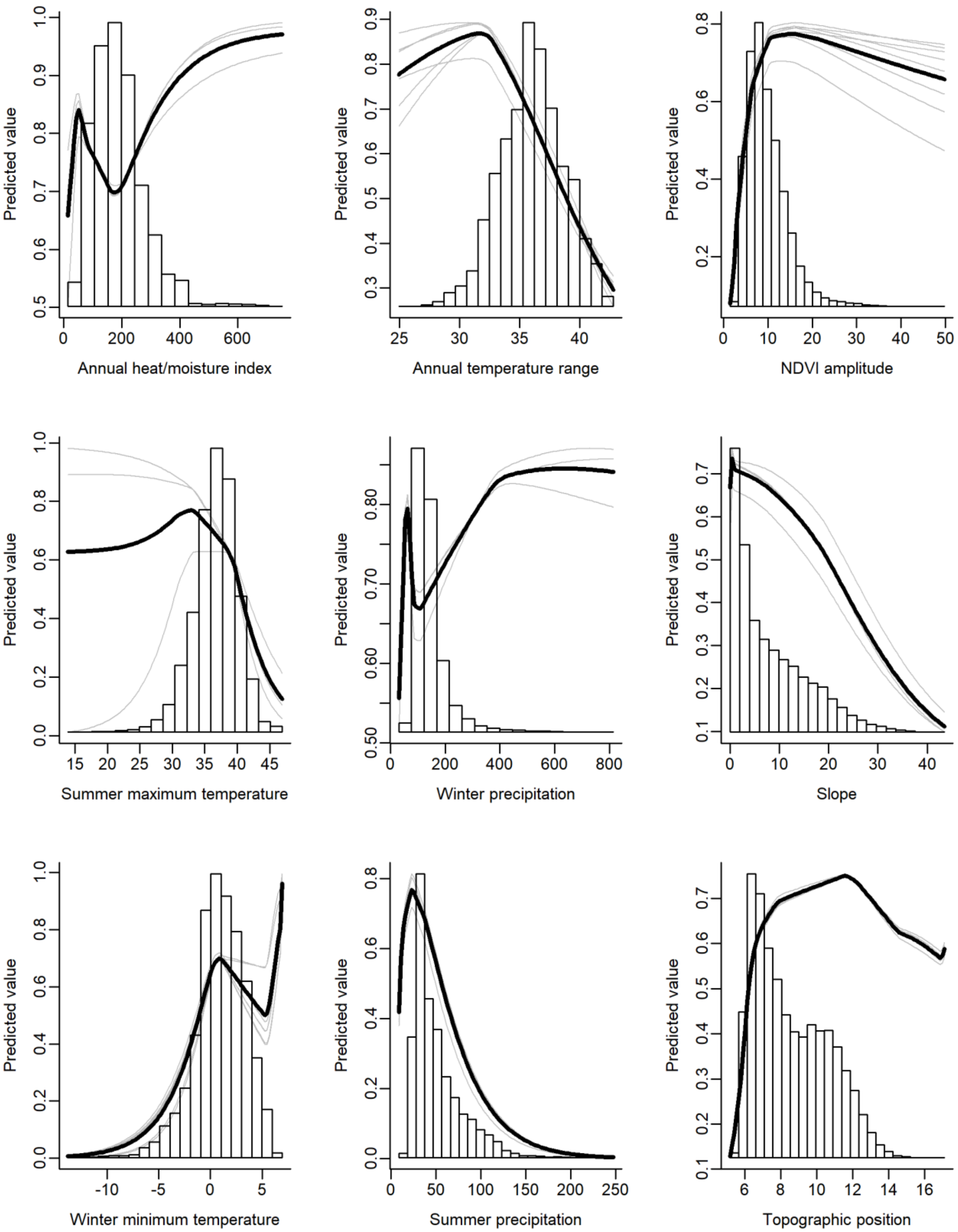

MaxEnt response curves for *Amsinckia tessellata*

7. *Astragalus didymocarpus* SDM and response curves (GAM, Random Forest, Maxent)

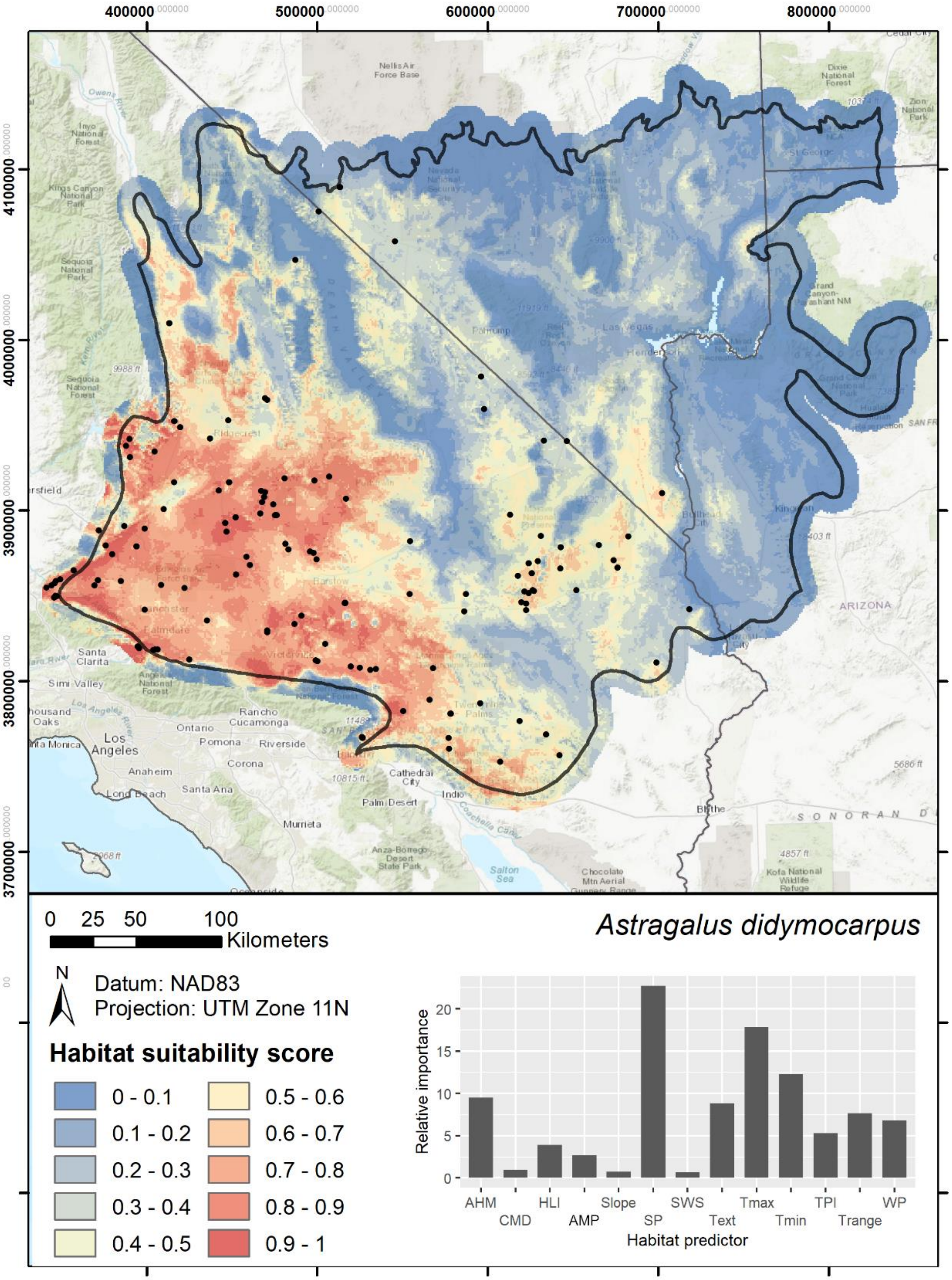

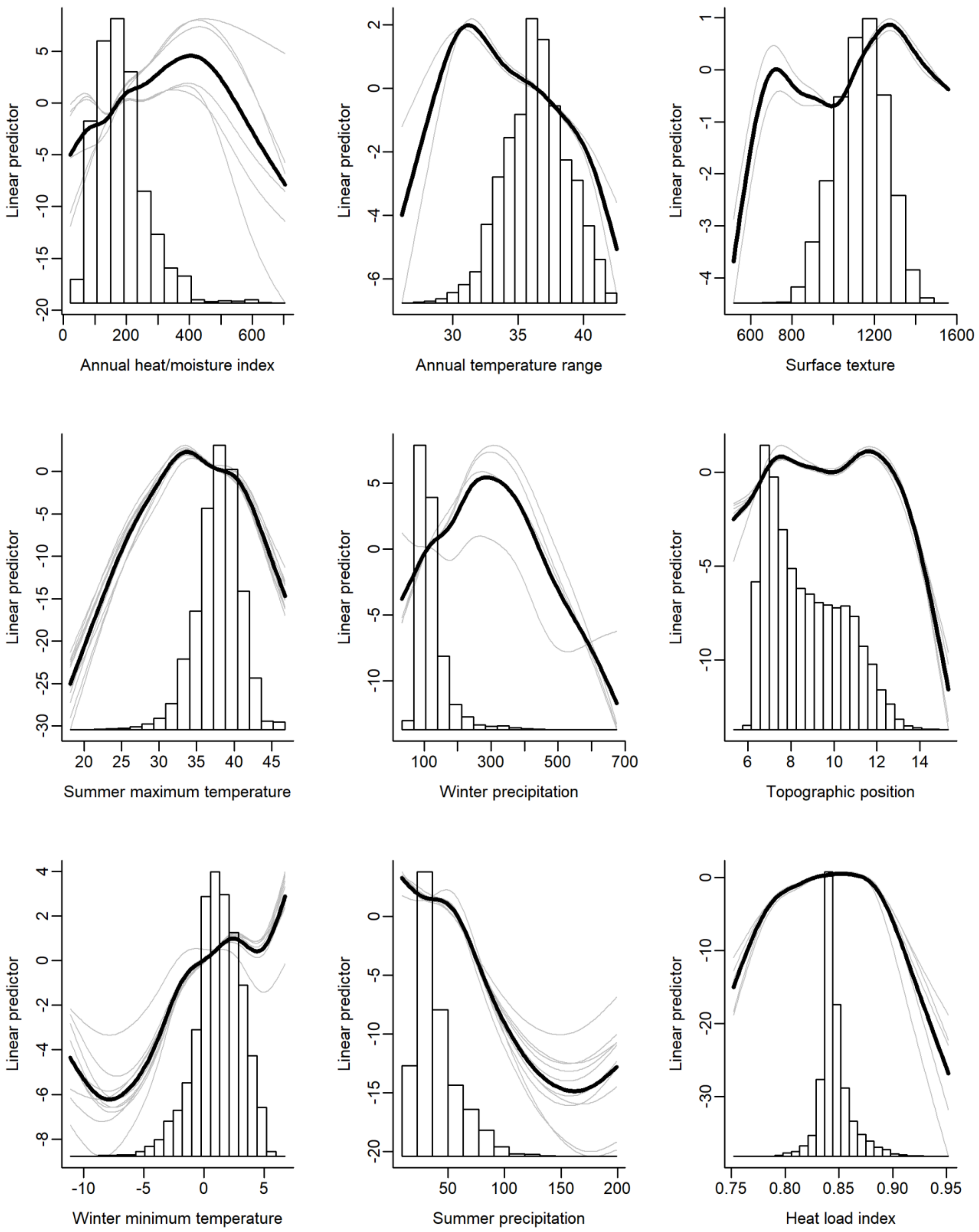

GAM response curves for *Astragalus didymocarpus*

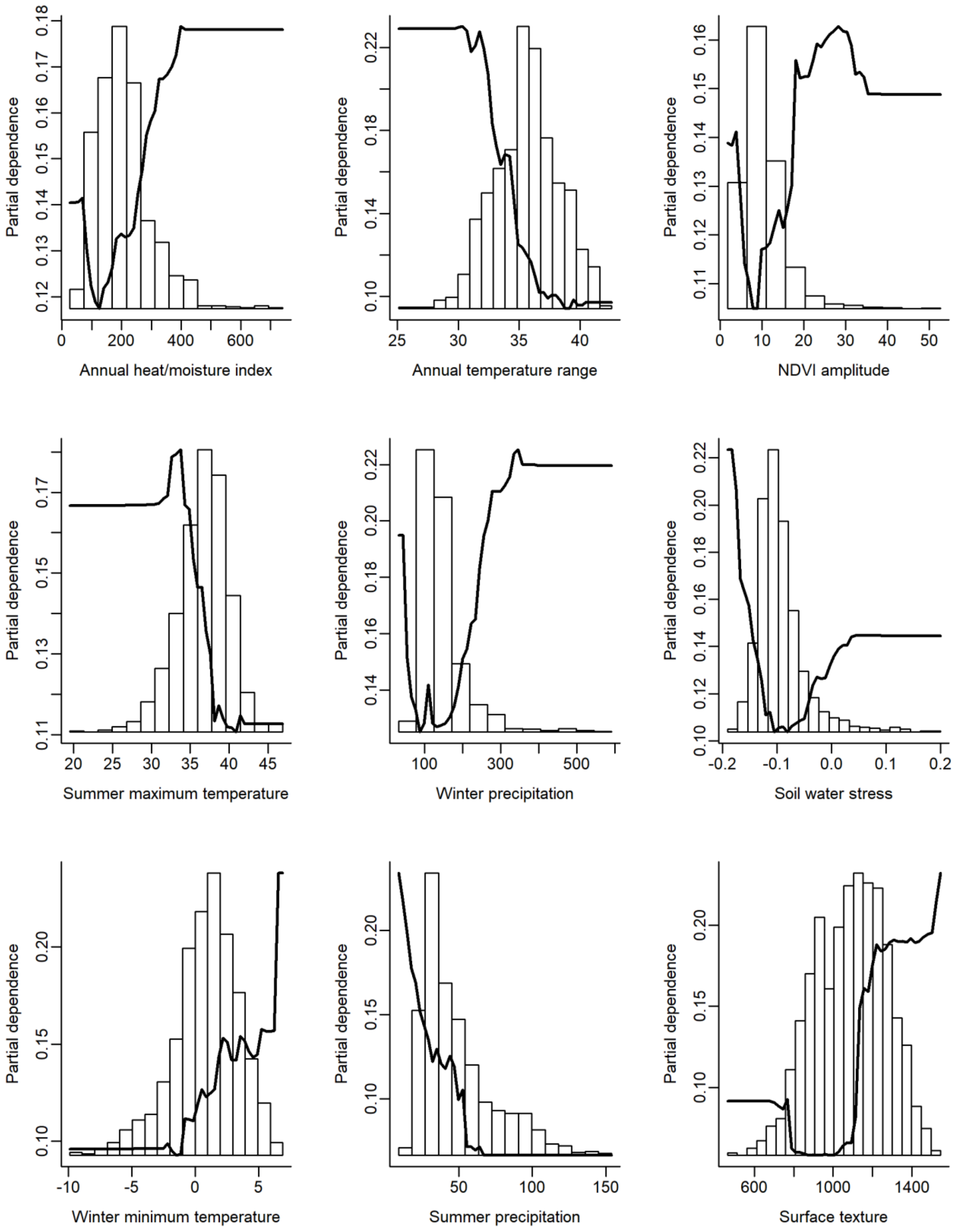

Random Forest response curves for *Astragalus didymocarpus*

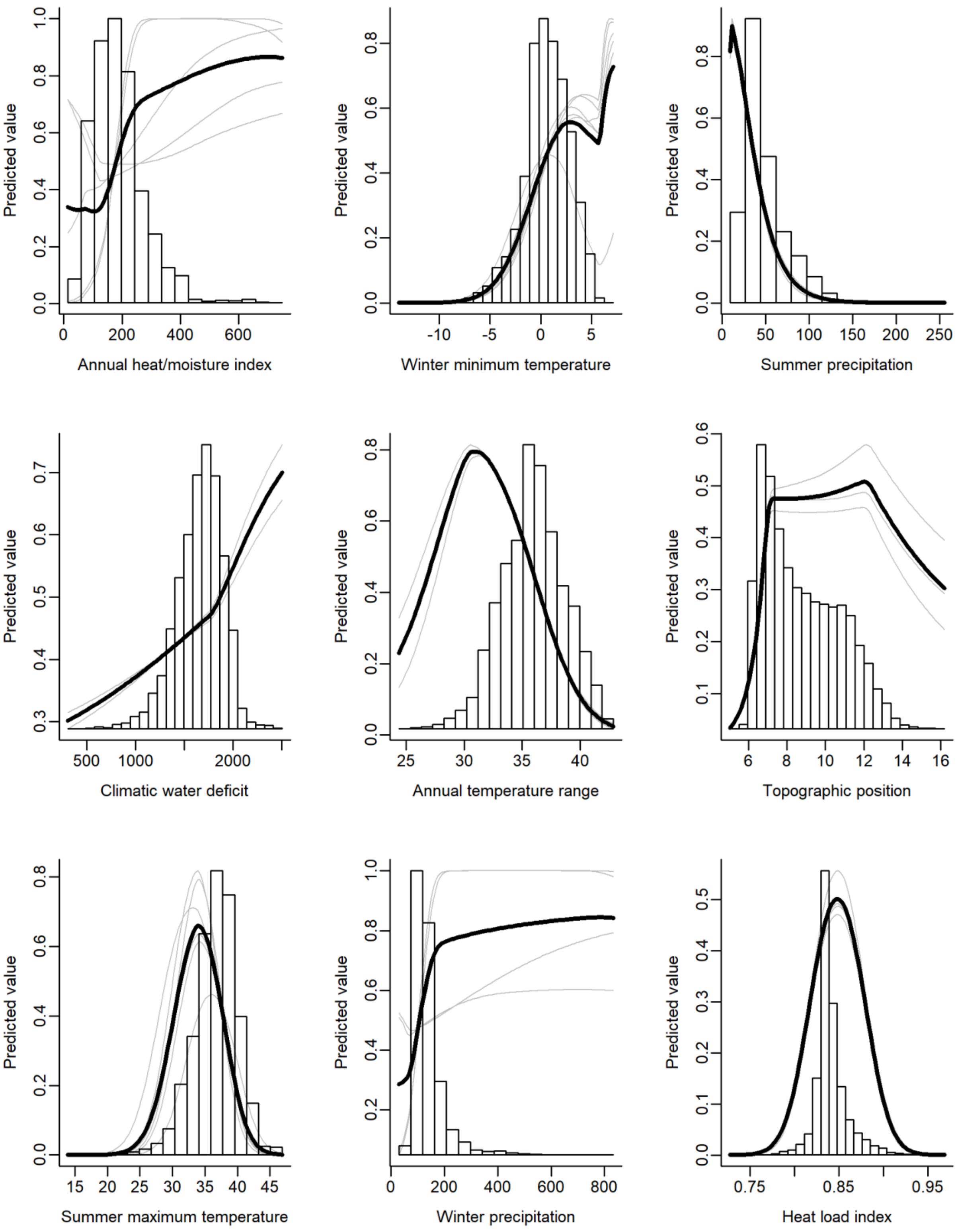

MaxEnt response curves for *Astragalus didymocarpus*

8. *Asclepias erosa* SDM and response curves (GAM, Random Forest, Maxent)

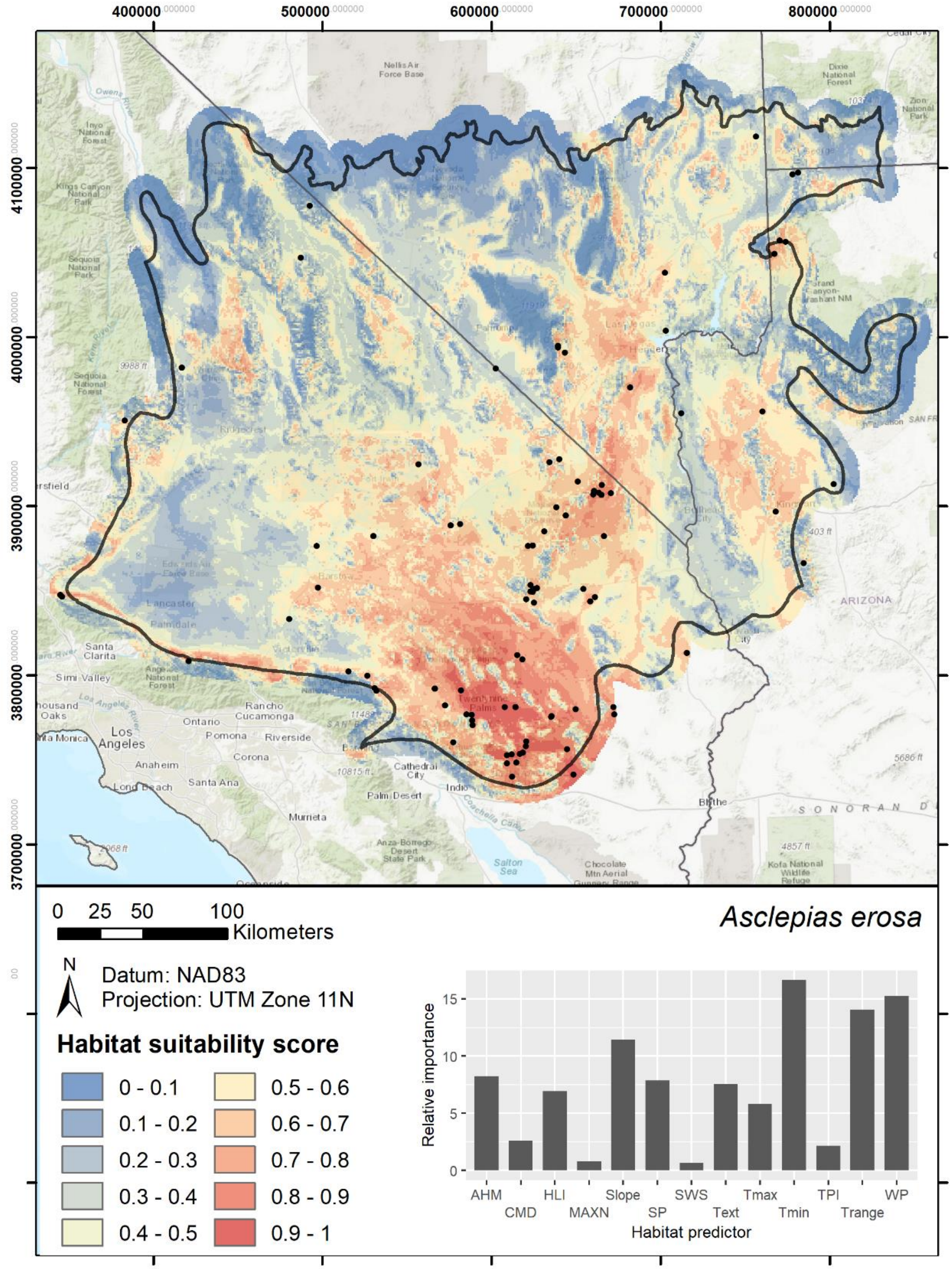

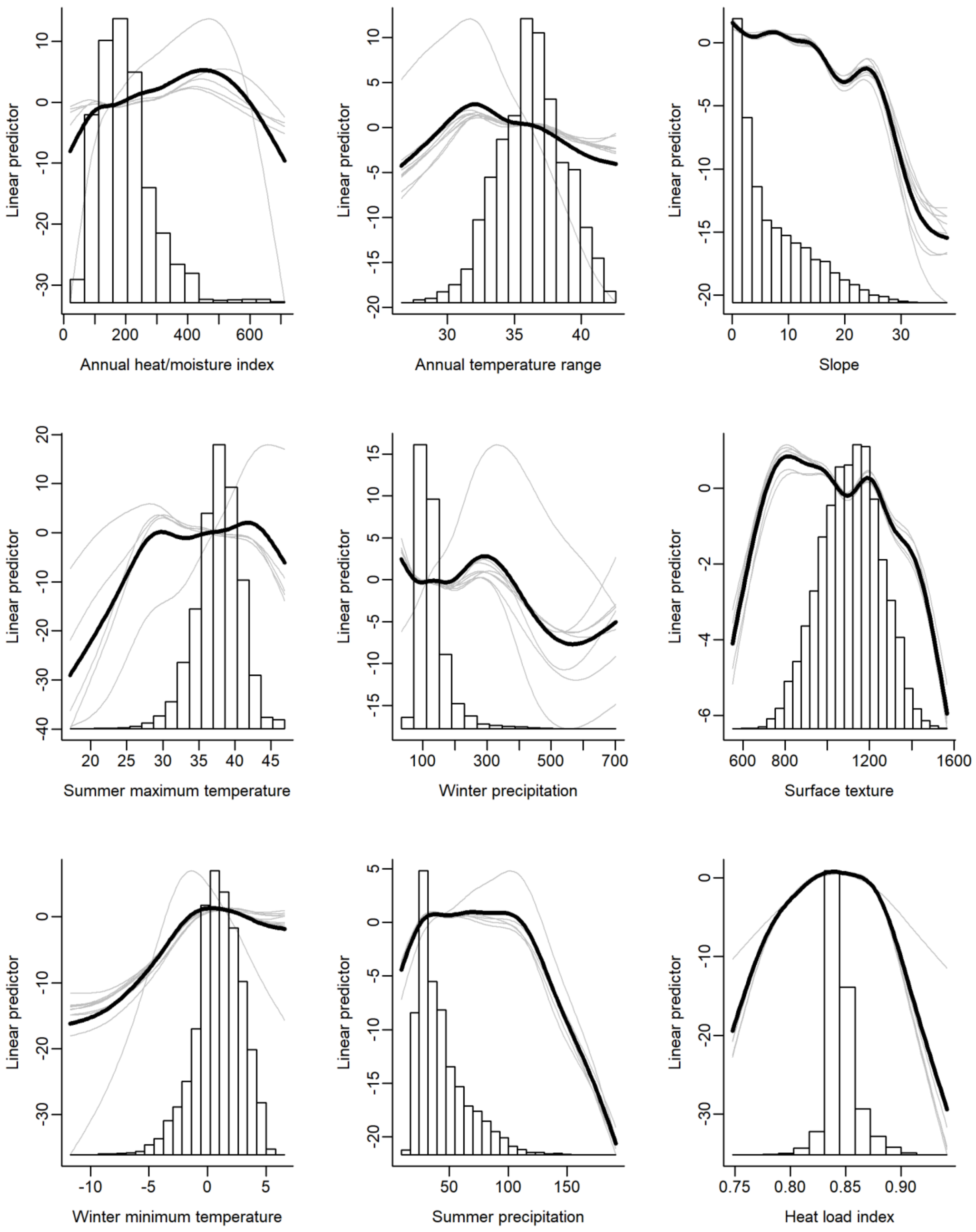

GAM response curves for *Asclepias erosa*

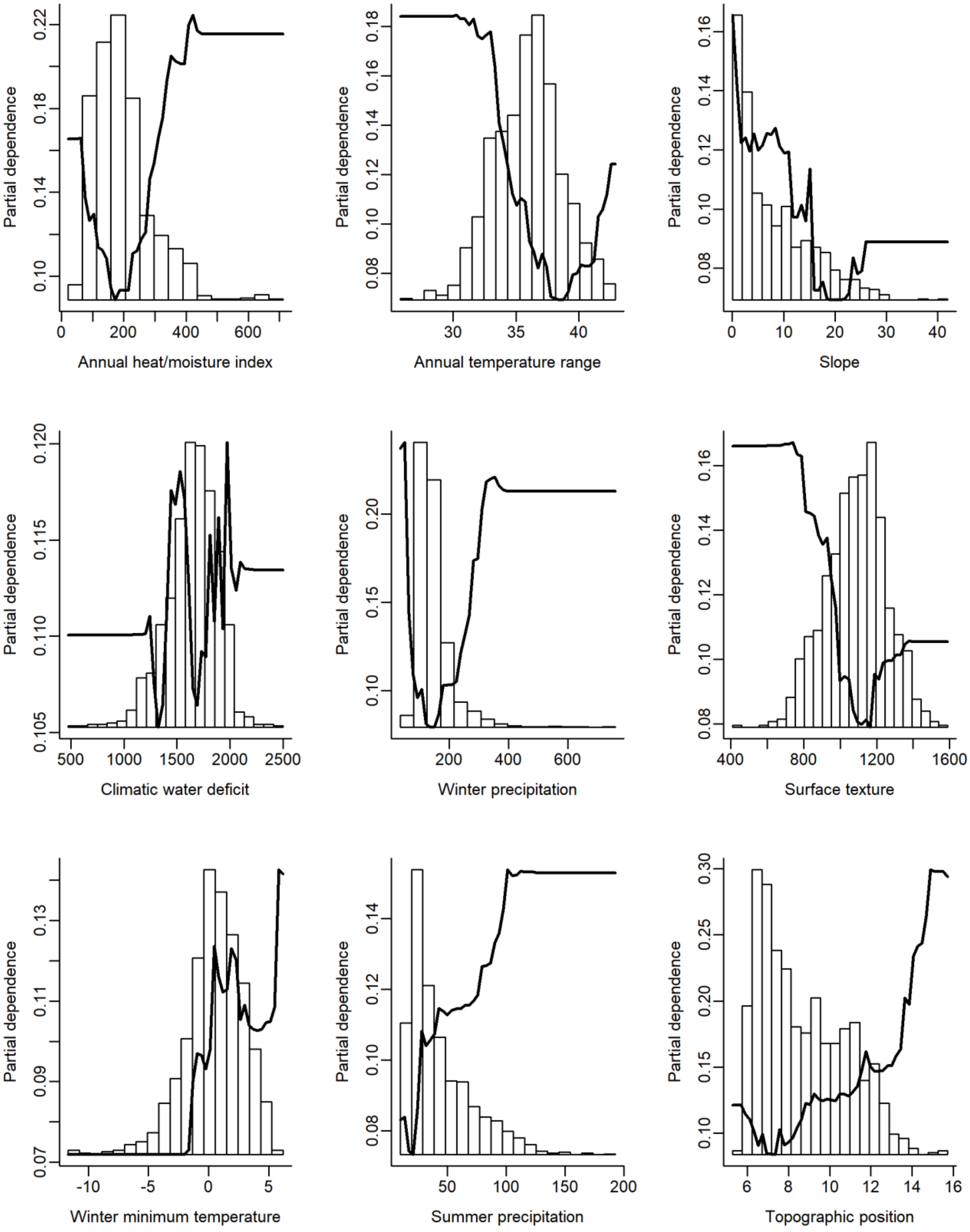

Random Forest response curves for *Asclepias erosa*

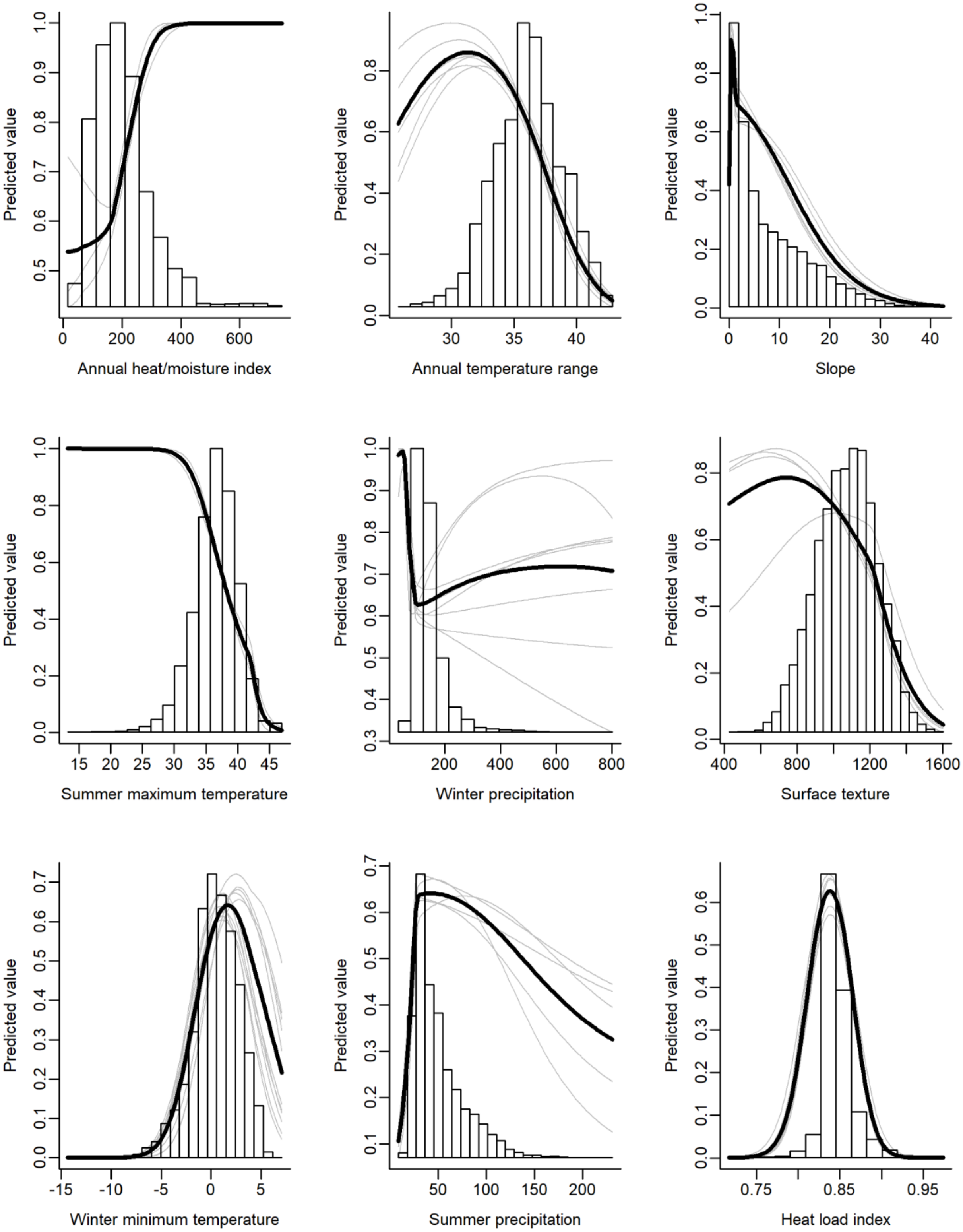

MaxEnt response curves for *Asclepias erosa*

9. *Astragalus layneae* SDM and response curves (GAM, Random Forest, Maxent)

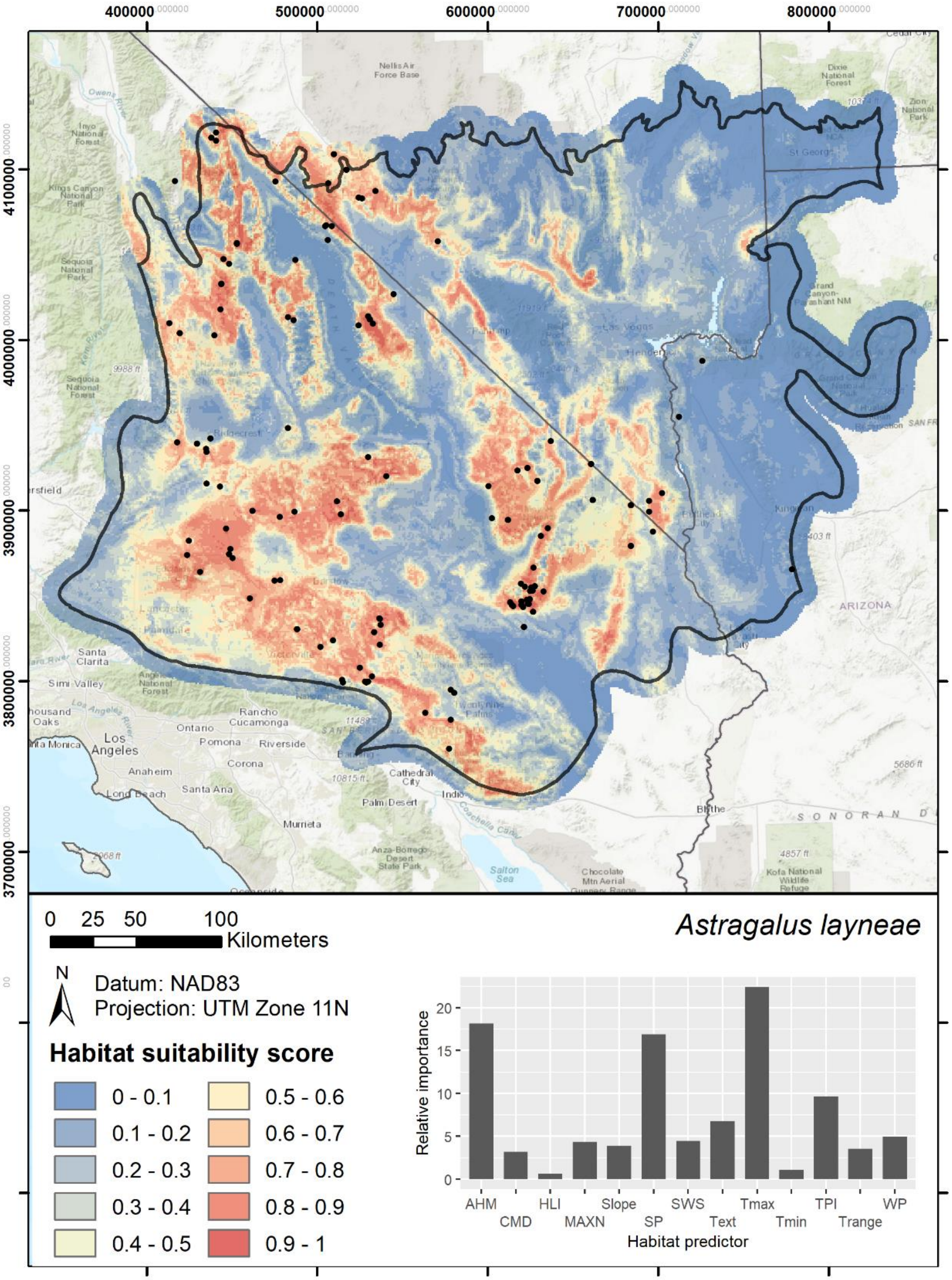

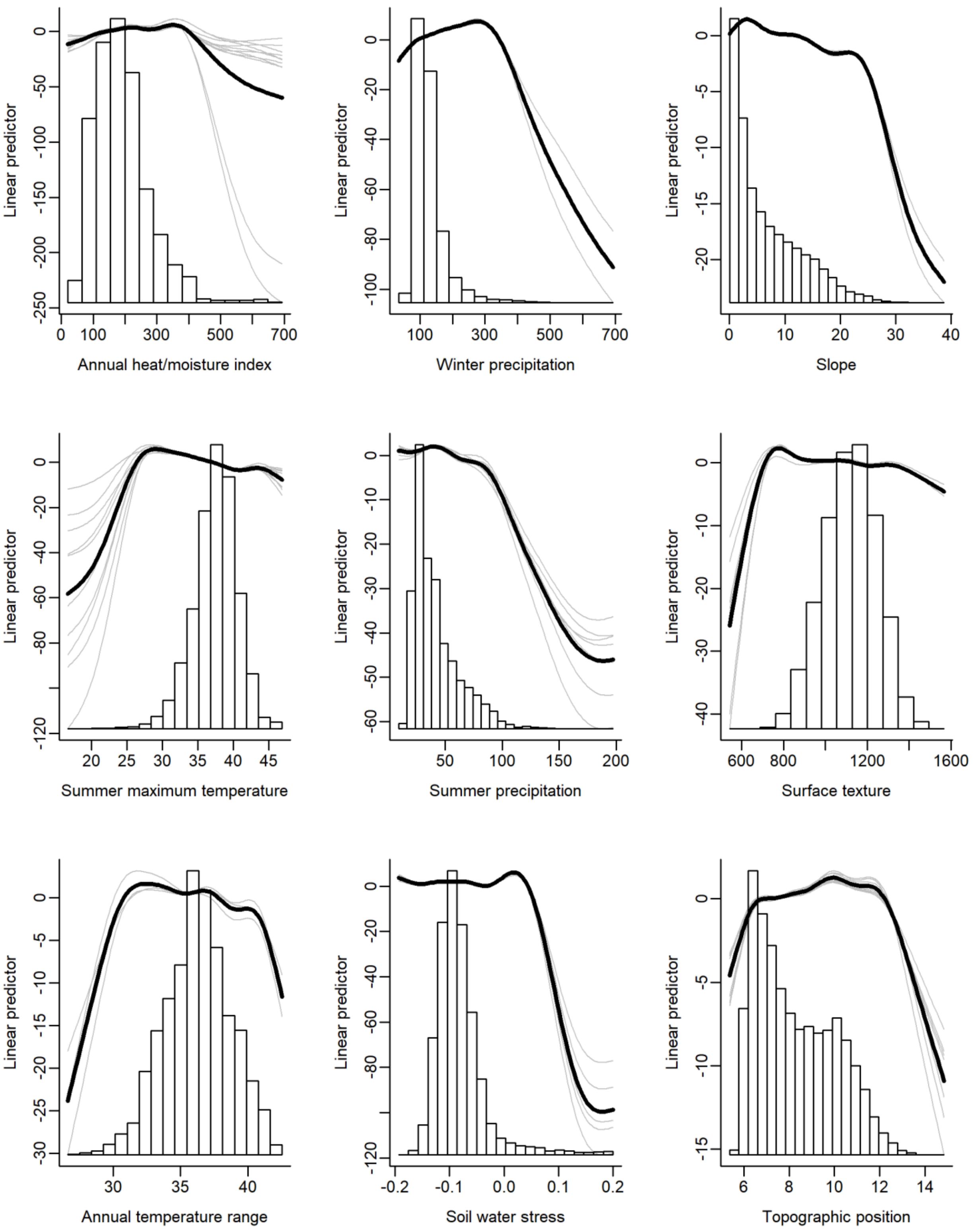

GAM response curves for *Astragalus layneae*

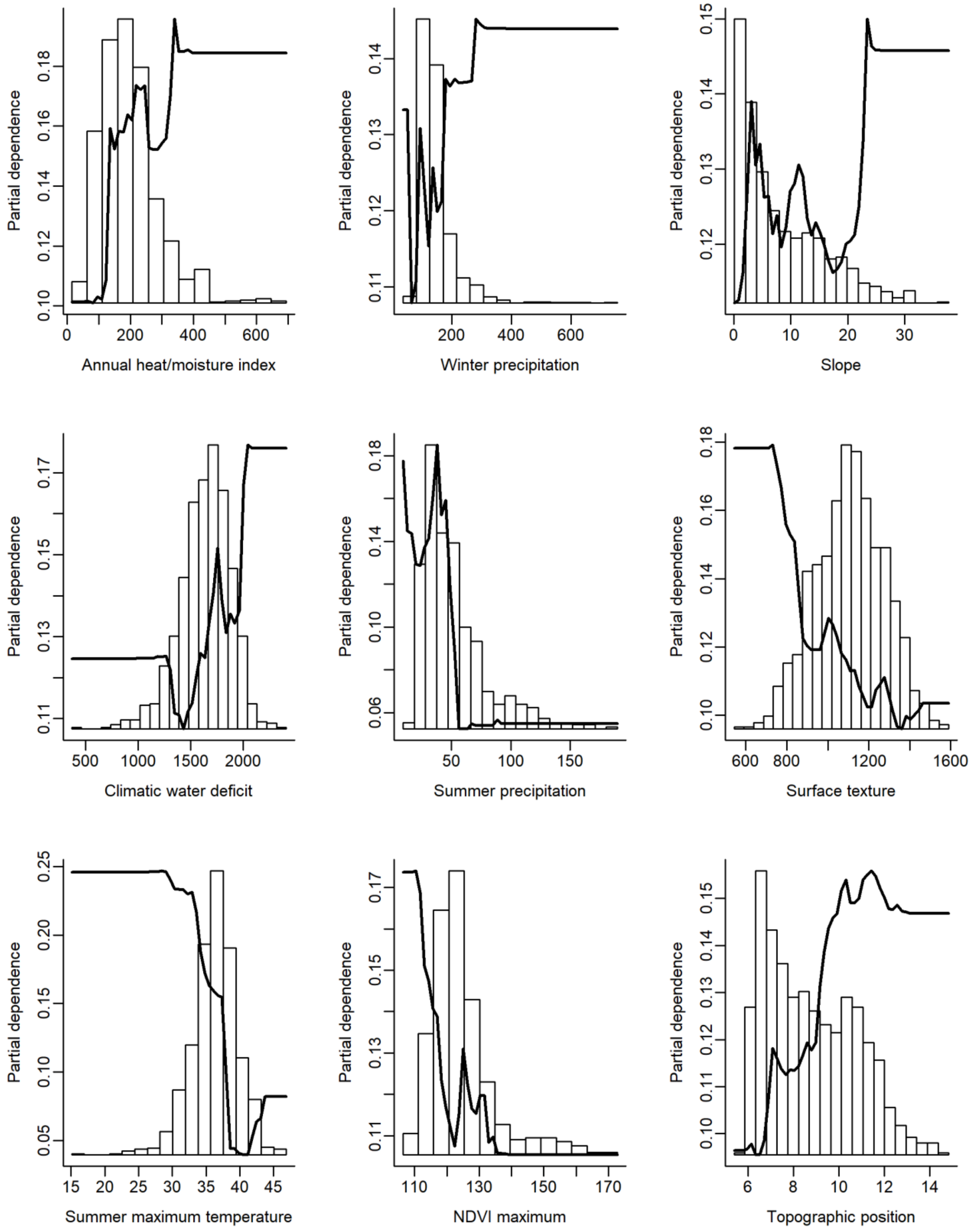

Random Forest response curves for *Astragalus layneae*

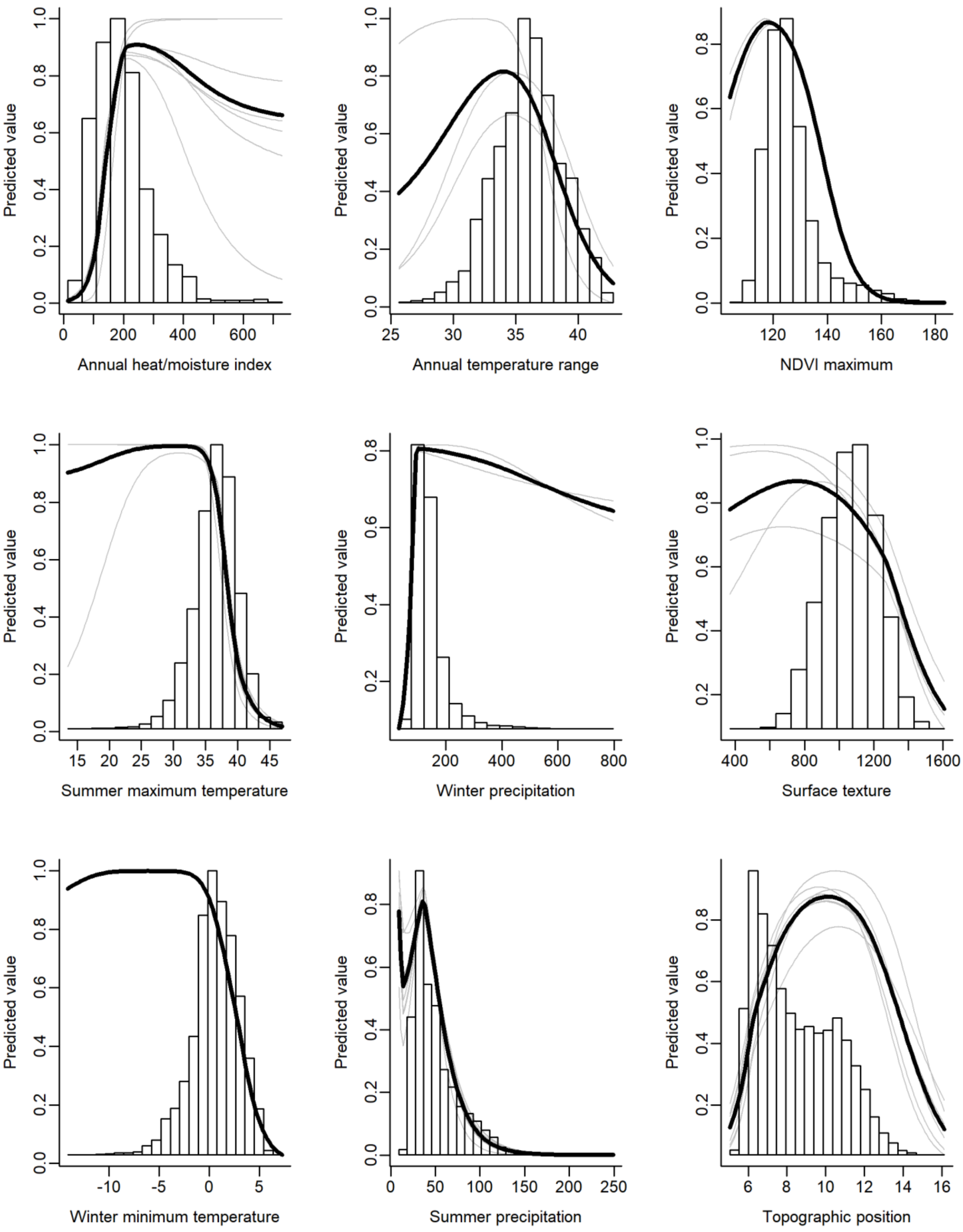

MaxEnt response curves for *Astragalus layneae*

10. *Atriplex hymenelytra* SDM and response curves (GAM, Random Forest, Maxent)

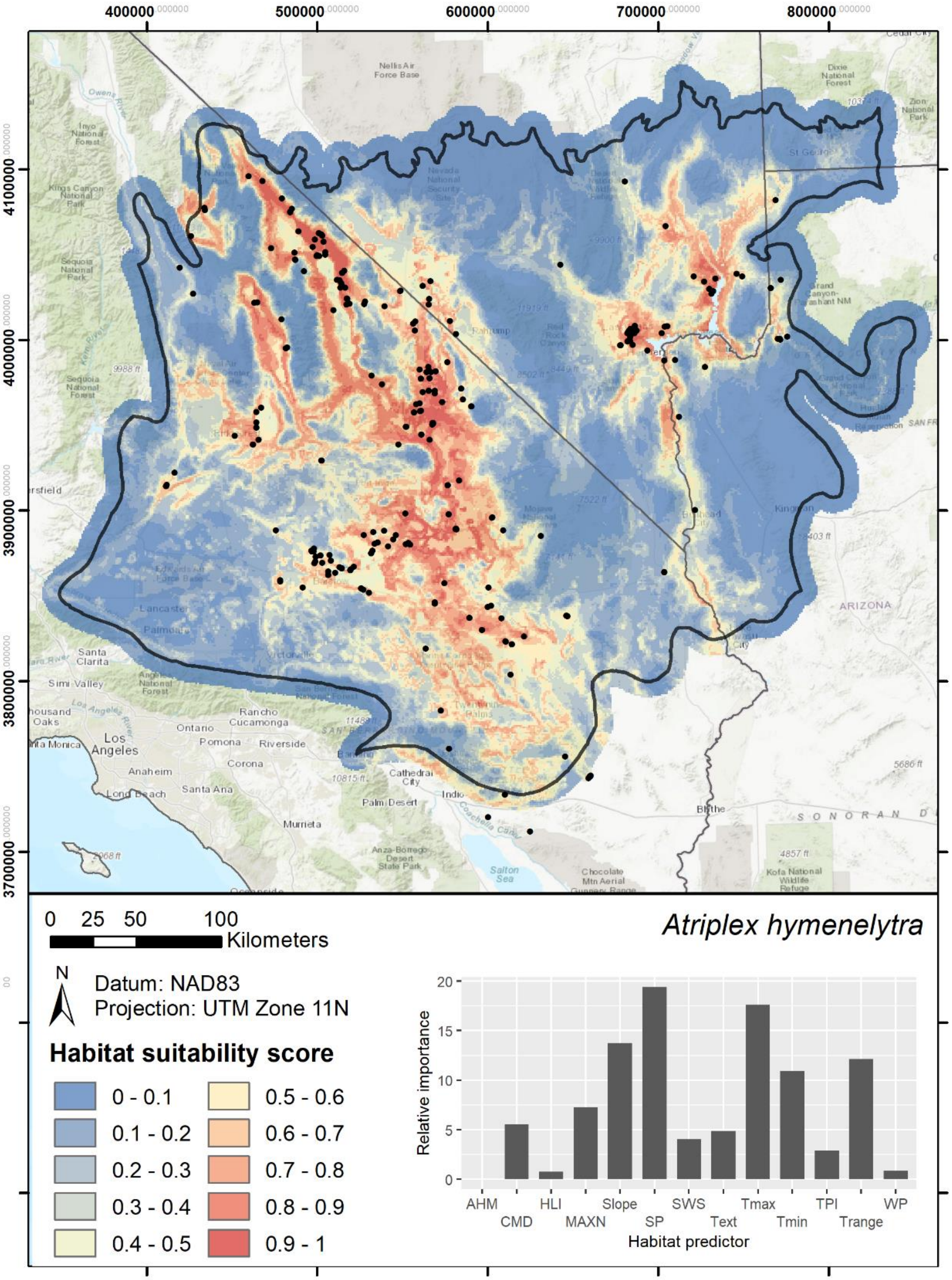

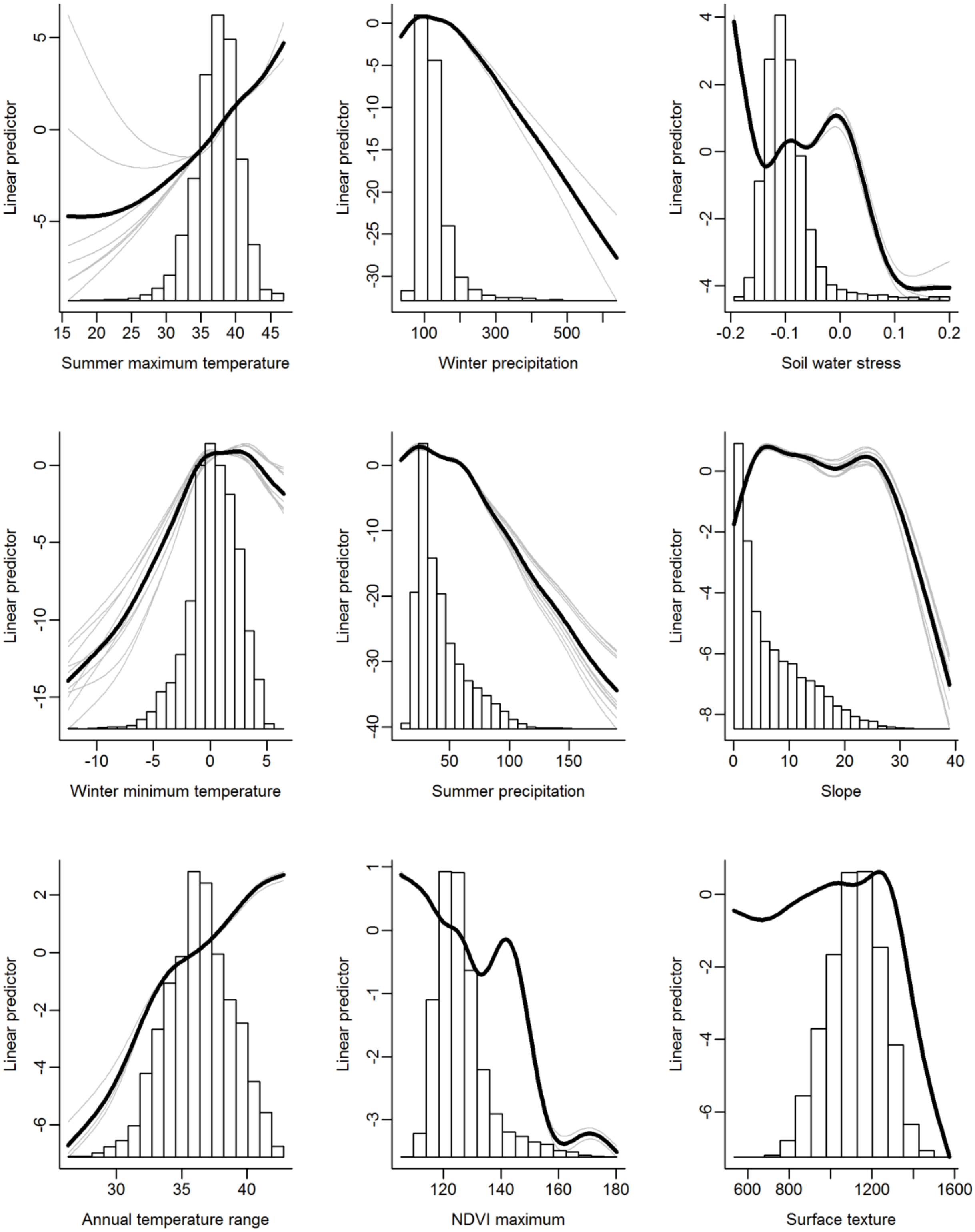

GAM response curves for *Atriplex hymenelytra*

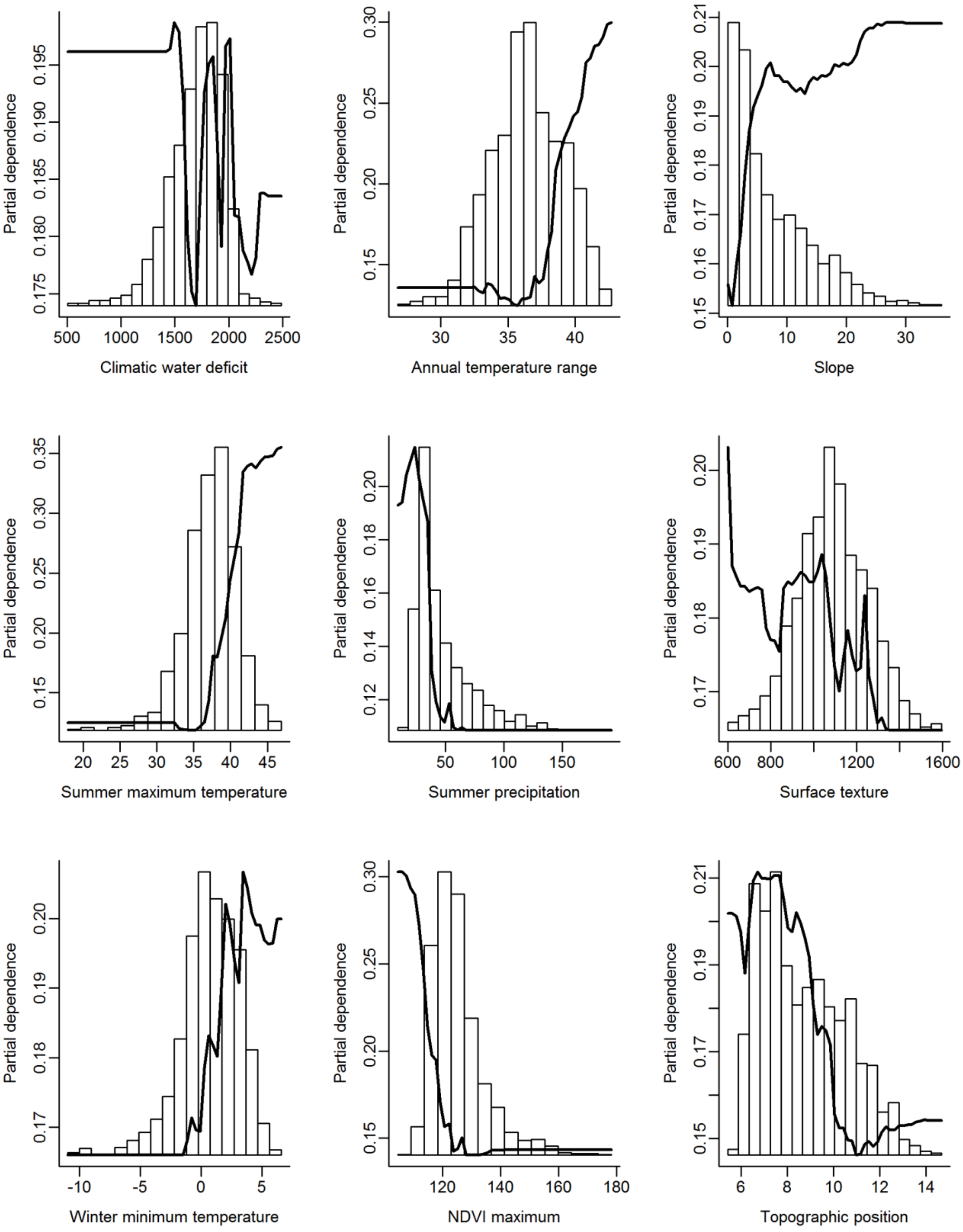

Random Forest response curves for *Atriplex hymenelytra*

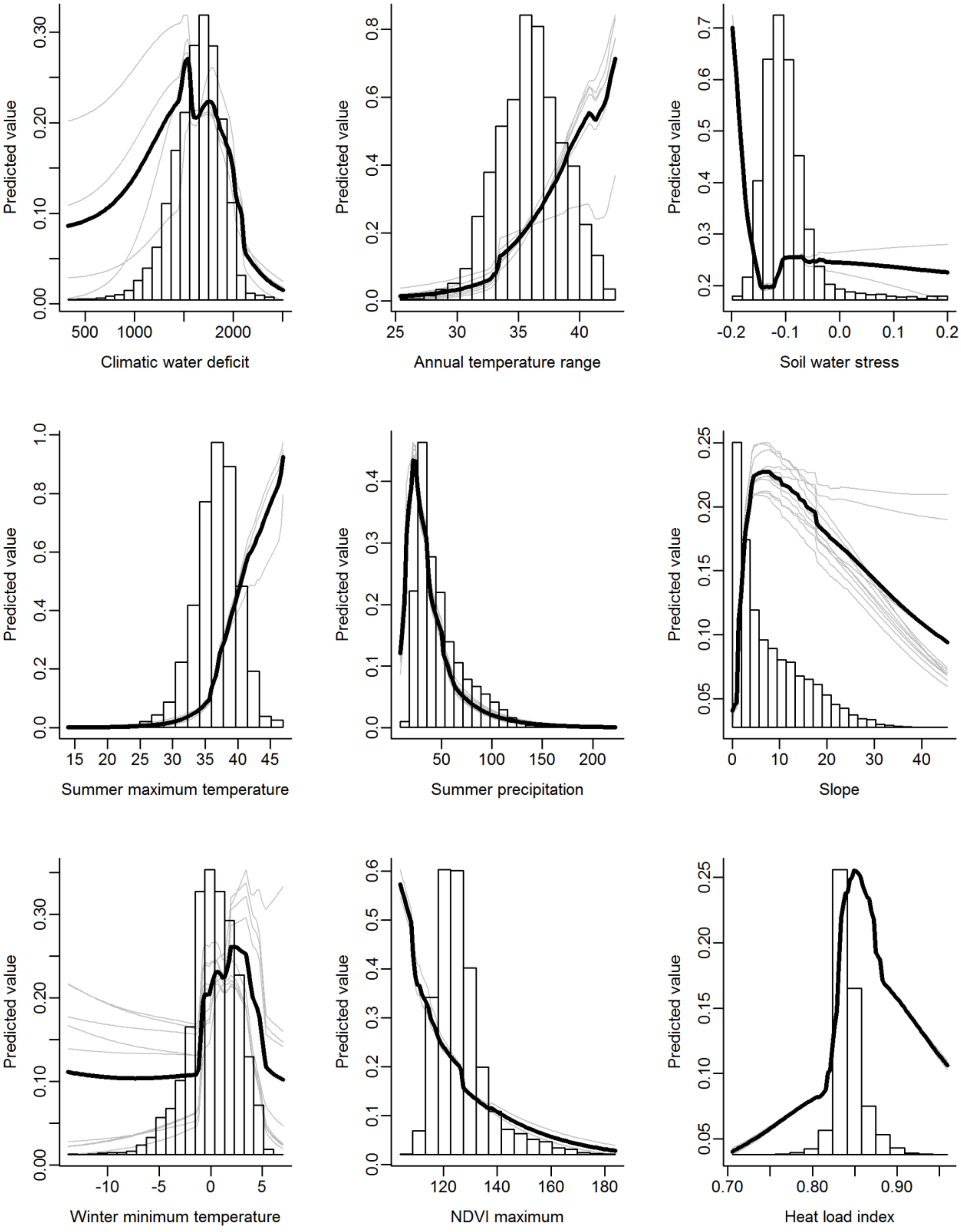

MaxEnt response curves for *Atriplex hymenelytra*

11. *Baileya multiradiata* SDM and response curves (GAM, Random Forest, Maxent)

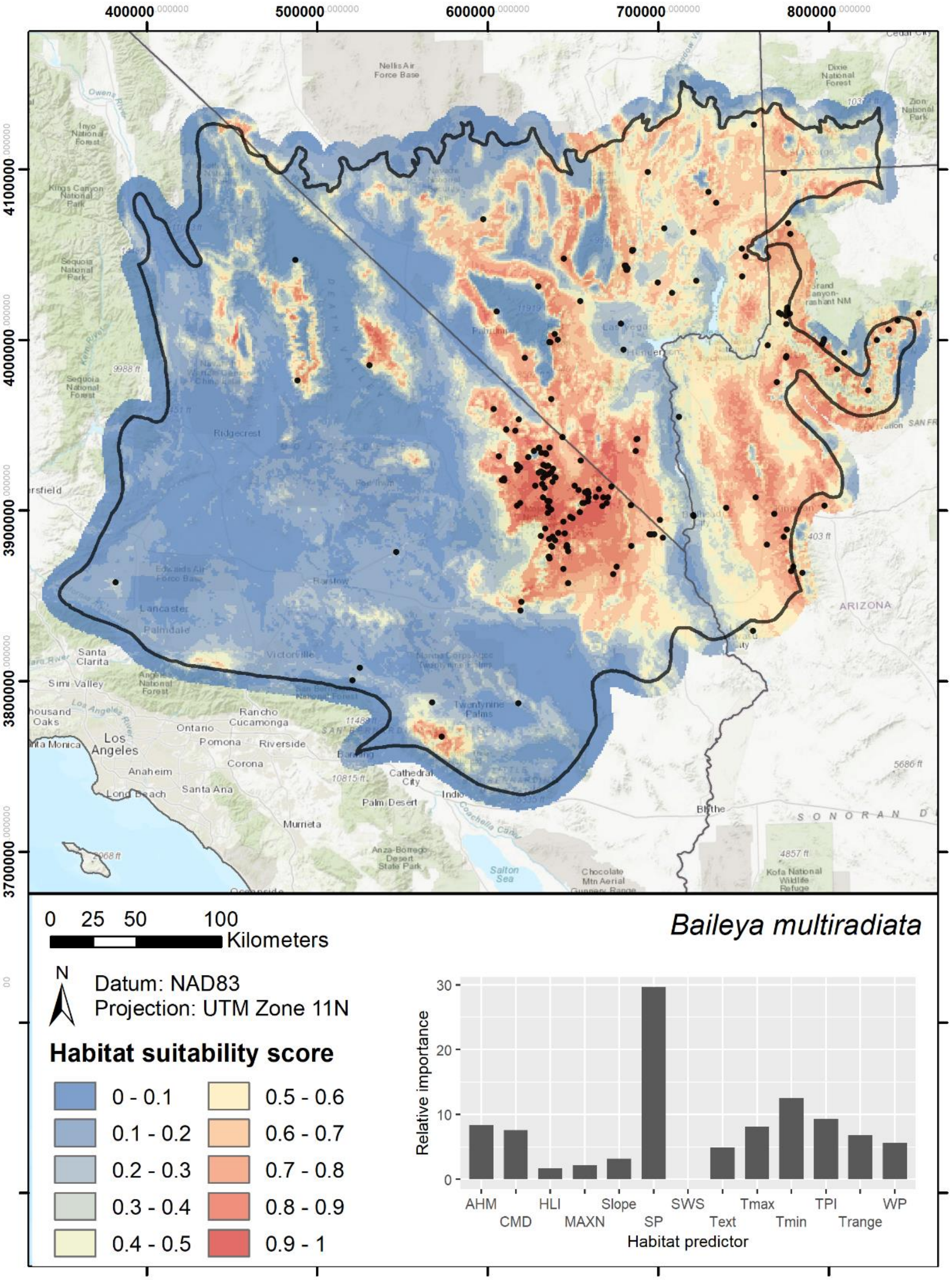

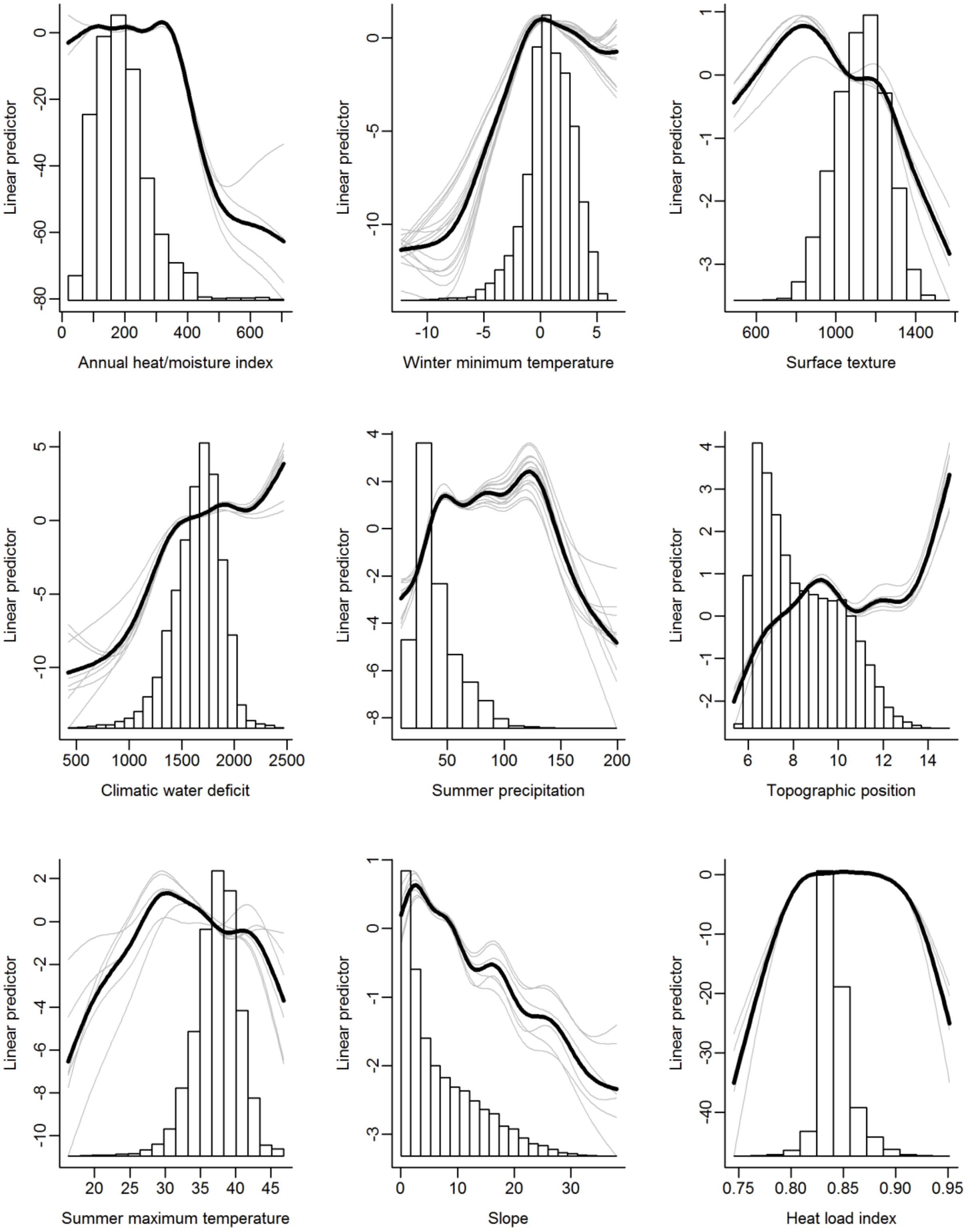

GAM response curves for *Baileya multiradiata*

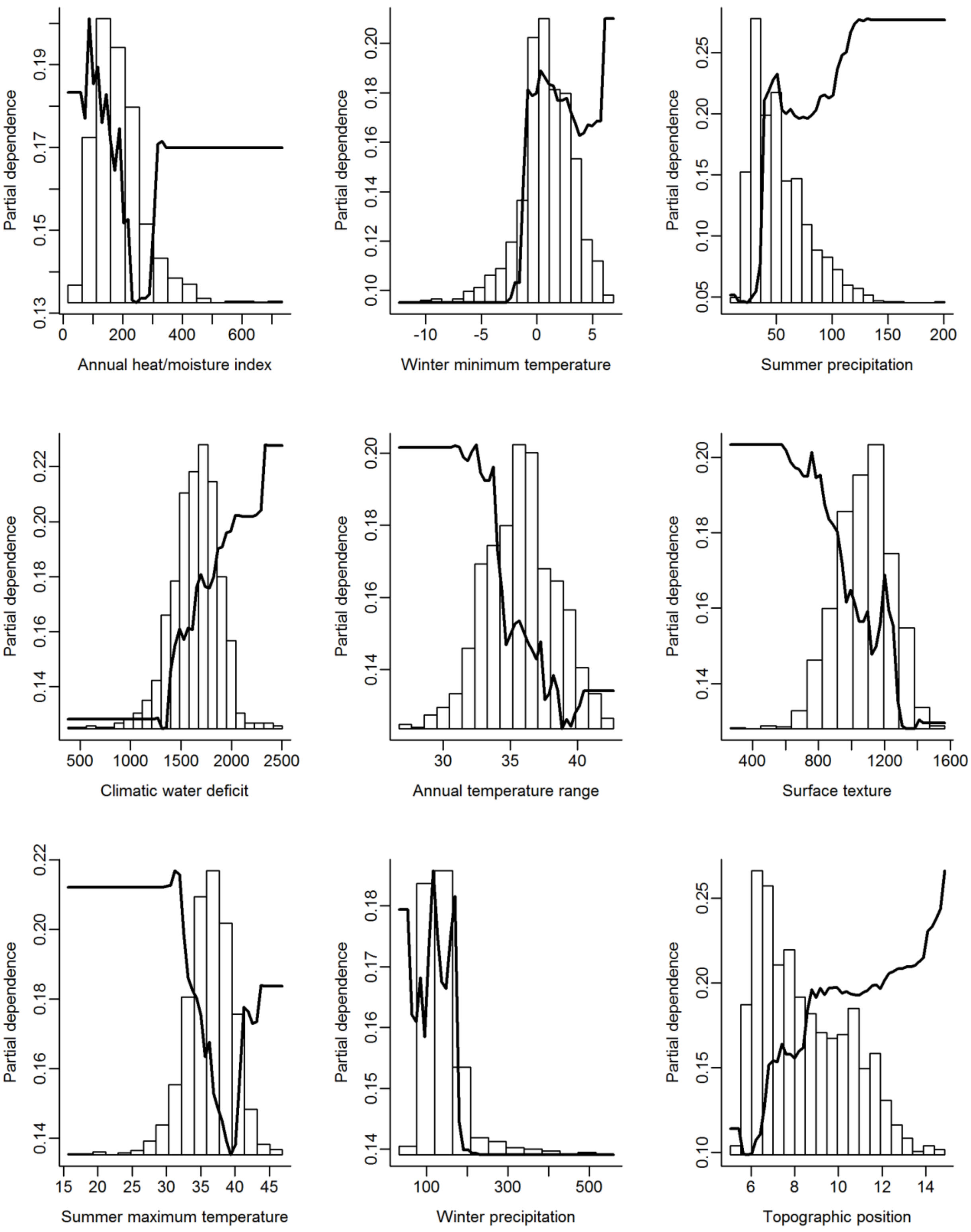

Random Forest response curves for *Baileya multiradiata*

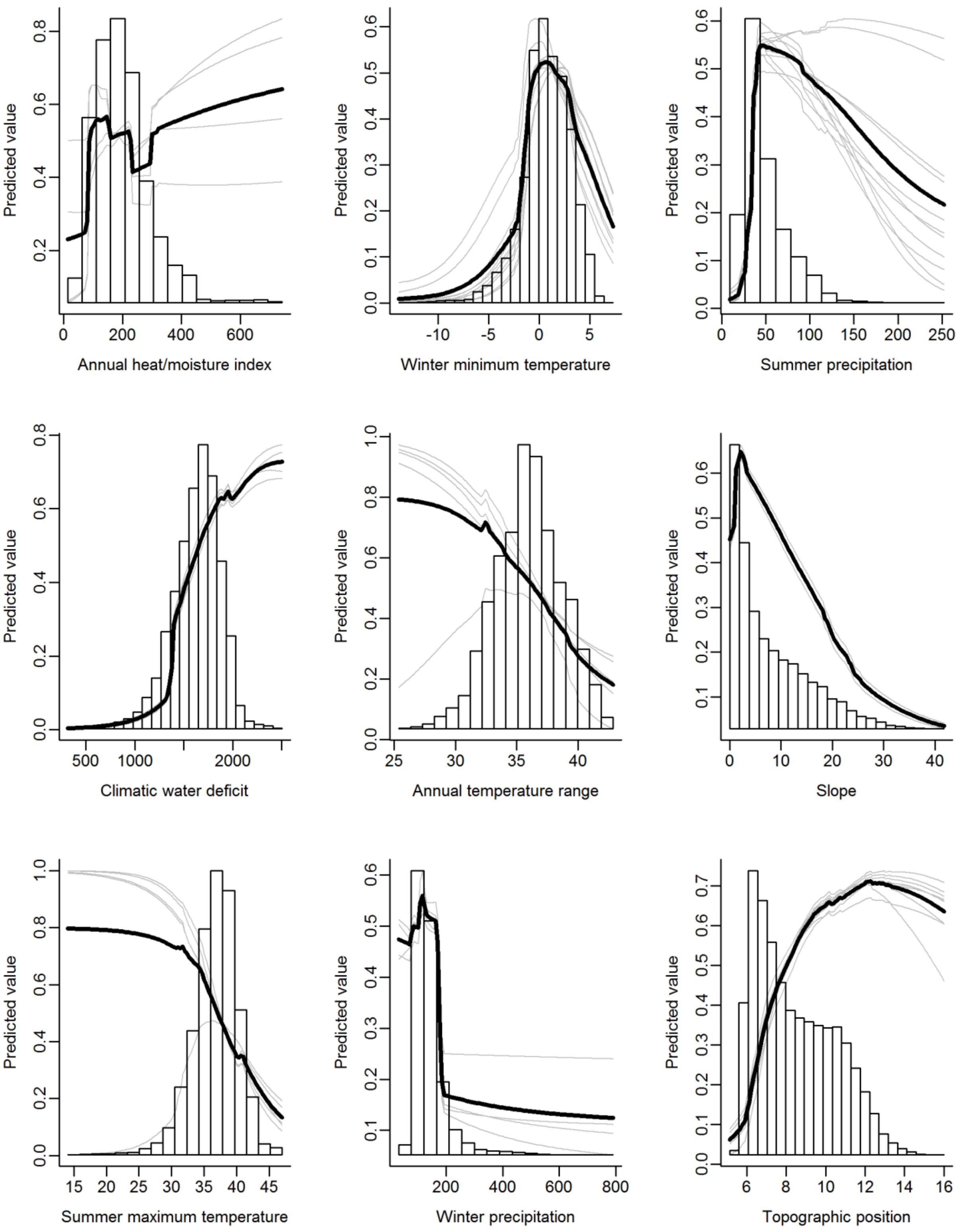

MaxEnt response curves for *Baileya multiradiata*

12. *Chylismia brevipes* SDM and response curves (GAM, Random Forest, Maxent)

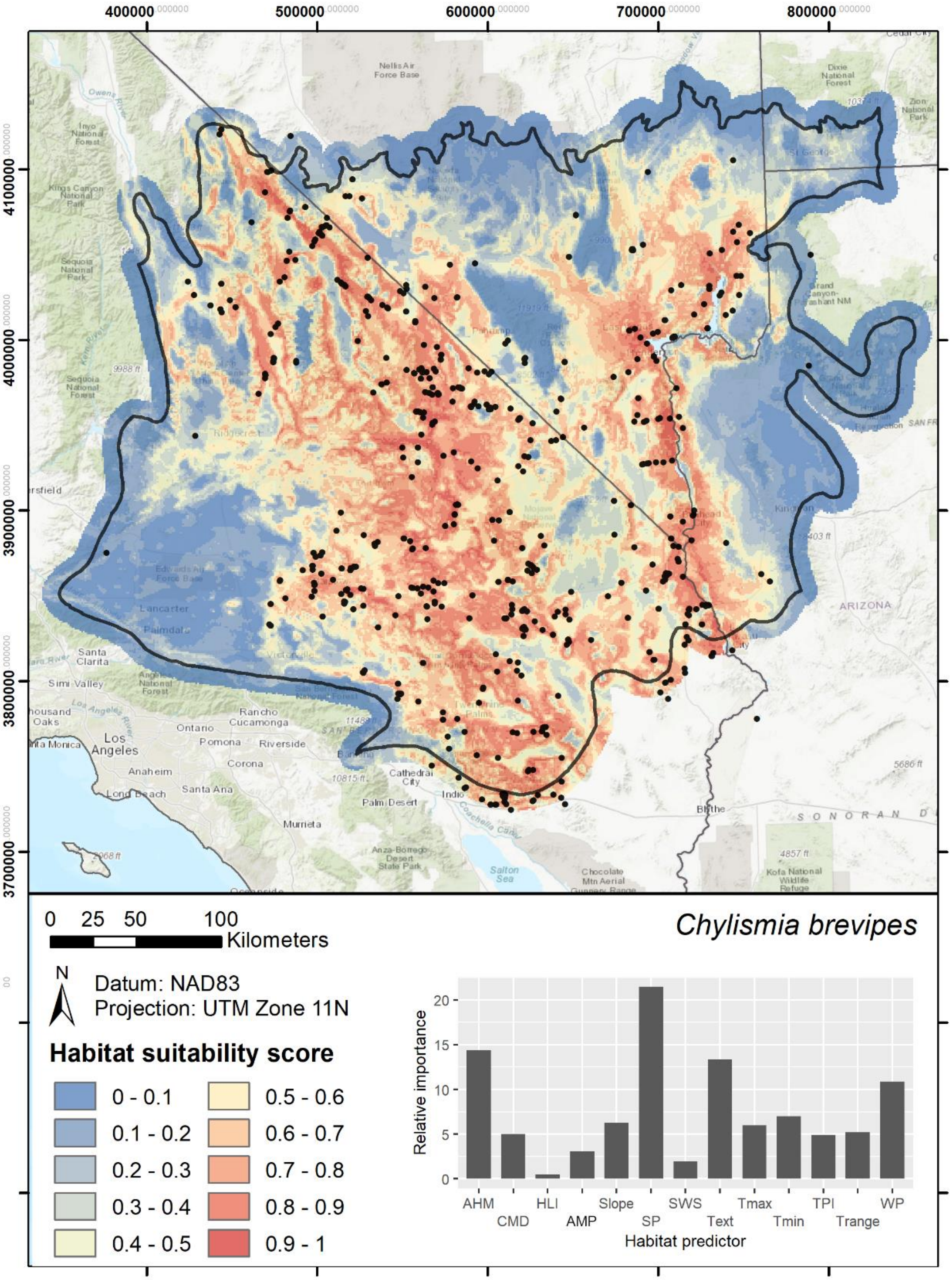

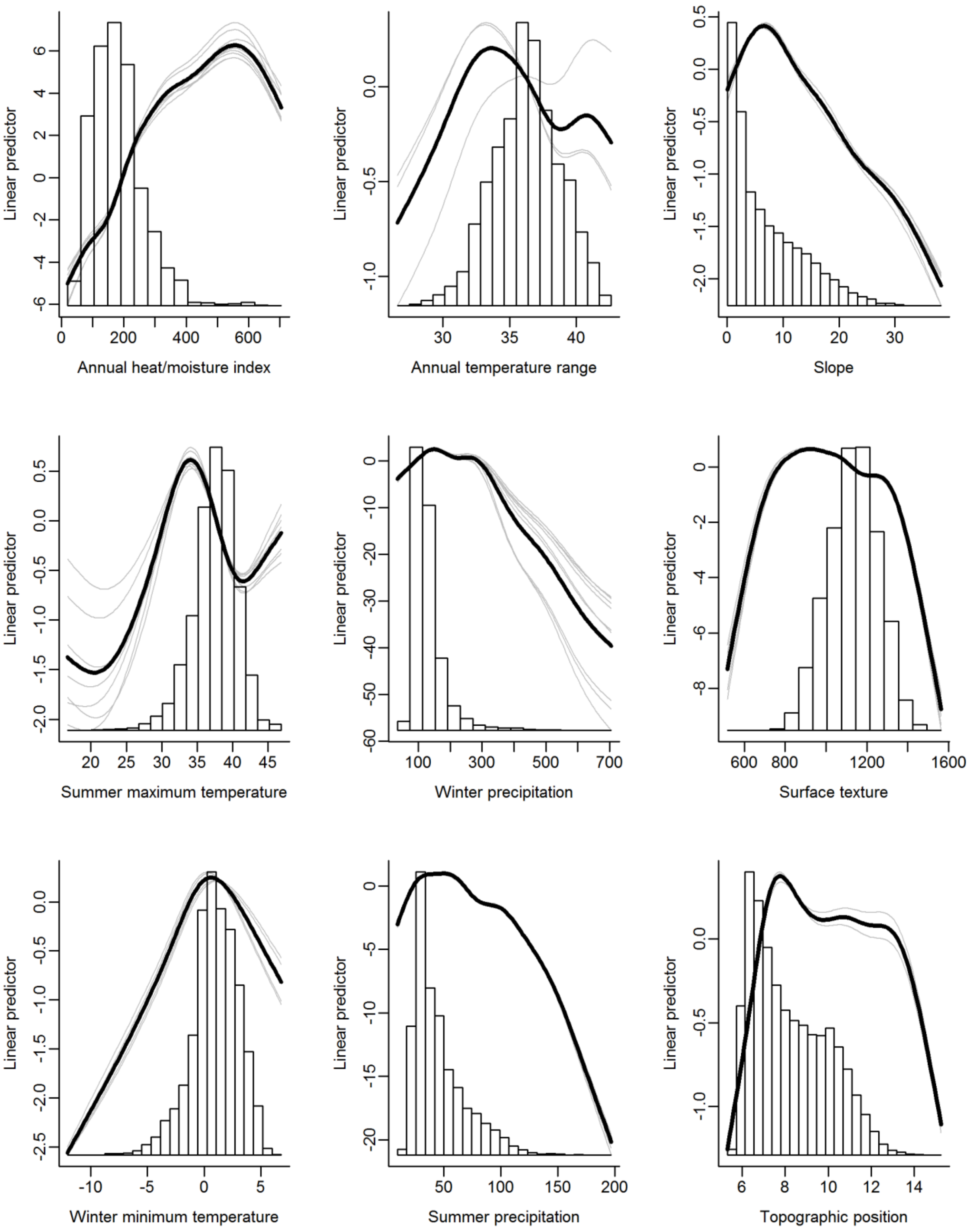

GAM response curves for *Chylismia brevipes*

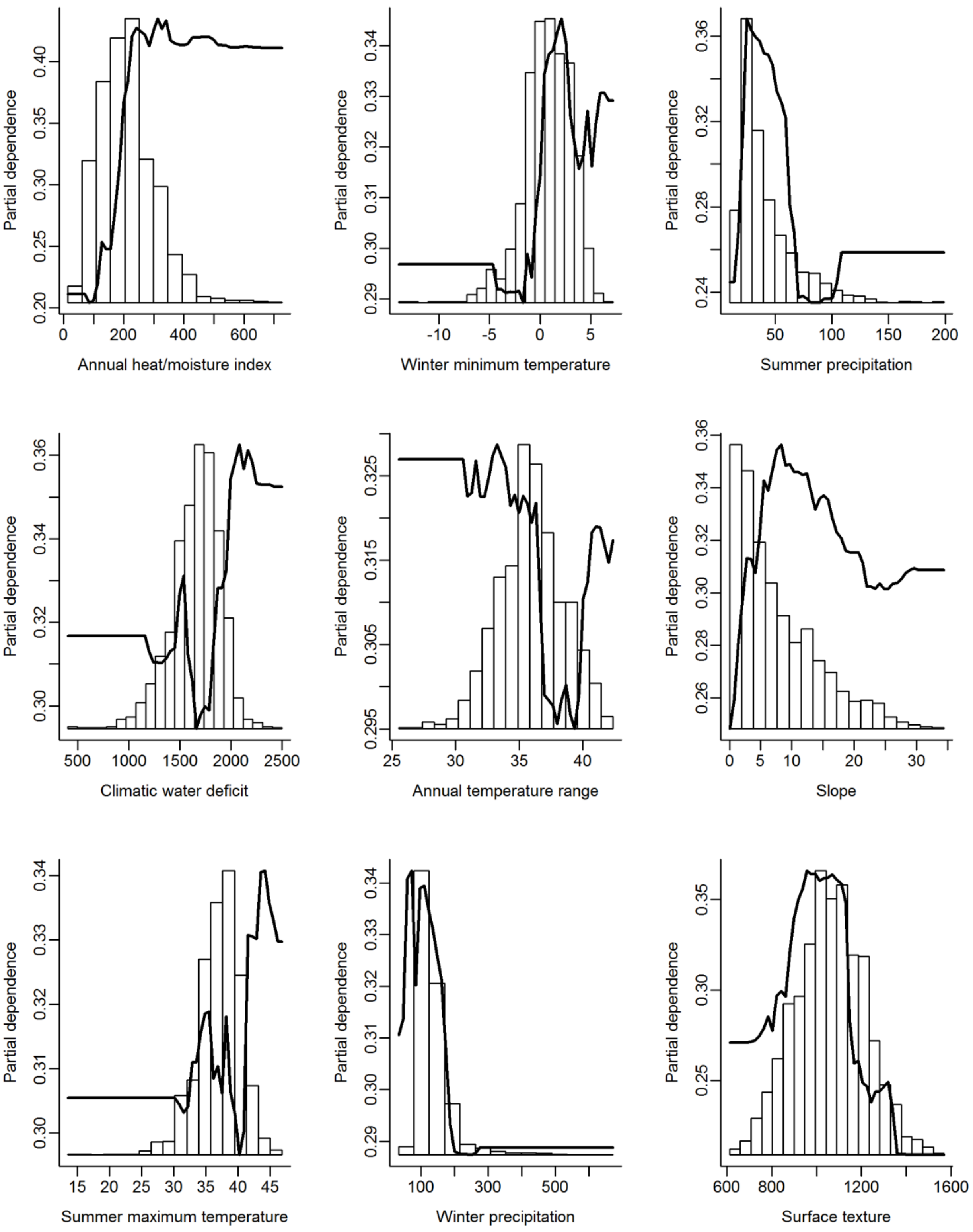

Random Forest response curves for *Chylismia brevipes*

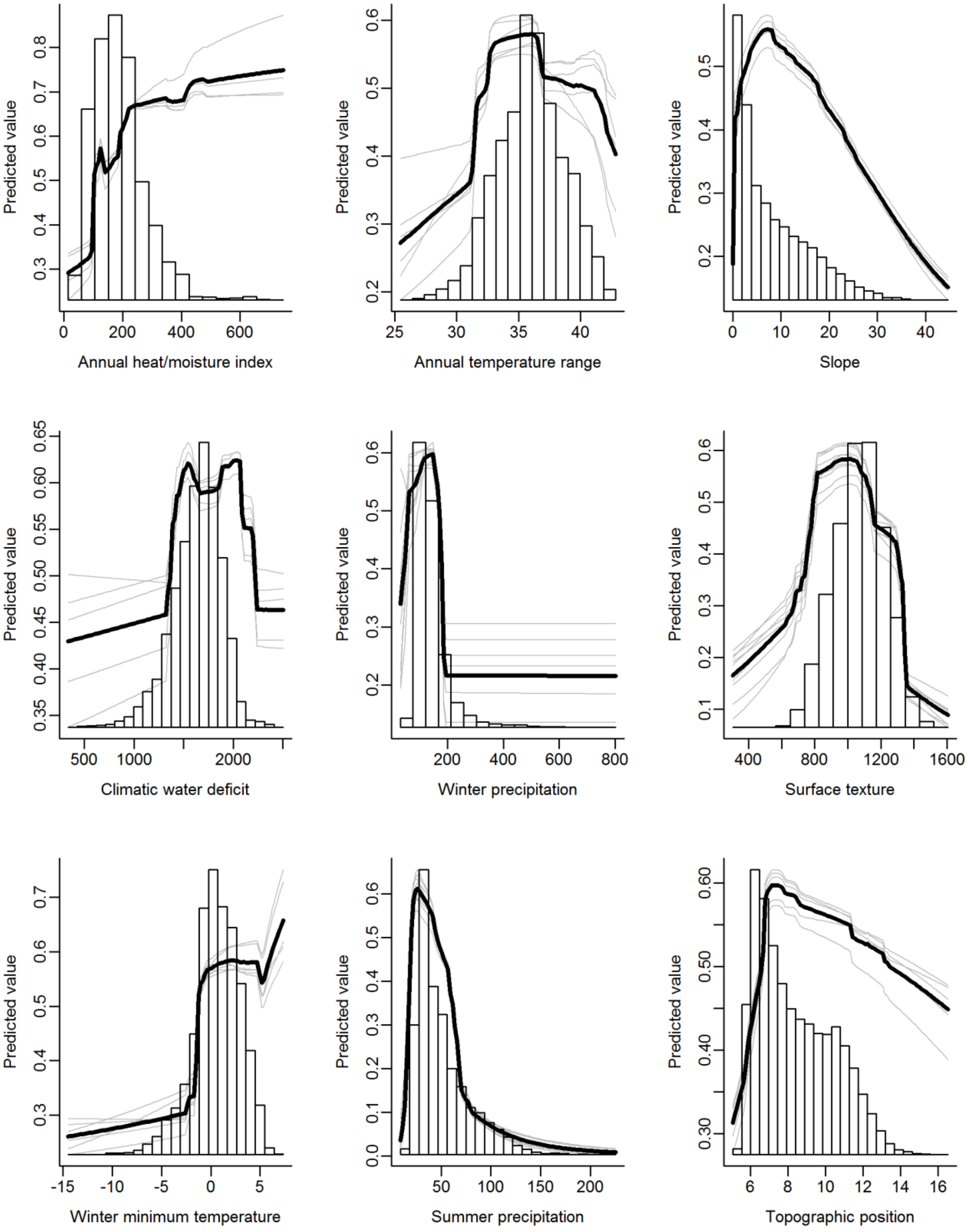

MaxEnt response curves for *Chylismia brevipes*

13. *Chilopsis linearis* SDM and response curves (GAM, Random Forest, Maxent)

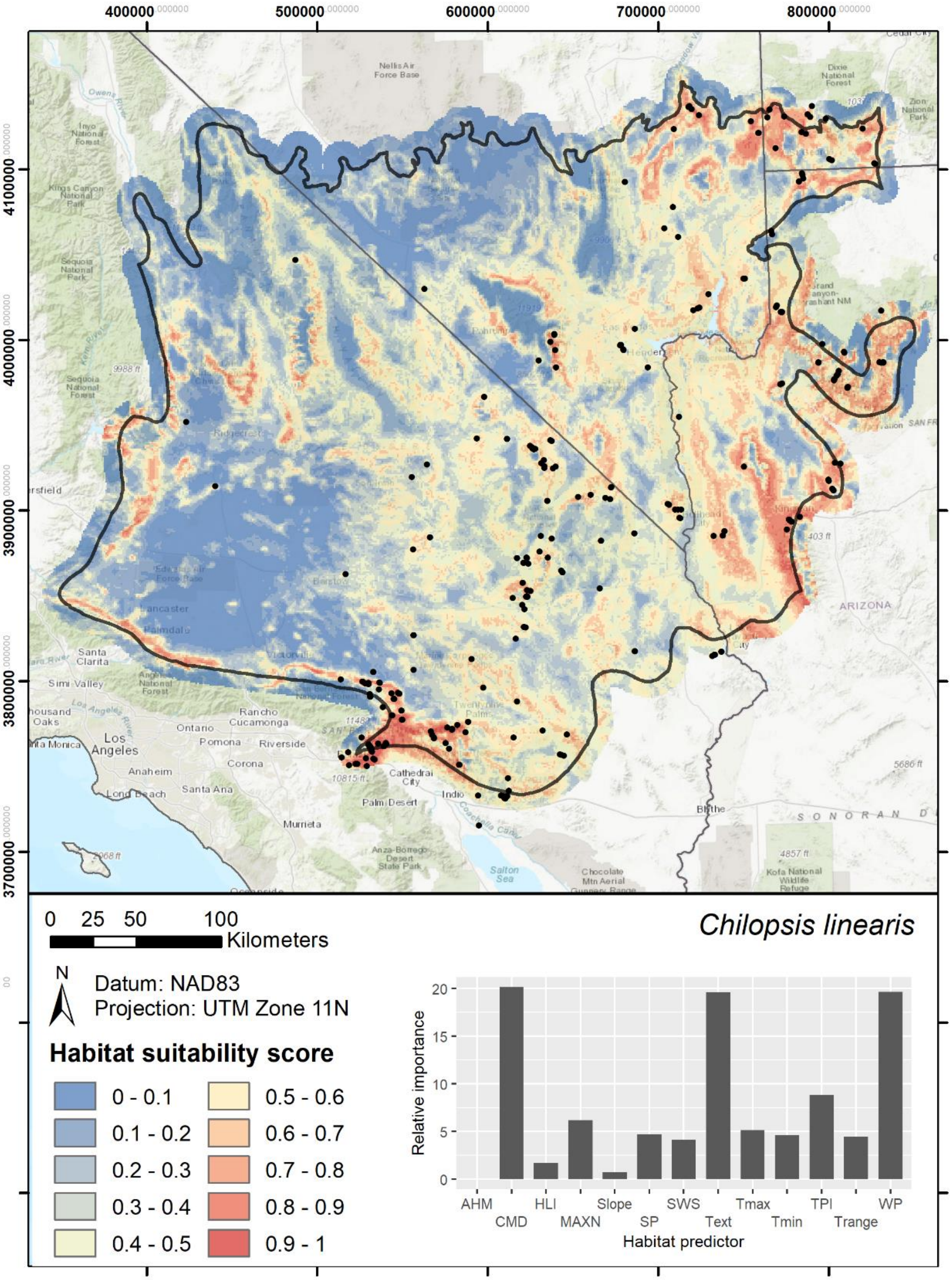

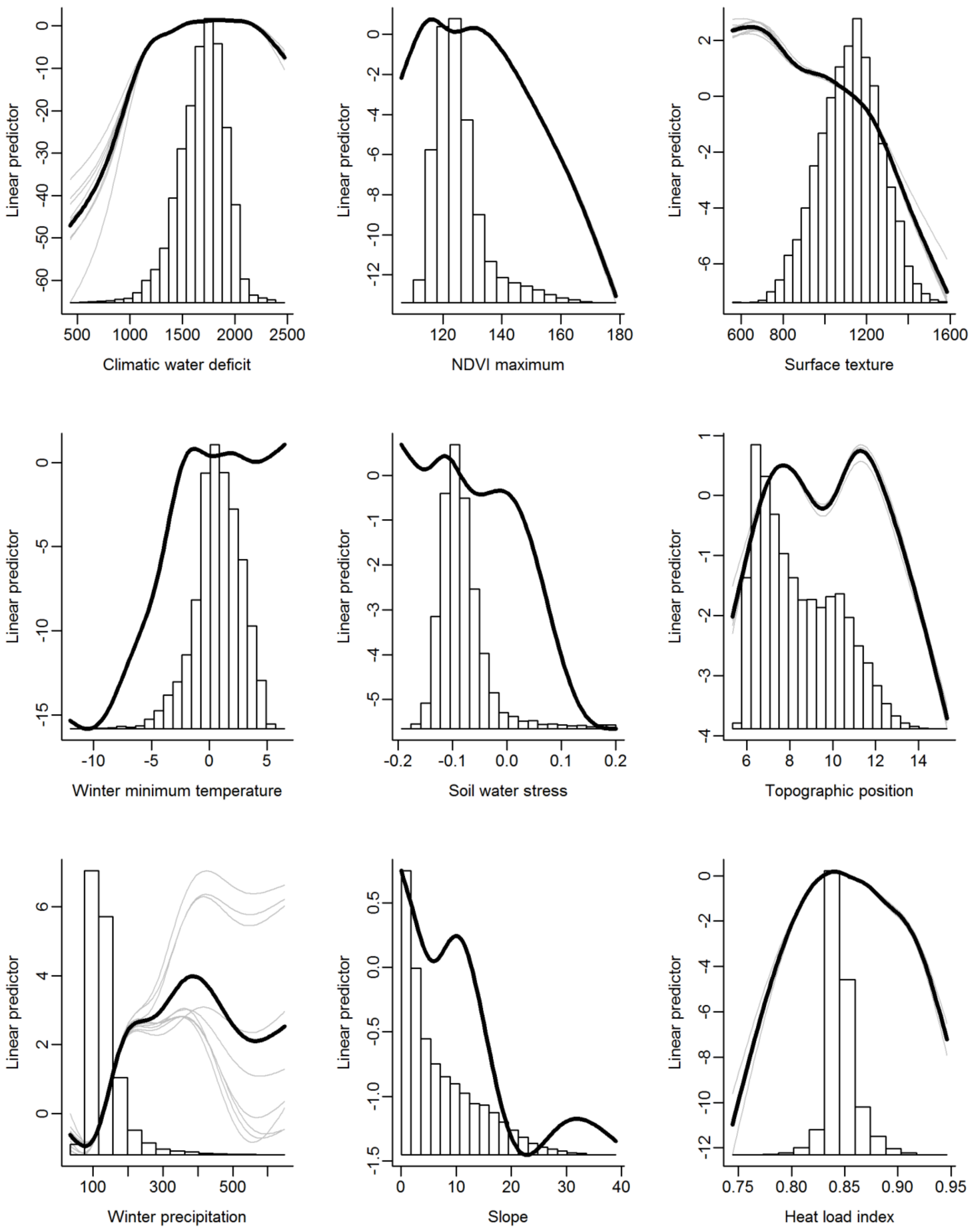

GAM response curves for *Chilopsis linearis*

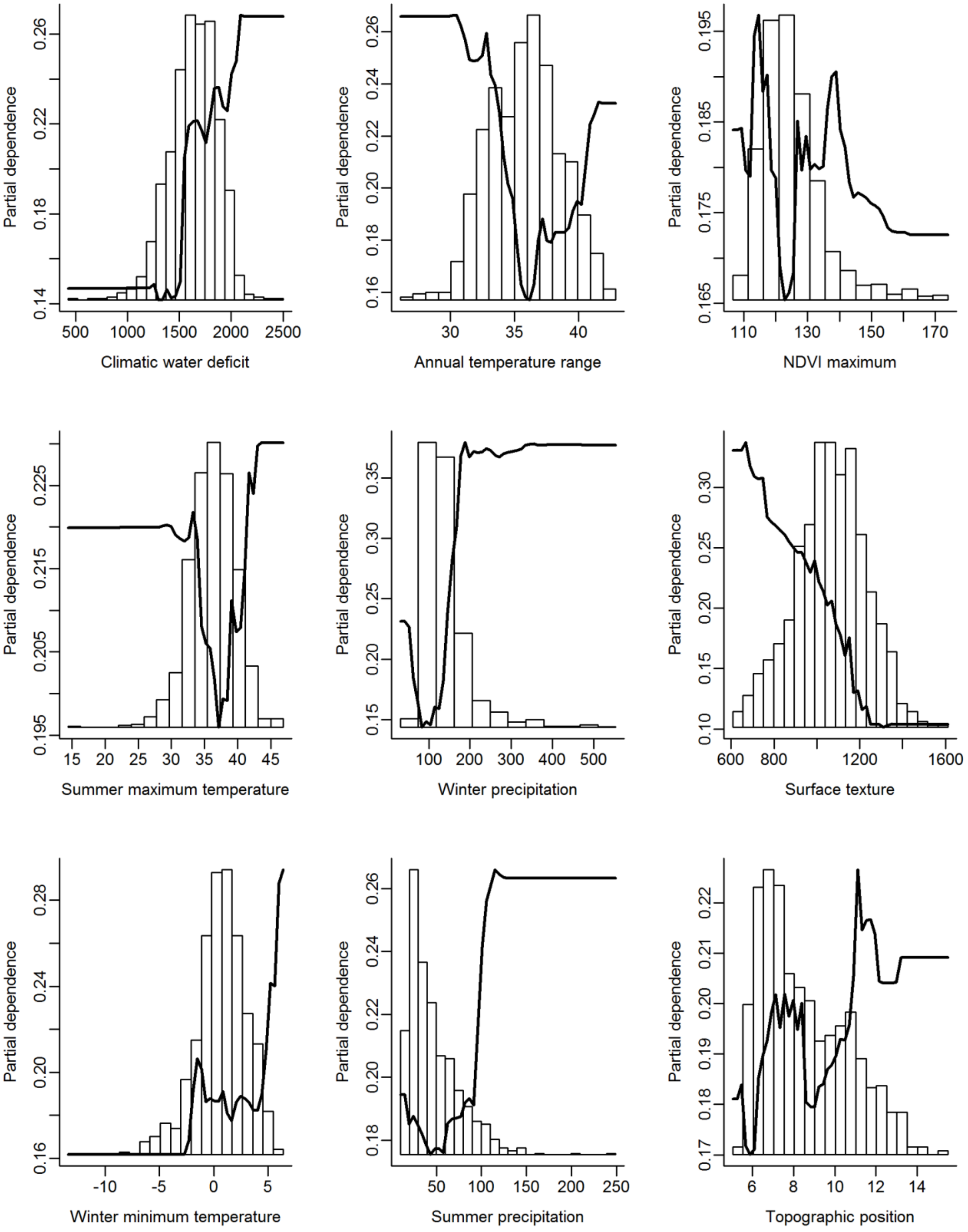

Random Forest response curves for *Chilopsis linearis*

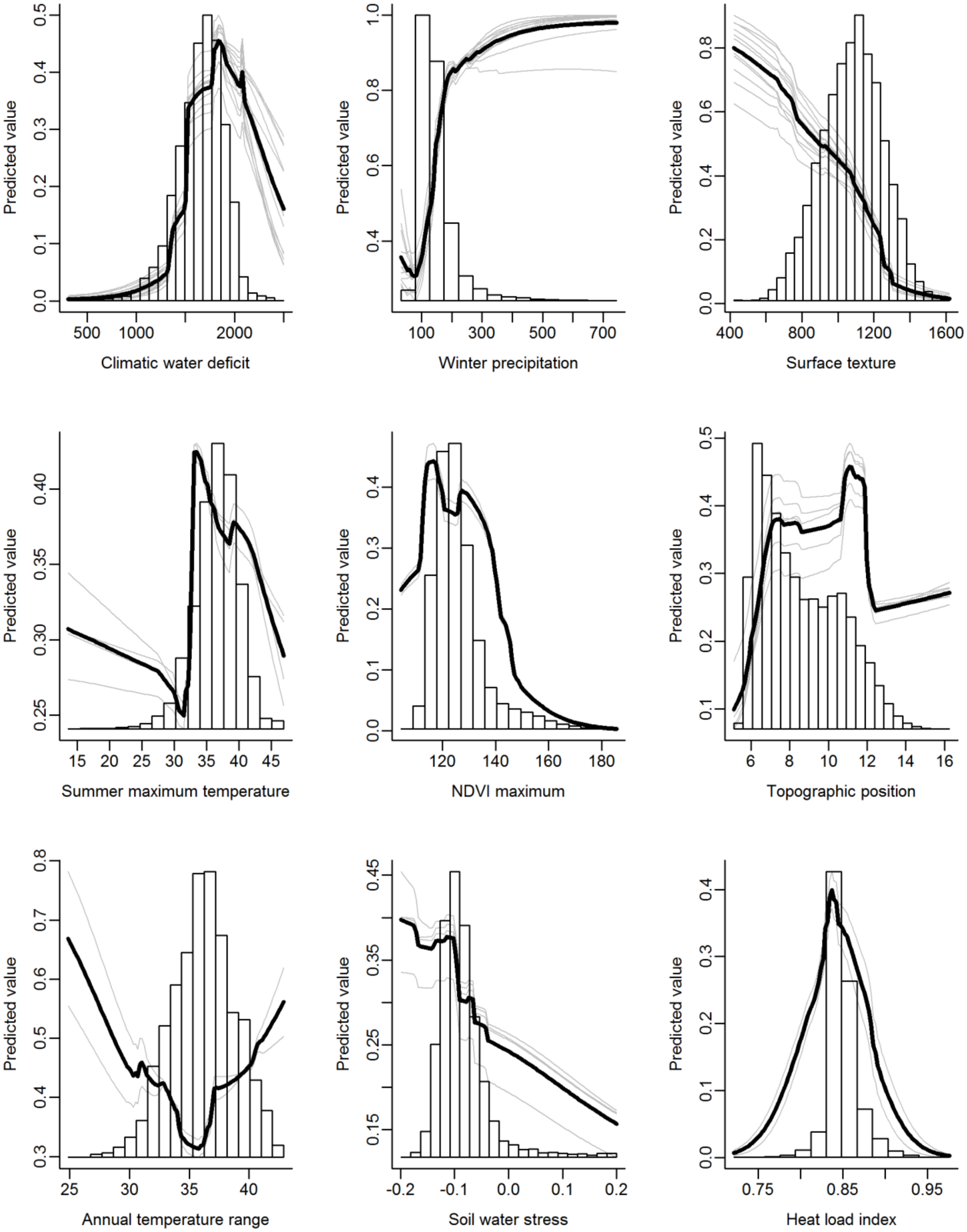

MaxEnt response curves for *Chilopsis linearis*

14. *Cryptantha micrantha* SDM and response curves (GAM, Random Forest, Maxent)

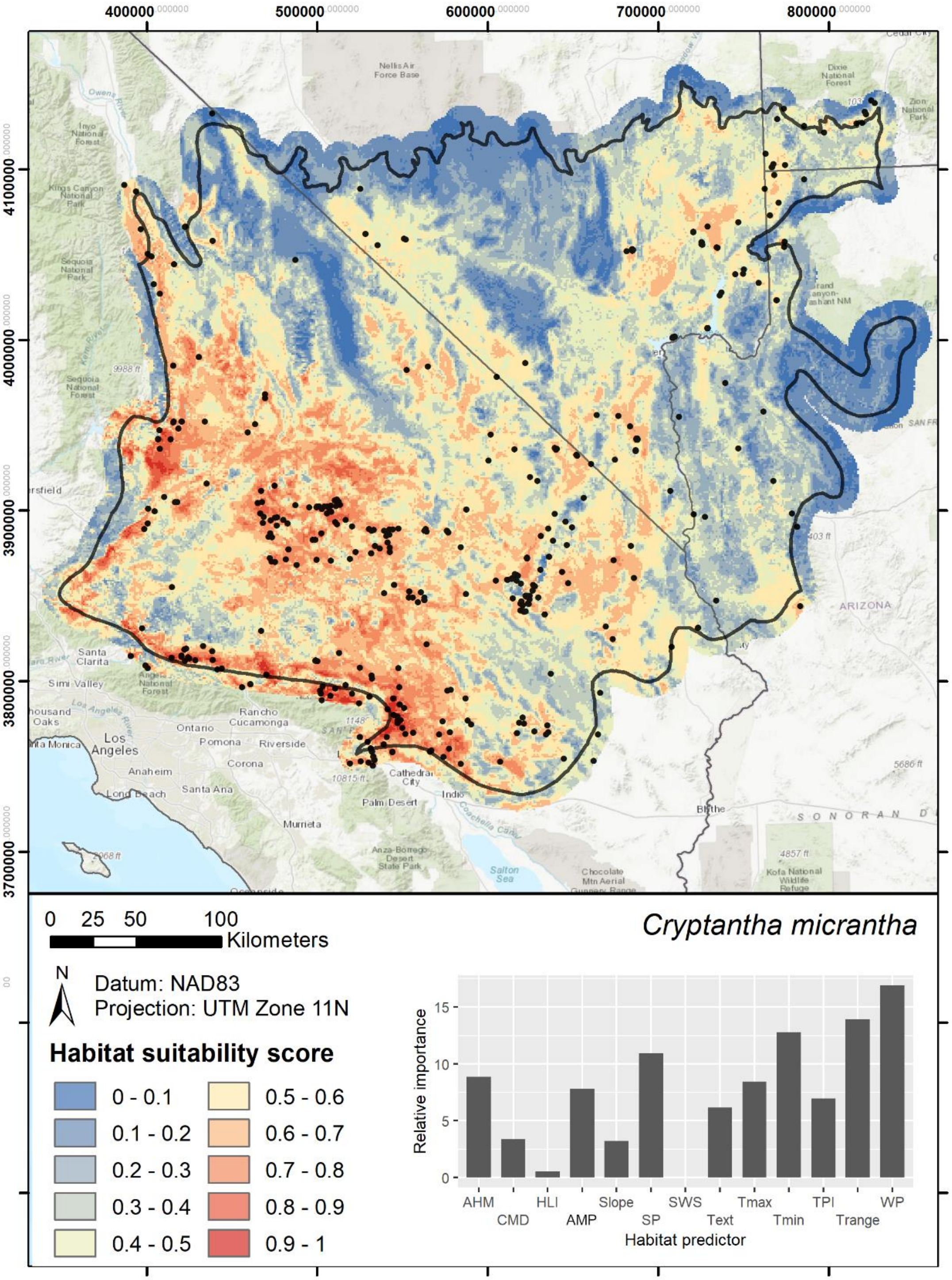

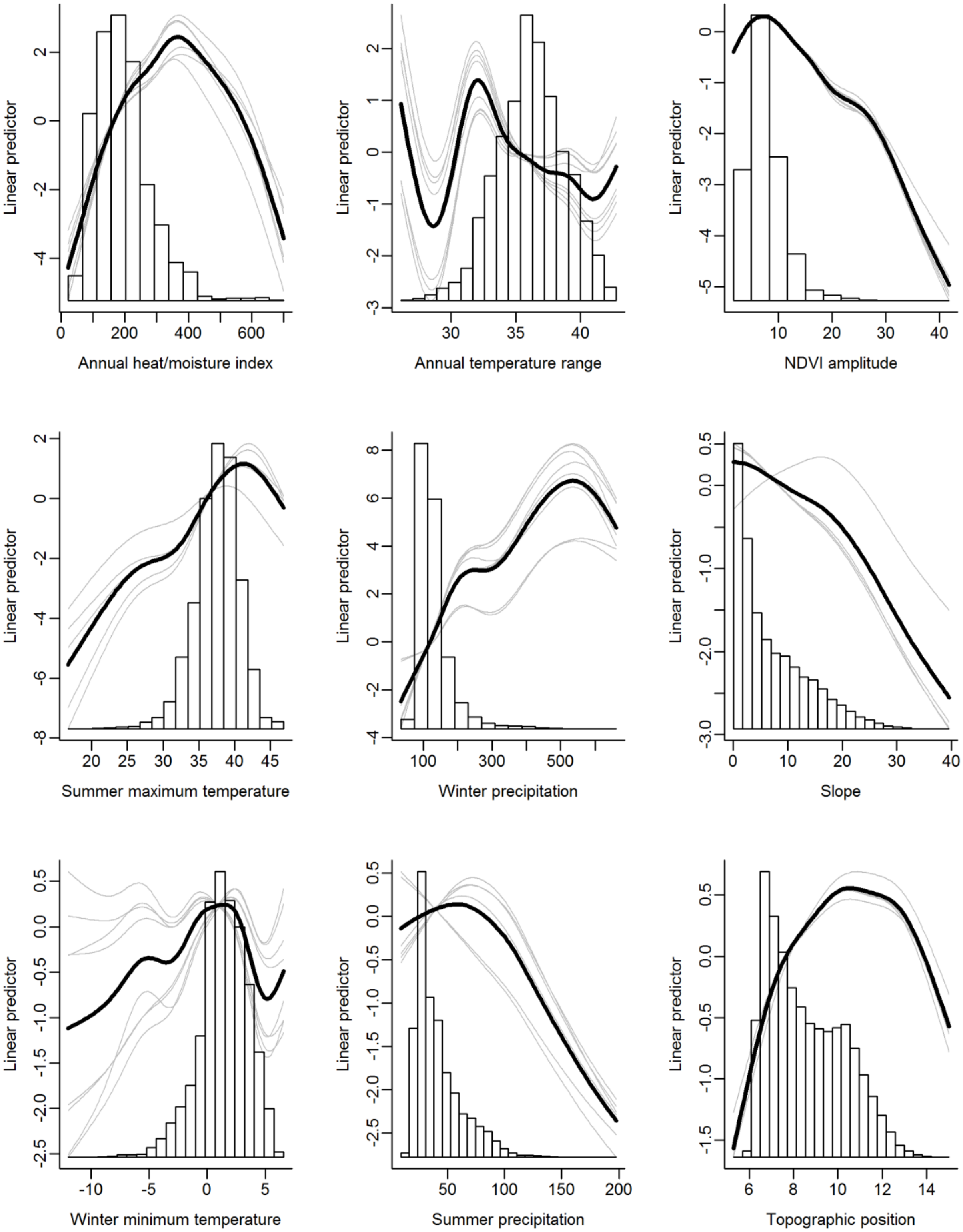

GAM response curves for *Cryptantha micrantha*

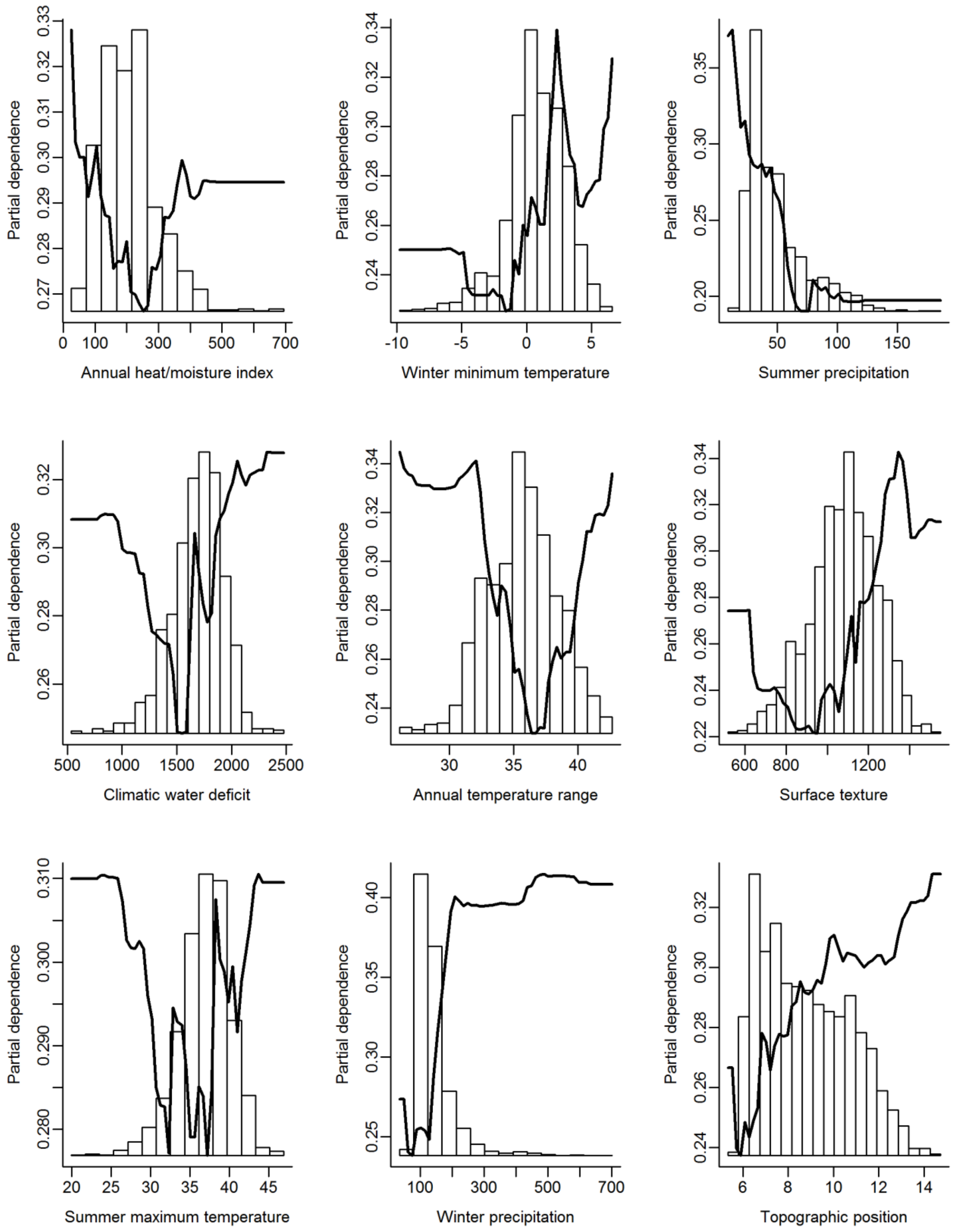

Random Forest response curves for *Cryptantha micrantha*

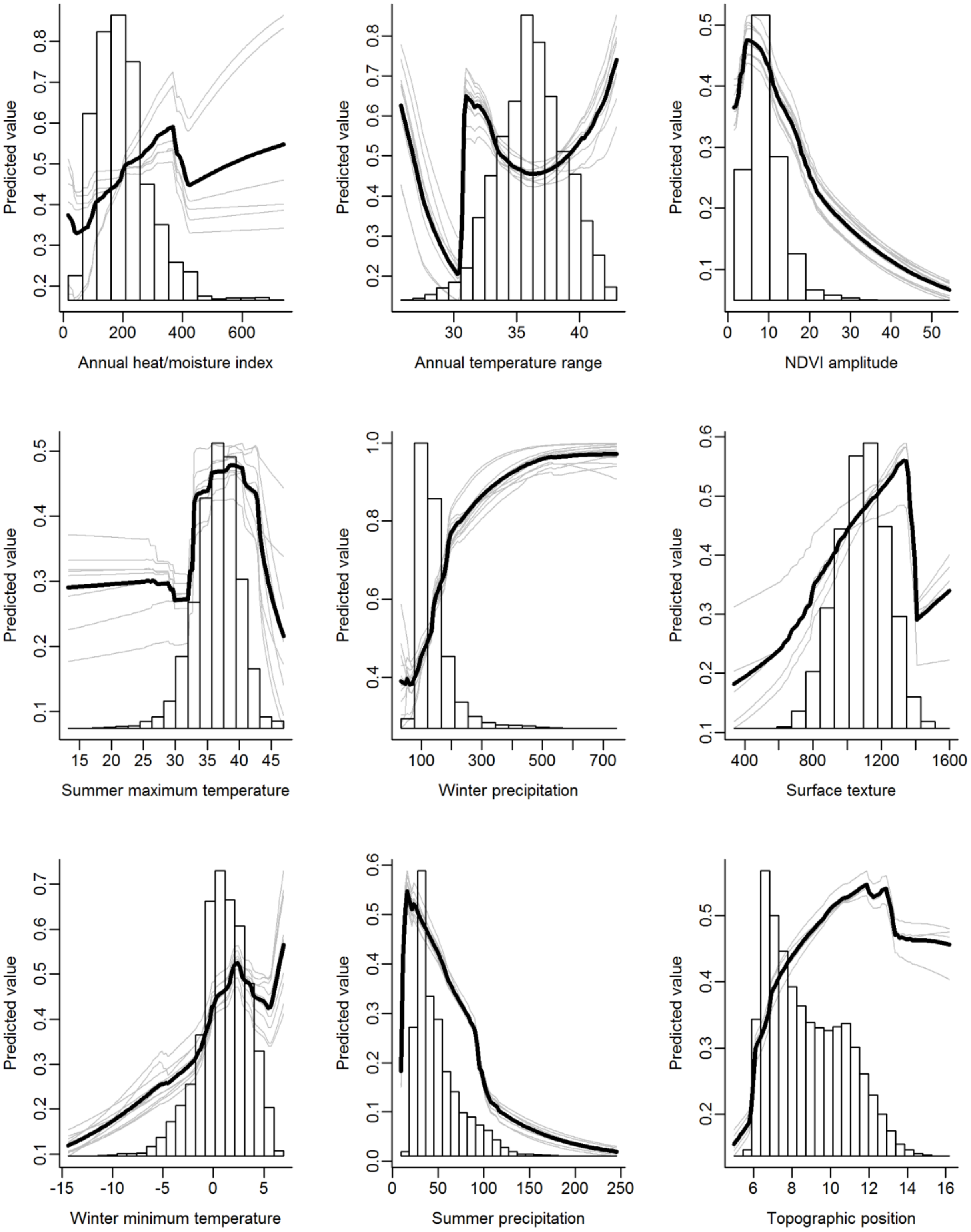

MaxEnt response curves for *Cryptantha micrantha*

15. *Cryptantha nevadensis* SDM and response curves (GAM, Random Forest, Maxent)

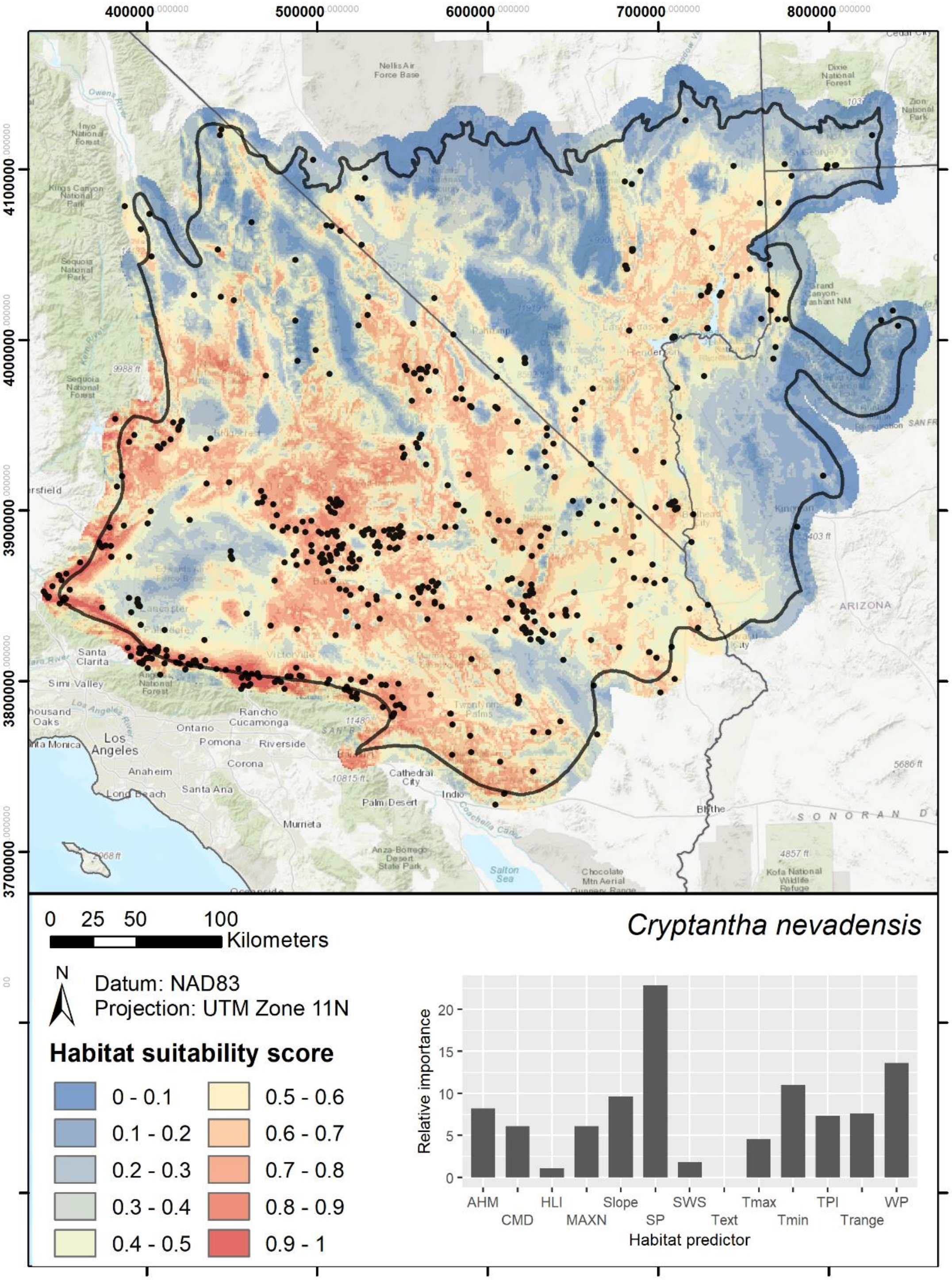

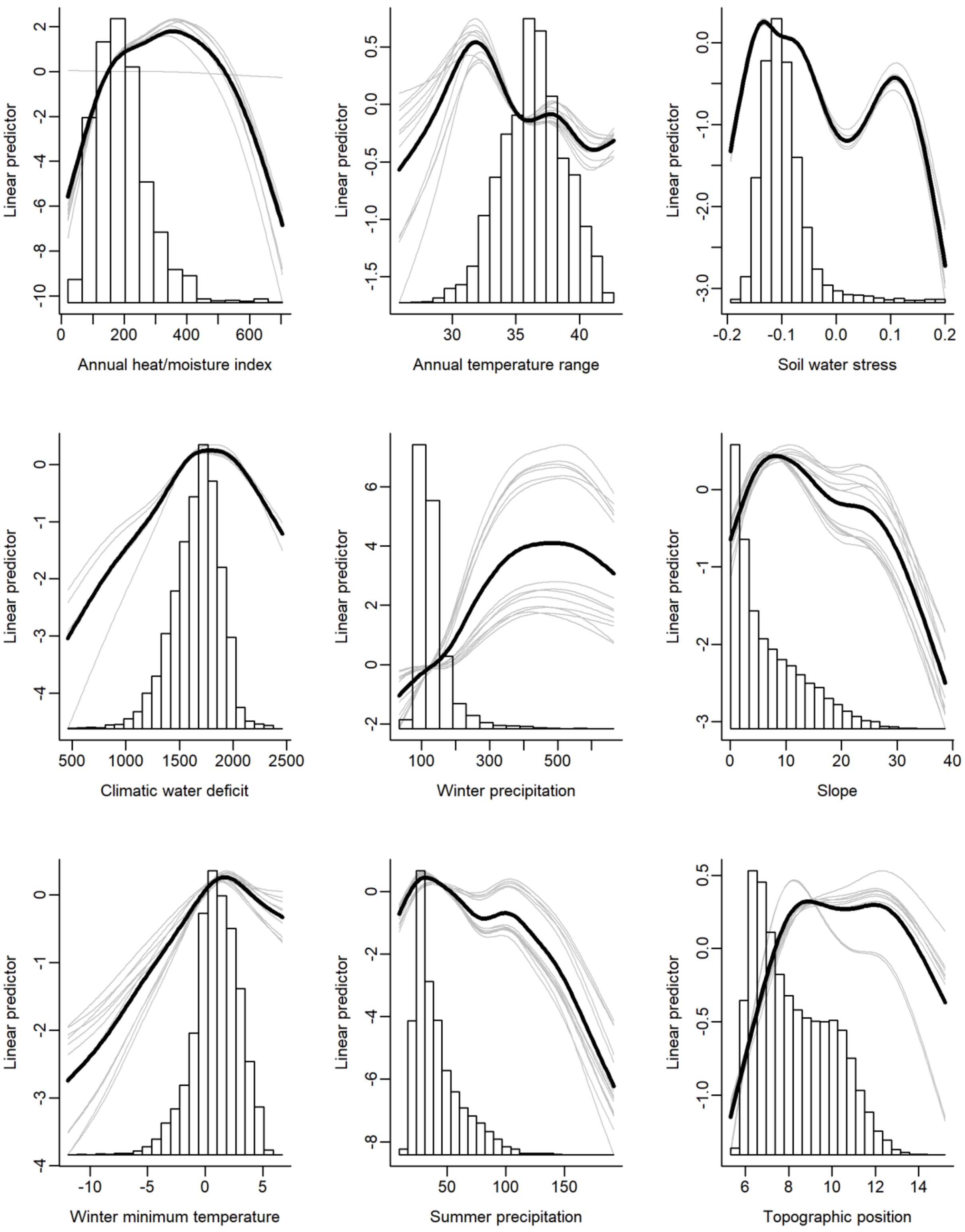

GAM response curves for *Cryptantha nevadensis*

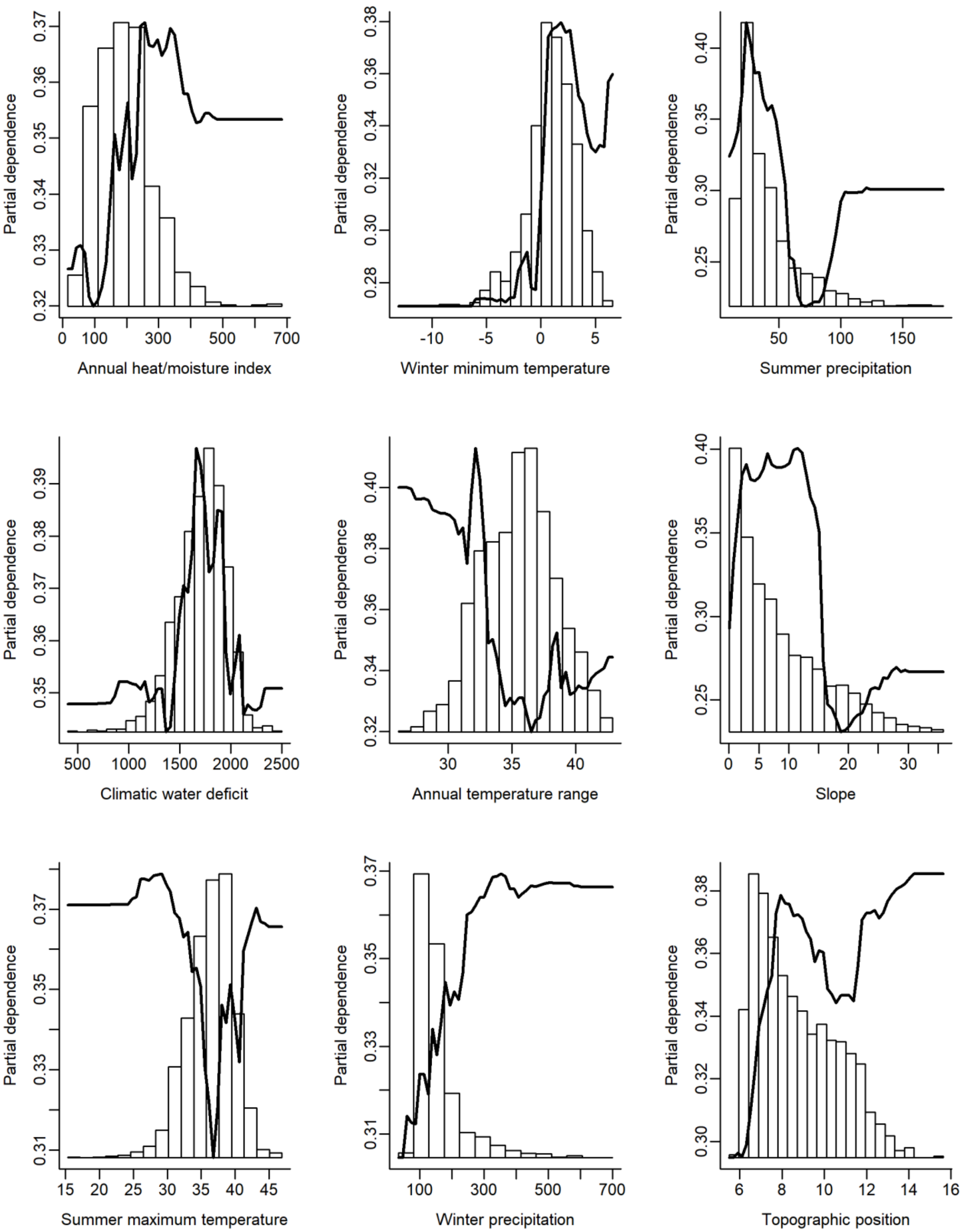

Random Forest response curves for *Cryptantha nevadensis*

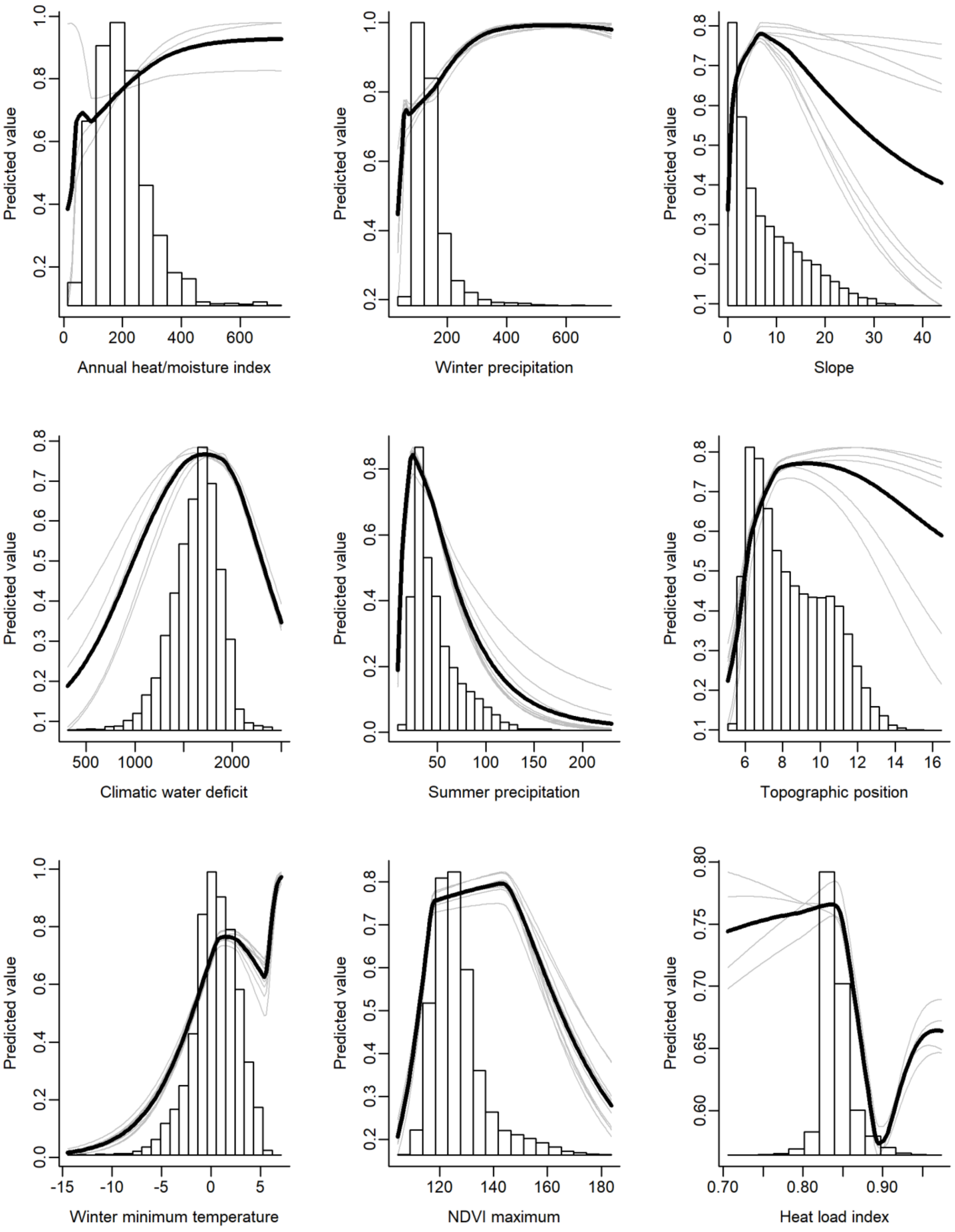

MaxEnt response curves for *Cryptantha nevadensis*

16. *Descurainia pinnata* SDM and response curves (GAM, Random Forest, Maxent)

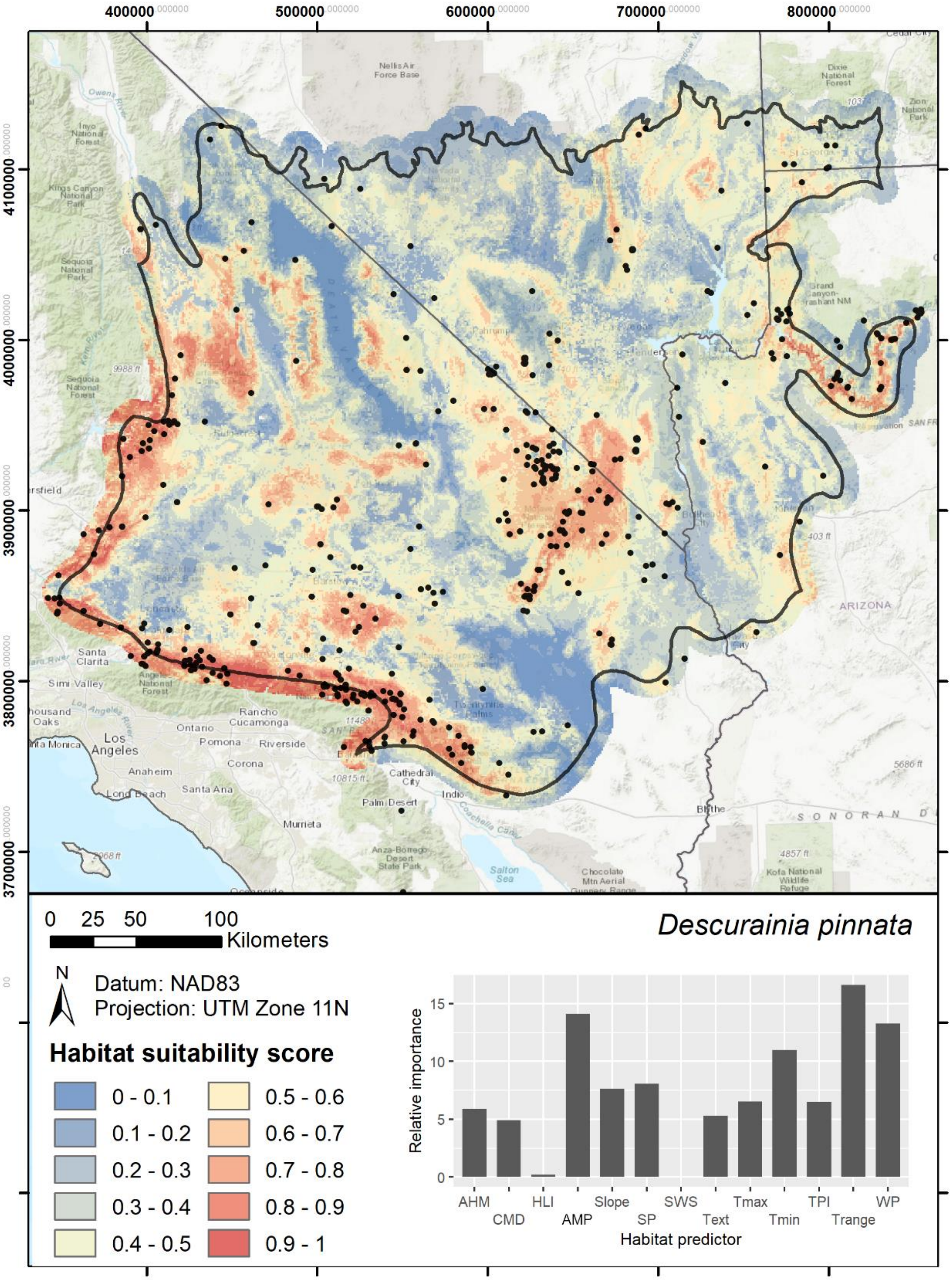

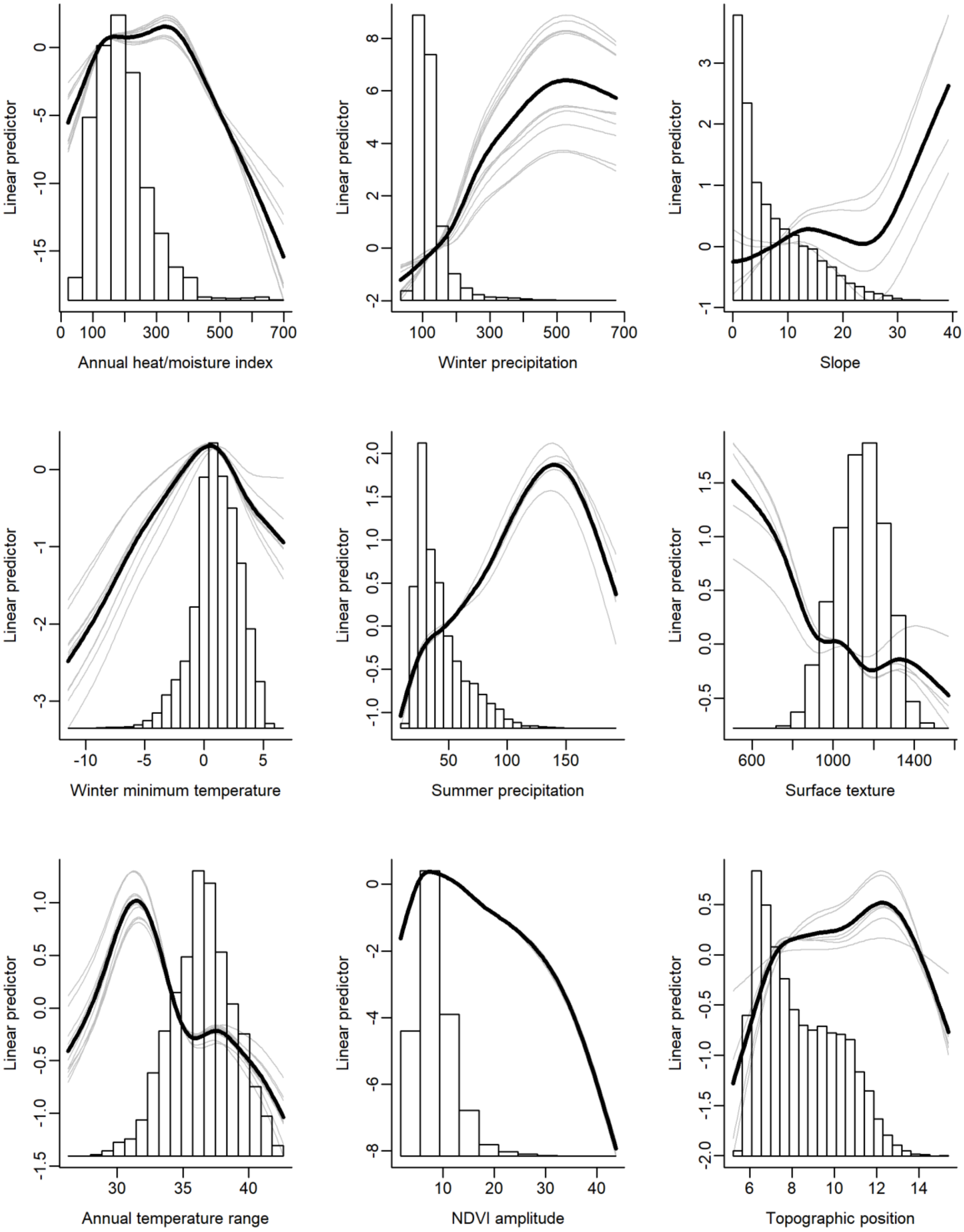

GAM response curves for *Descurainia pinnata*

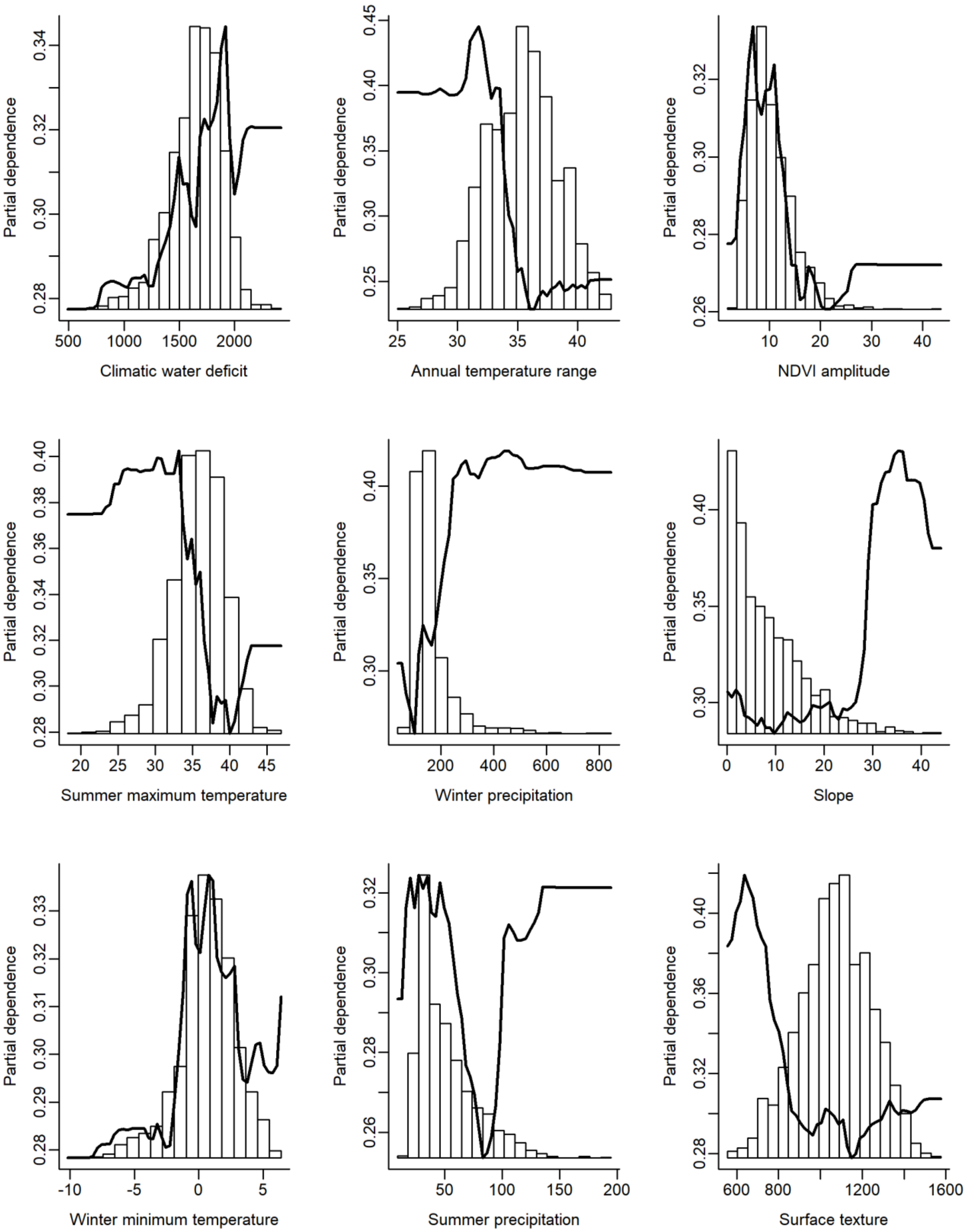

Random Forest response curves for *Descurainia pinnata*

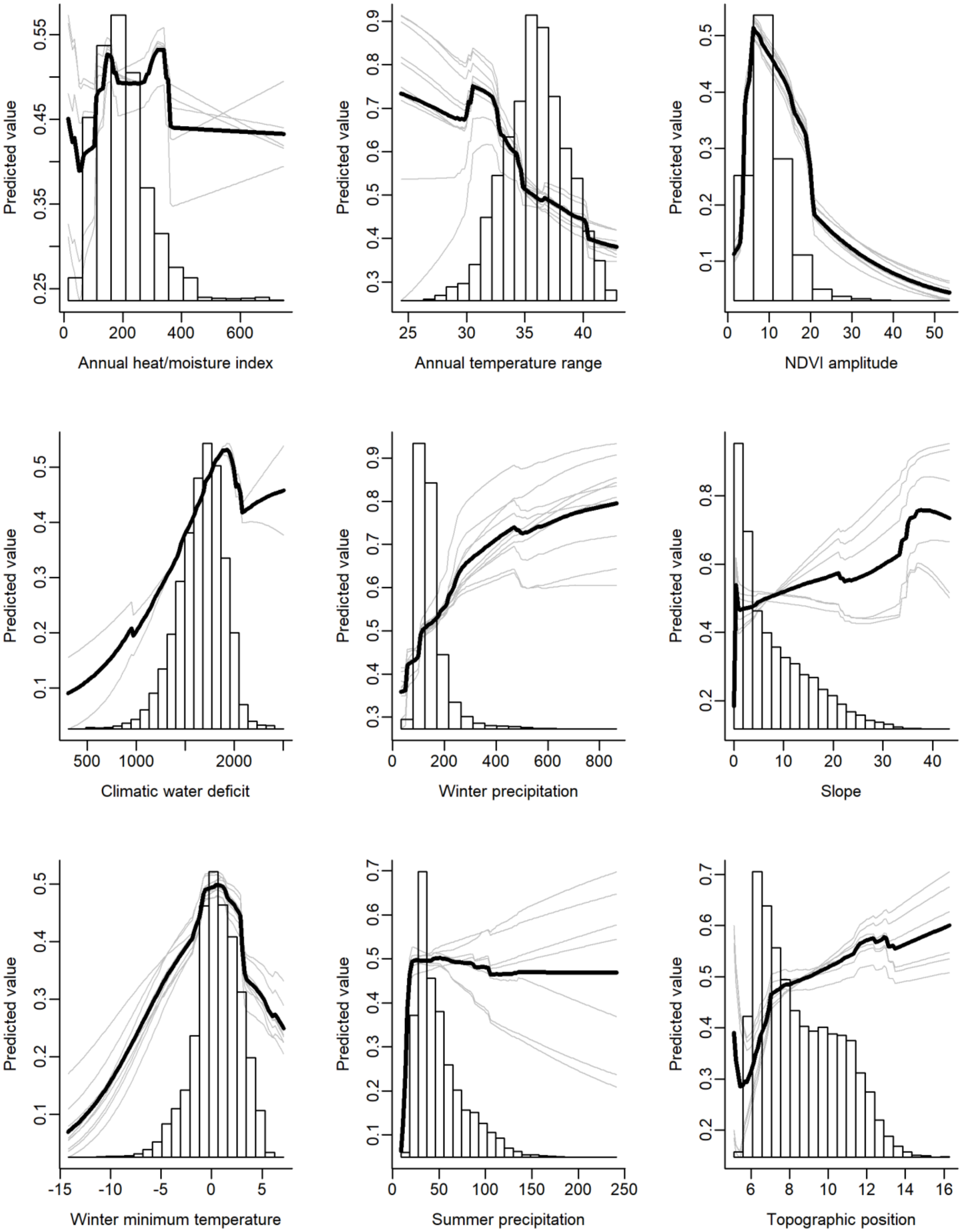

MaxEnt response curves for *Descurainia pinnata*

17. *Echinocereus engelmannii* SDM and response curves (GAM, Random Forest, Maxent)

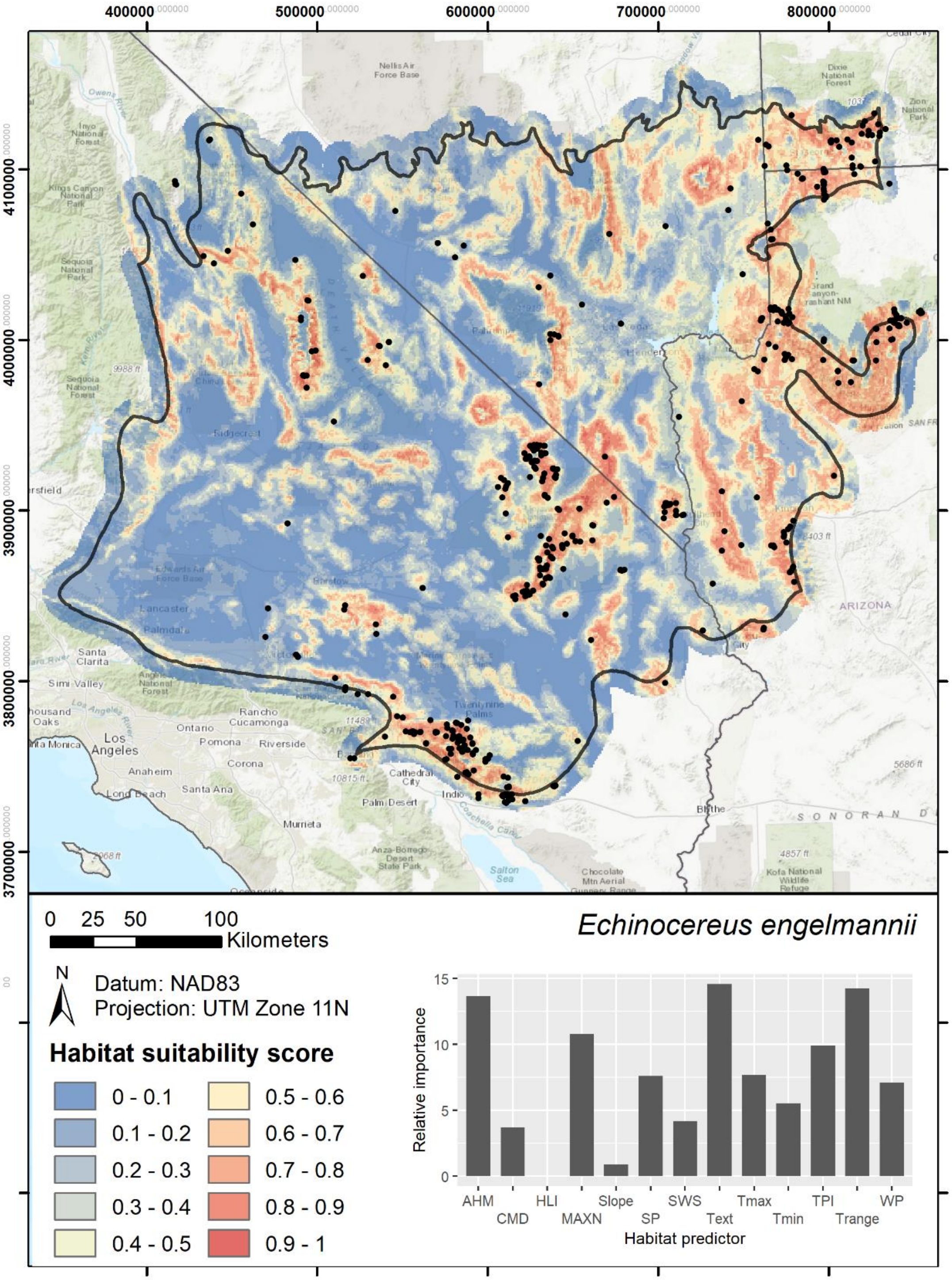

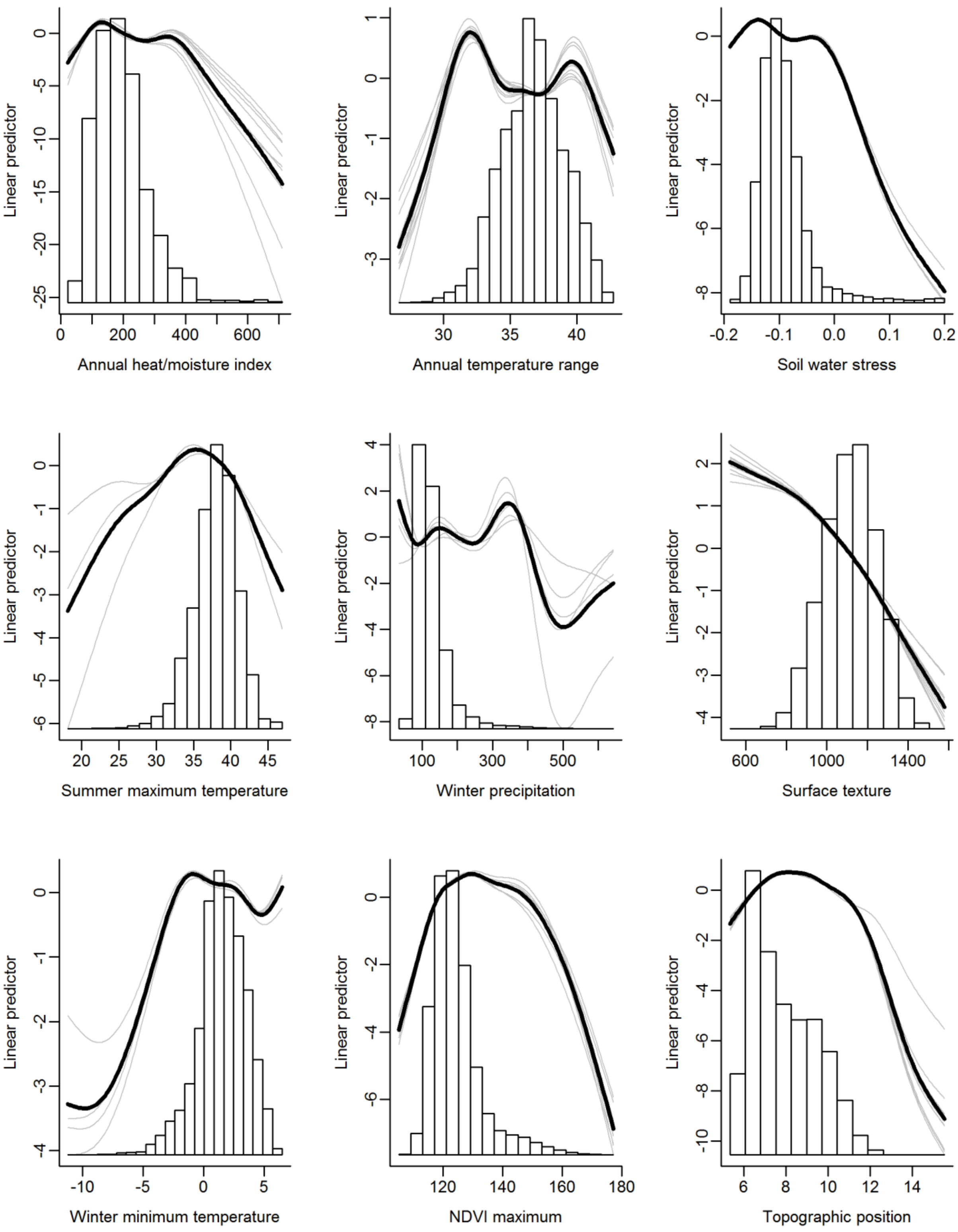

GAM response curves for *Echinocereus engelmannii*

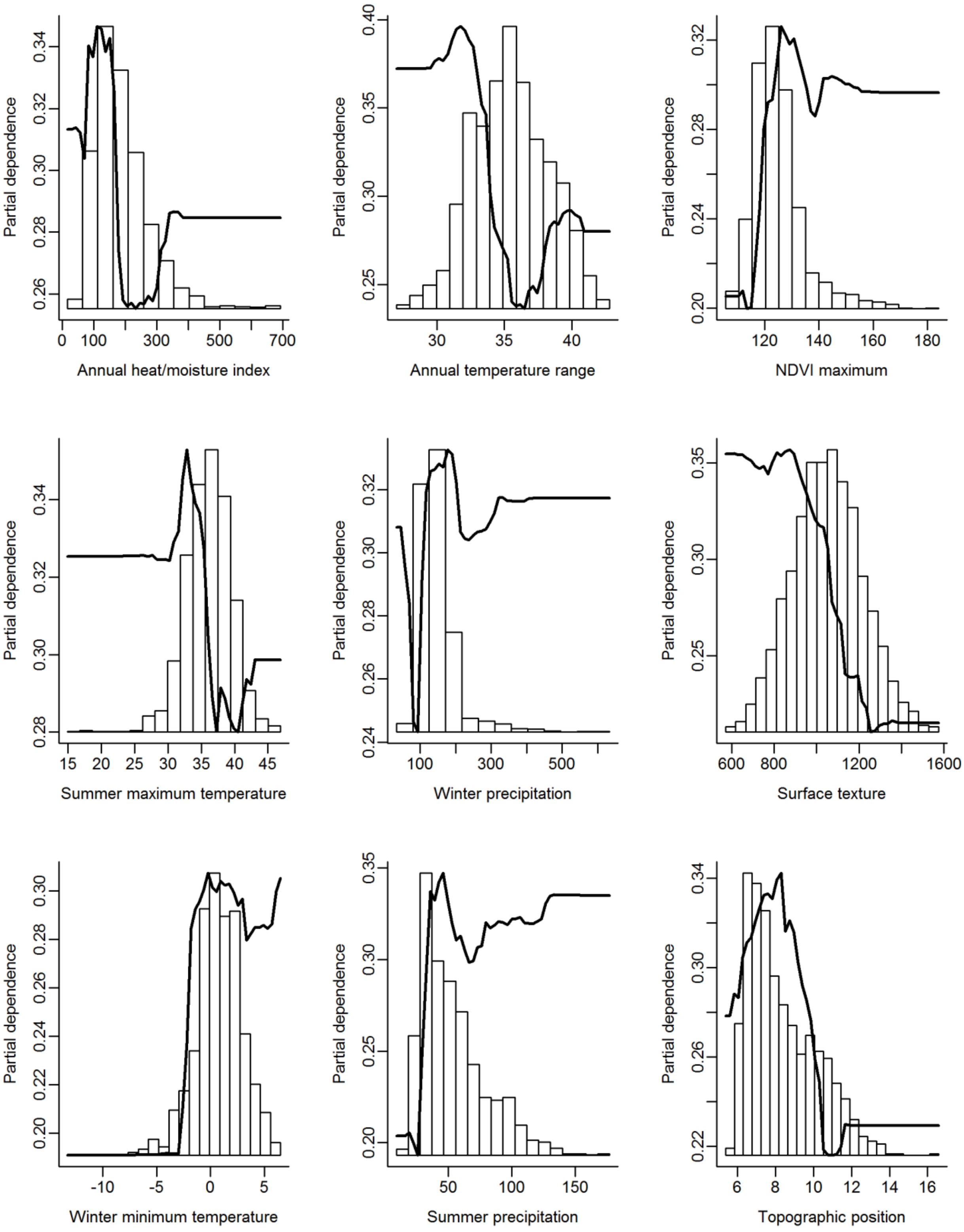

Random Forest response curves for *Echinocereus engelmannii*

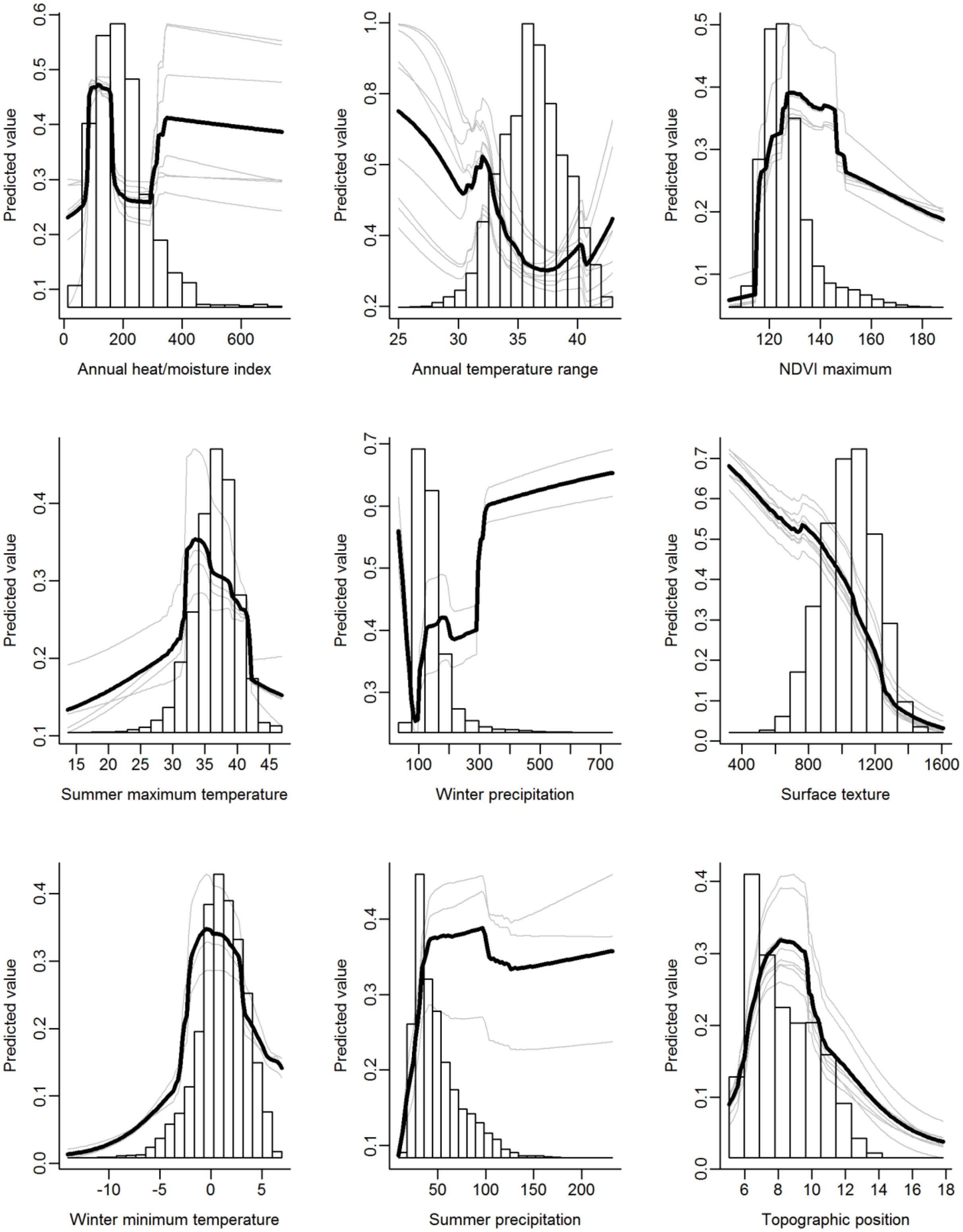

MaxEnt response curves for *Echinocereus engelmannii*

18. *Encelia farinosa* SDM and response curves (GAM, Random Forest, Maxent)

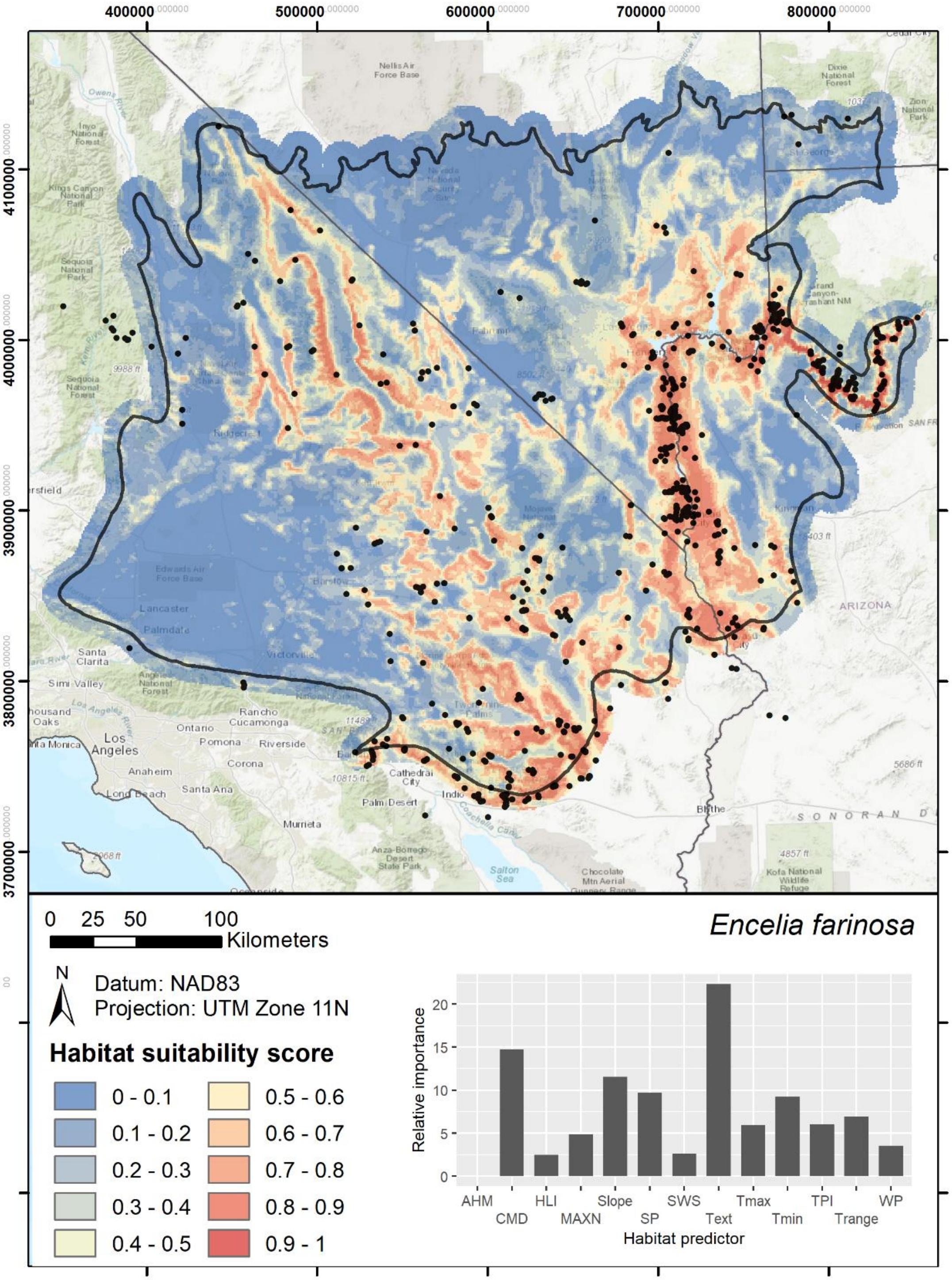

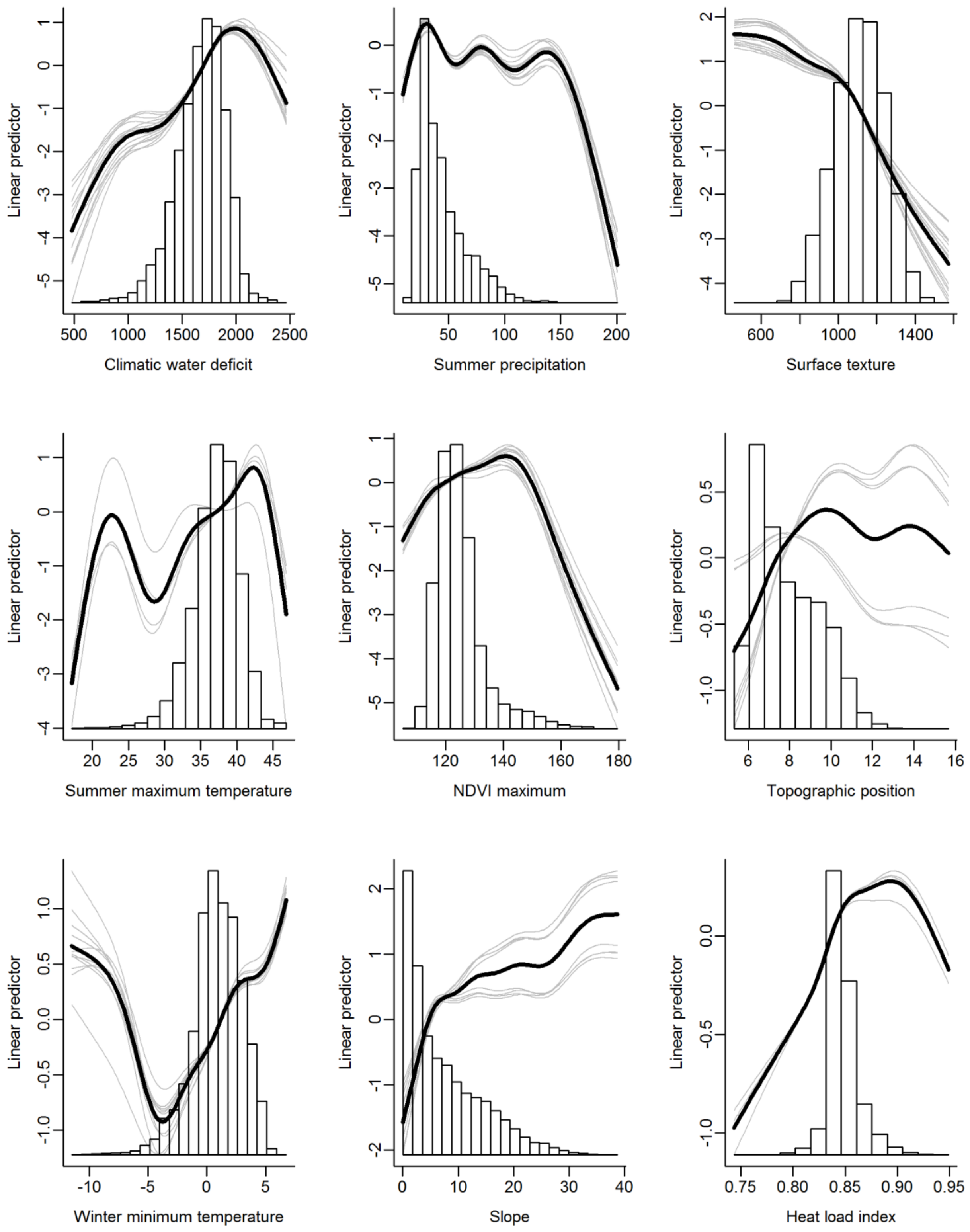

GAM response curves for *Encelia farinosa*

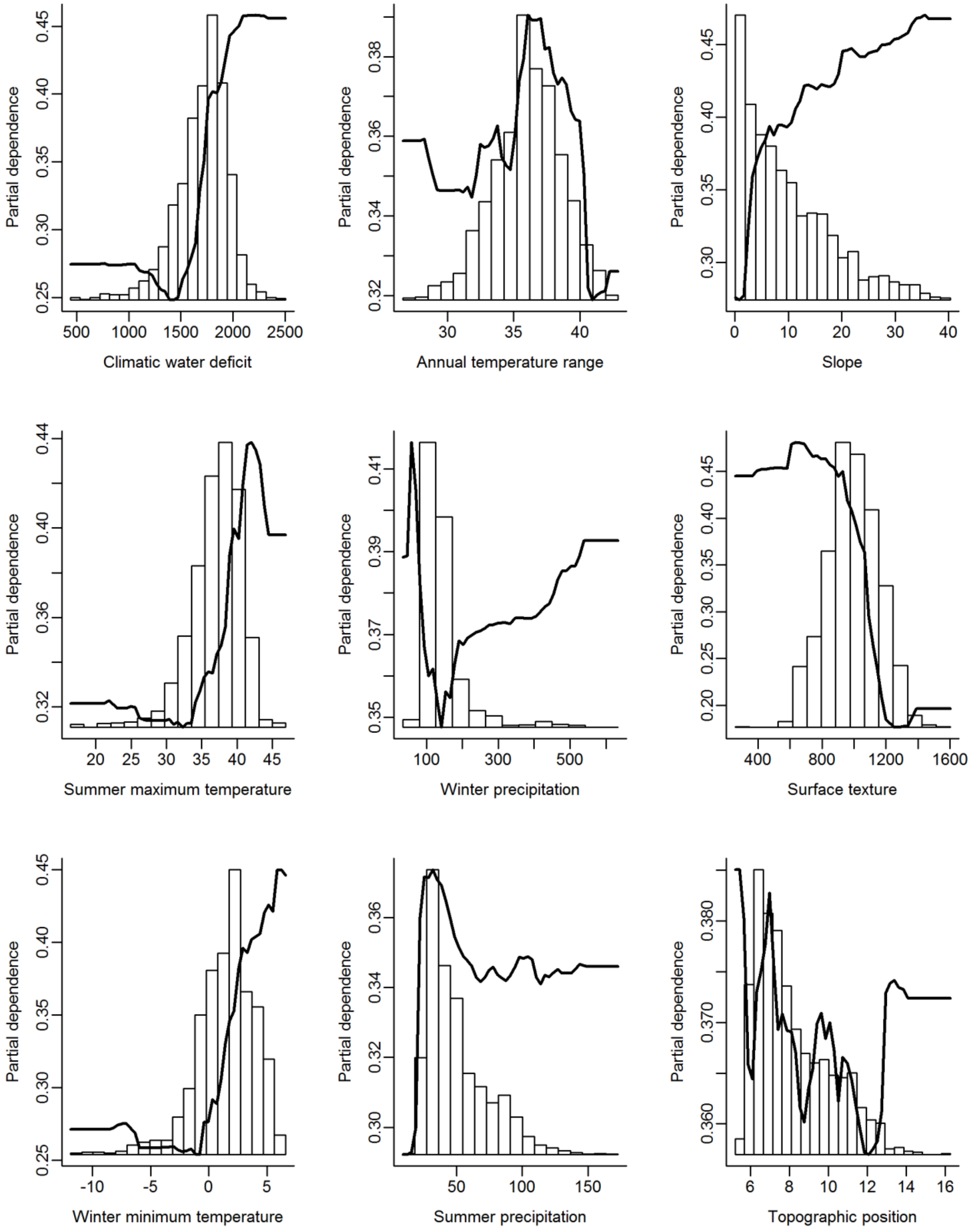

Random Forest response curves for *Encelia farinosa*

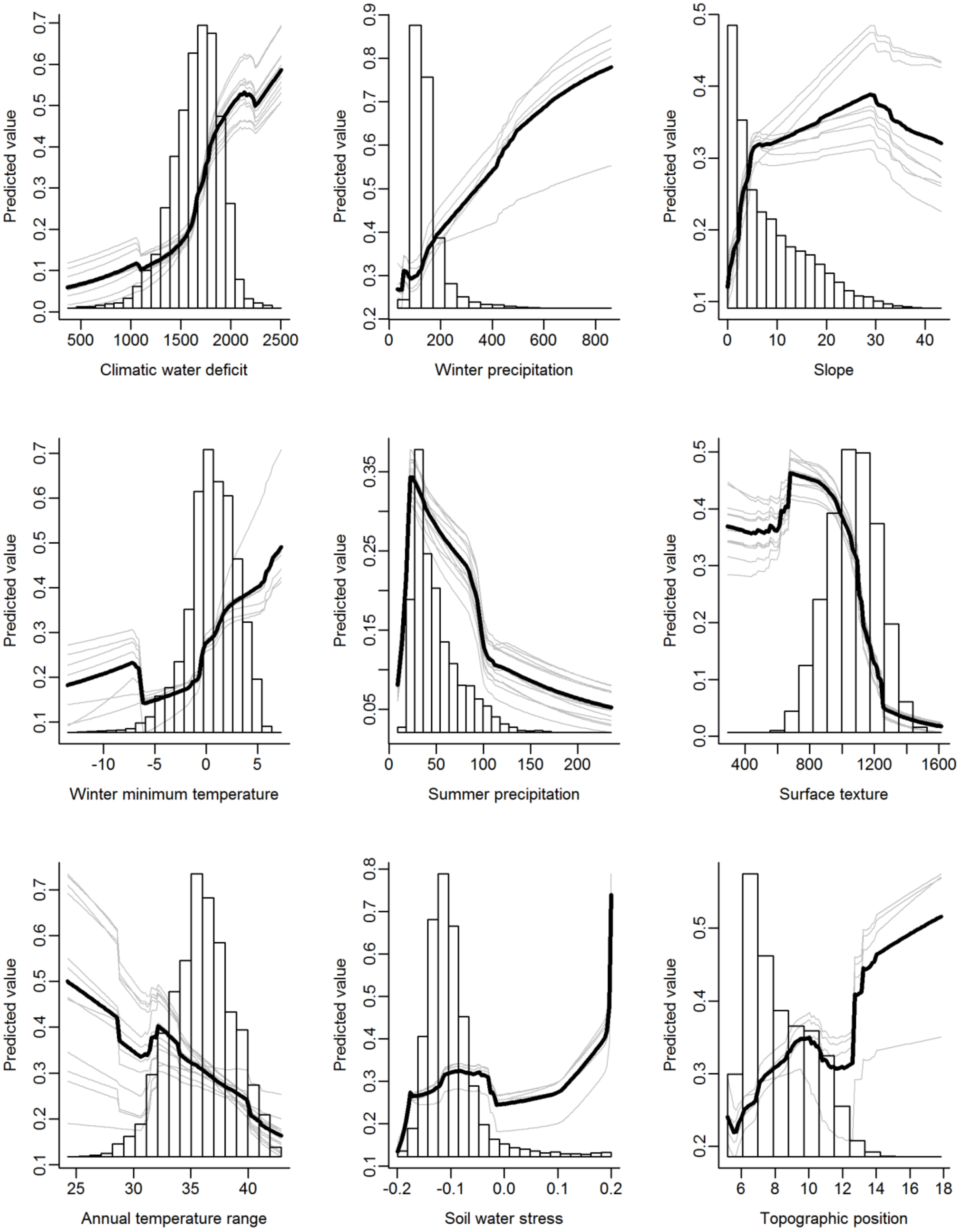

MaxEnt response curves for *Encelia farinosa*

19. *Ephedra nevadensis* SDM and response curves (GAM, Random Forest, Maxent)

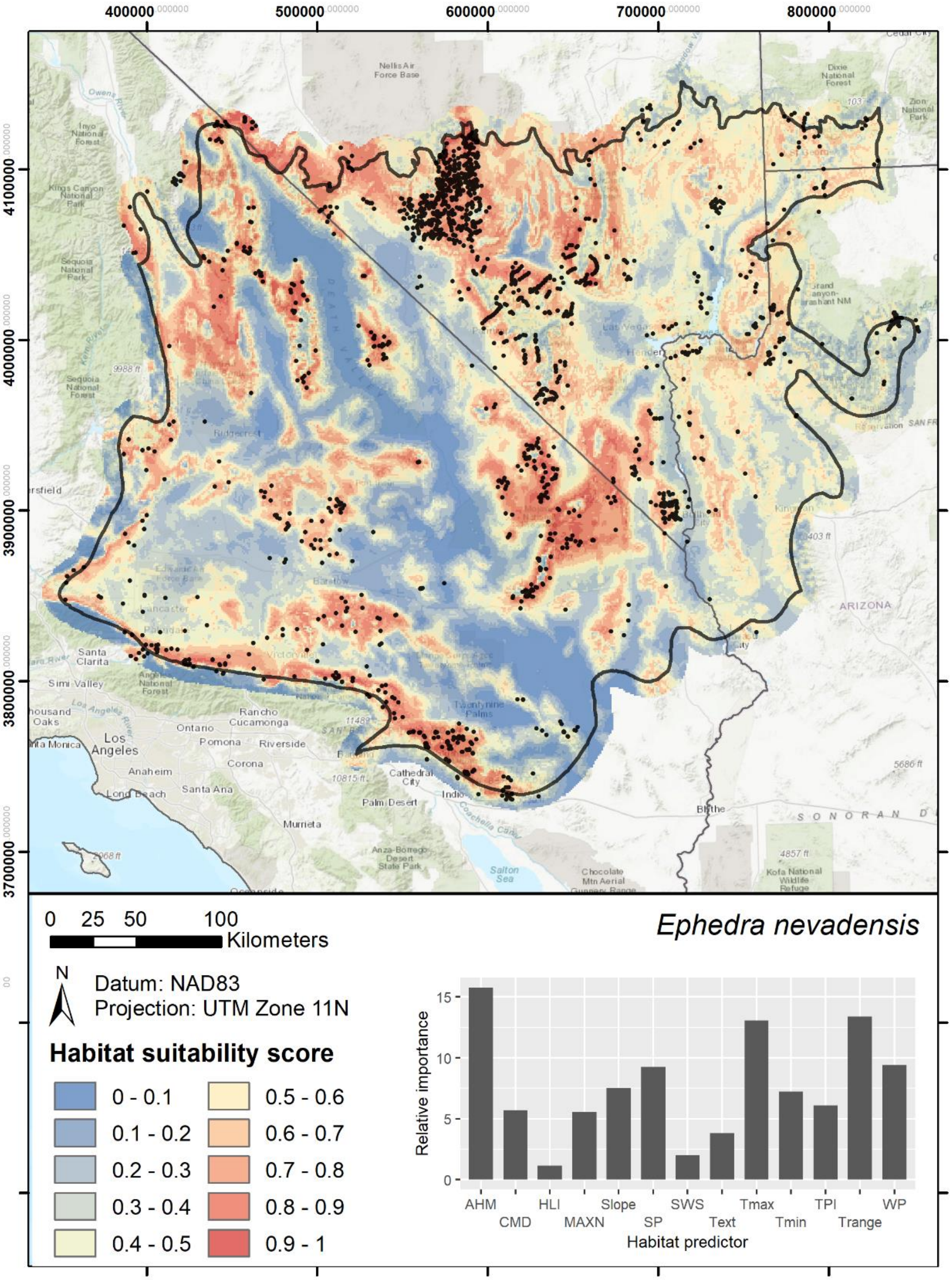

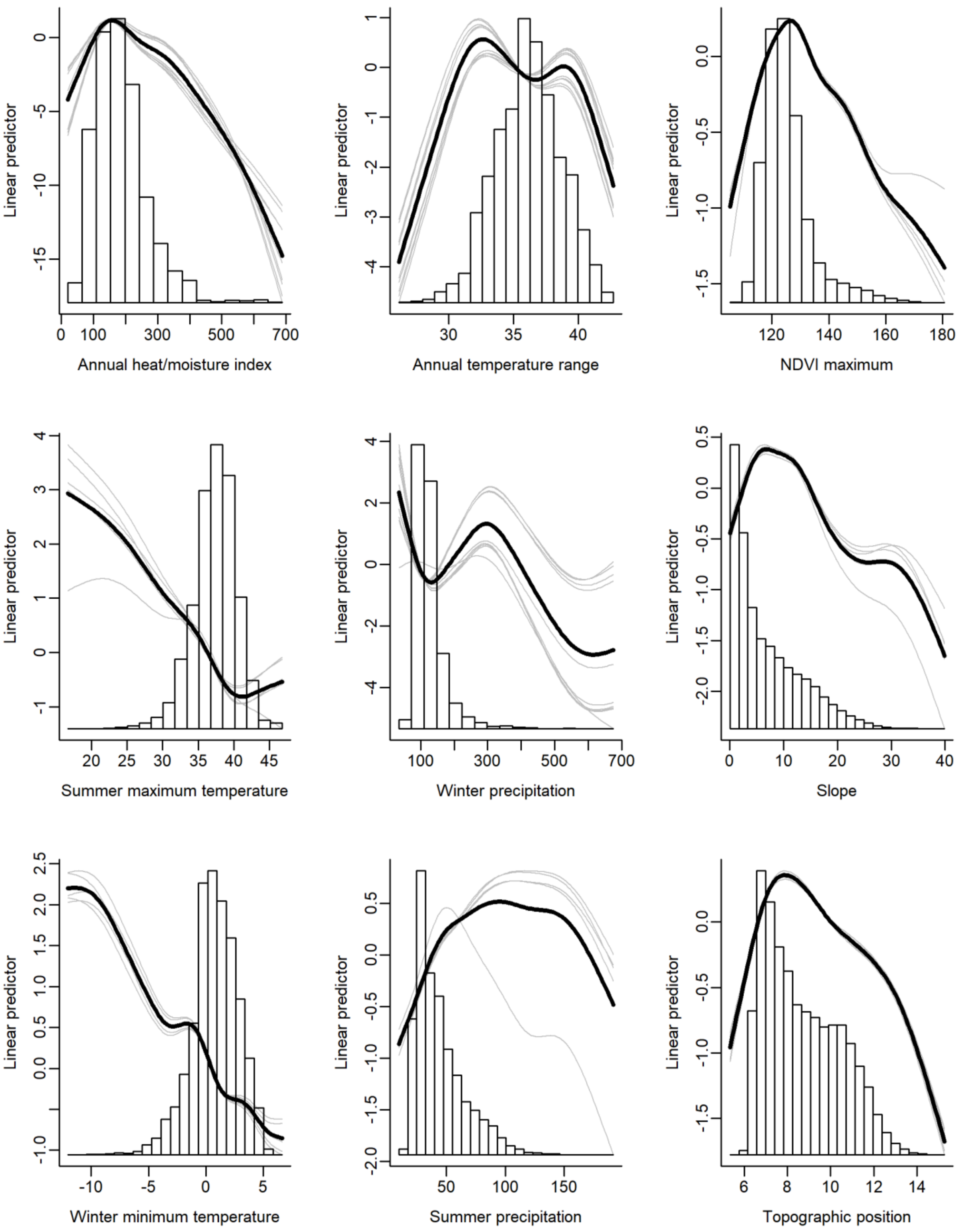

GAM response curves for *Ephedra nevadensis*

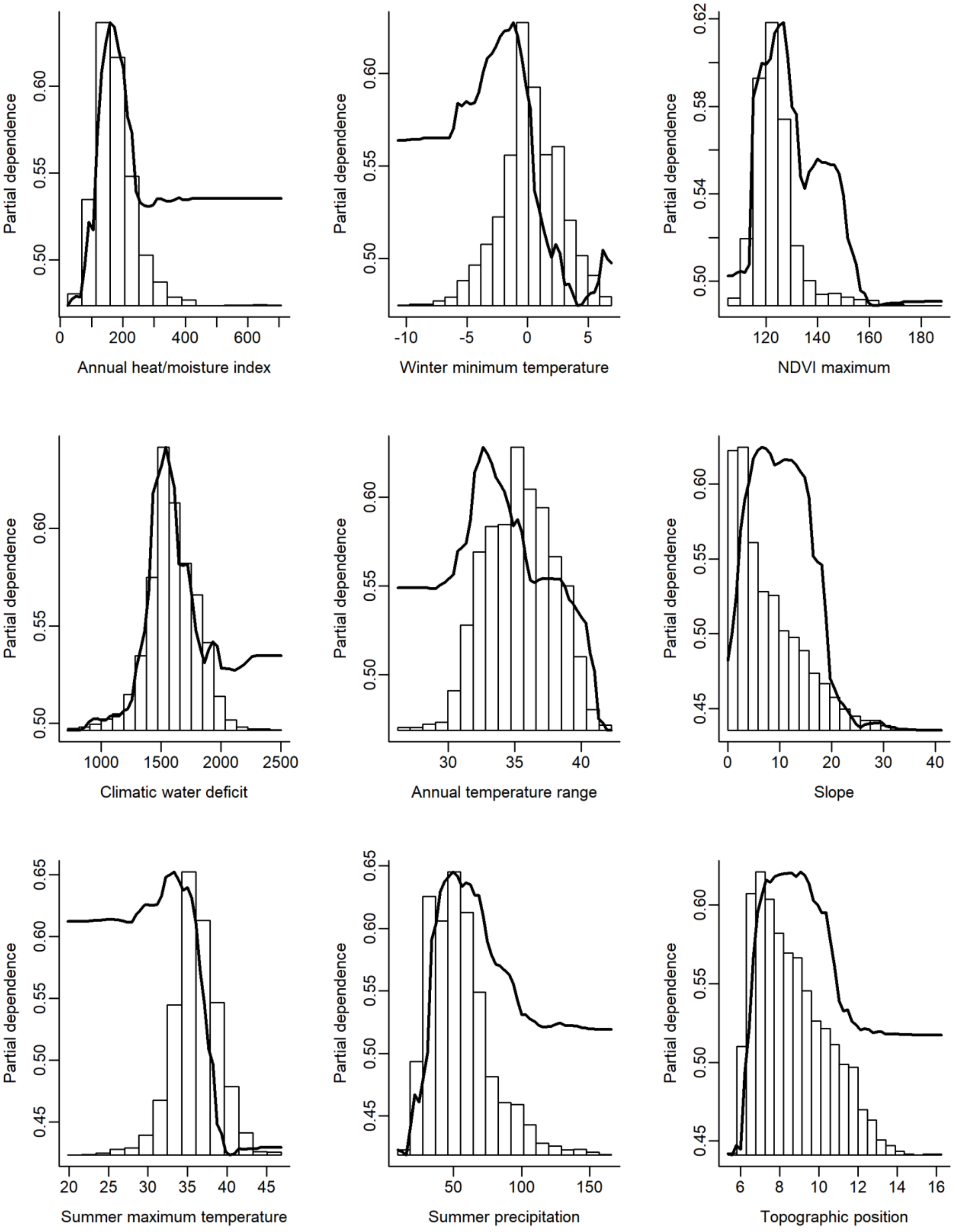

Random Forest response curves for *Ephedra nevadensis*

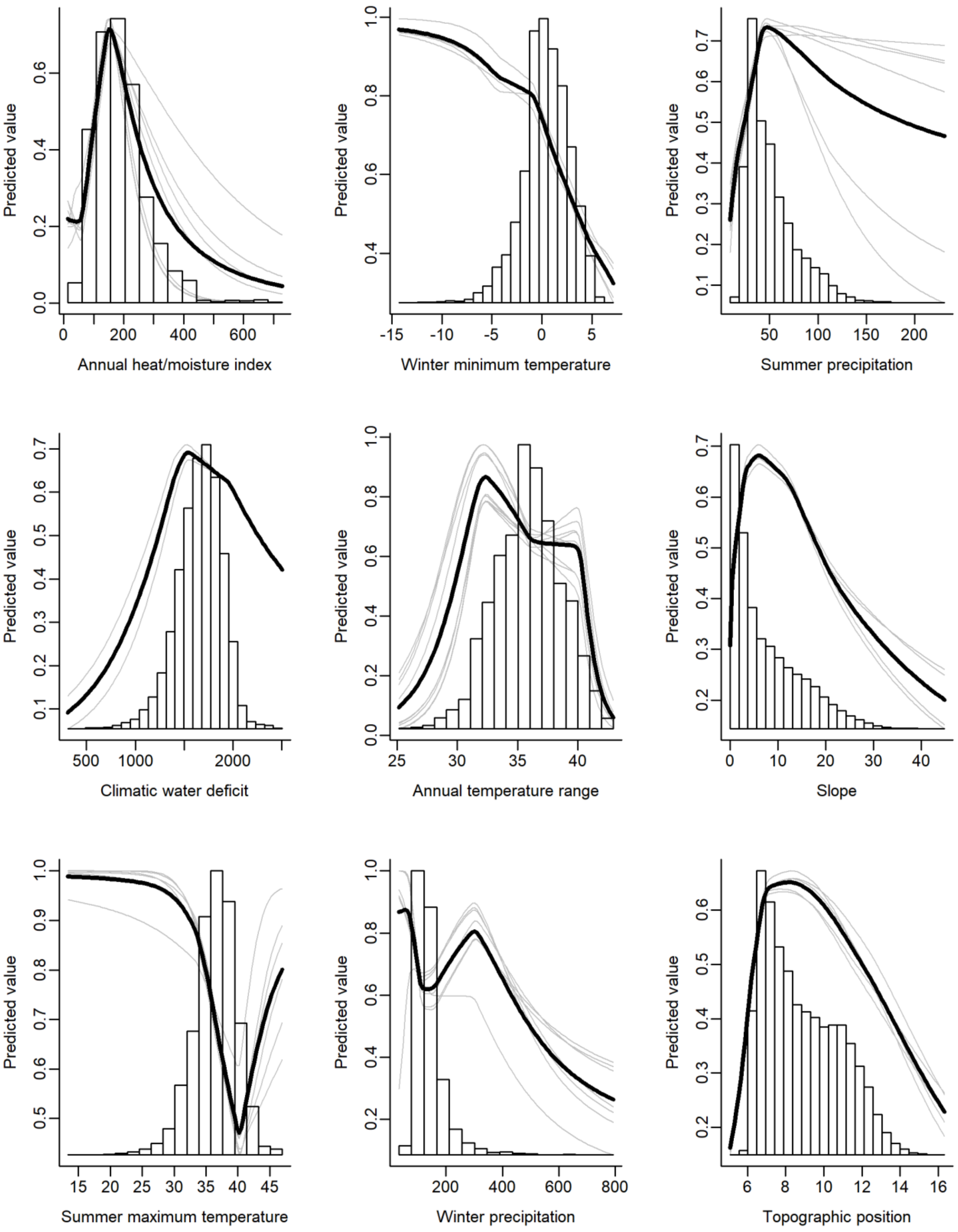

MaxEnt response curves for *Ephedra nevadensis*

20. *Ericameria cooperi* SDM and response curves (GAM, Random Forest, Maxent)

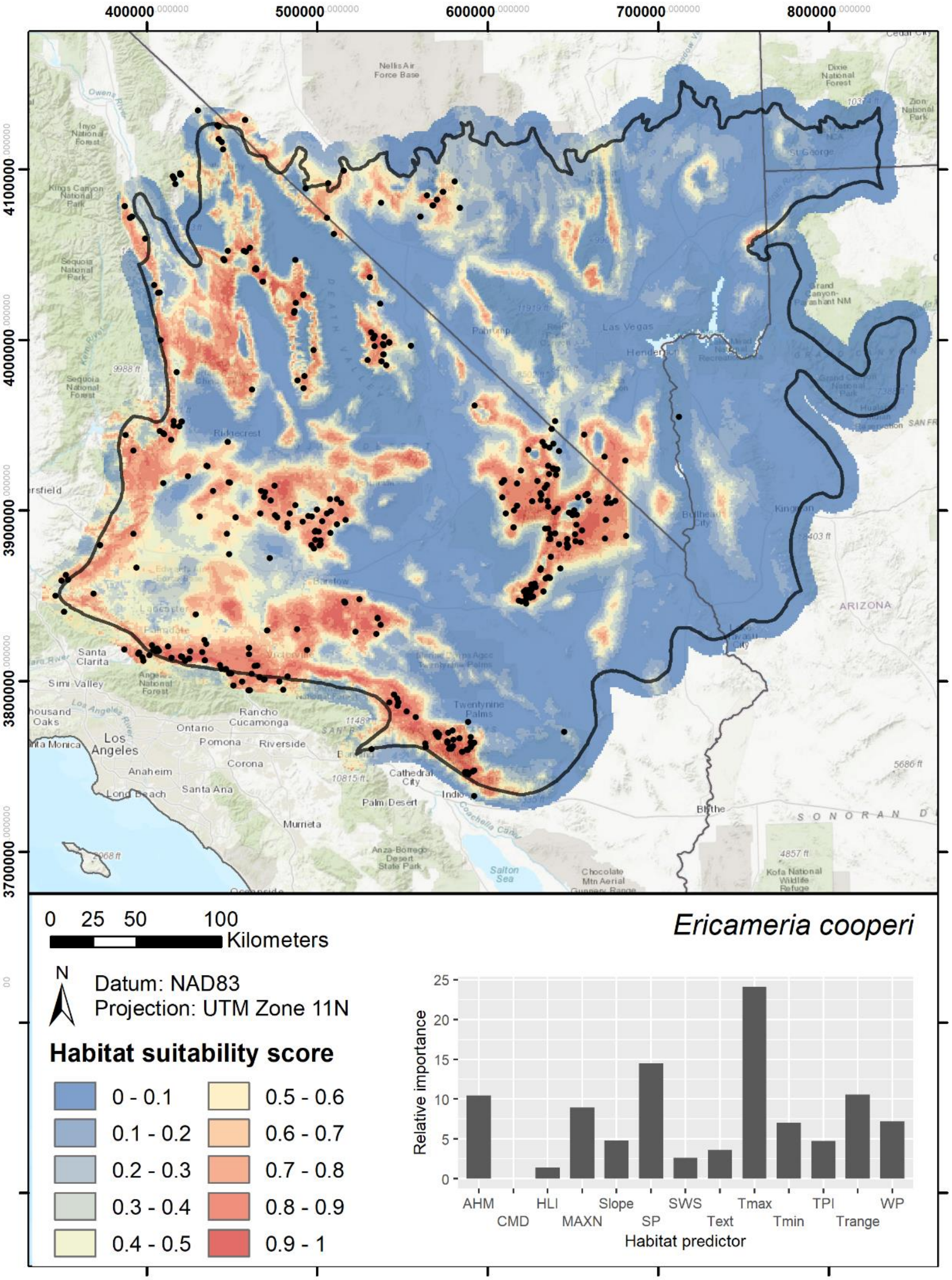

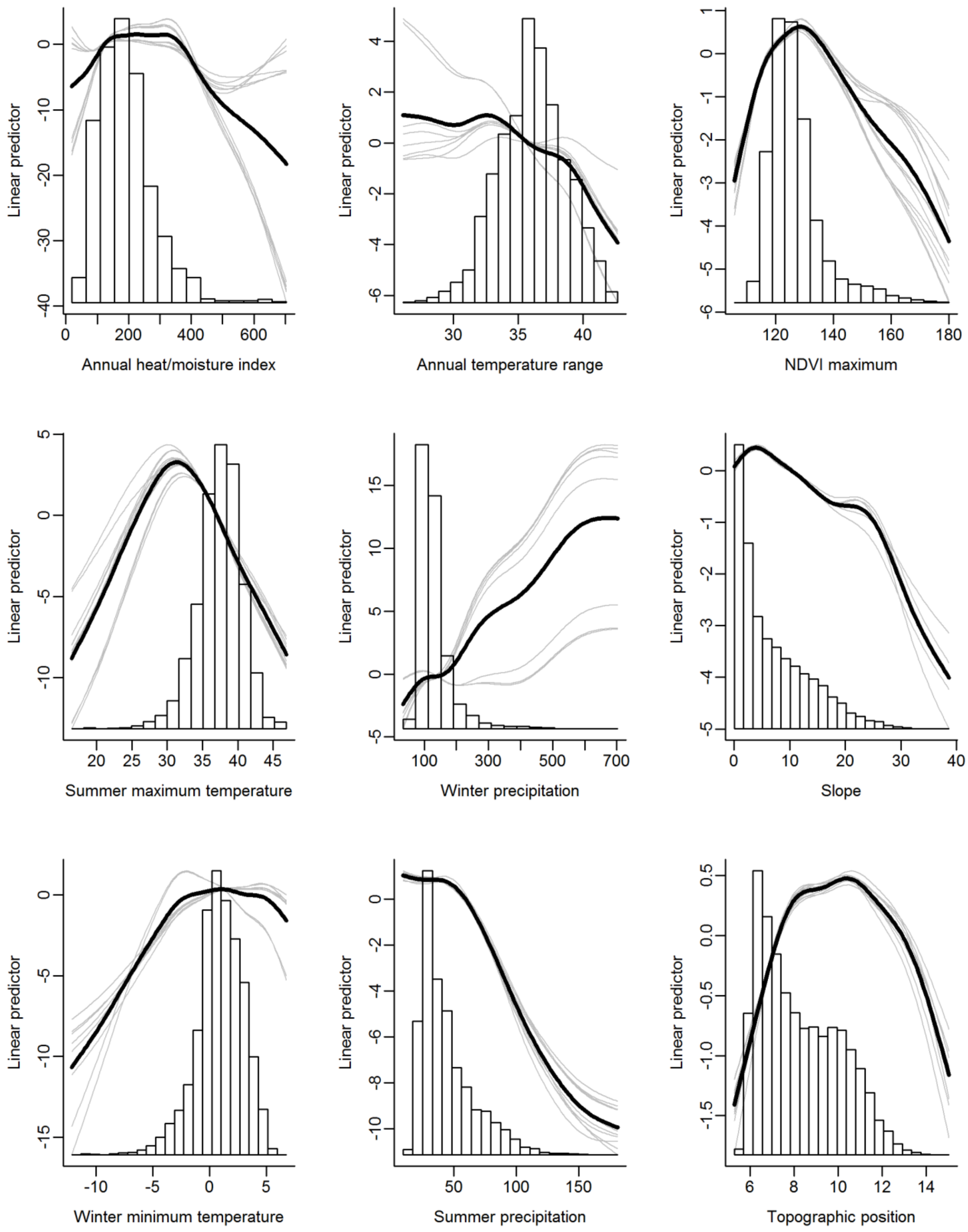

GAM response curves for *Ericameria cooperi*

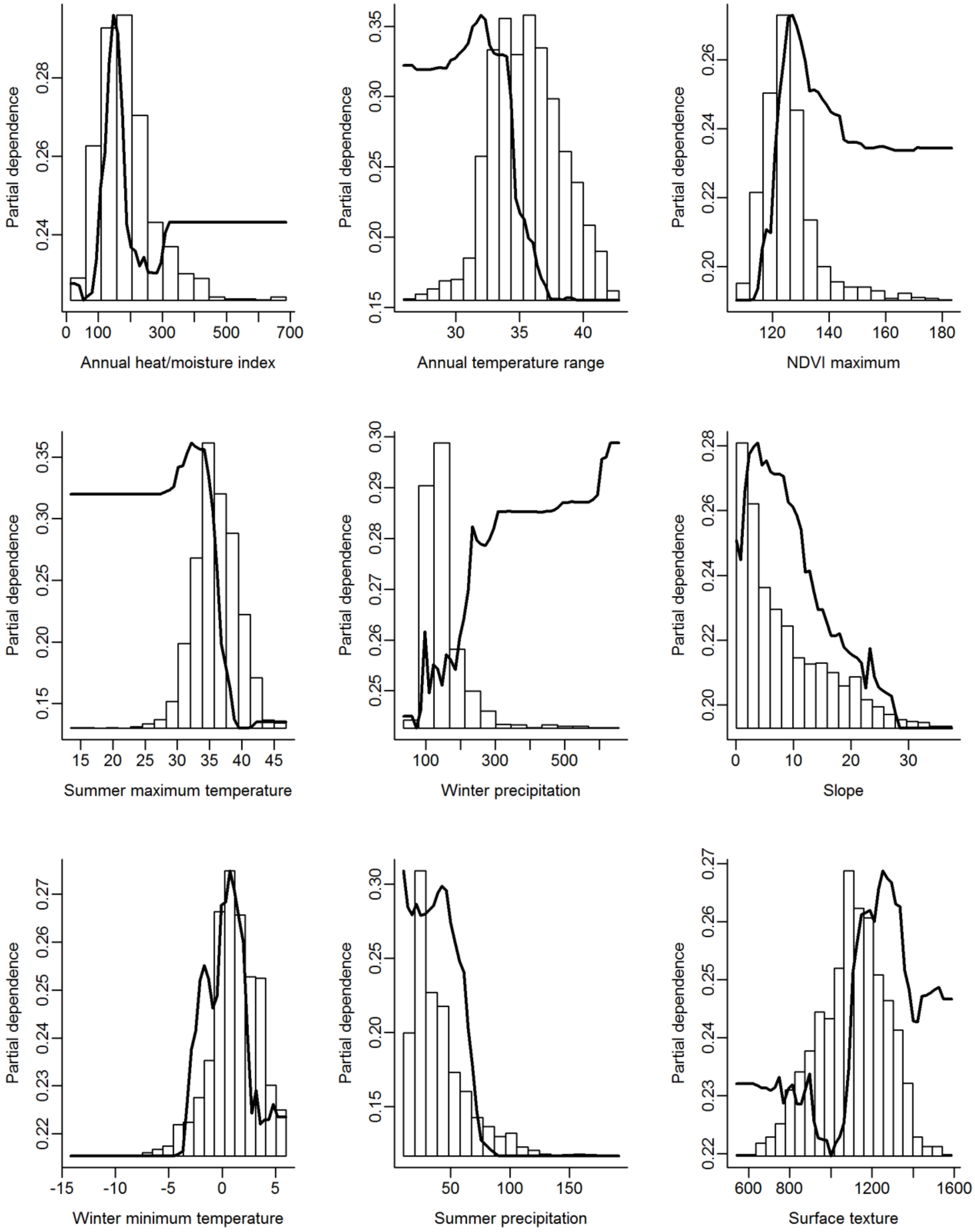

Random Forest response curves for *Ericameria cooperi*

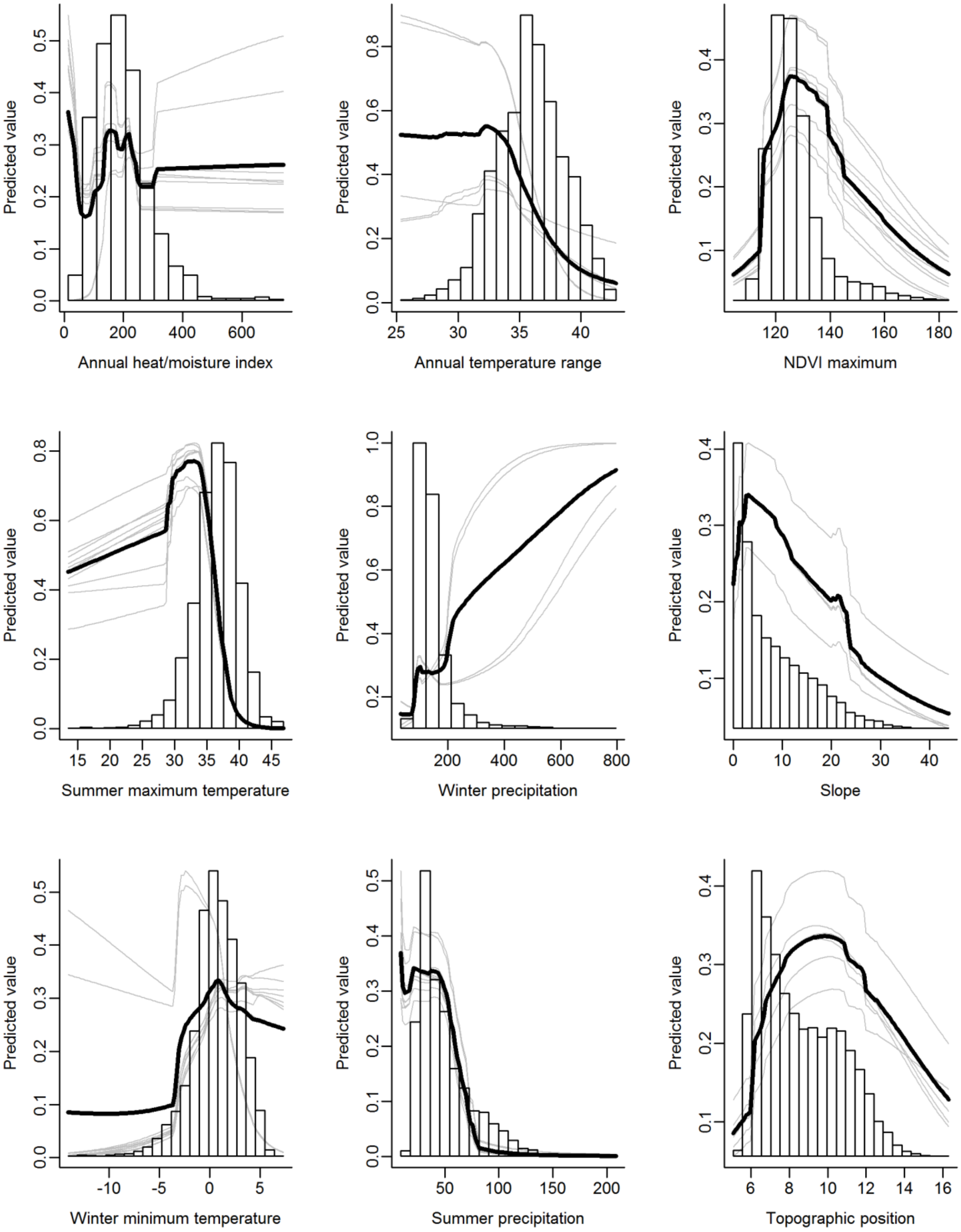

MaxEnt response curves for *Ericameria cooperi*

21. *Eriogonum fasciculatum* SDM and response curves (GAM, Random Forest, Maxent)

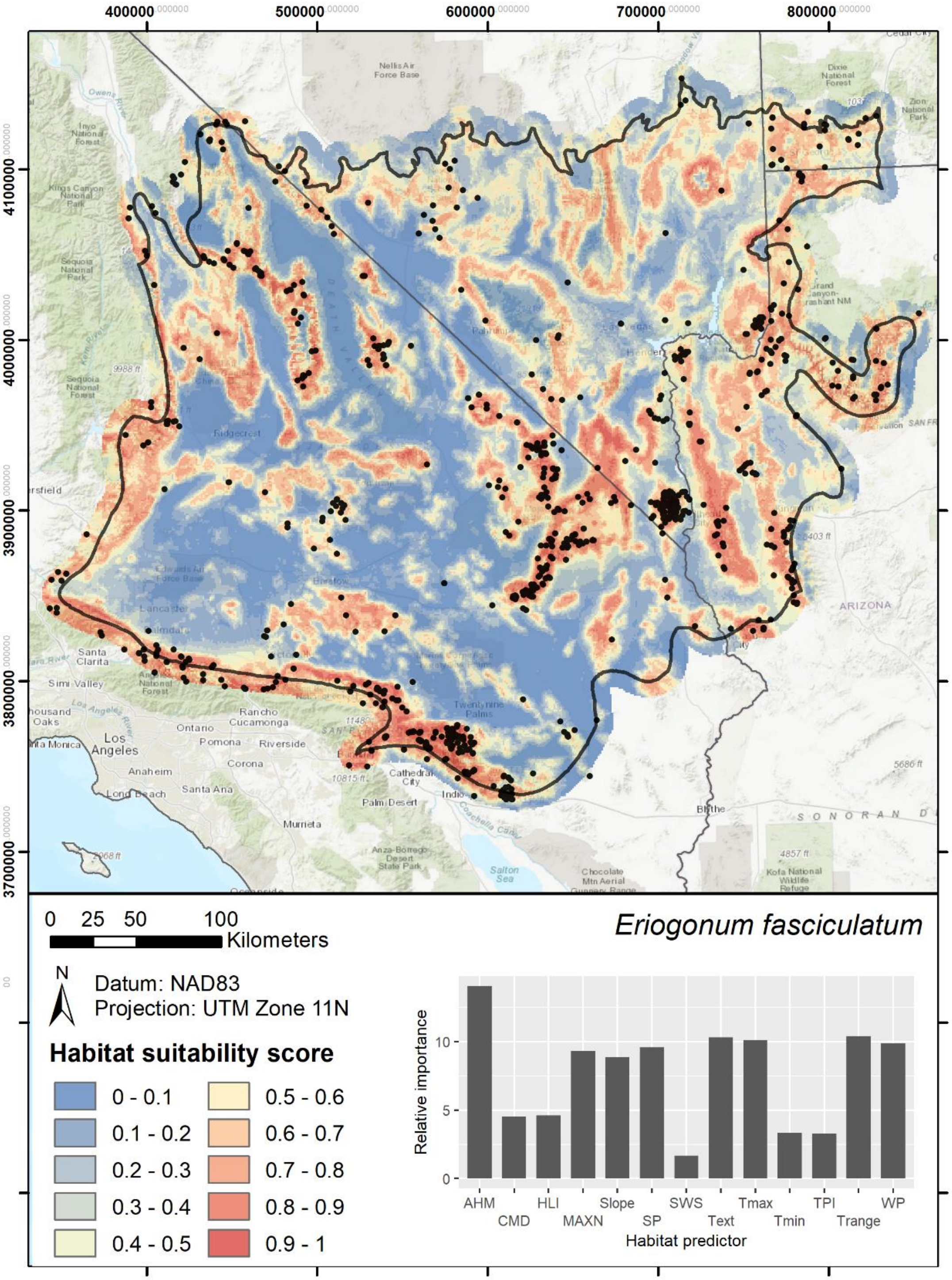

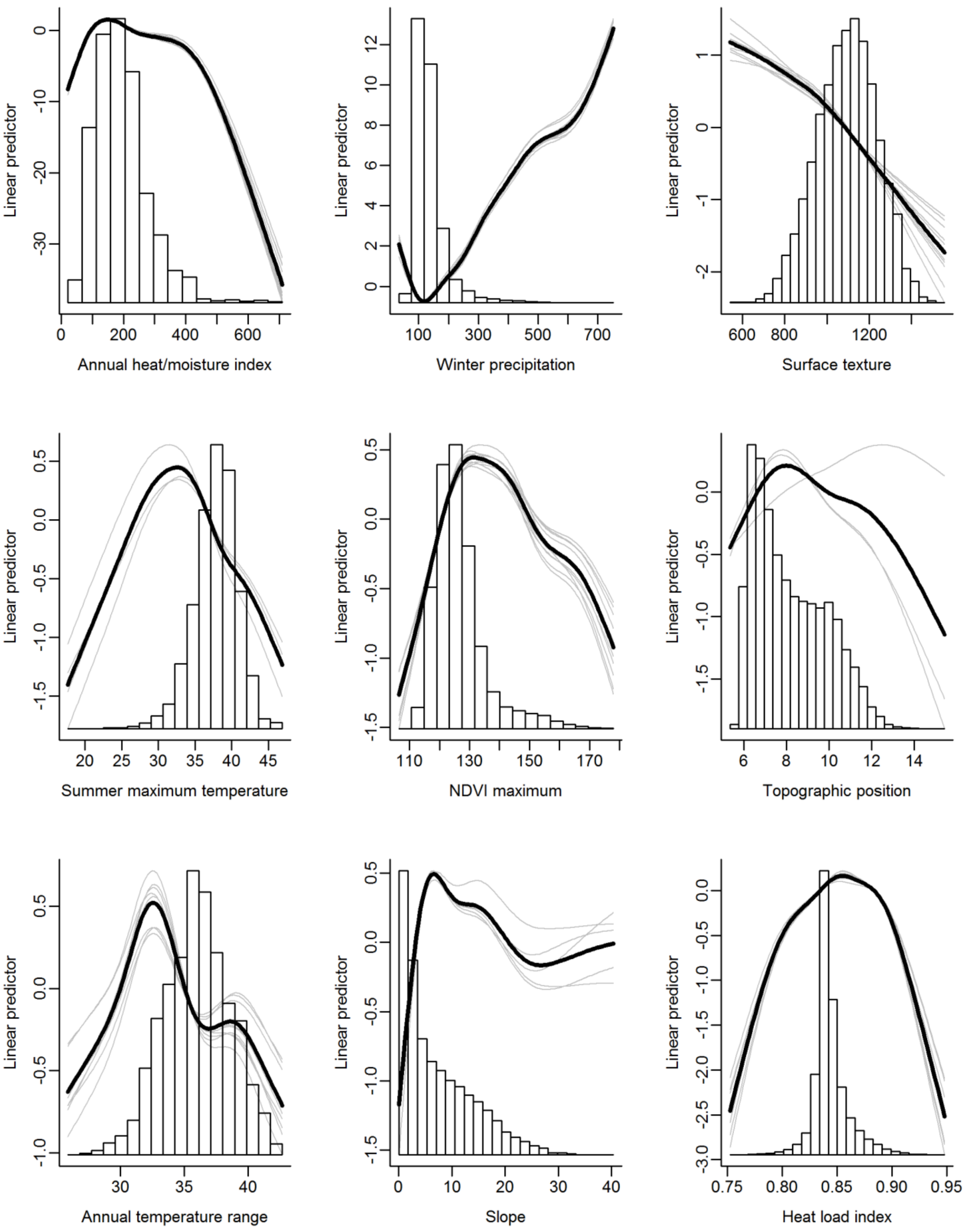

GAM response curves for *Eriogonum fasciculatum*

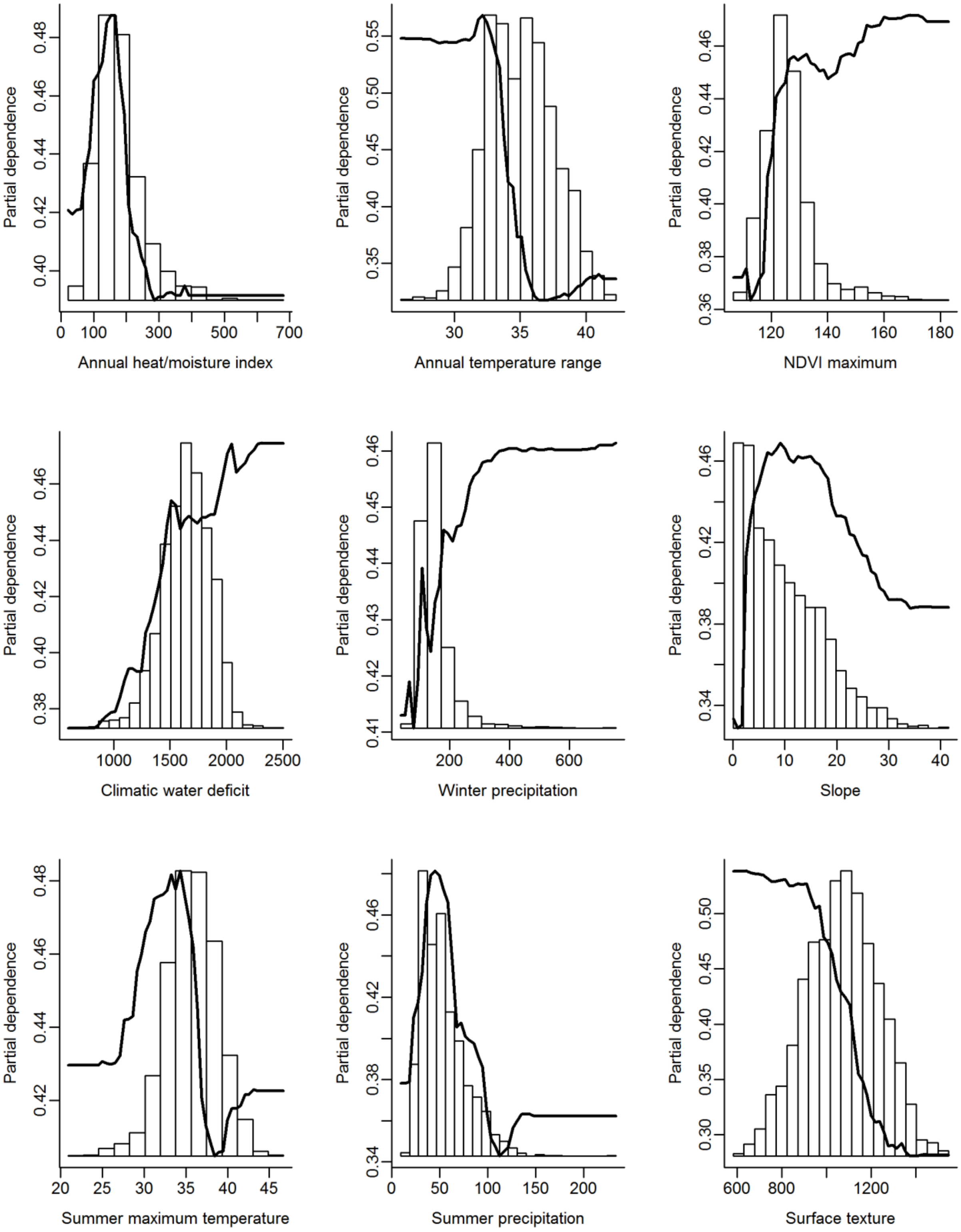

Random Forest response curves for *Eriogonum fasciculatum*

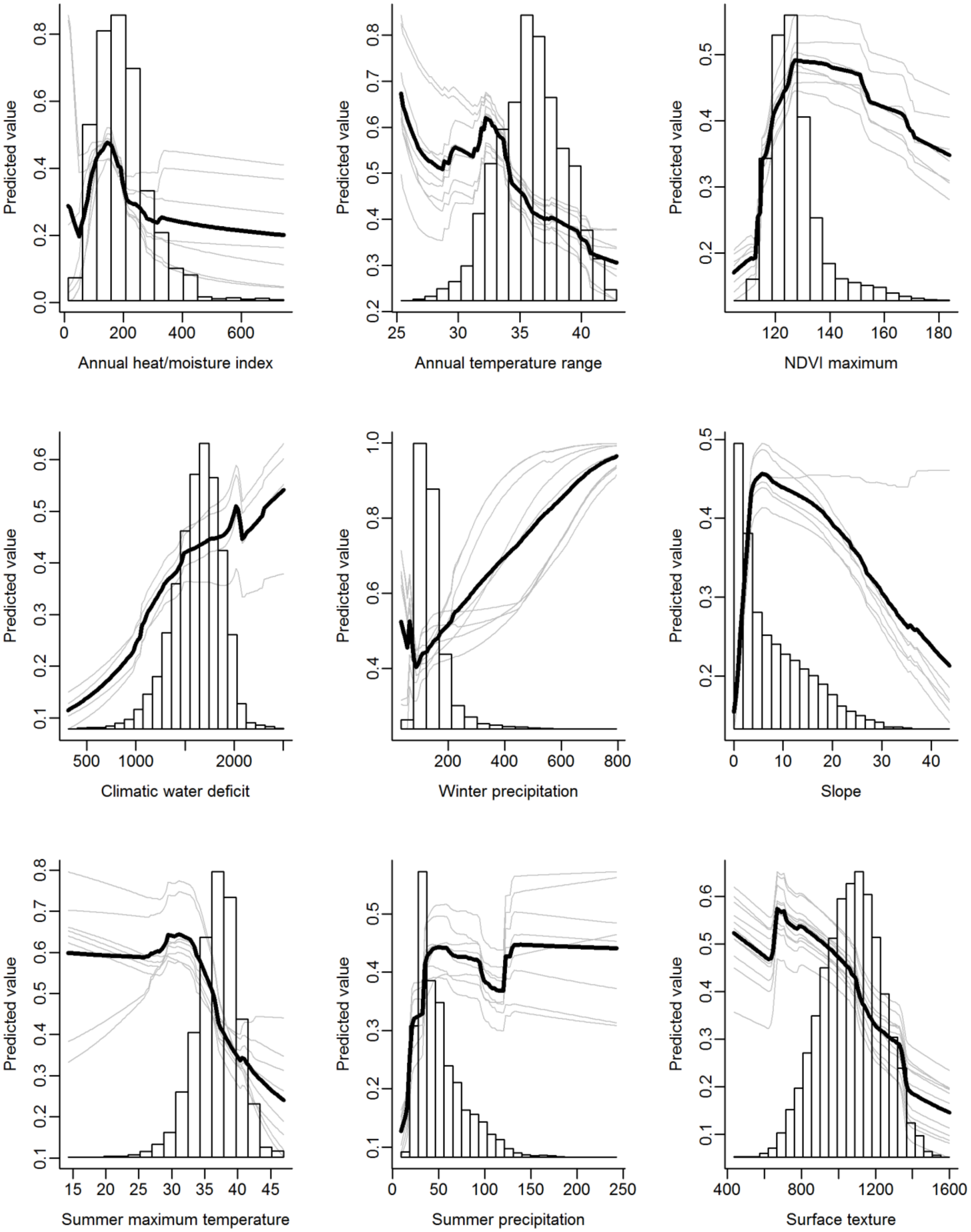

MaxEnt response curves for *Eriogonum fasciculatum*

22. *Eriogonum inflatum* SDM and response curves (GAM, Random Forest, Maxent)

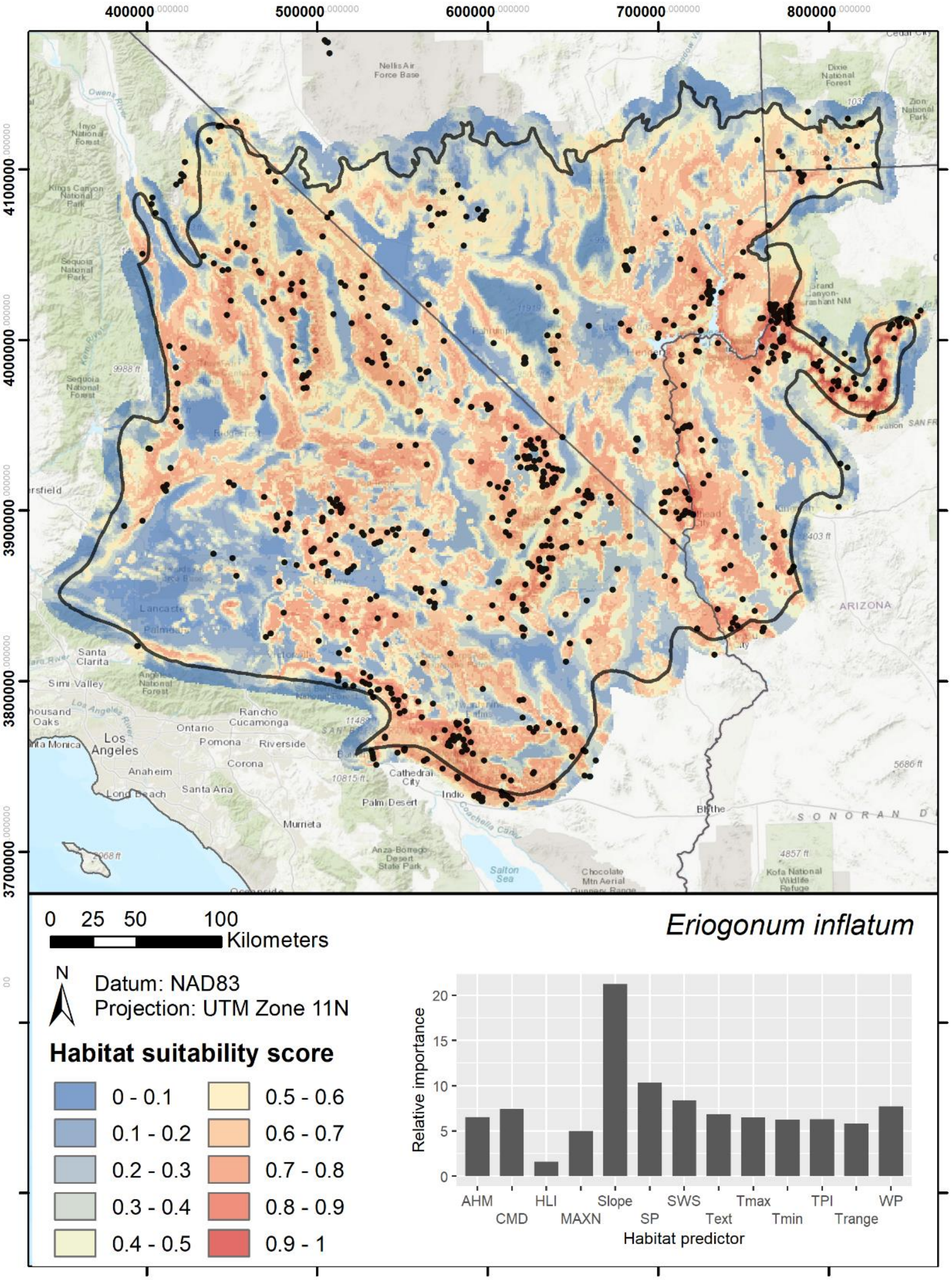

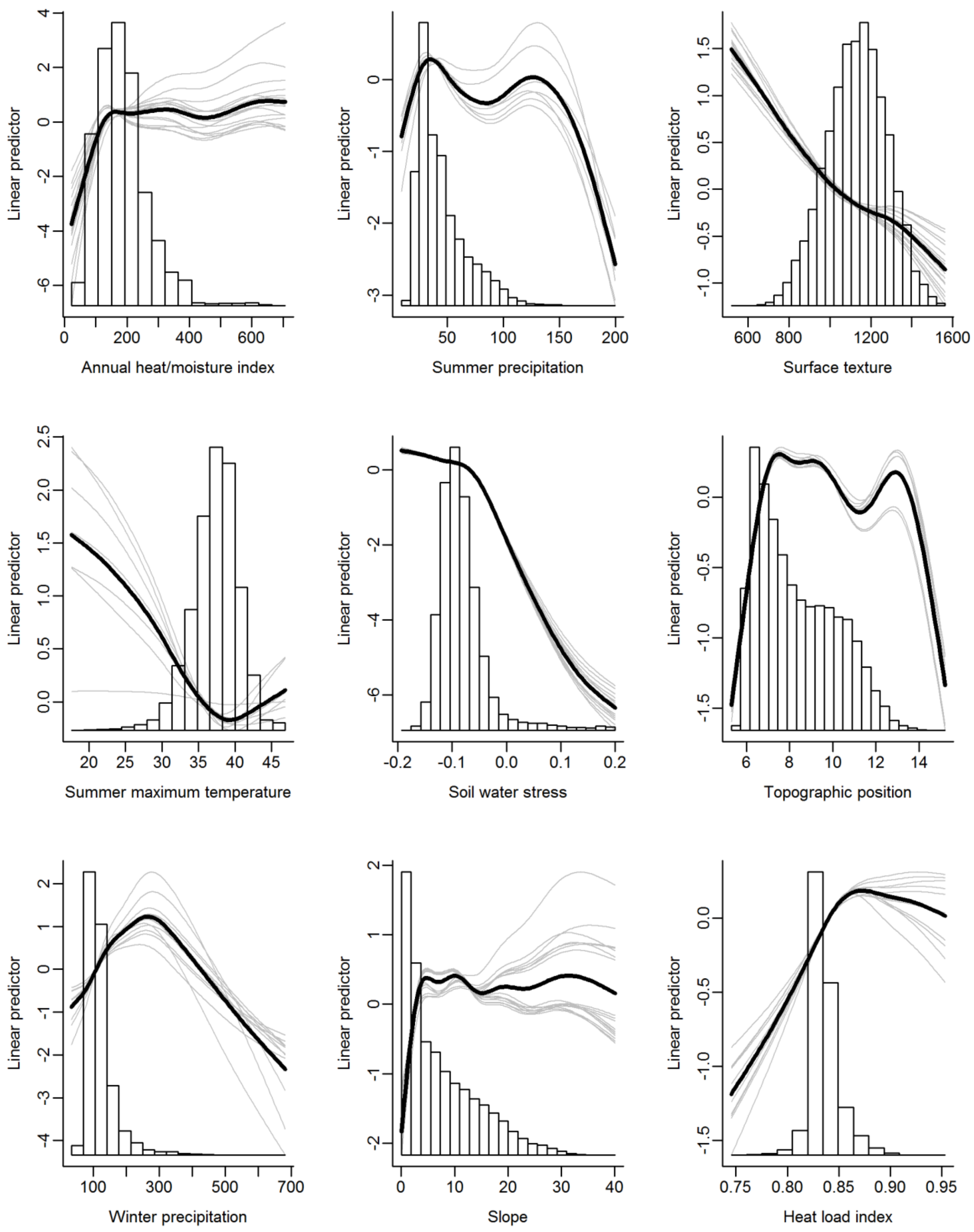

GAM response curves for *Eriogonum inflatum*

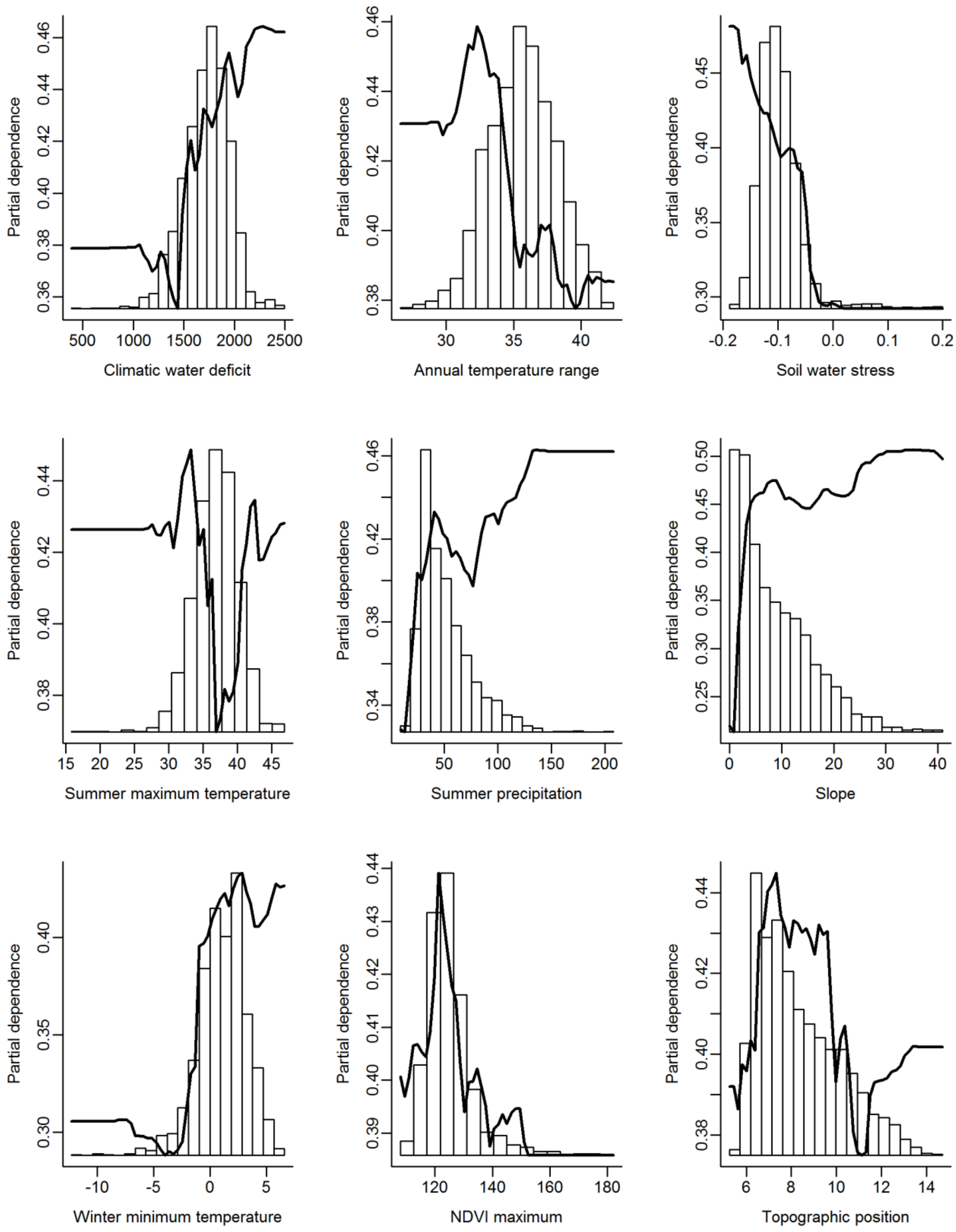

Random Forest response curves for *Eriogonum inflatum*

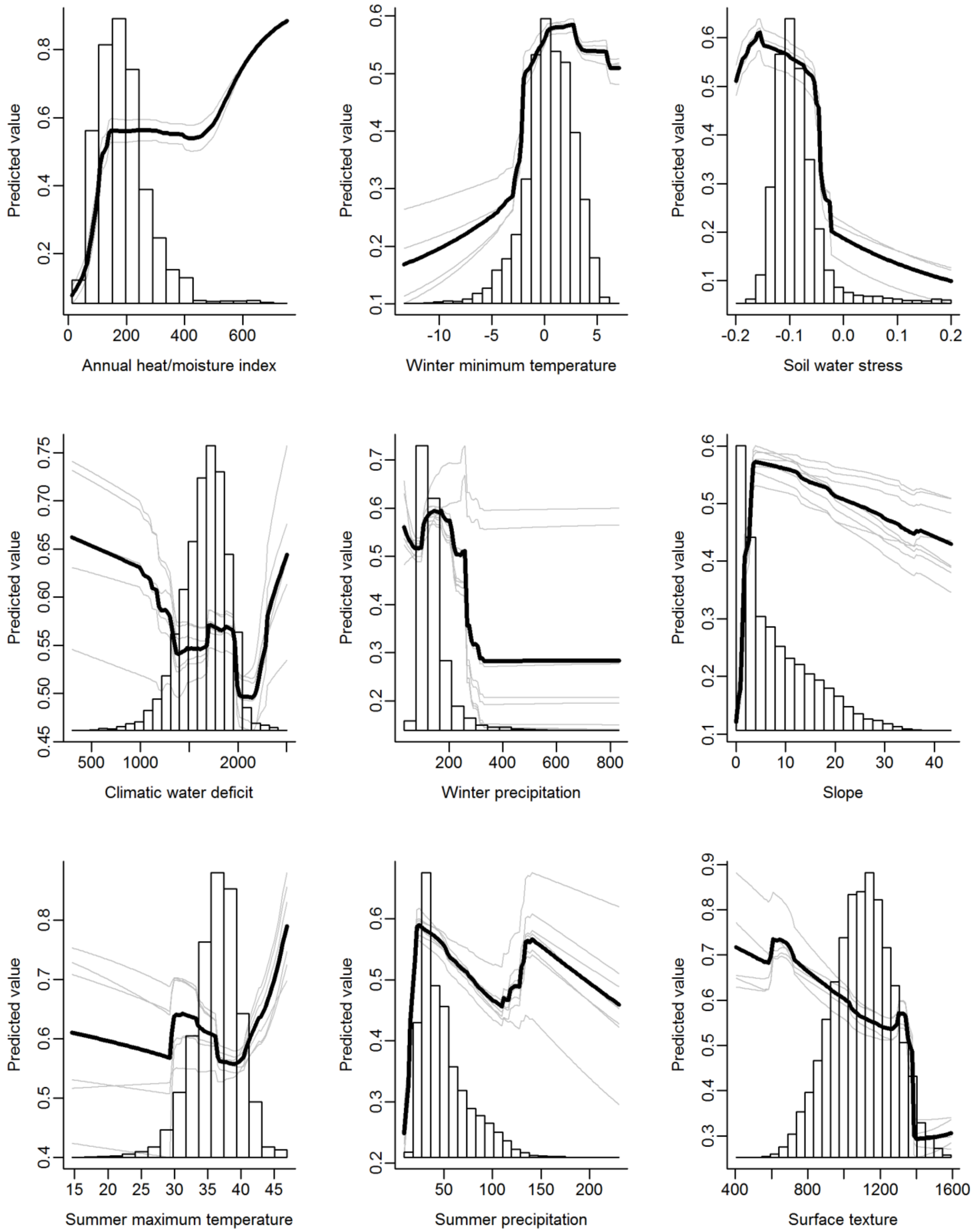

MaxEnt response curves for *Eriogonum inflatum*

23. *Euphorbia albomarginata* SDM and response curves (GAM, Random Forest, Maxent)

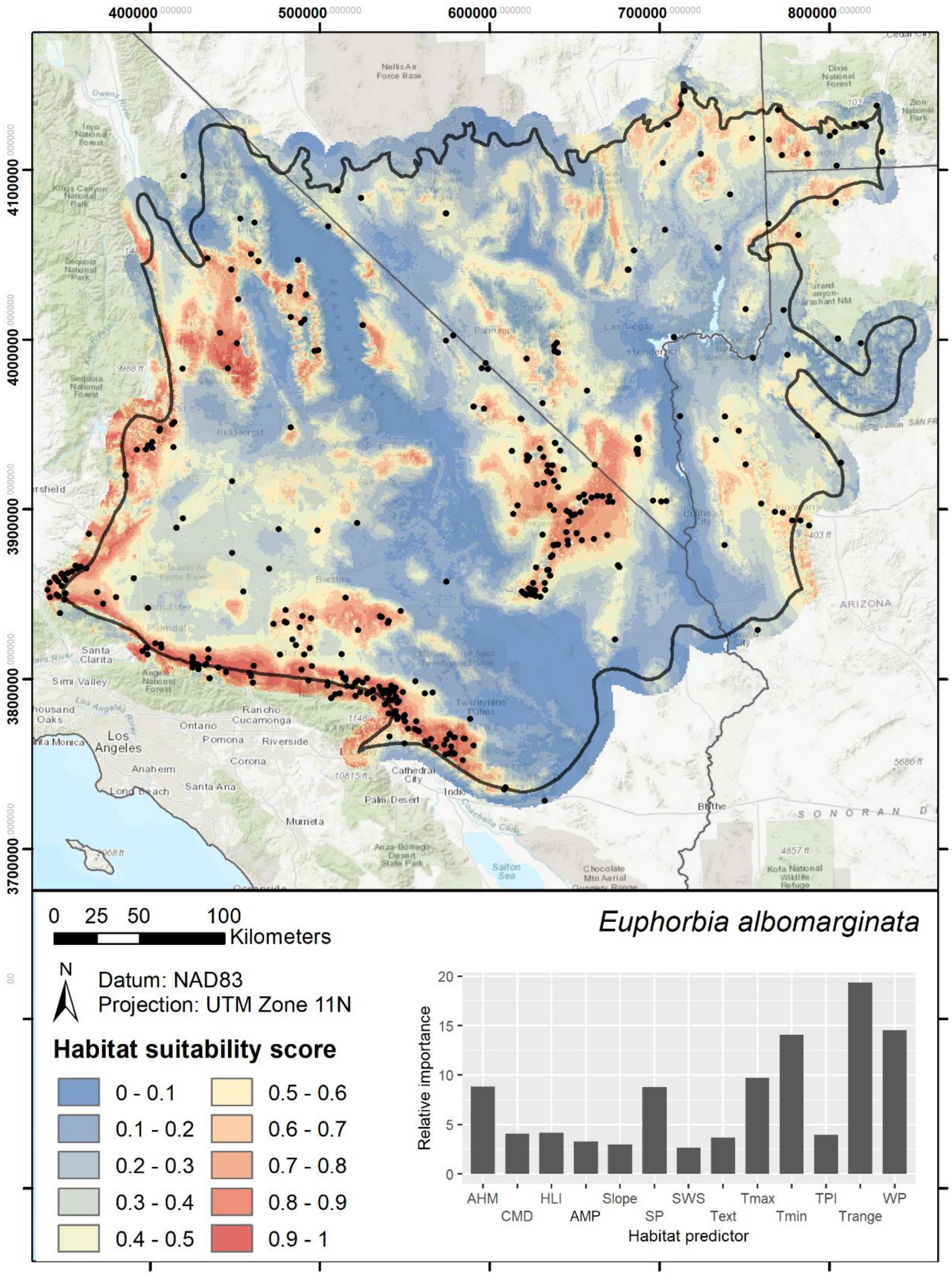

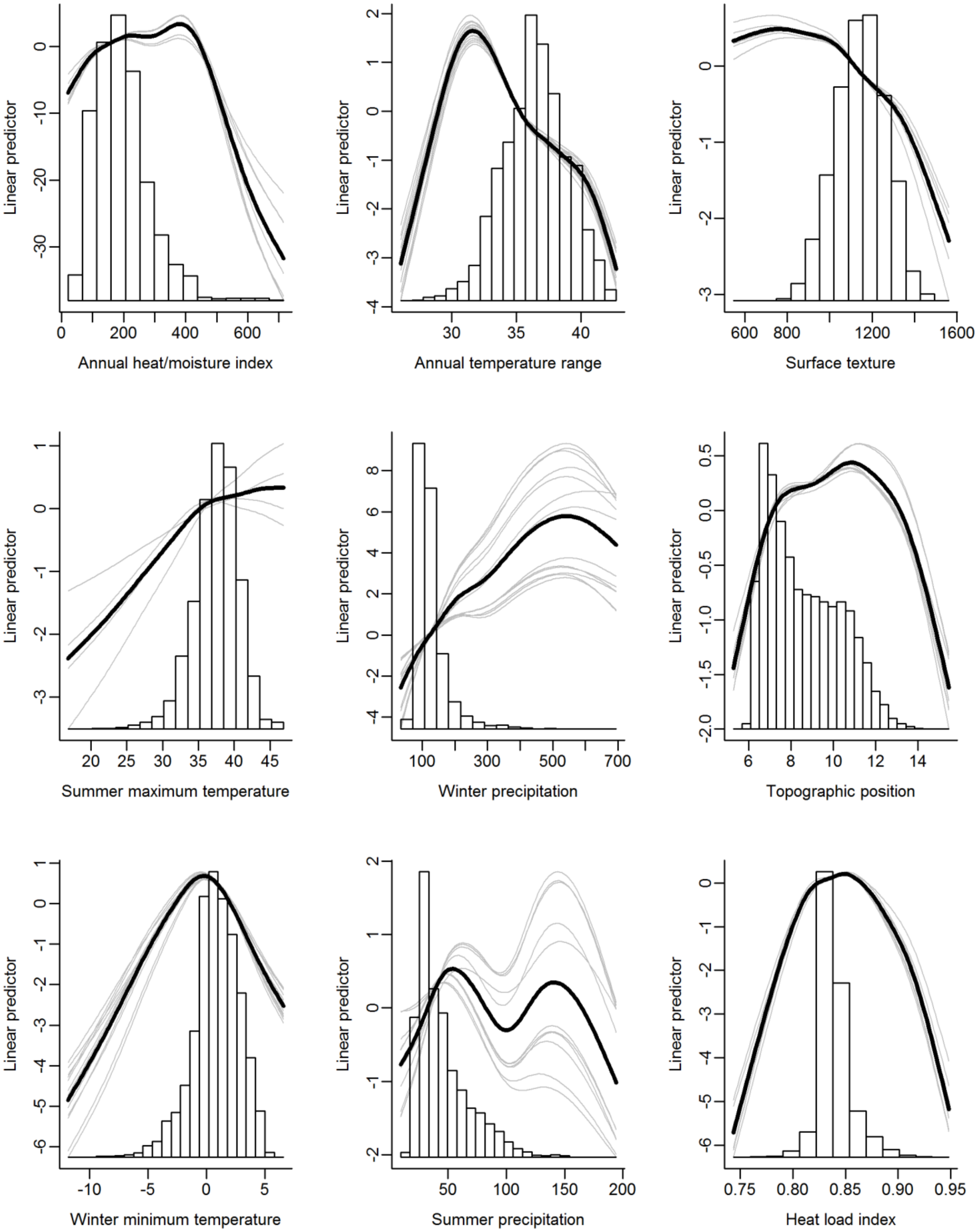

GAM response curves for *Euphorbia albomarginata*

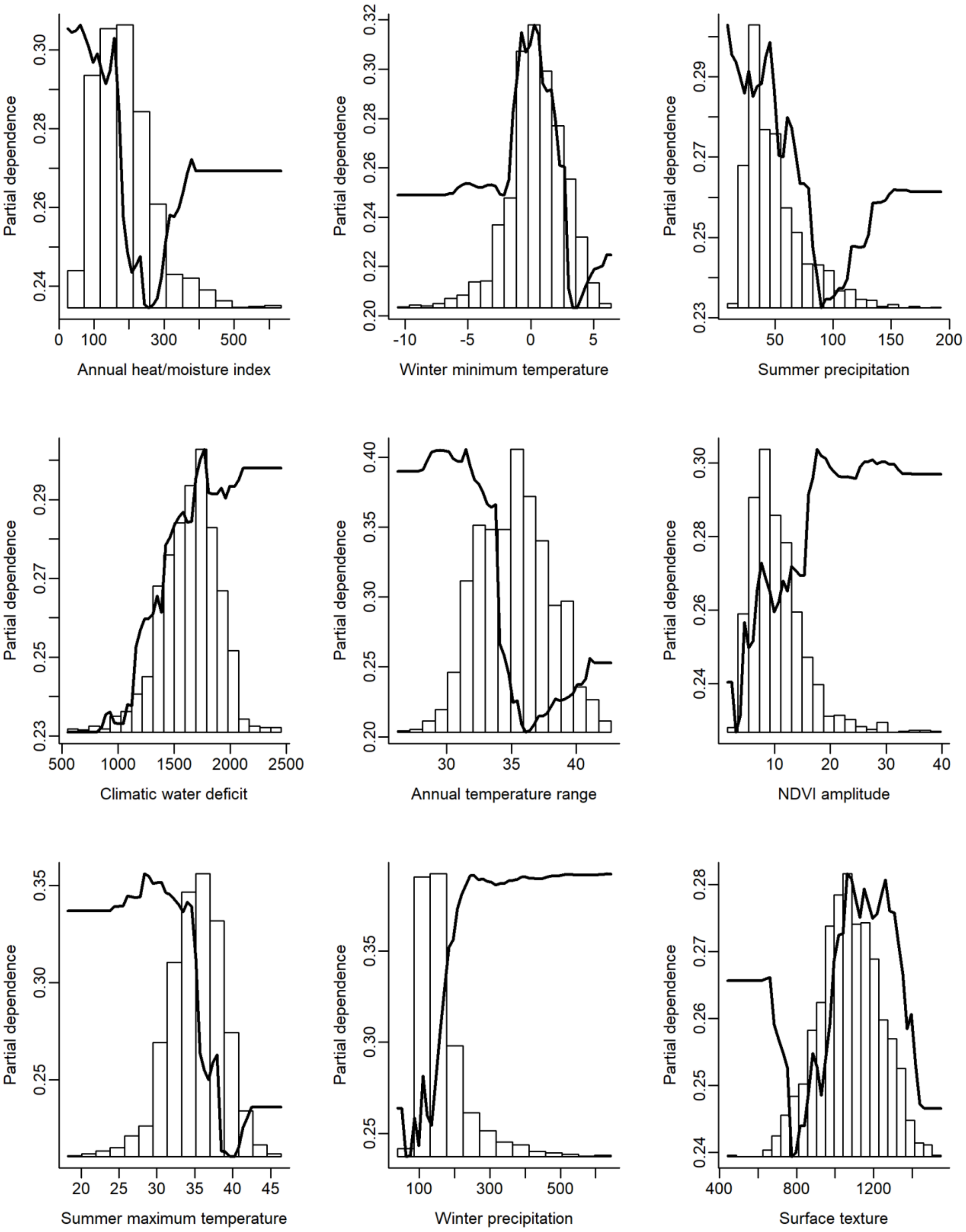

Random Forest response curves for *Euphorbia albomarginata*

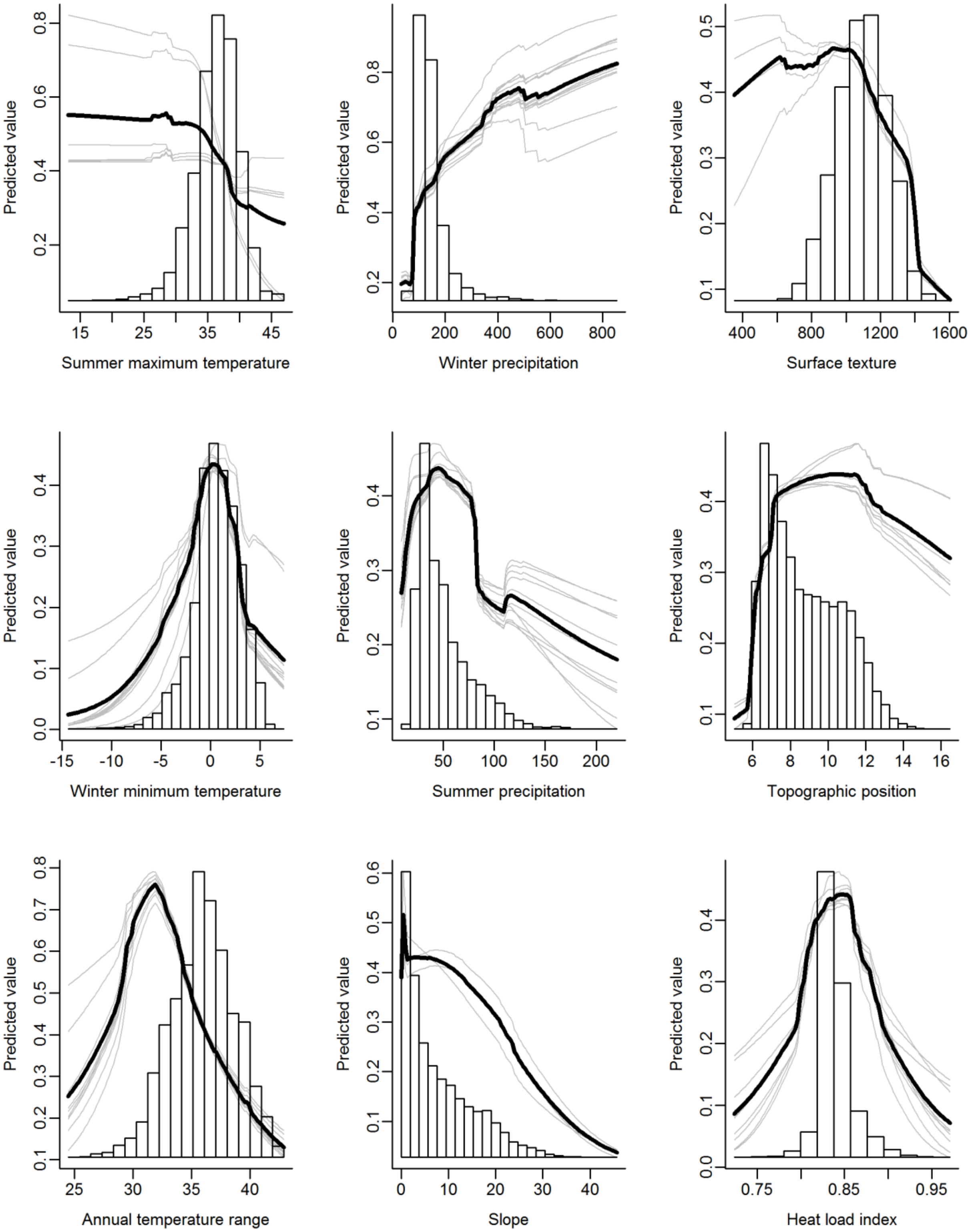

MaxEnt response curves for *Euphorbia albomarginata*

24. *Hilaria rigida* SDM and response curves (GAM, Random Forest, Maxent)

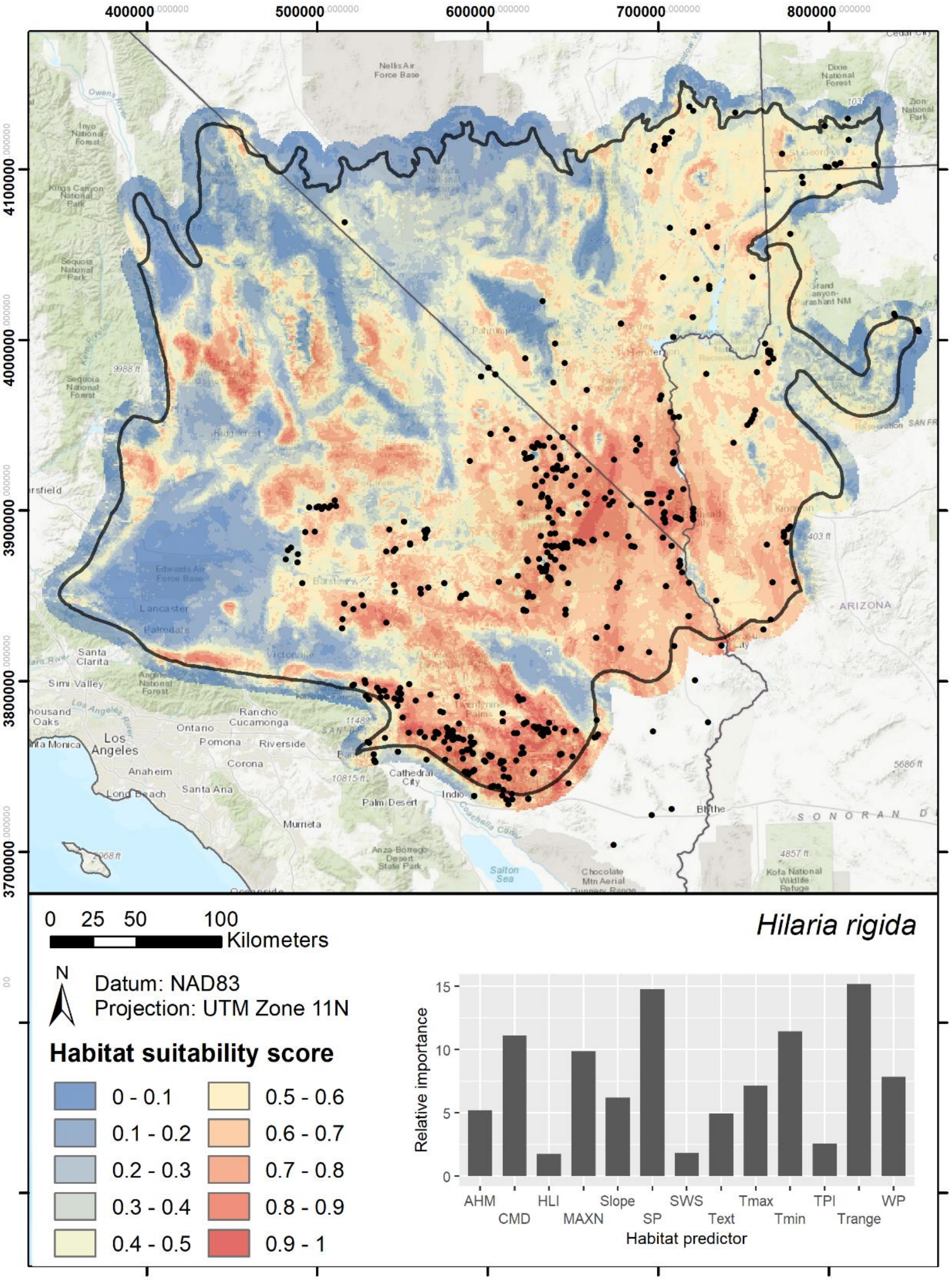

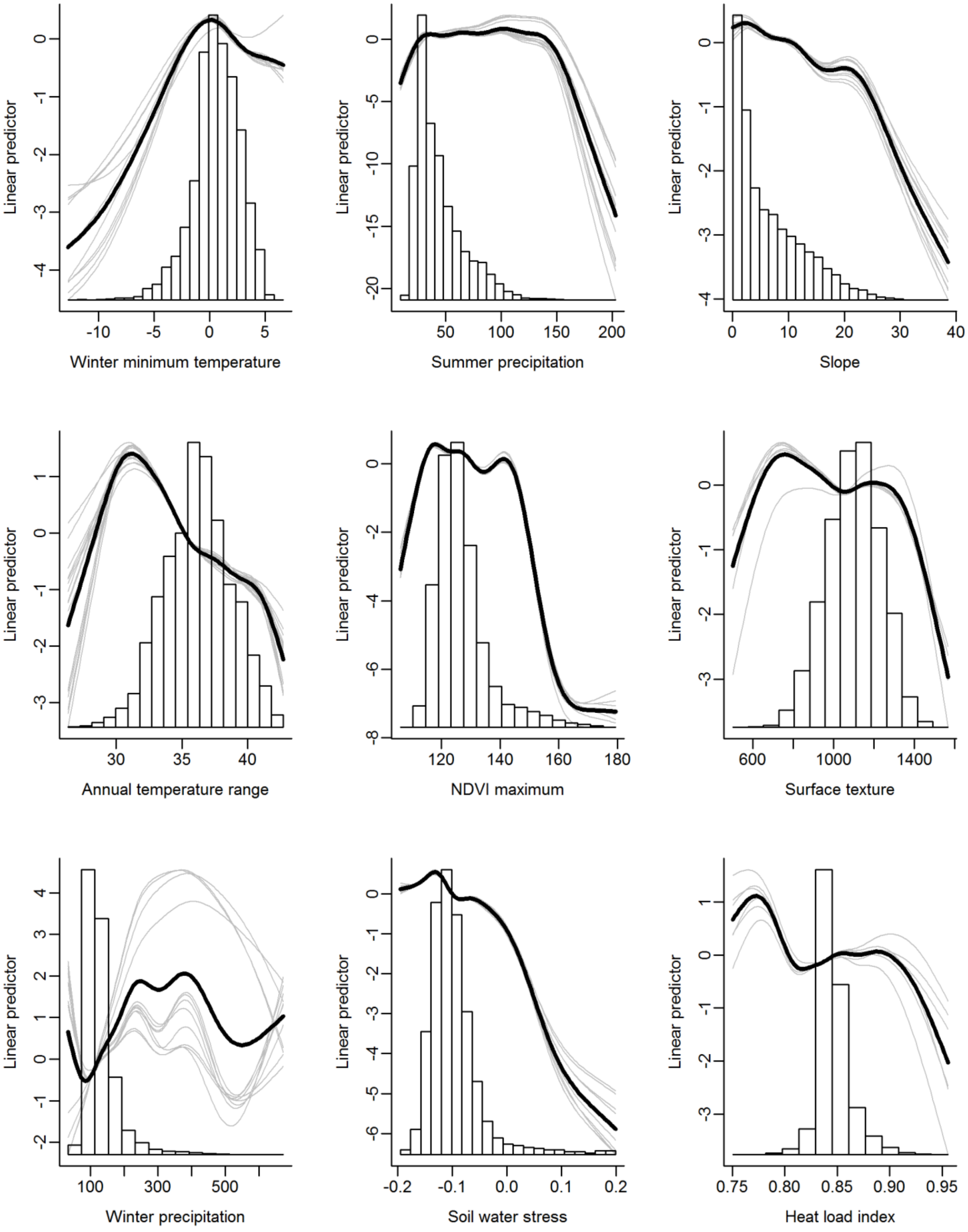

GAM response curves for *Hilaria rigida*

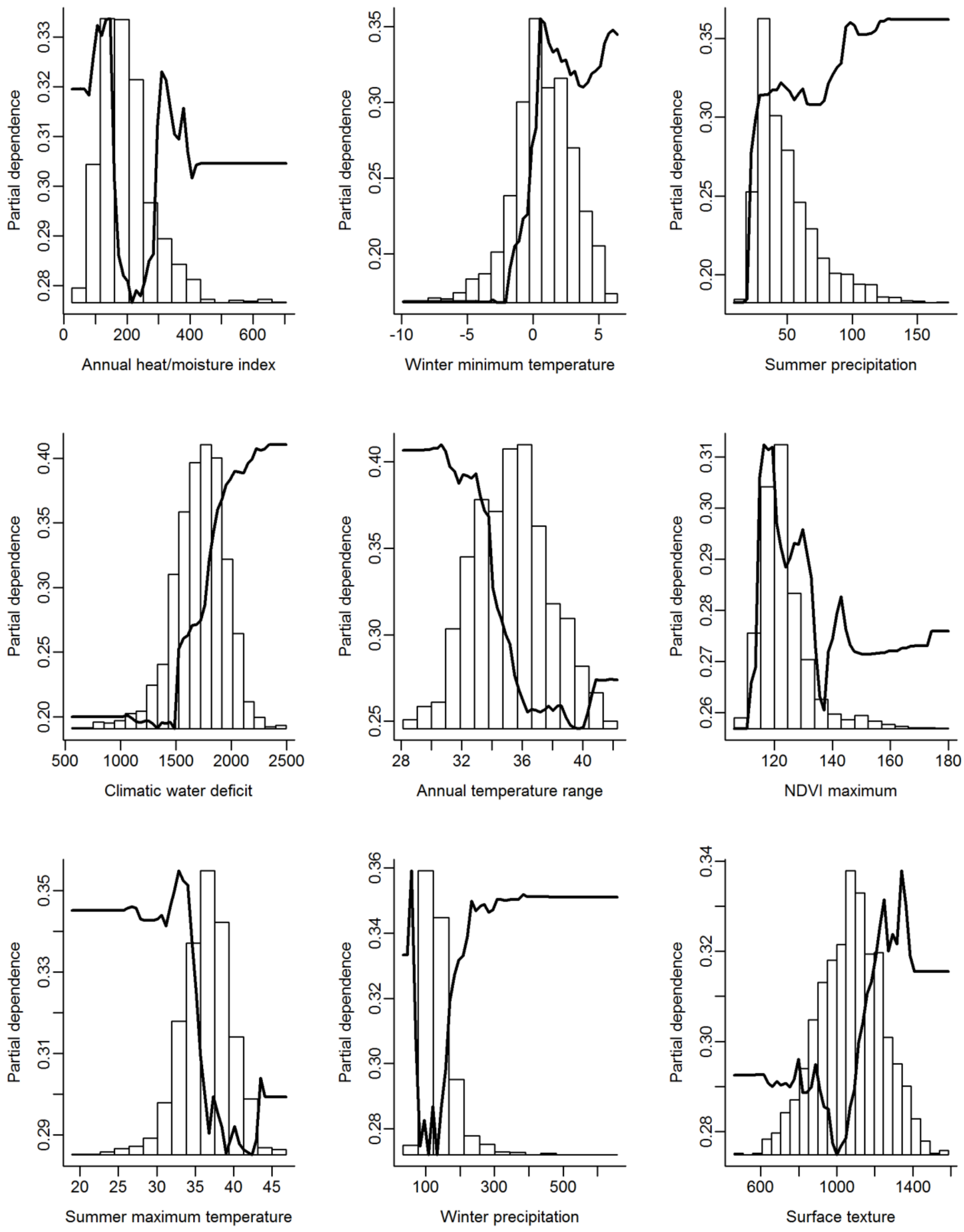

Random Forest response curves for *Hilaria rigida*

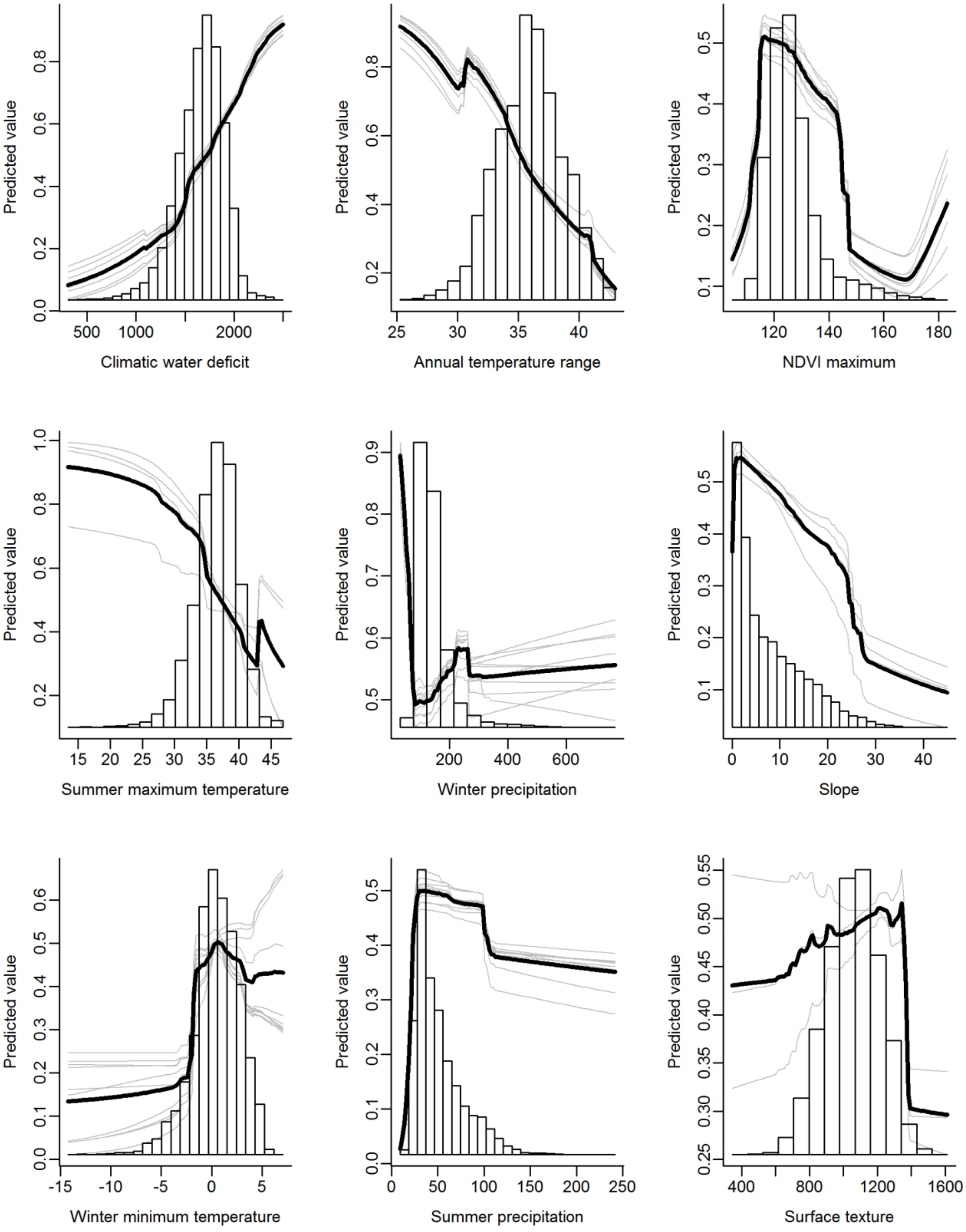

MaxEnt response curves for *Hilaria rigida*

25. *Krameria bicolor* SDM and response curves (GAM, Random Forest, Maxent)

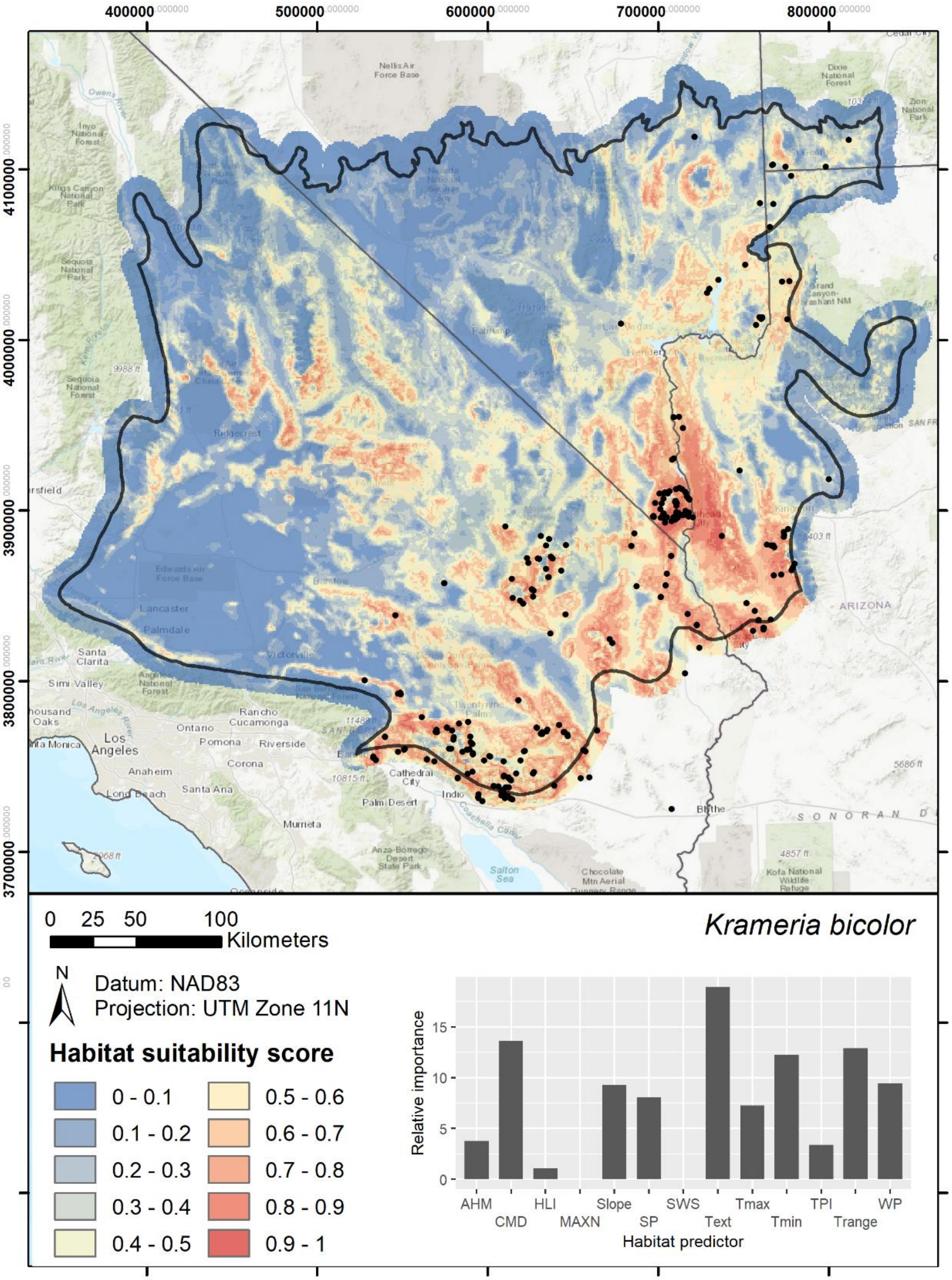

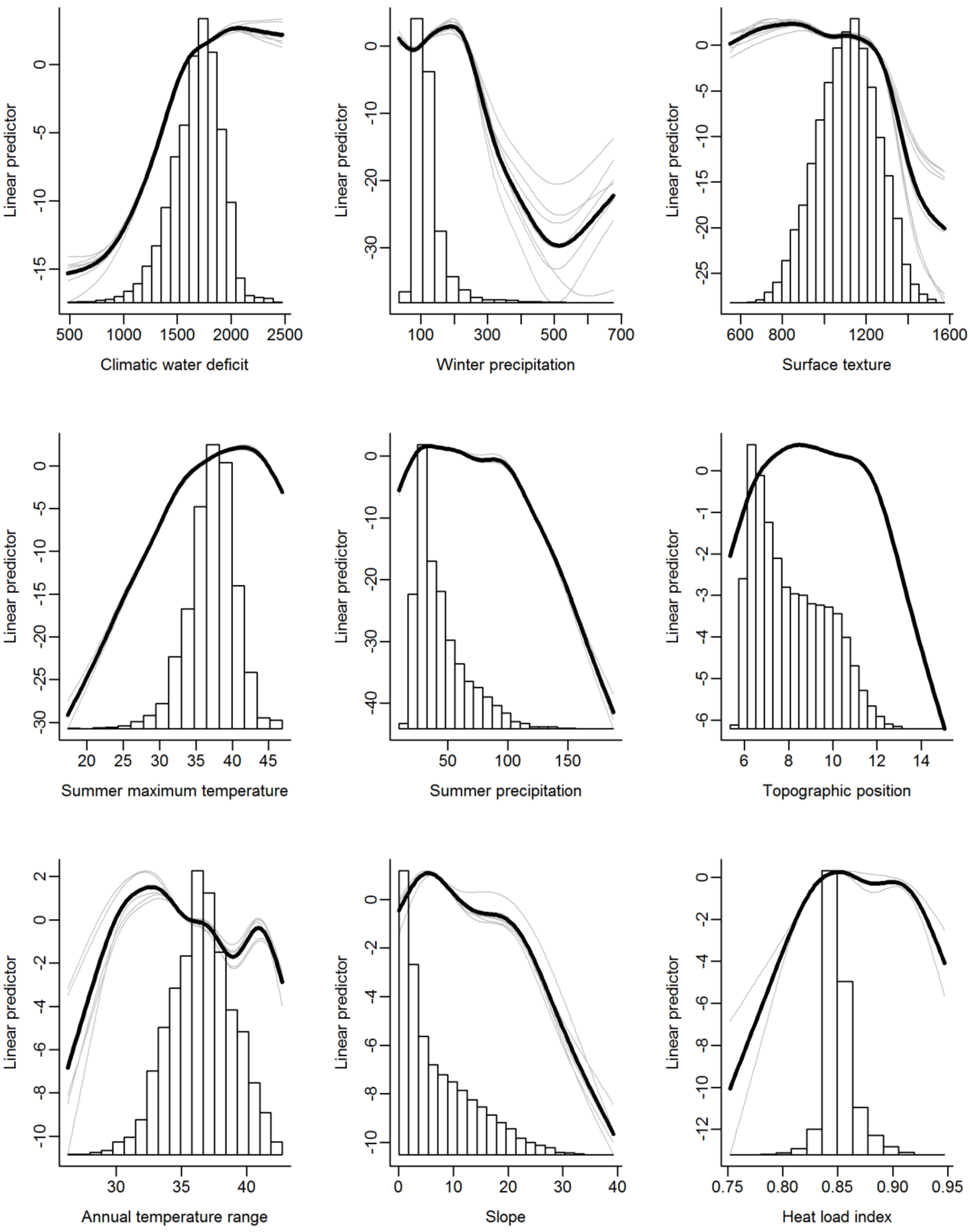

GAM response curves for *Krameria bicolor*

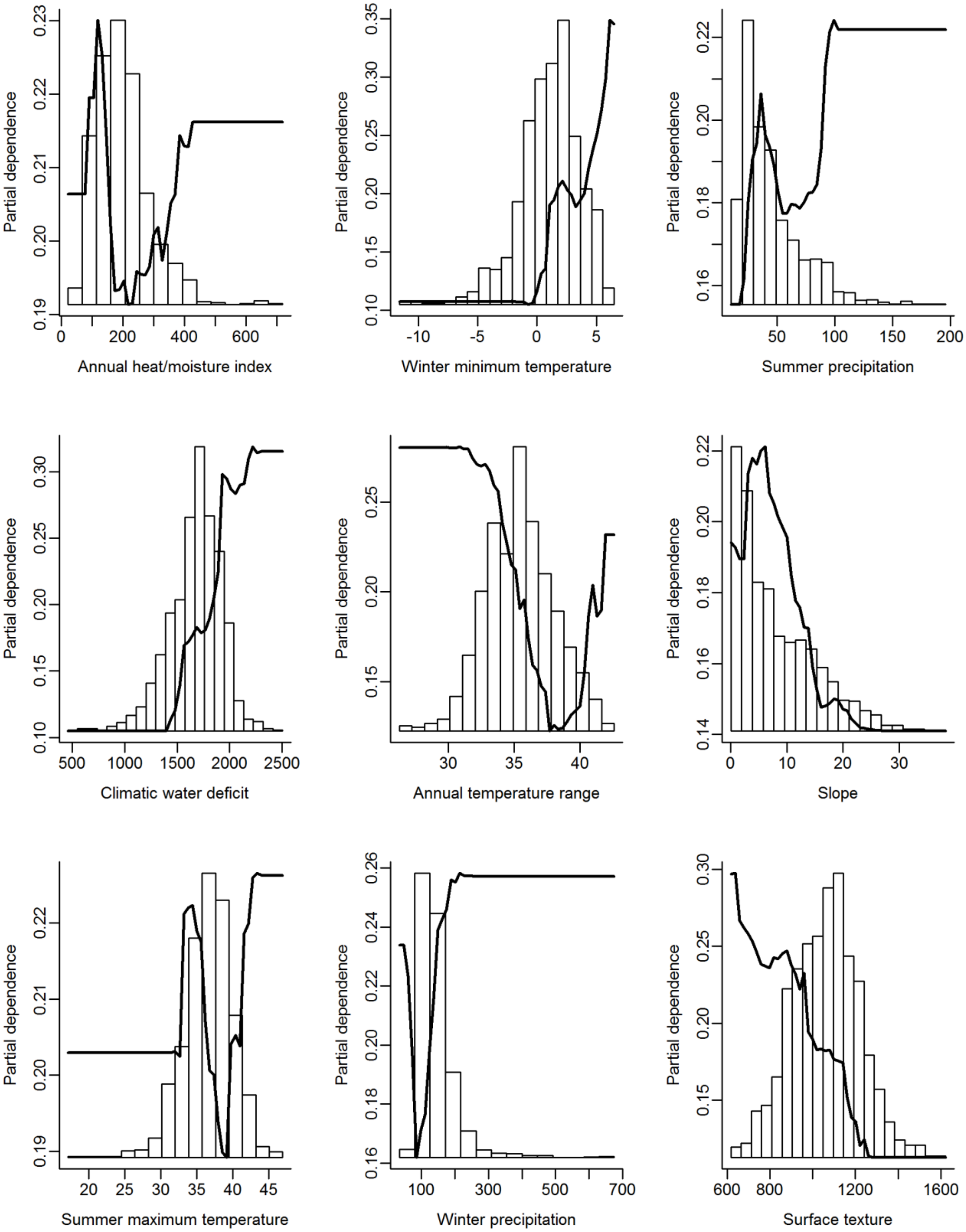

Random Forest response curves for *Krameria bicolor*

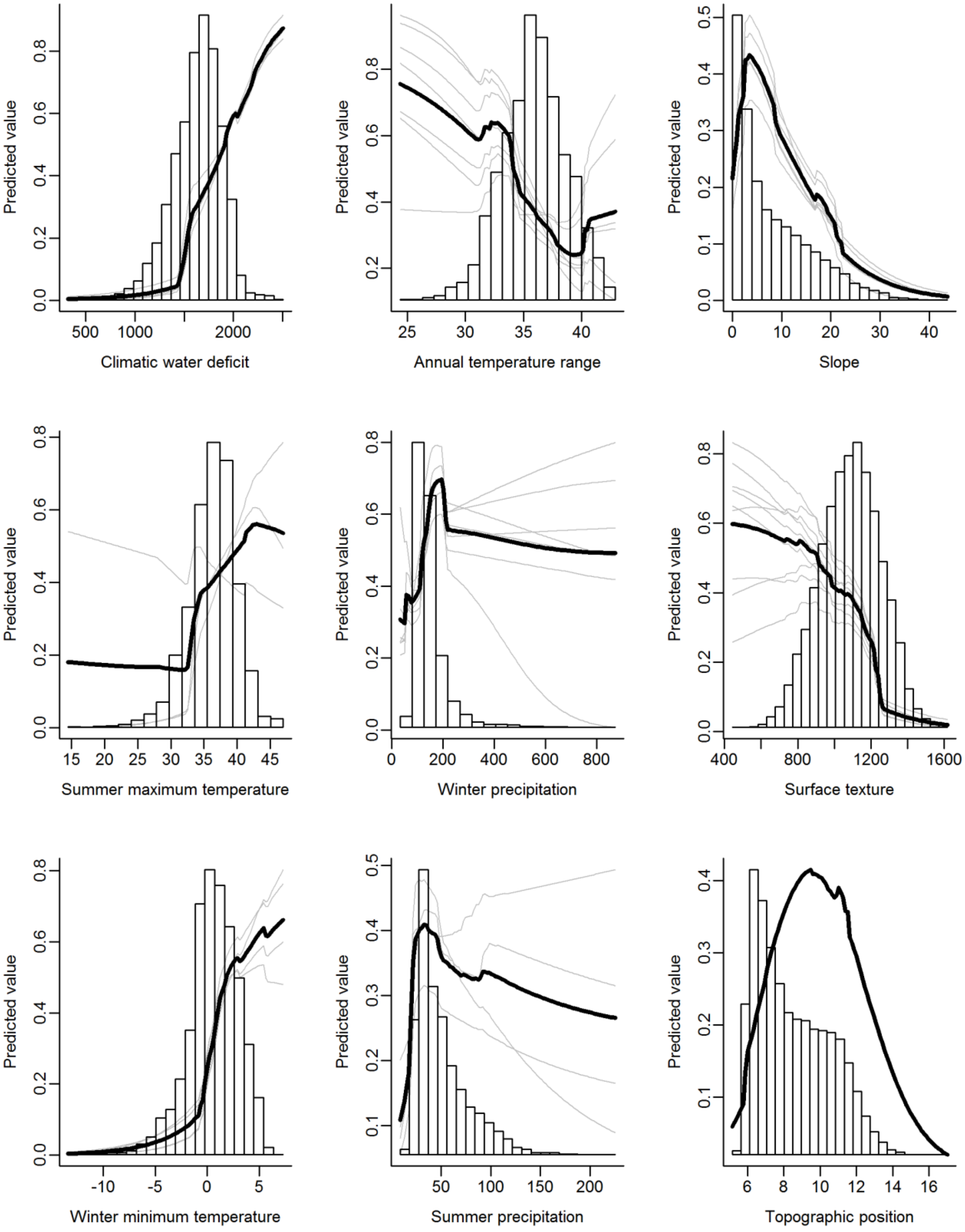

MaxEnt response curves for *Krameria bicolor*

26. *Krameria erecta* SDM and response curves (GAM, Random Forest, Maxent)

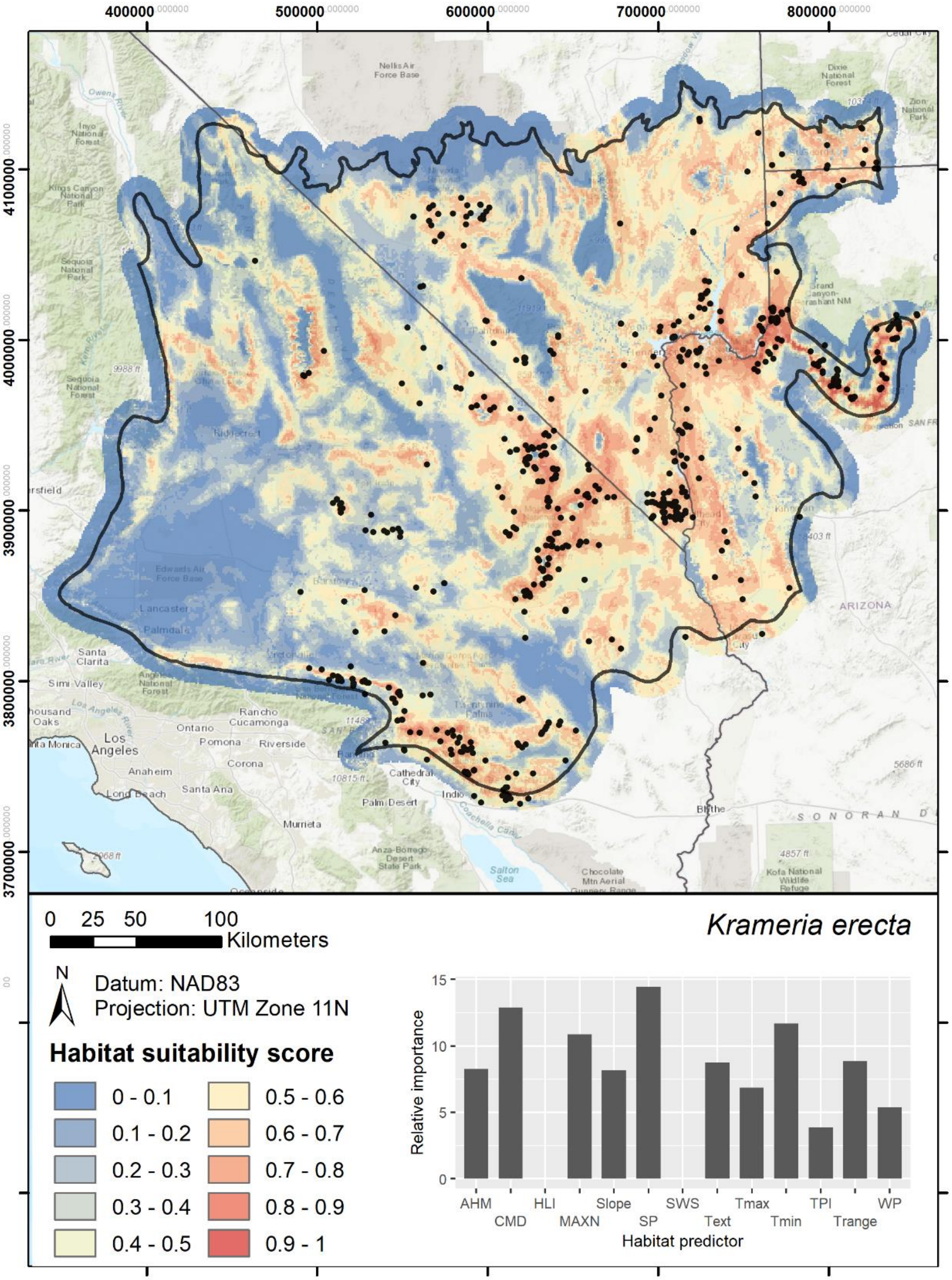

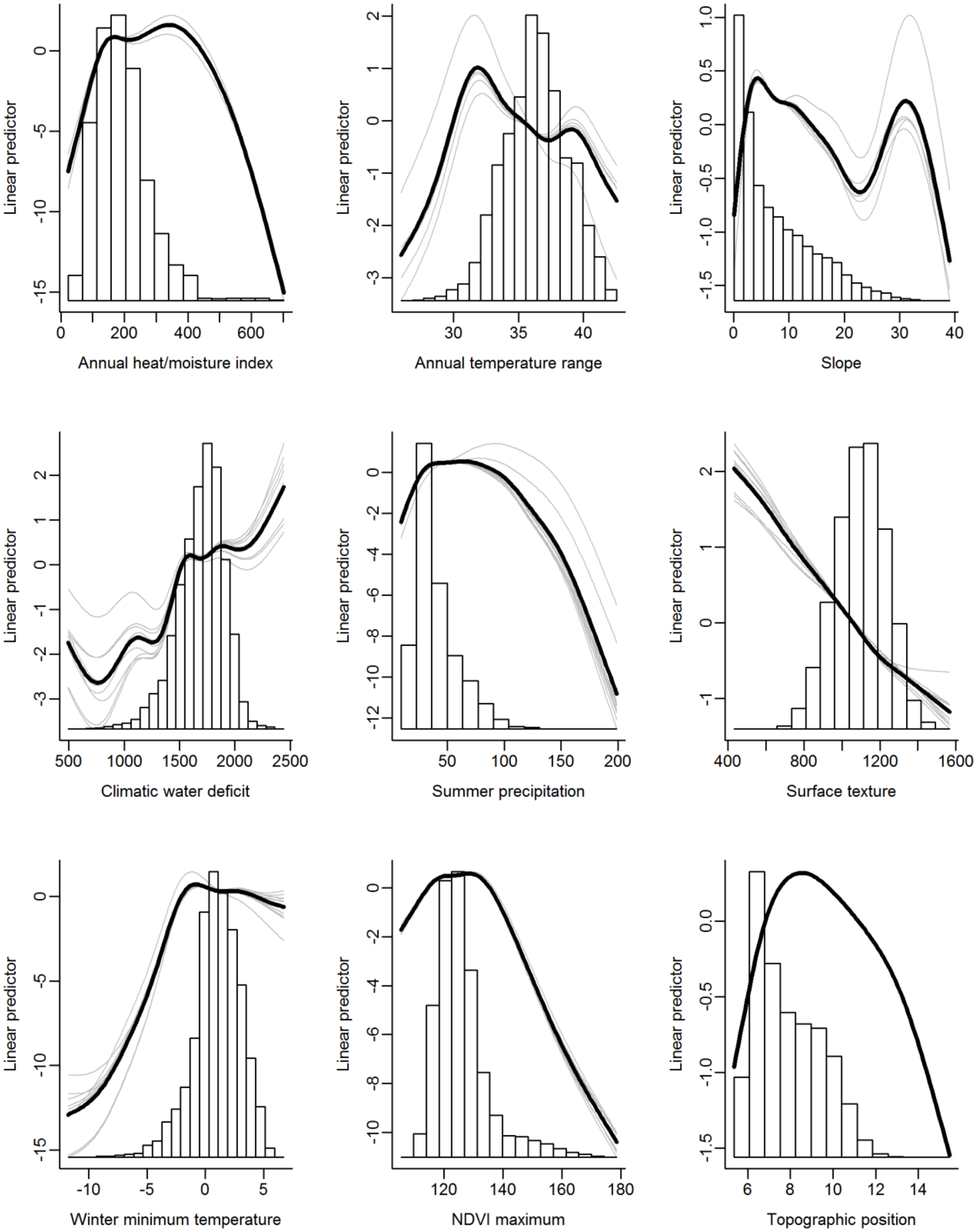

GAM response curves for *Krameria erecta*

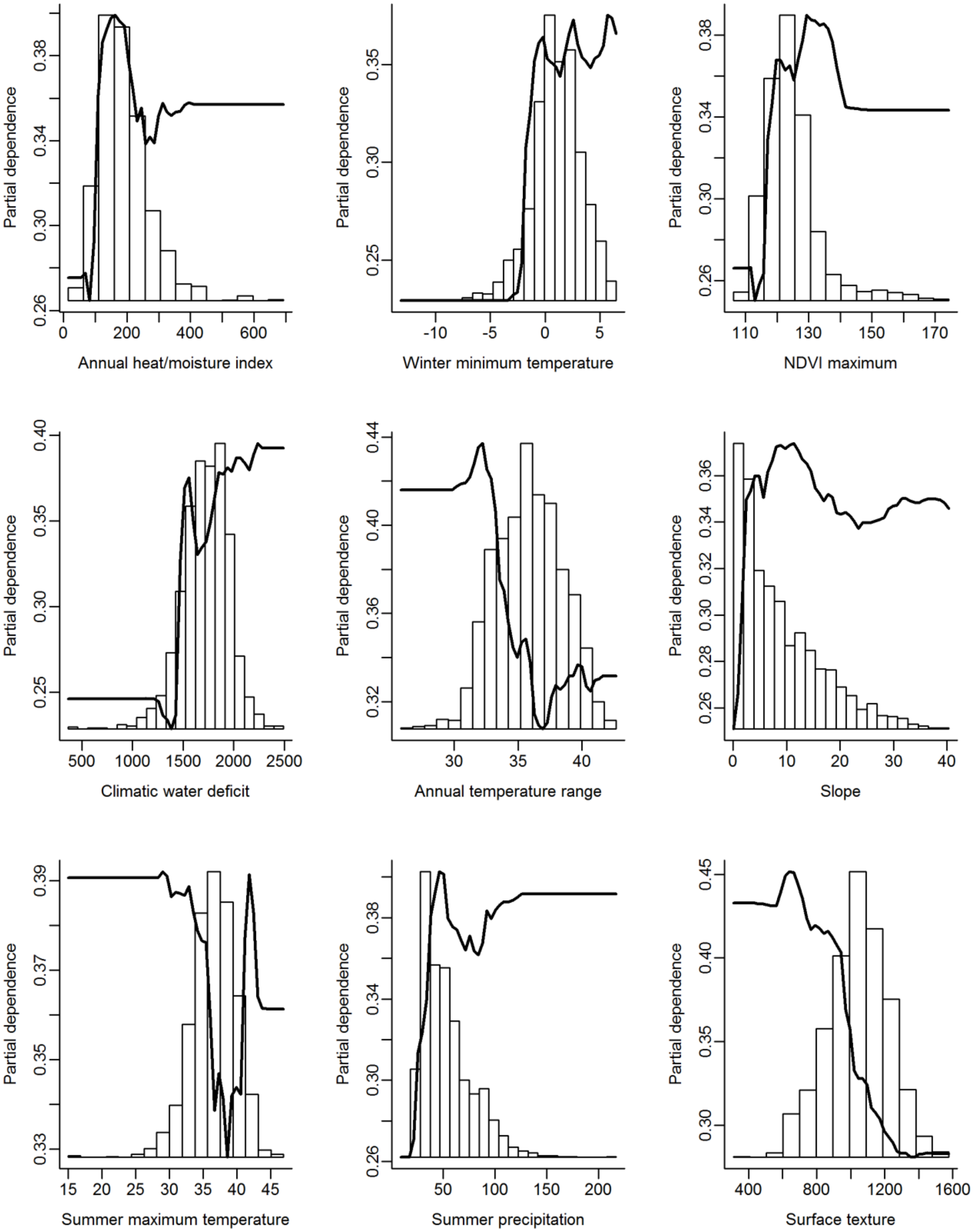

Random Forest response curves for *Krameria erecta*

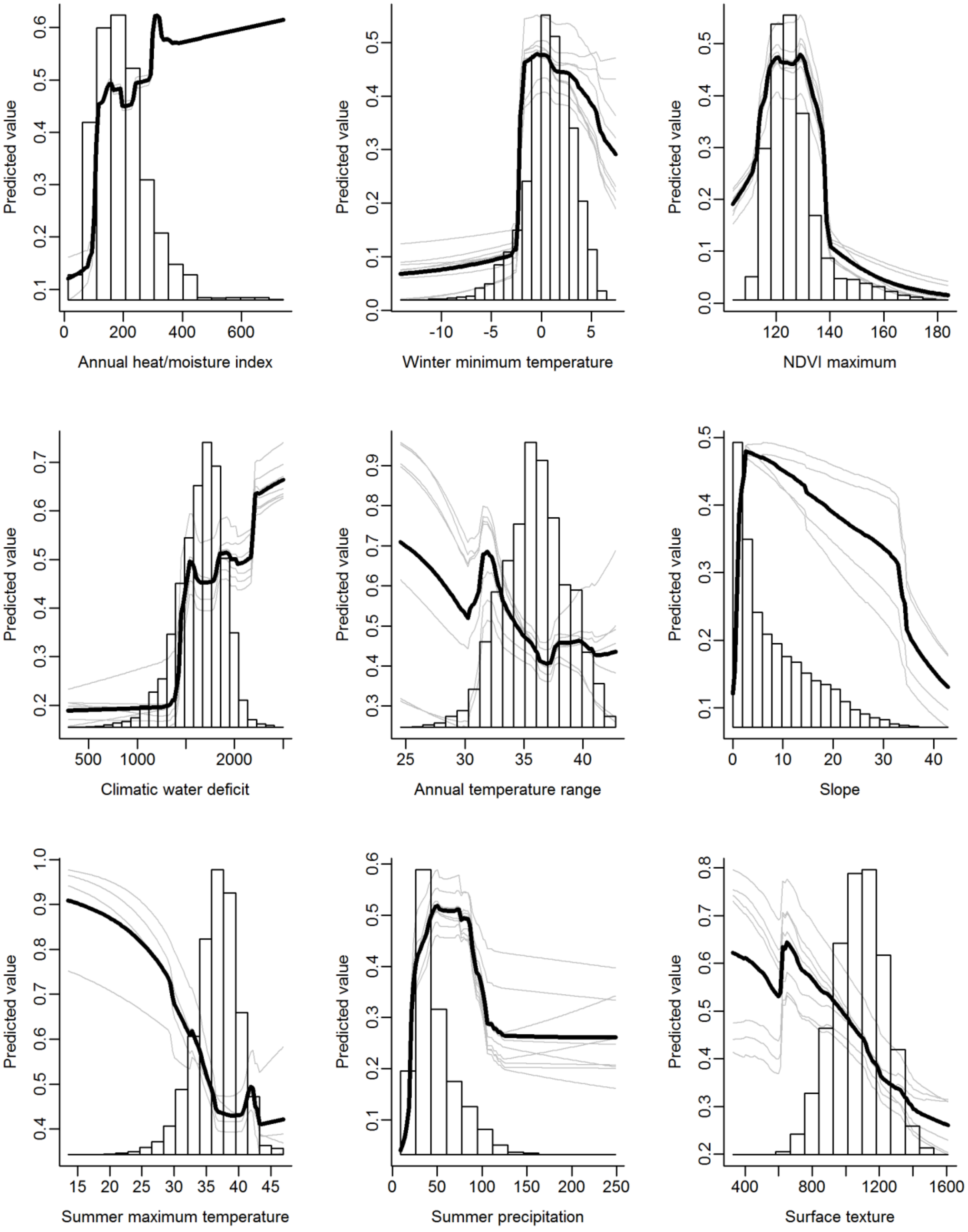

MaxEnt response curves for *Krameria erecta*

27. *Larrea tridentata* SDM and response curves (GAM, Random Forest, Maxent)

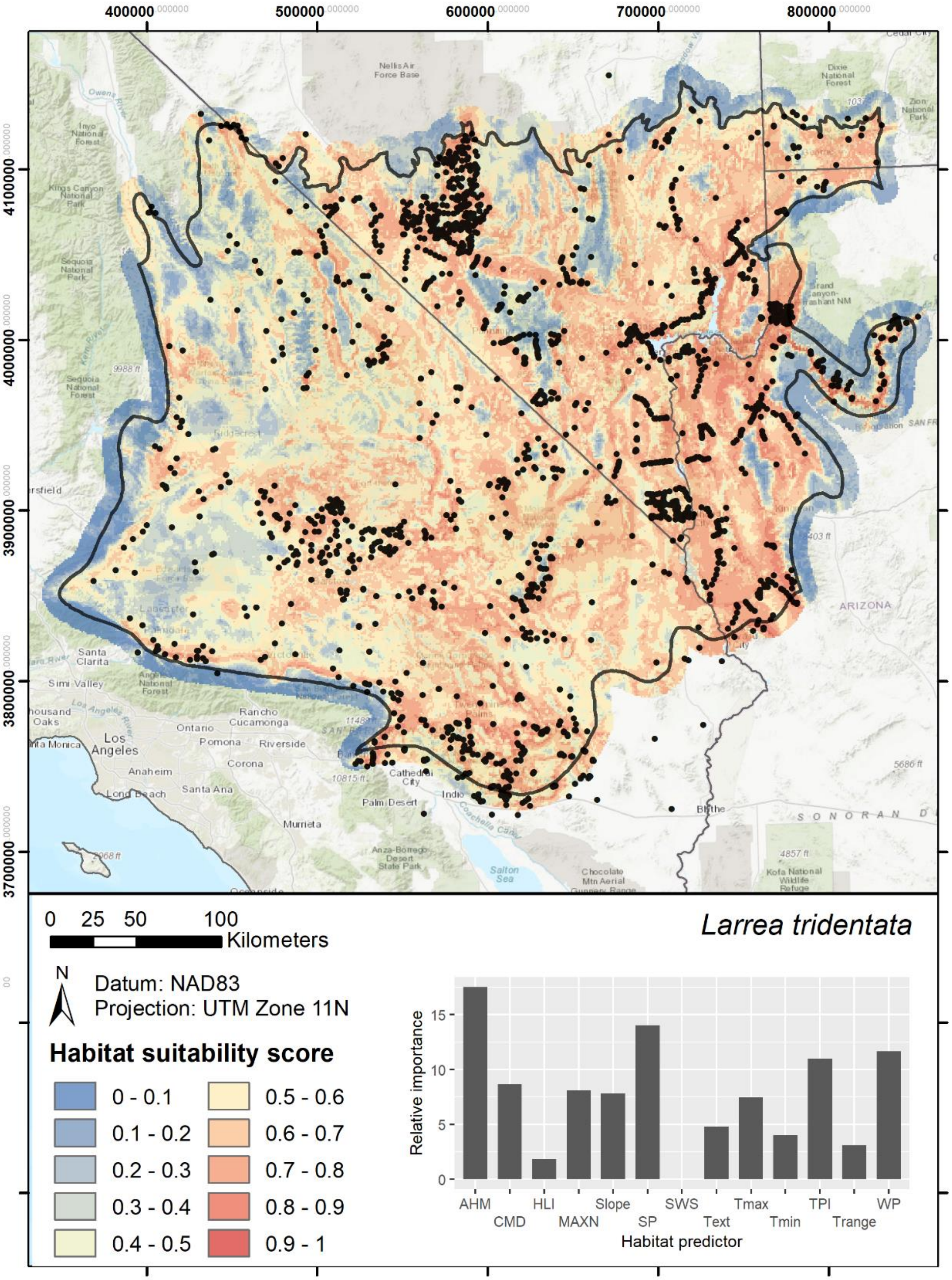

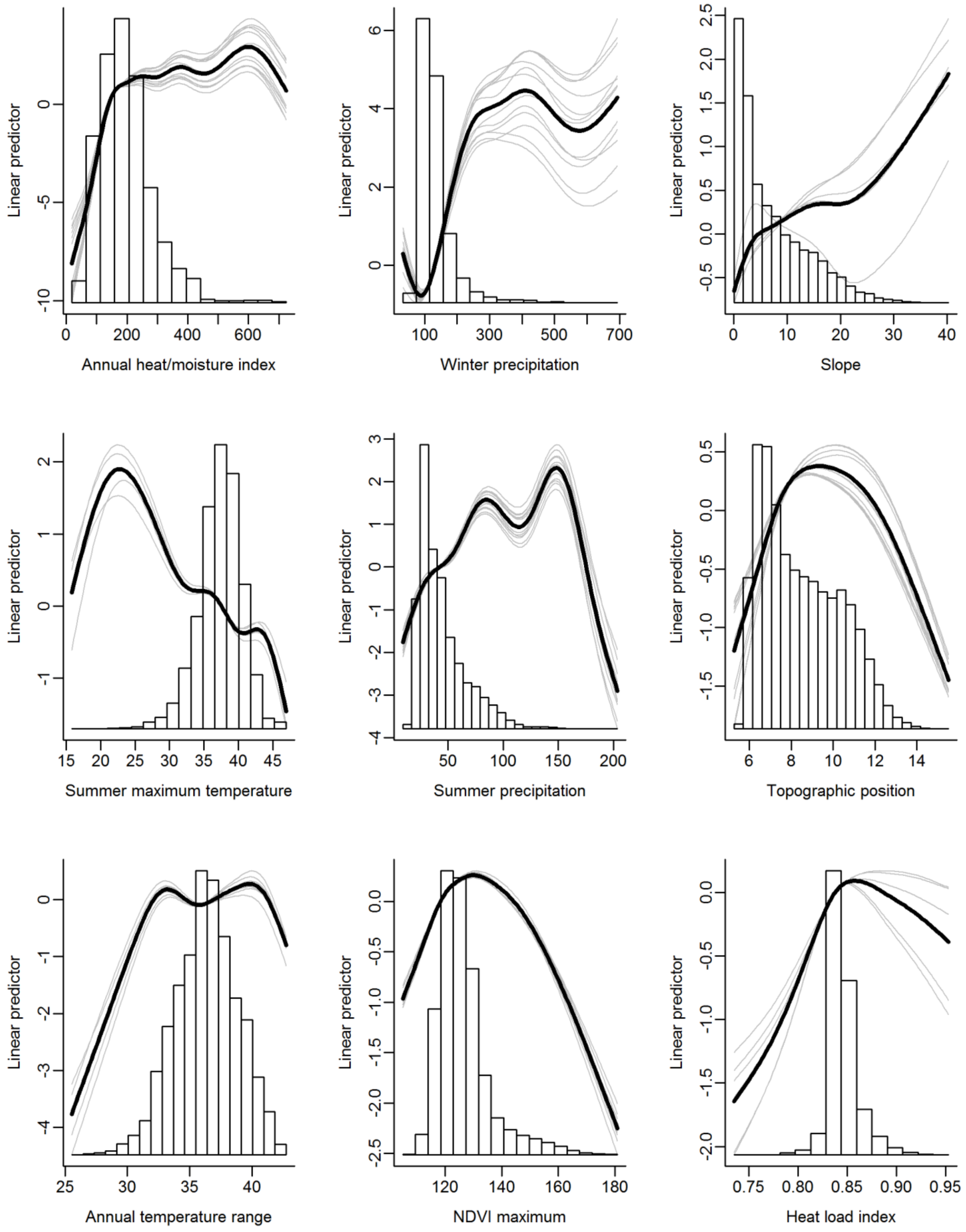

GAM response curves for *Larrea tridentata*

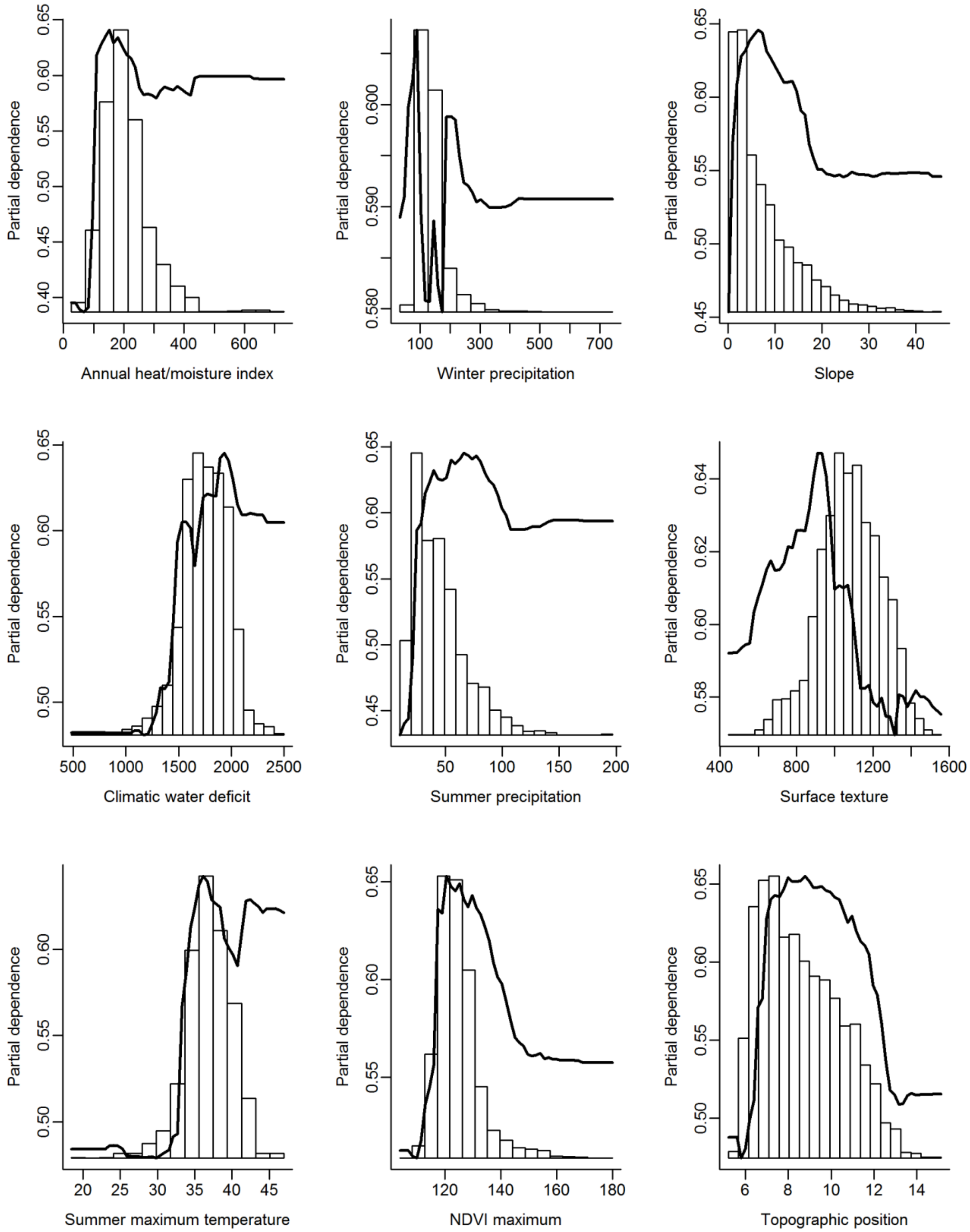

Random Forest response curves for *Larrea tridentata*

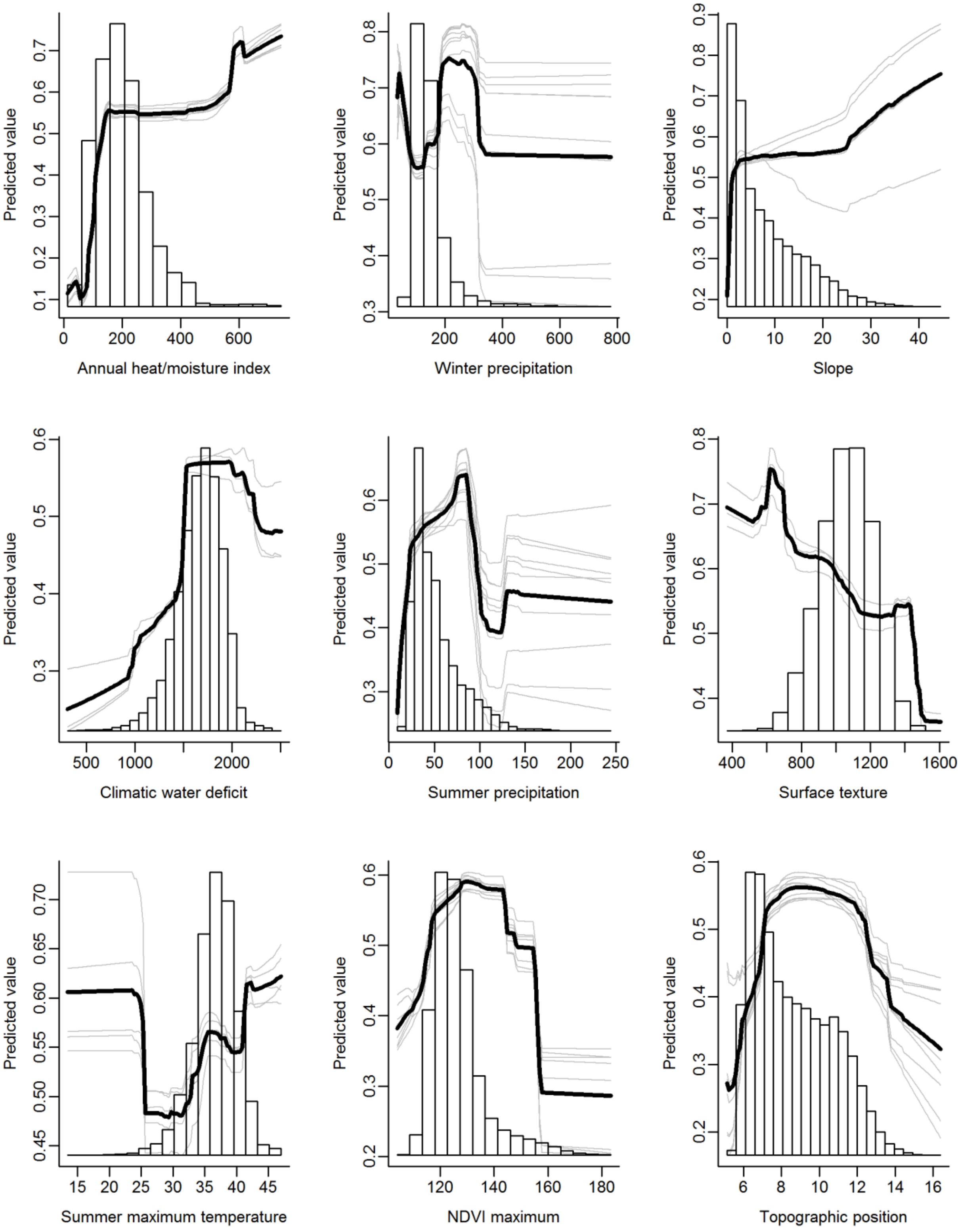

MaxEnt response curves for *Larrea tridentata*

28. *Lepidium lasiocarpum* SDM and response curves (GAM, Random Forest, Maxent)

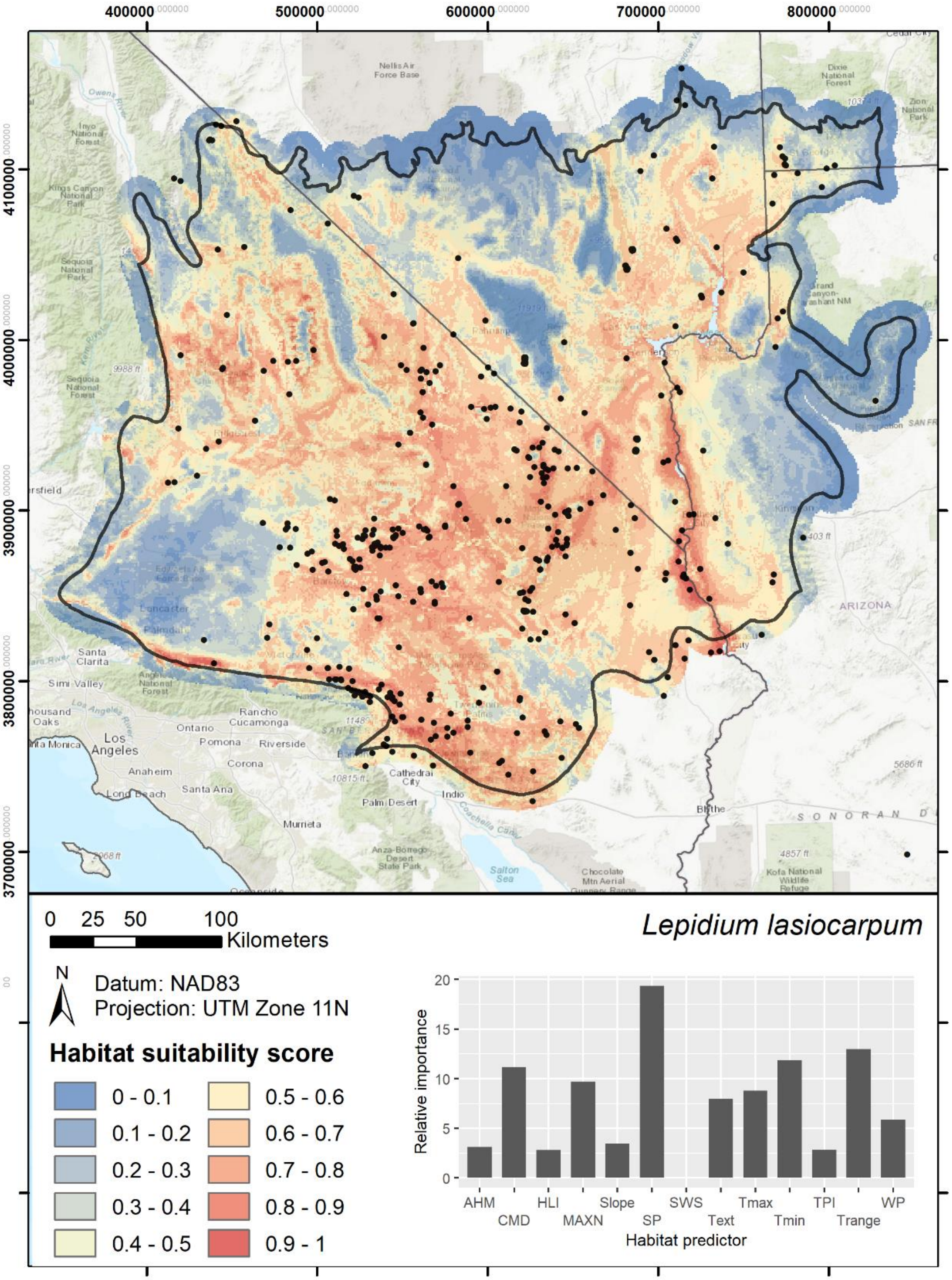

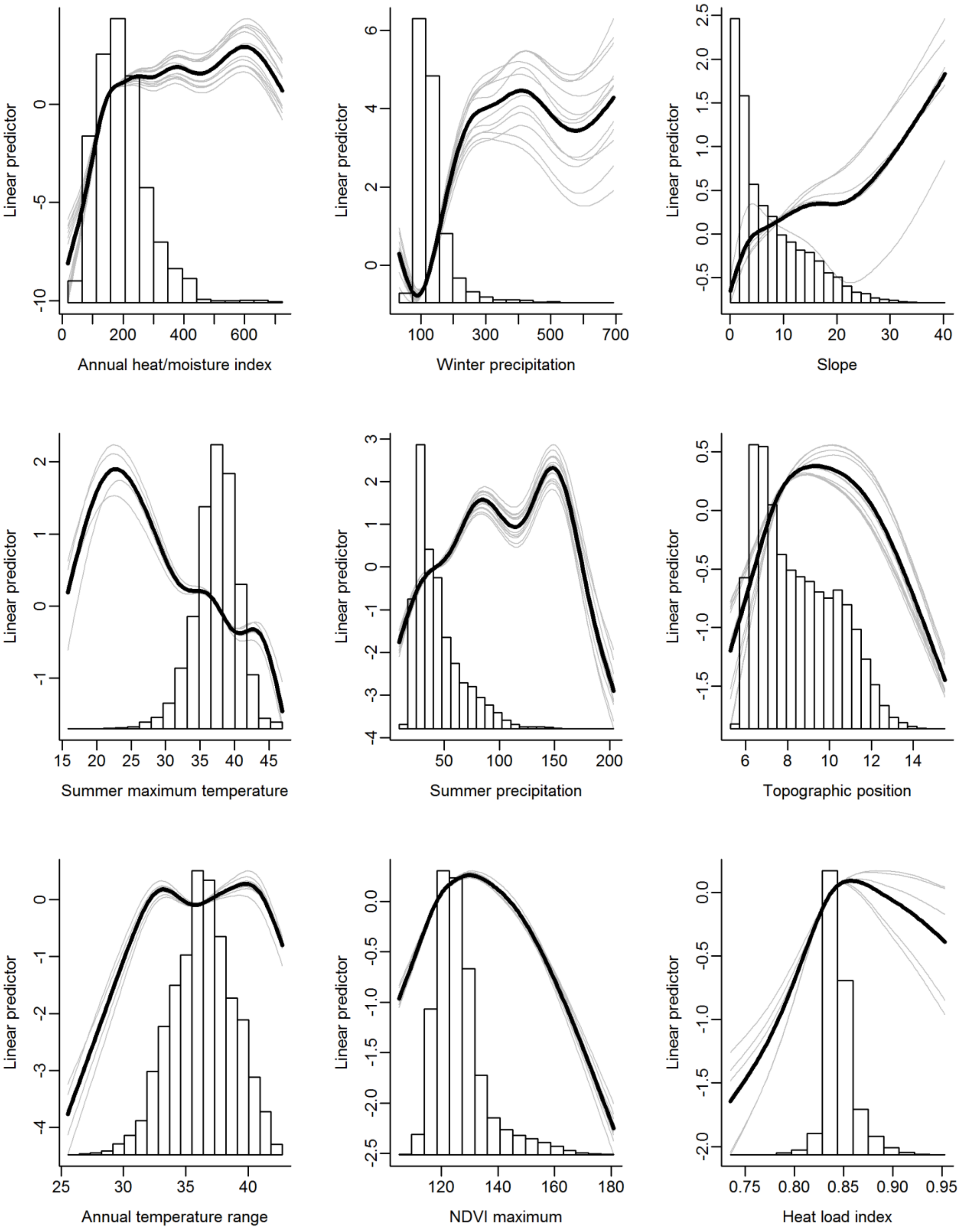

GAM response curves for *Lepidium lasiocarpum*

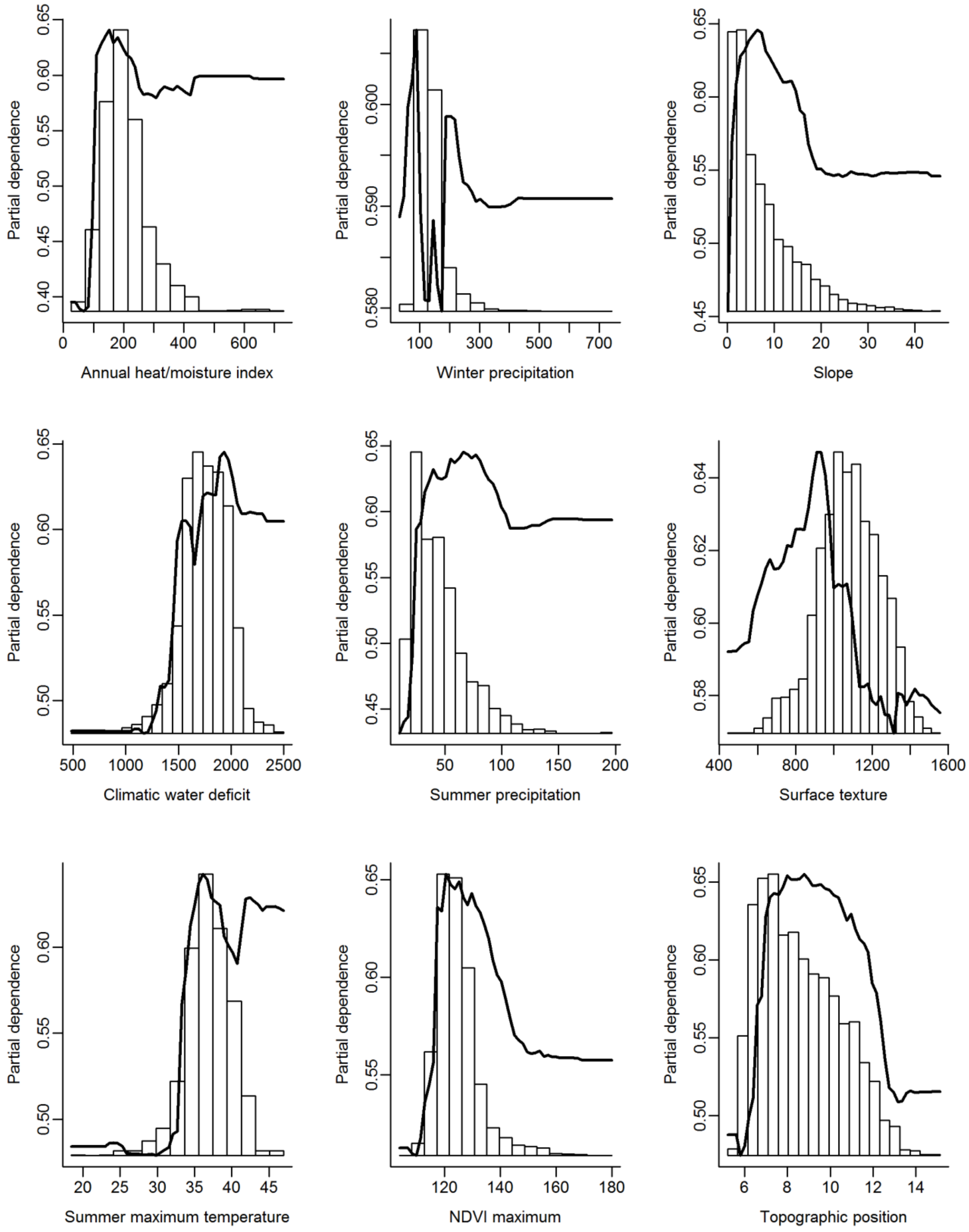

Random Forest response curves for *Lepidium lasiocarpum*

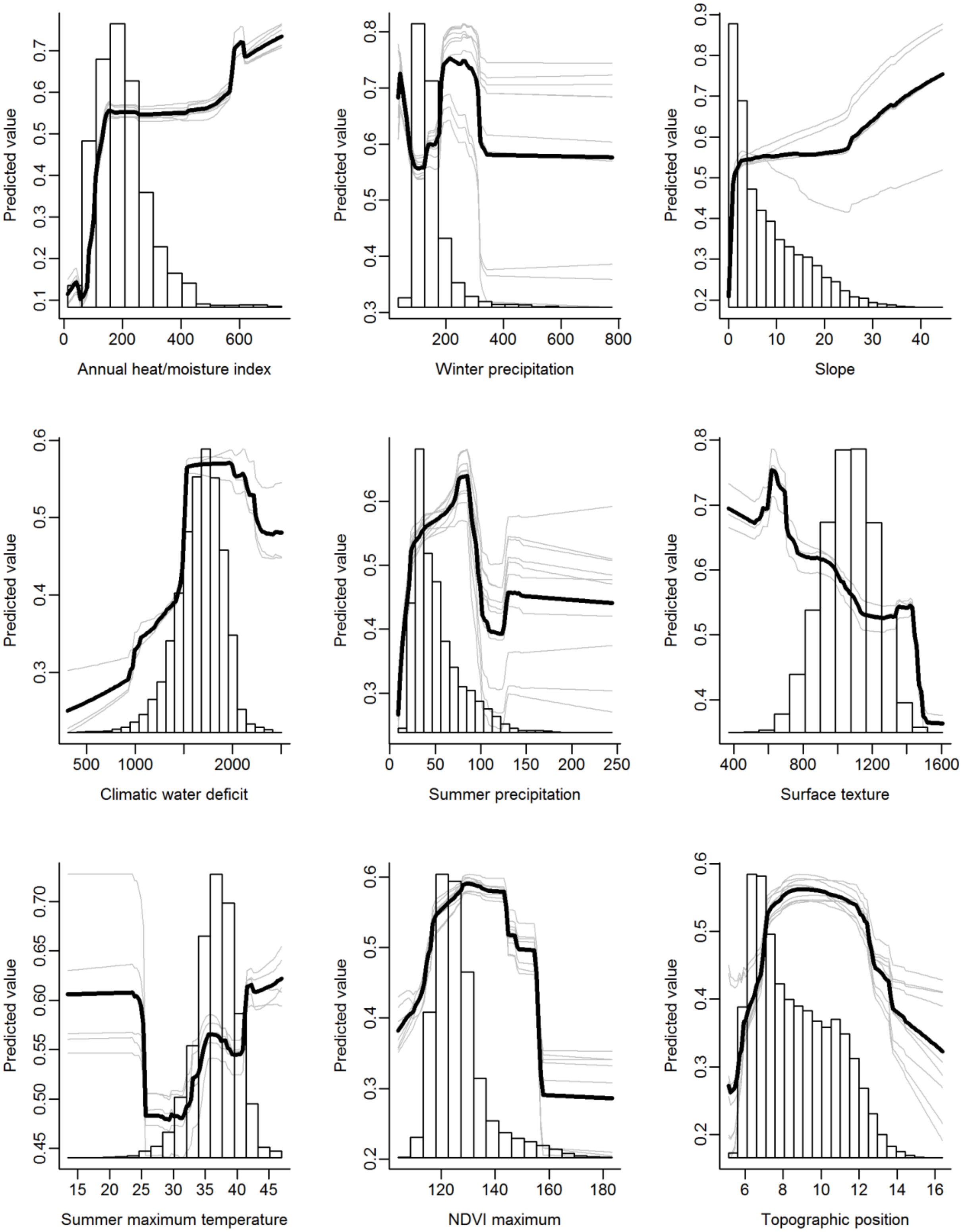

MaxEnt response curves for *Lepidium lasiocarpum*

29. *Lupinus odoratus* SDM and response curves (Random Forest)

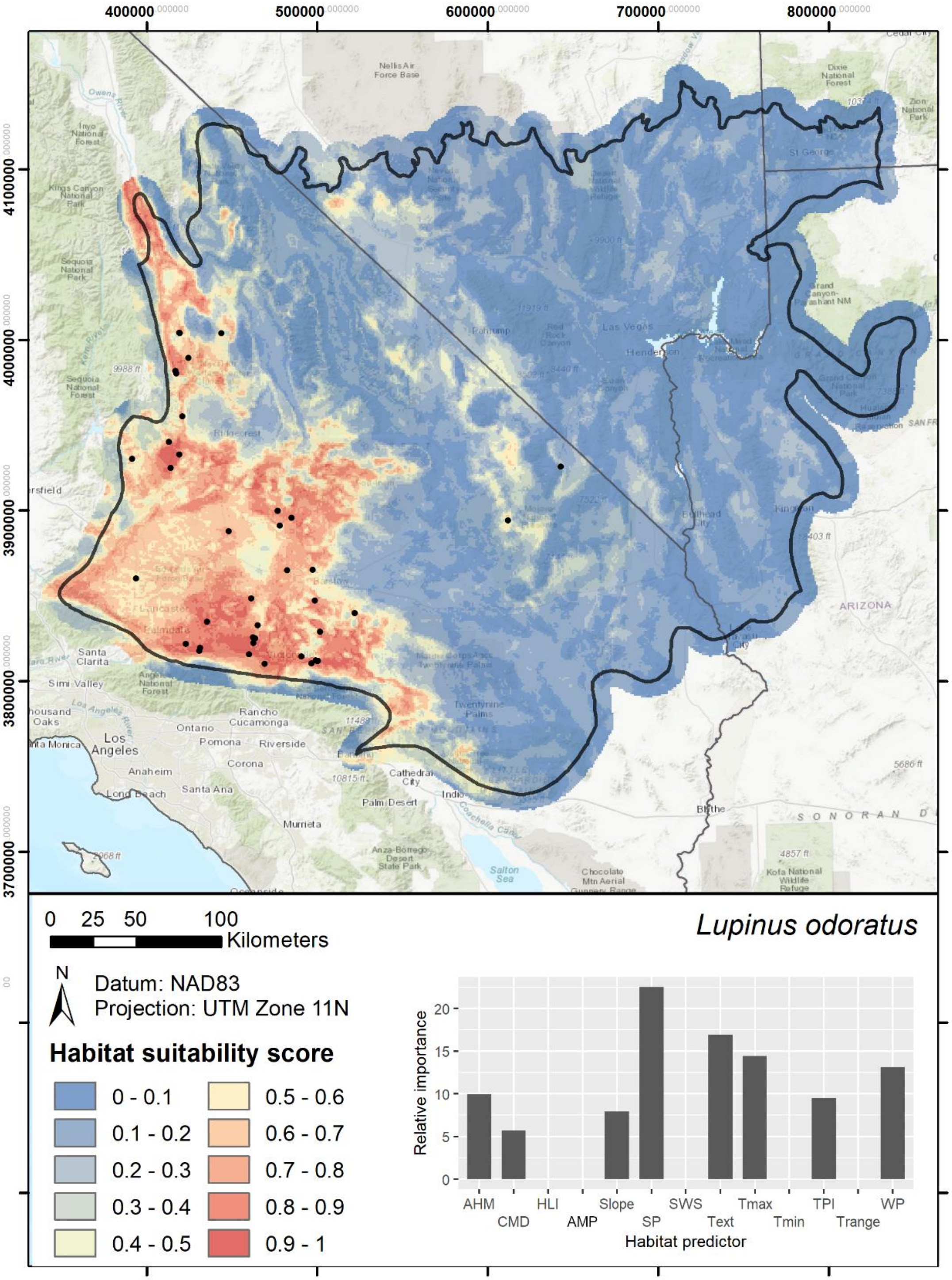

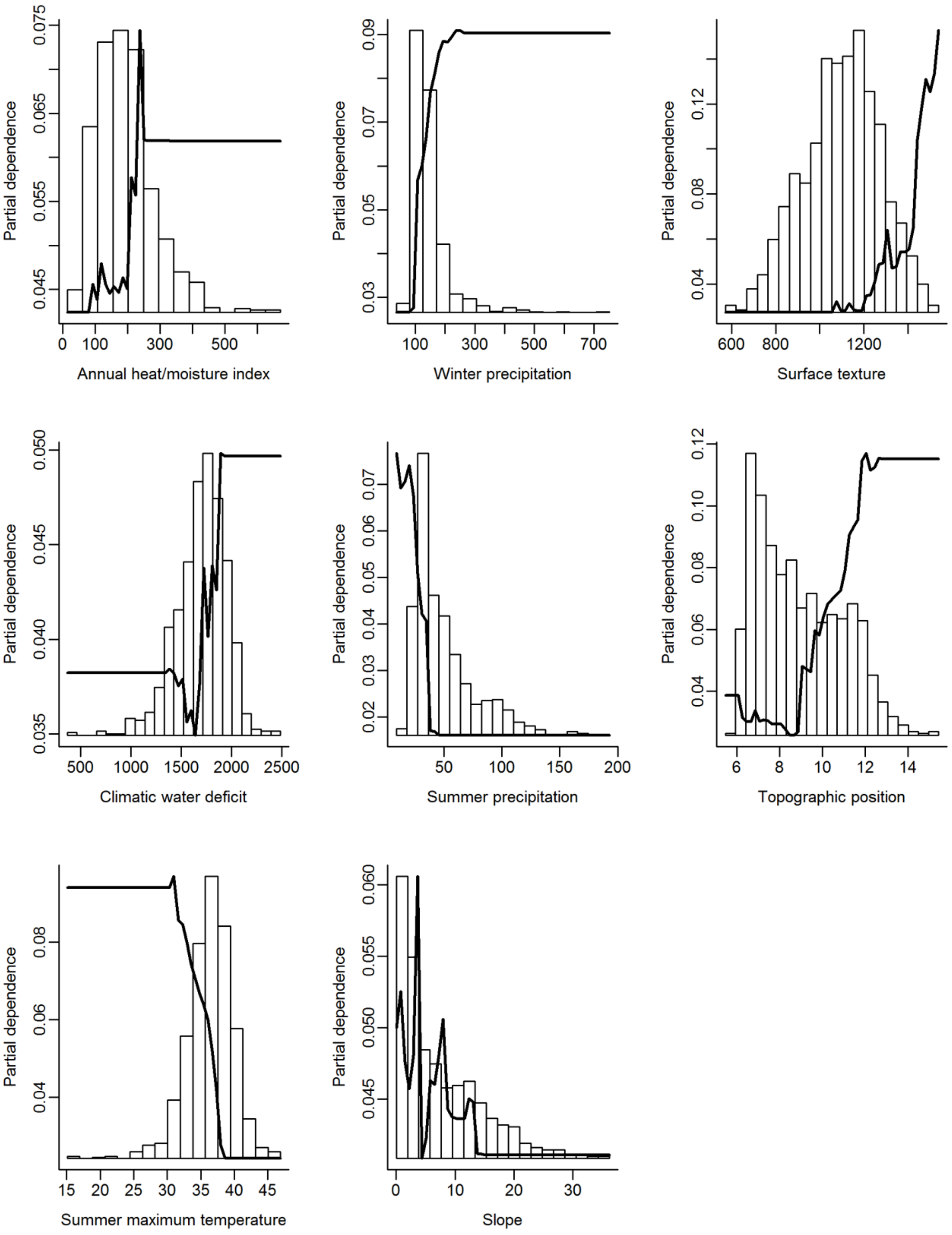

Random Forest response curves for *Lupinus odoratus*

30. *Lycium andersonii* SDM and response curves (GAM, Random Forest, Maxent)

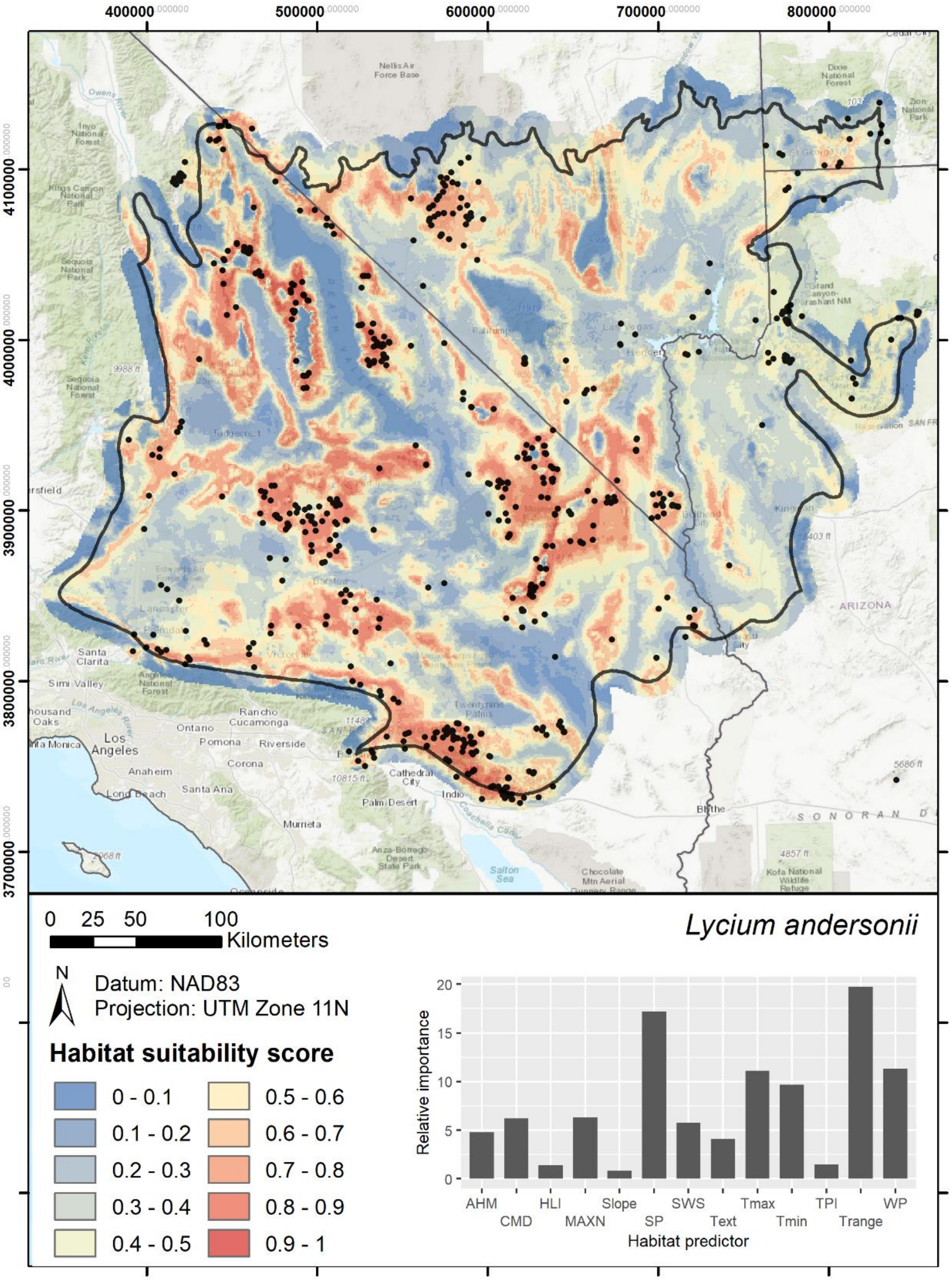

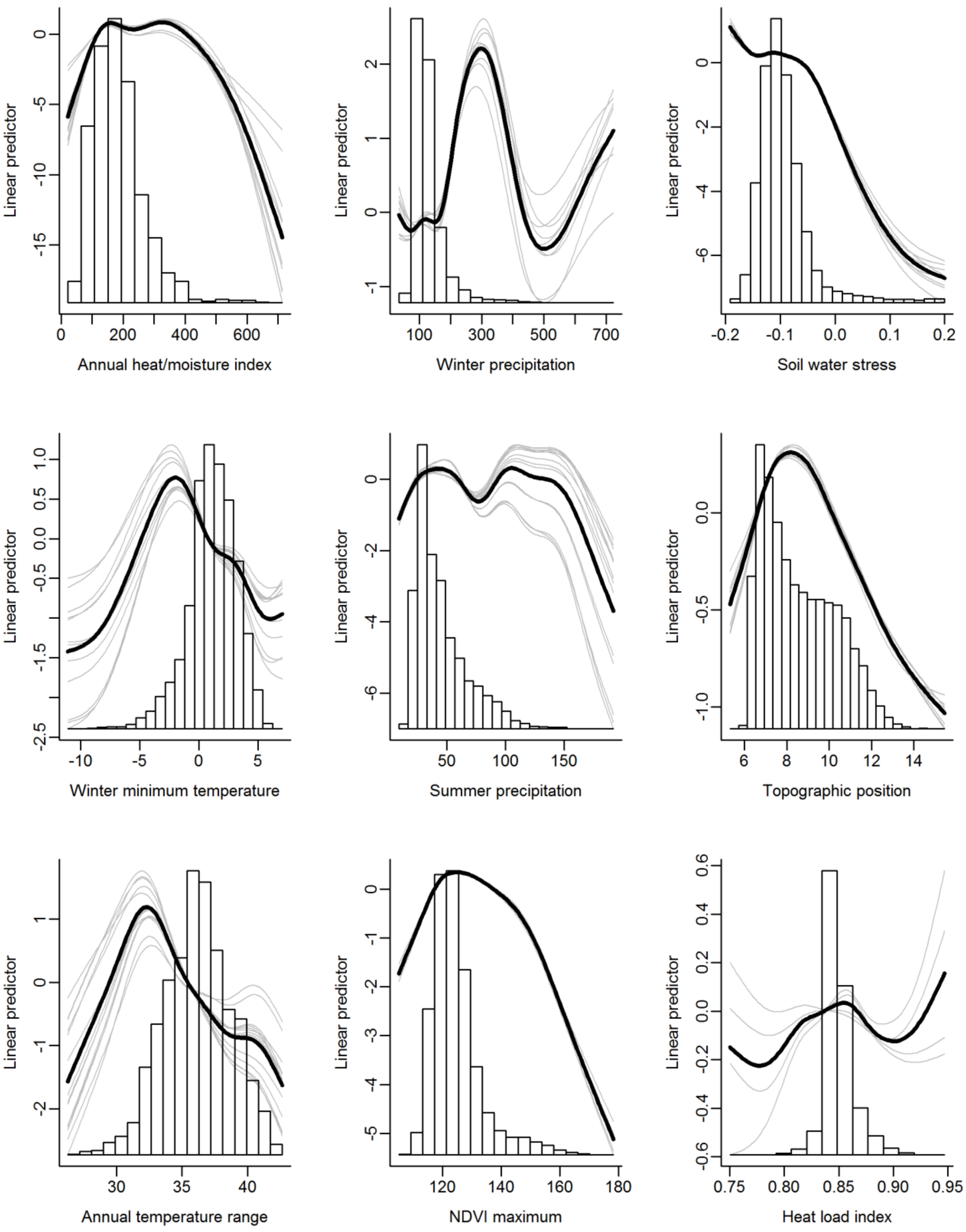

GAM response curves for *Lycium andersonii*

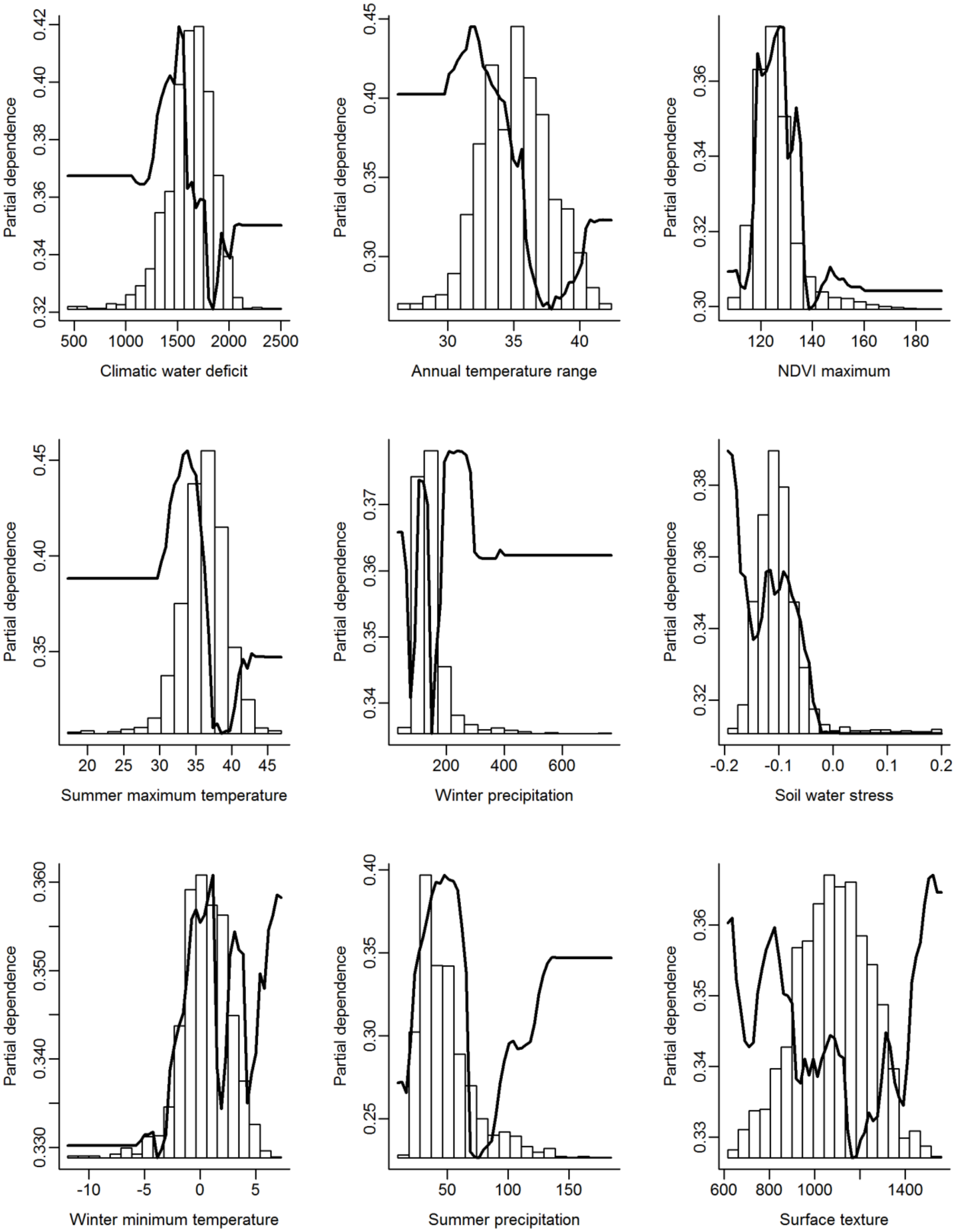

Random Forest response curves for *Lycium andersonii*

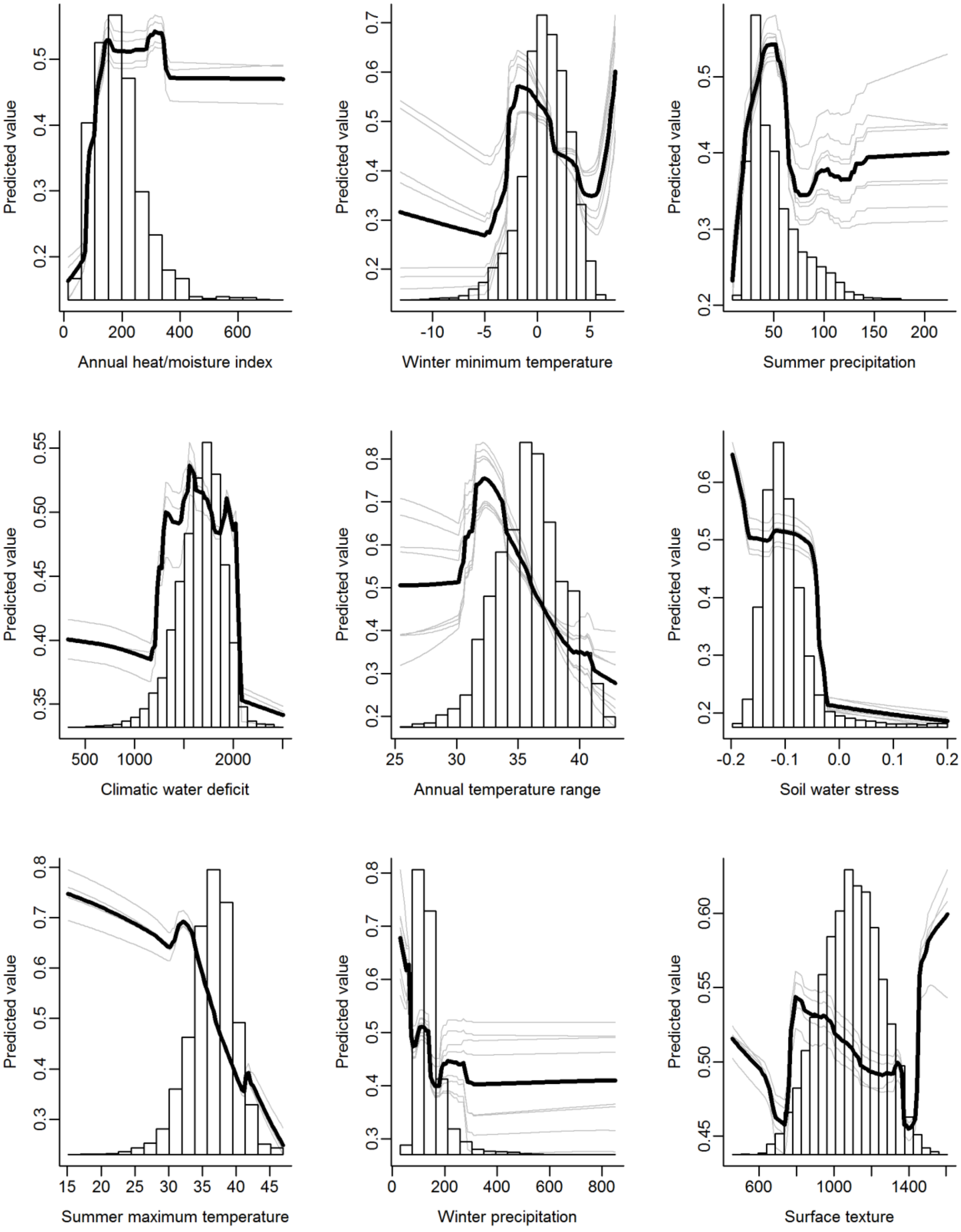

MaxEnt response curves for *Lycium andersonii*

31. *Lycium cooperi* SDM and response curves (GAM, Random Forest, Maxent)

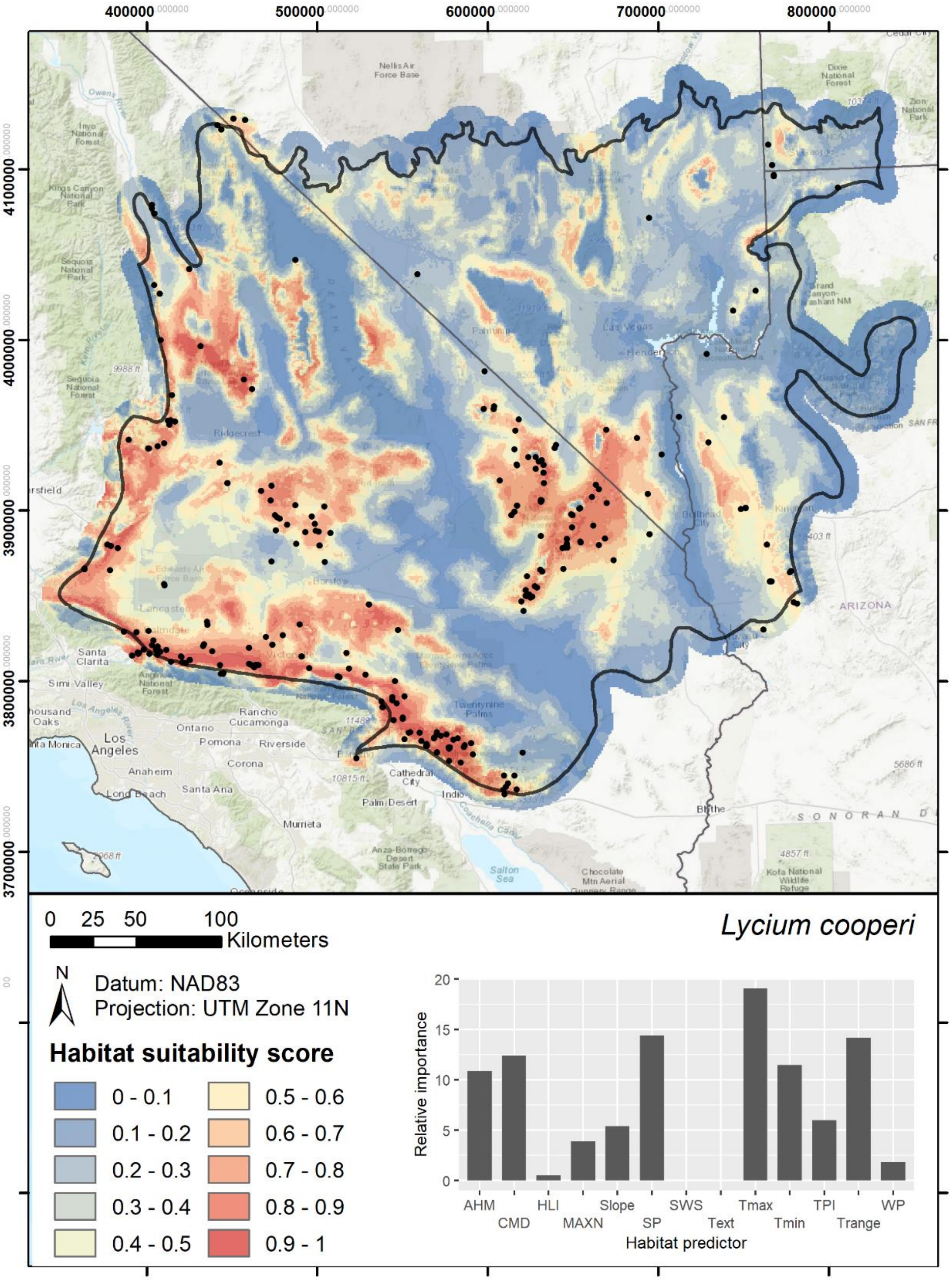

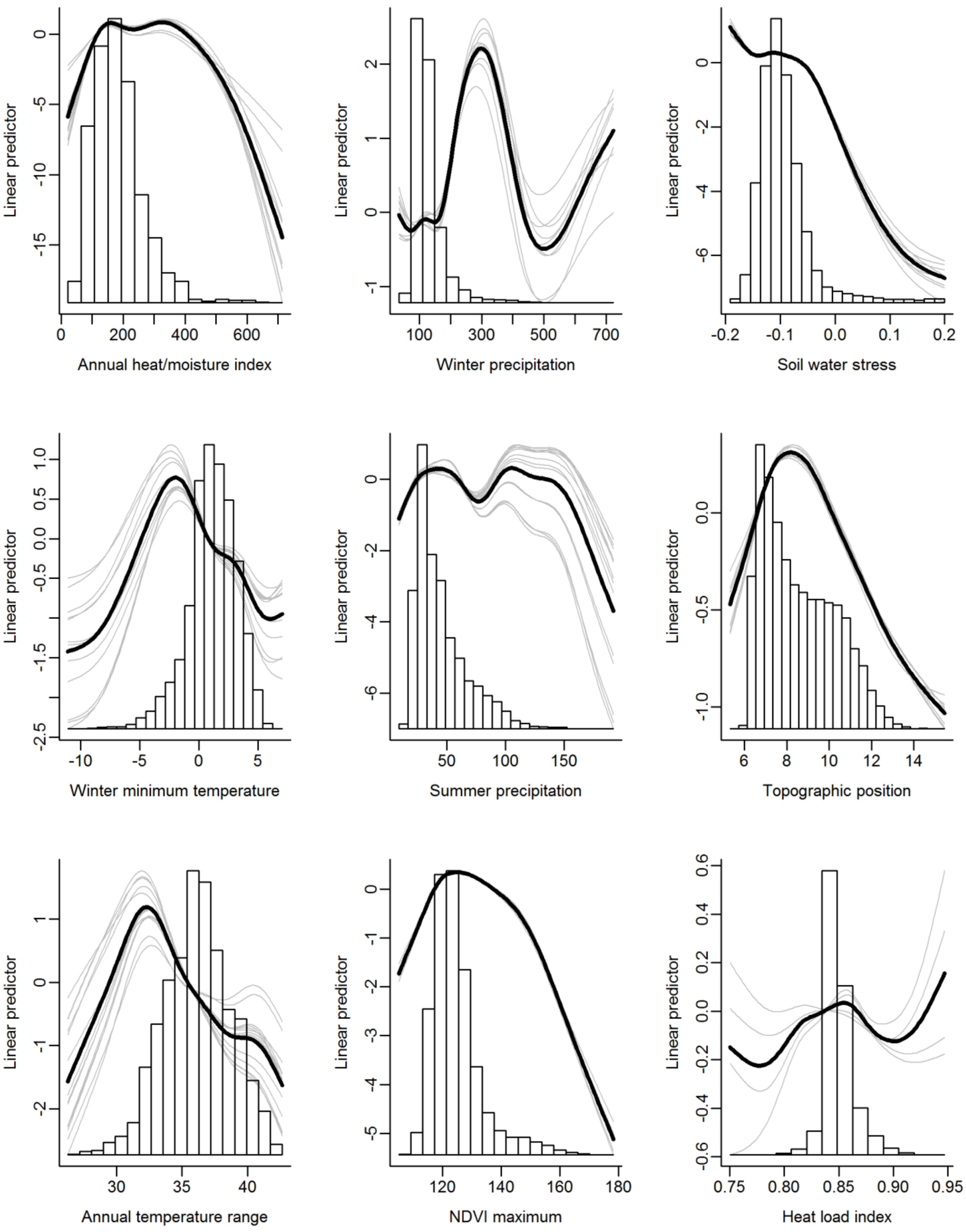

GAM response curves for *Lycium cooperi*

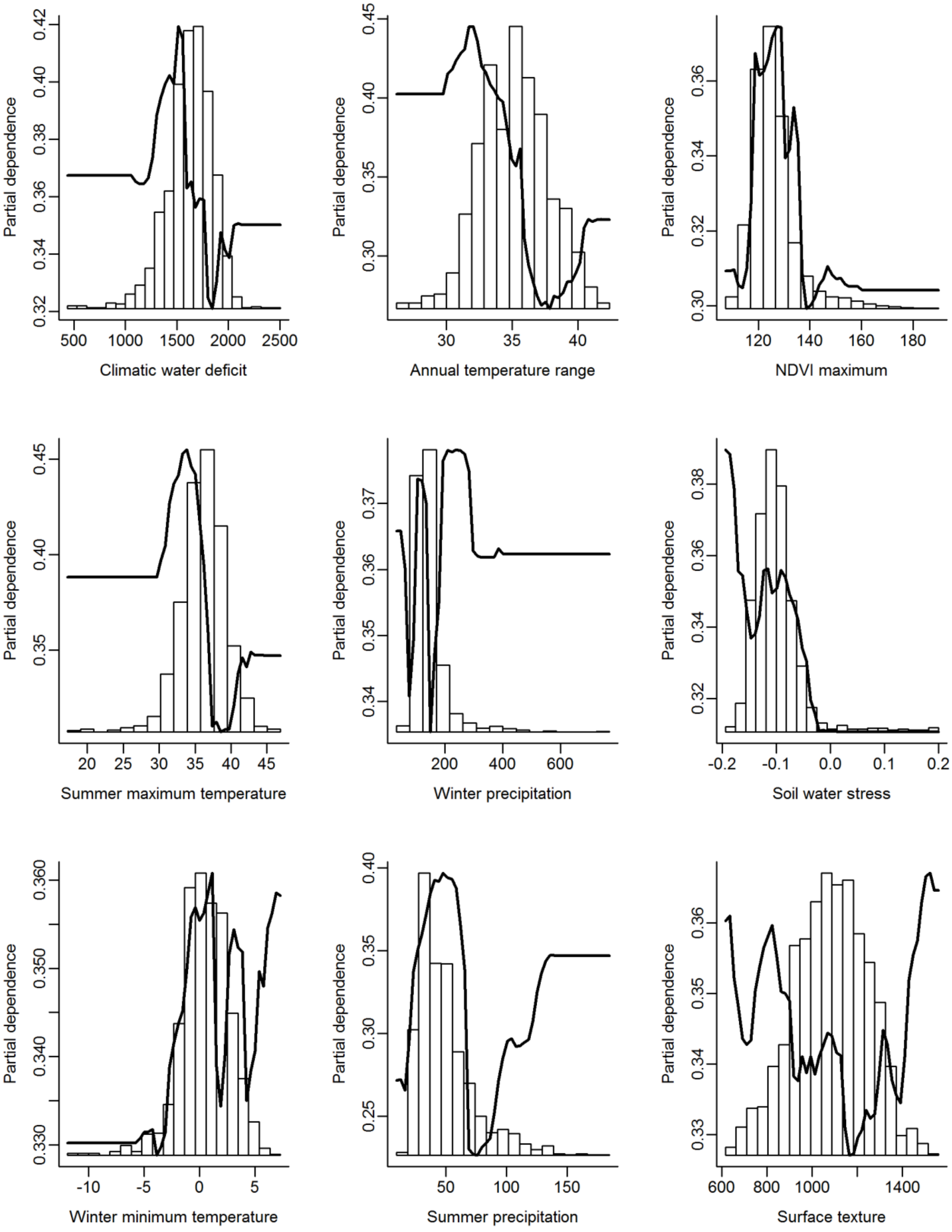

Random Forest response curves for *Lycium cooperi*

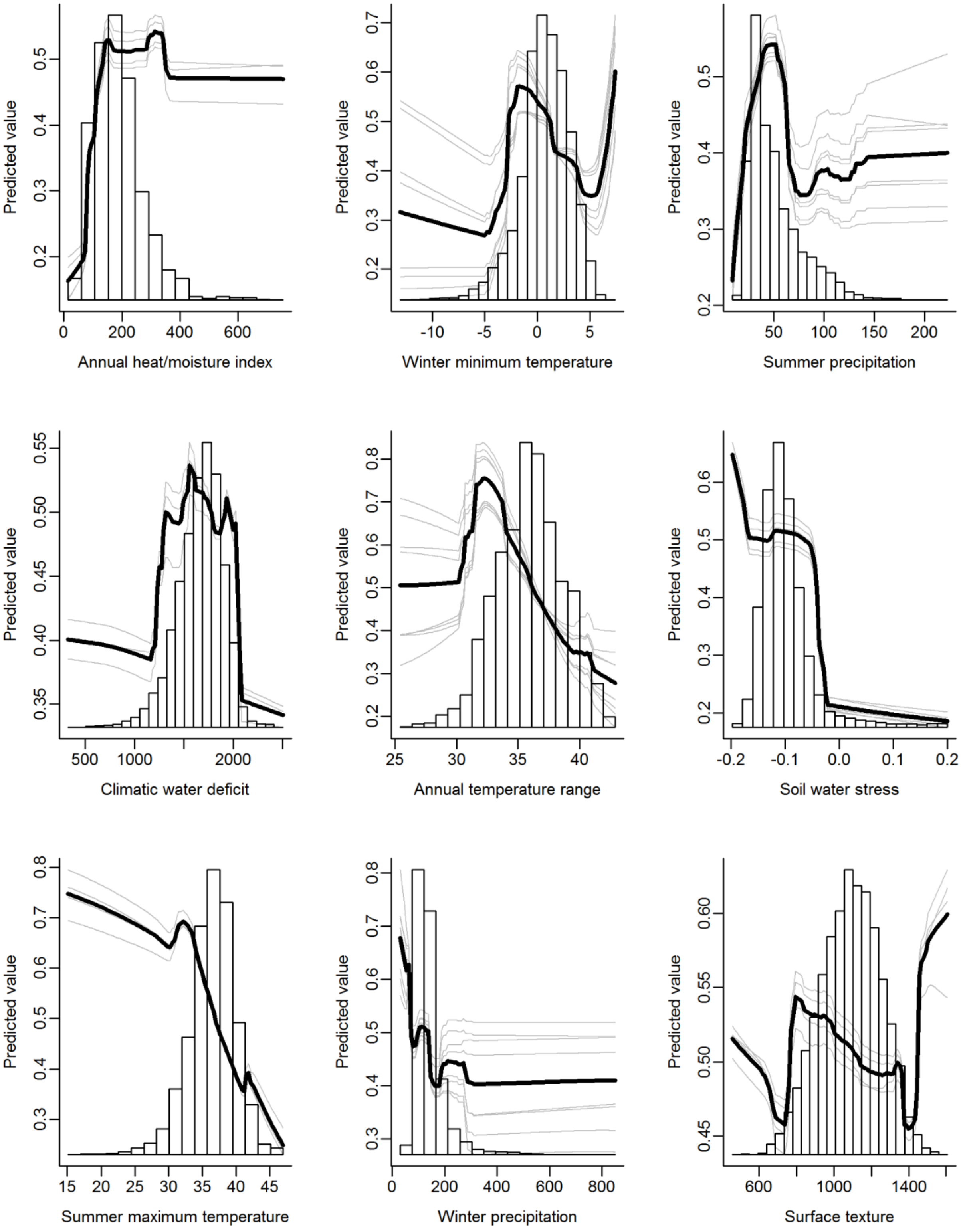

MaxEnt response curves for *Lycium cooperi*

32. *Lycium pallidum* SDM and response curves (GAM, Random Forest, Maxent)

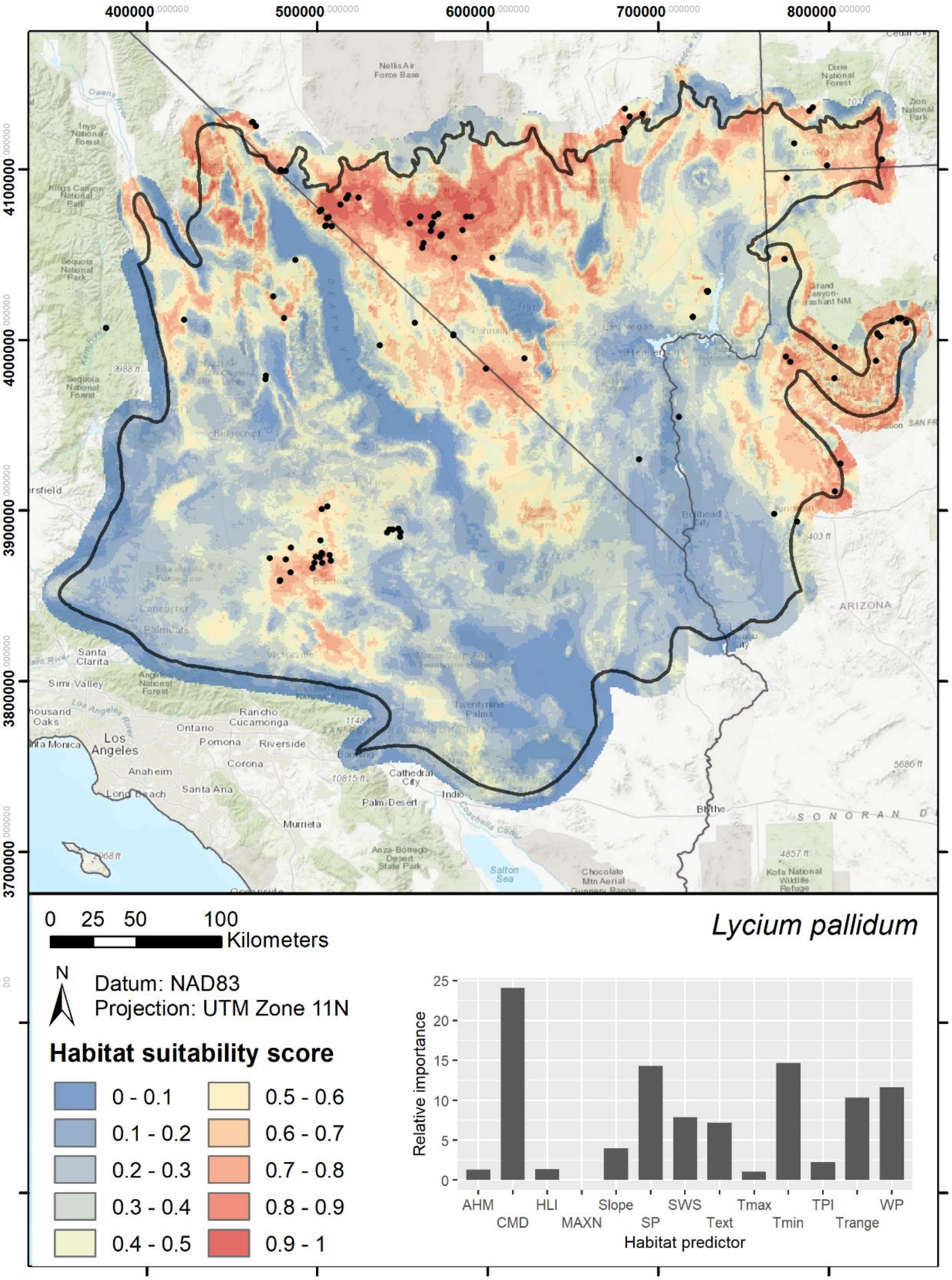

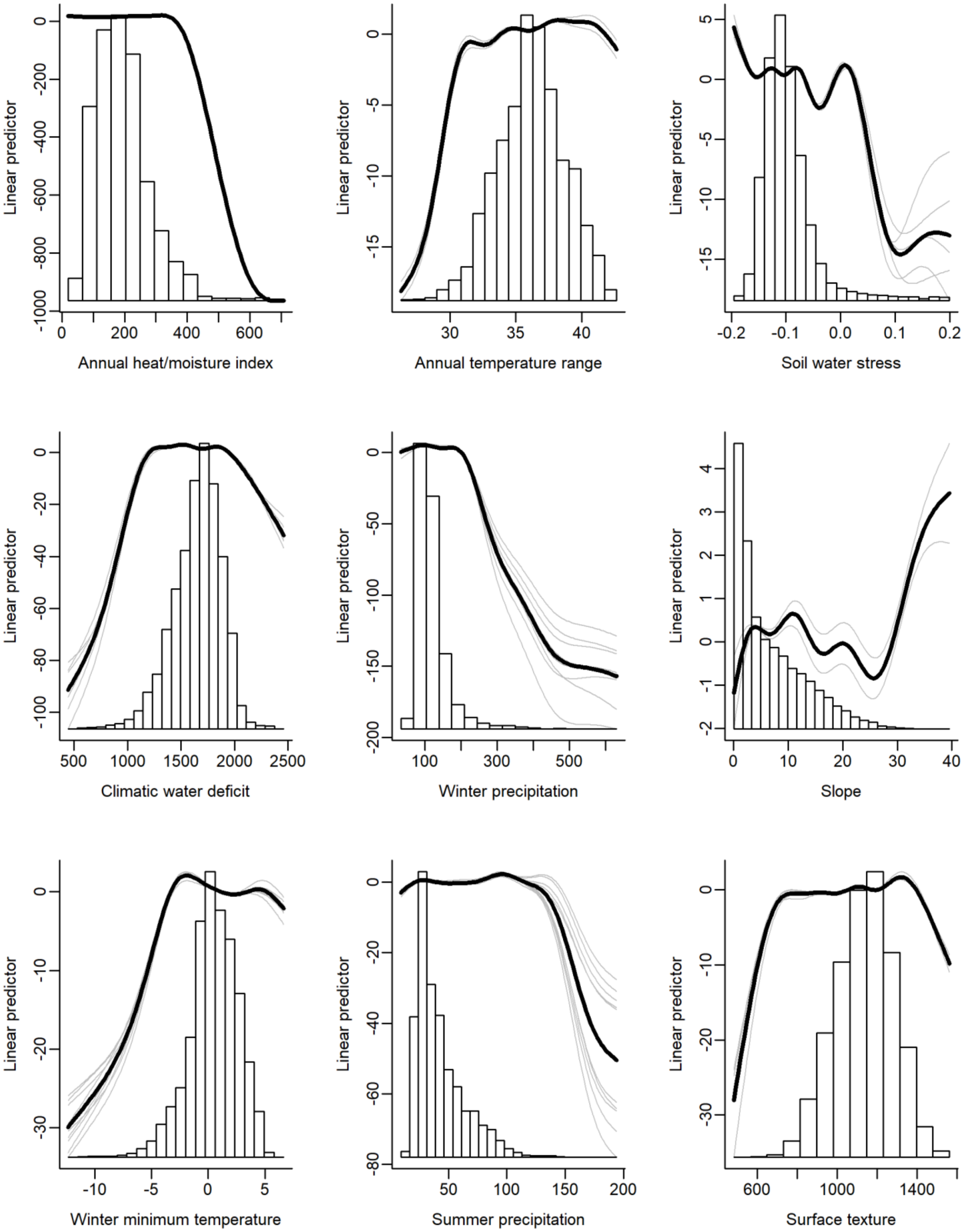

GAM response curves for *Lycium pallidum*

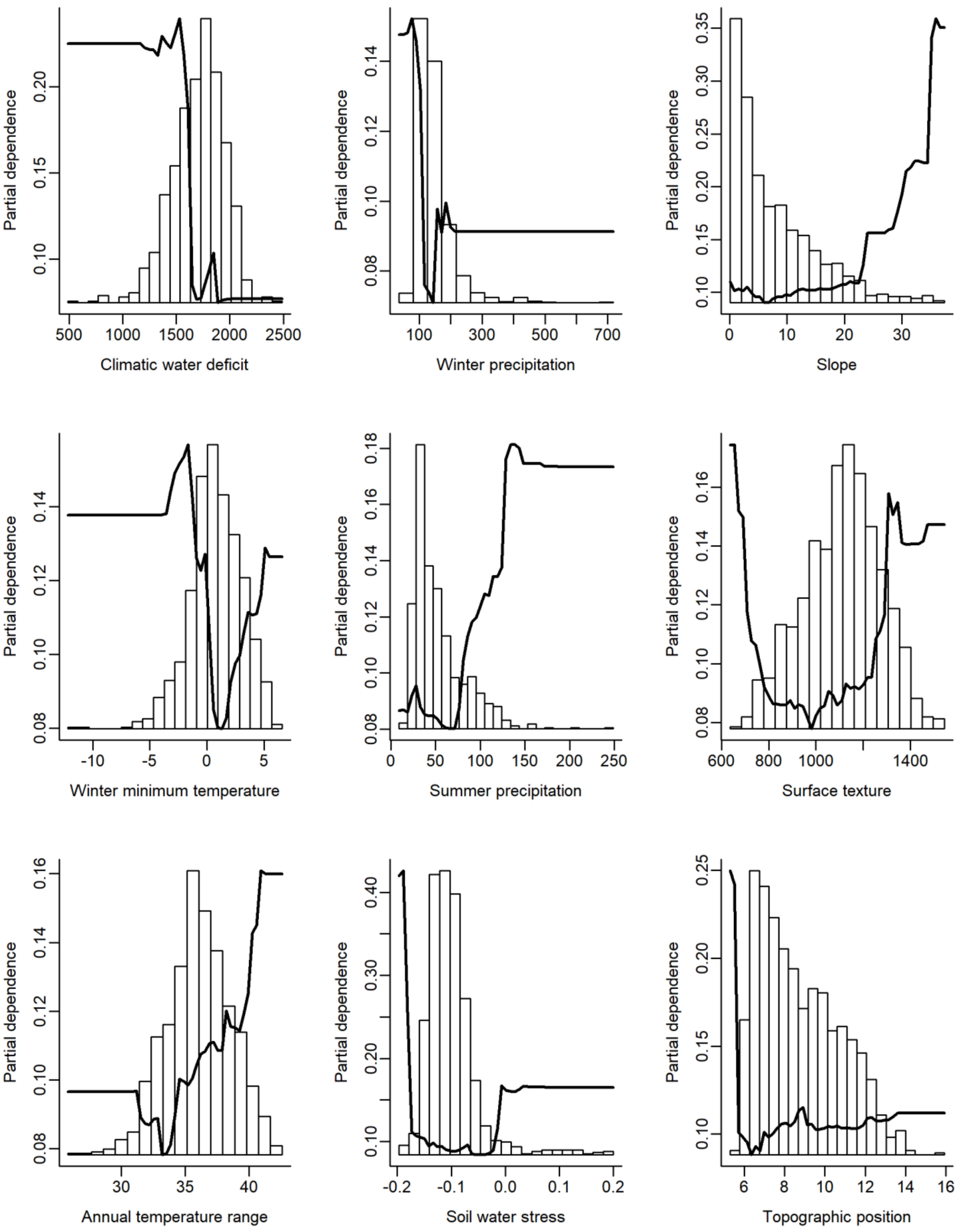

Random Forest response curves for *Lycium pallidum*

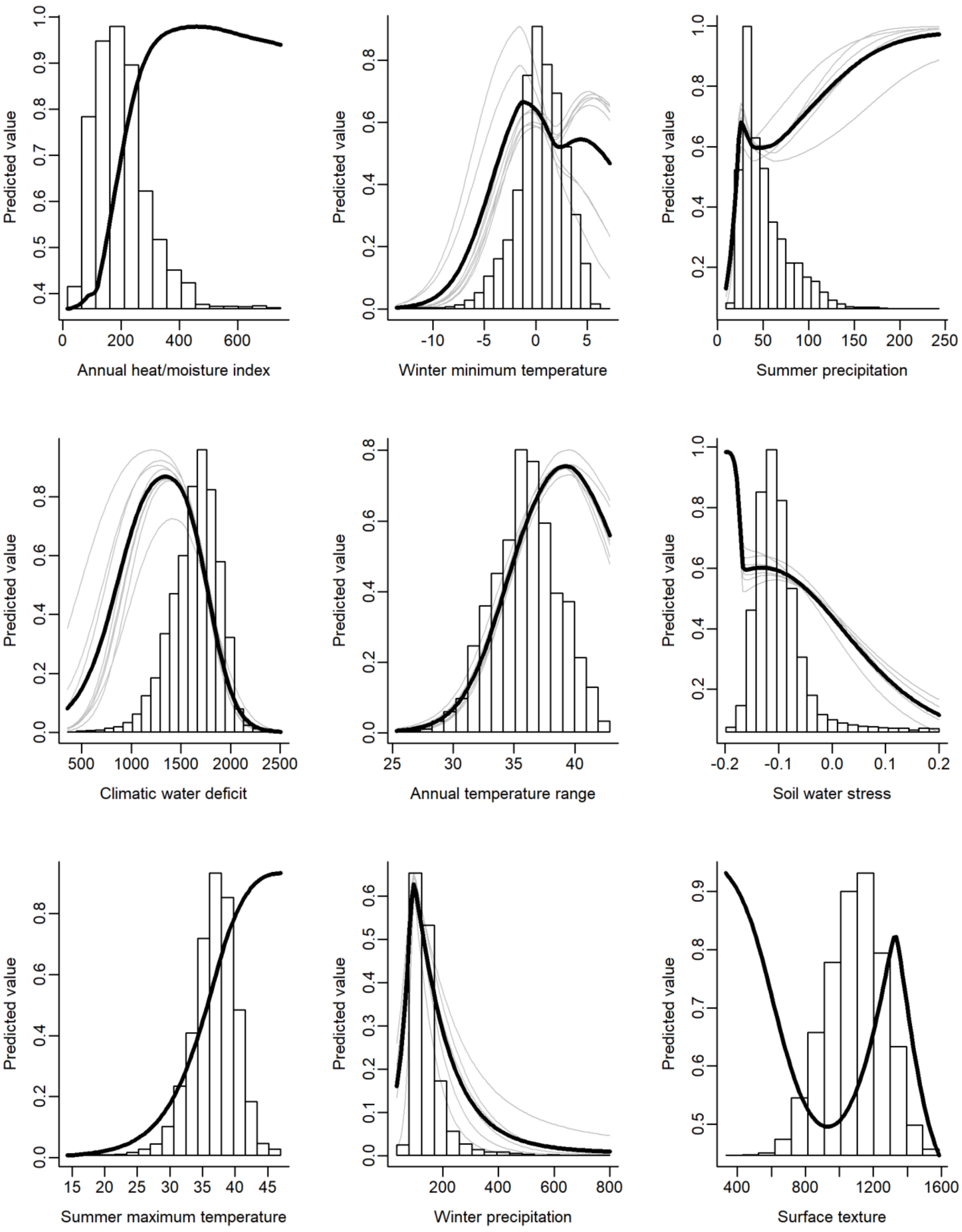

MaxEnt response curves for *Lycium pallidum*

33. *Malacothrix glabrata* SDM and response curves (GAM, Random Forest, Maxent)

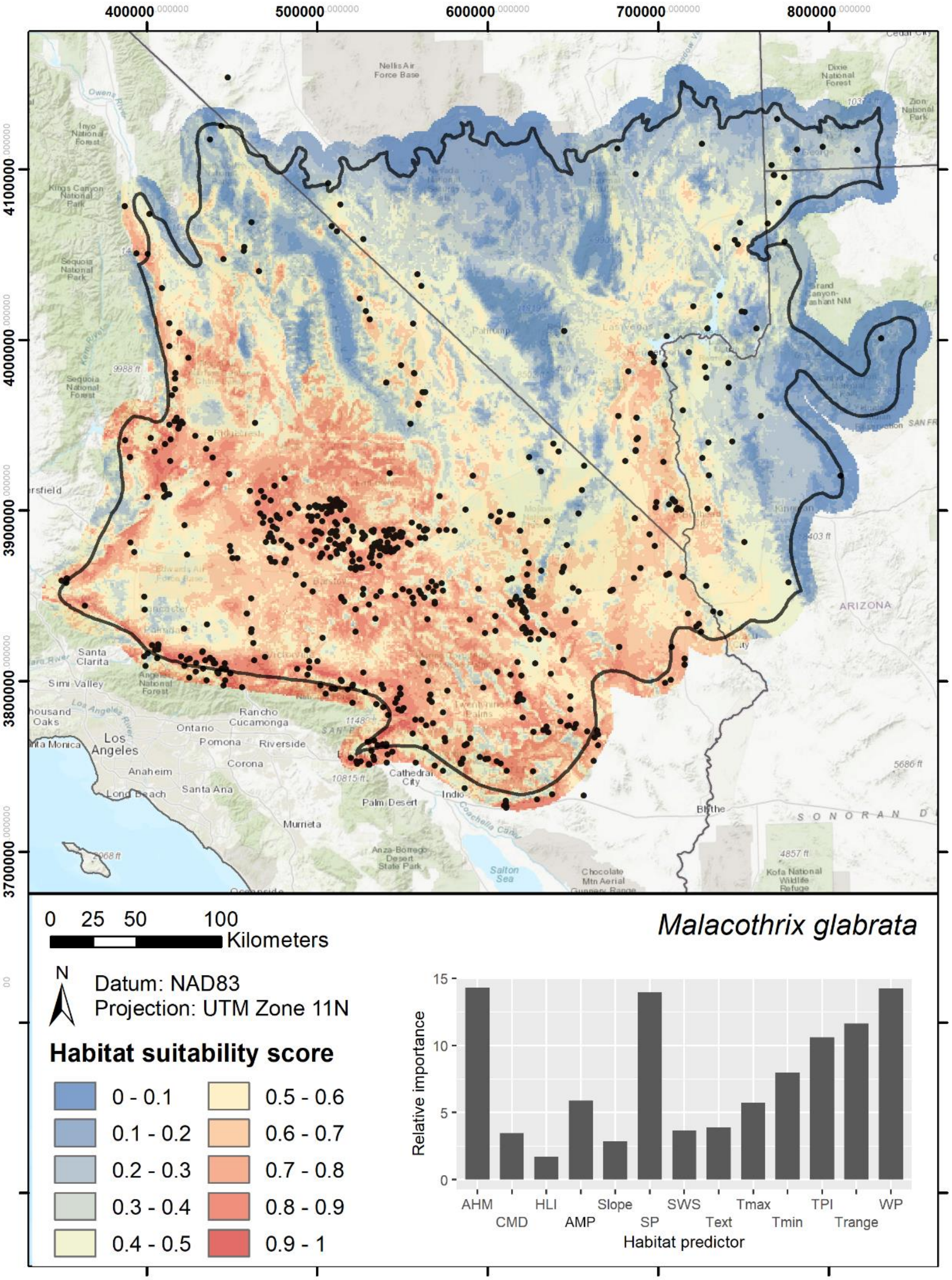

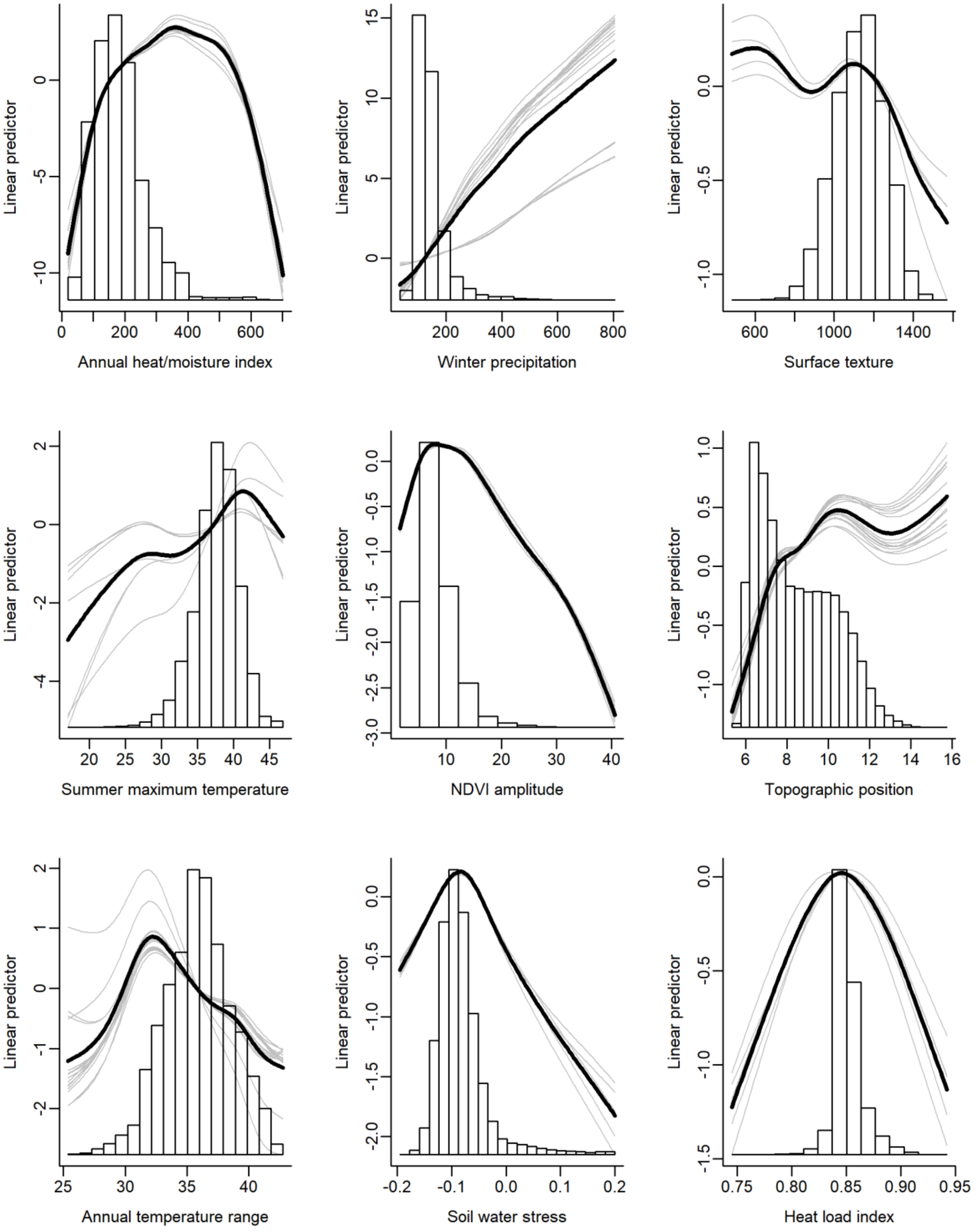

GAM response curves for *Malacothrix glabrata*

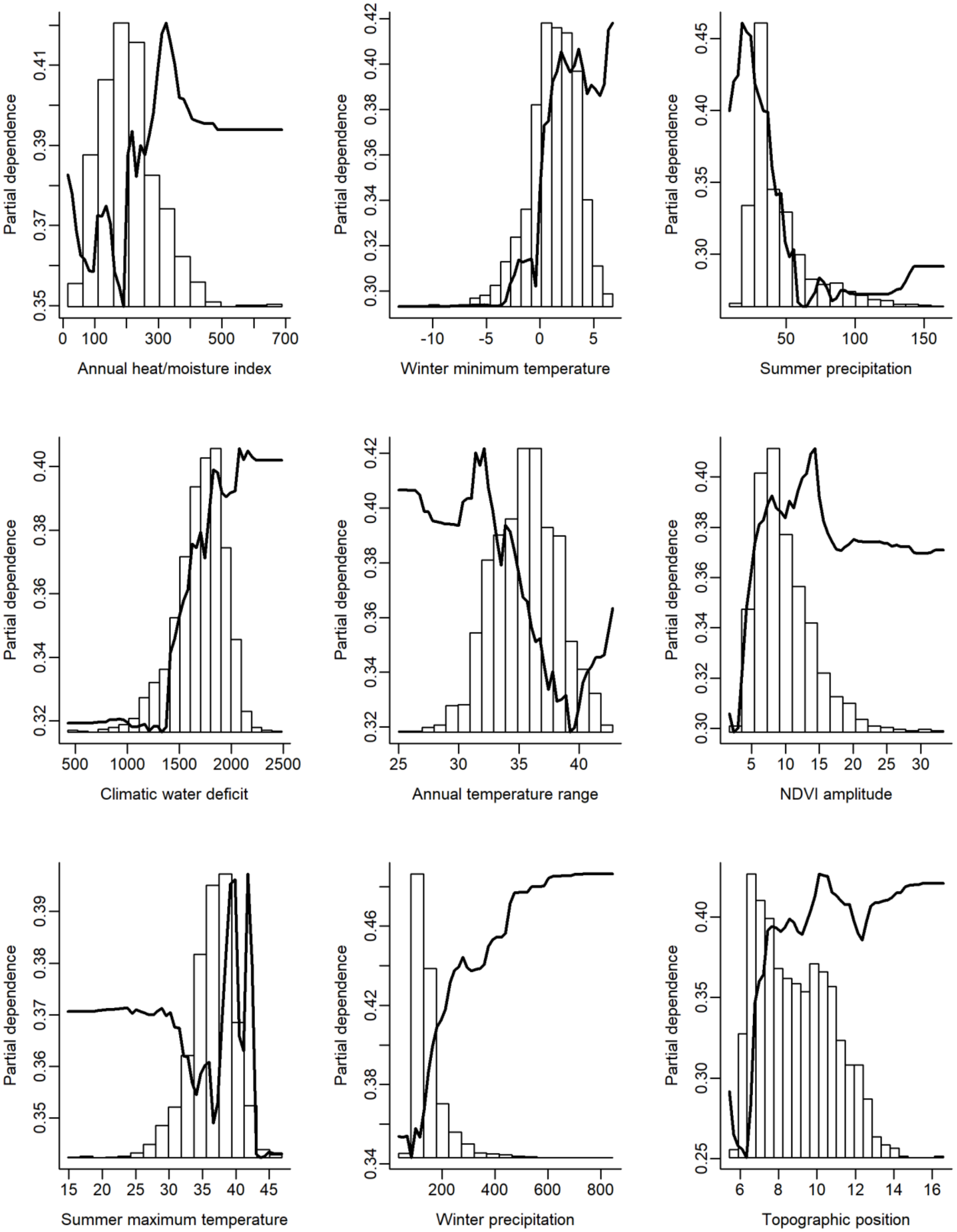

Random Forest response curves for *Malacothrix glabrata*

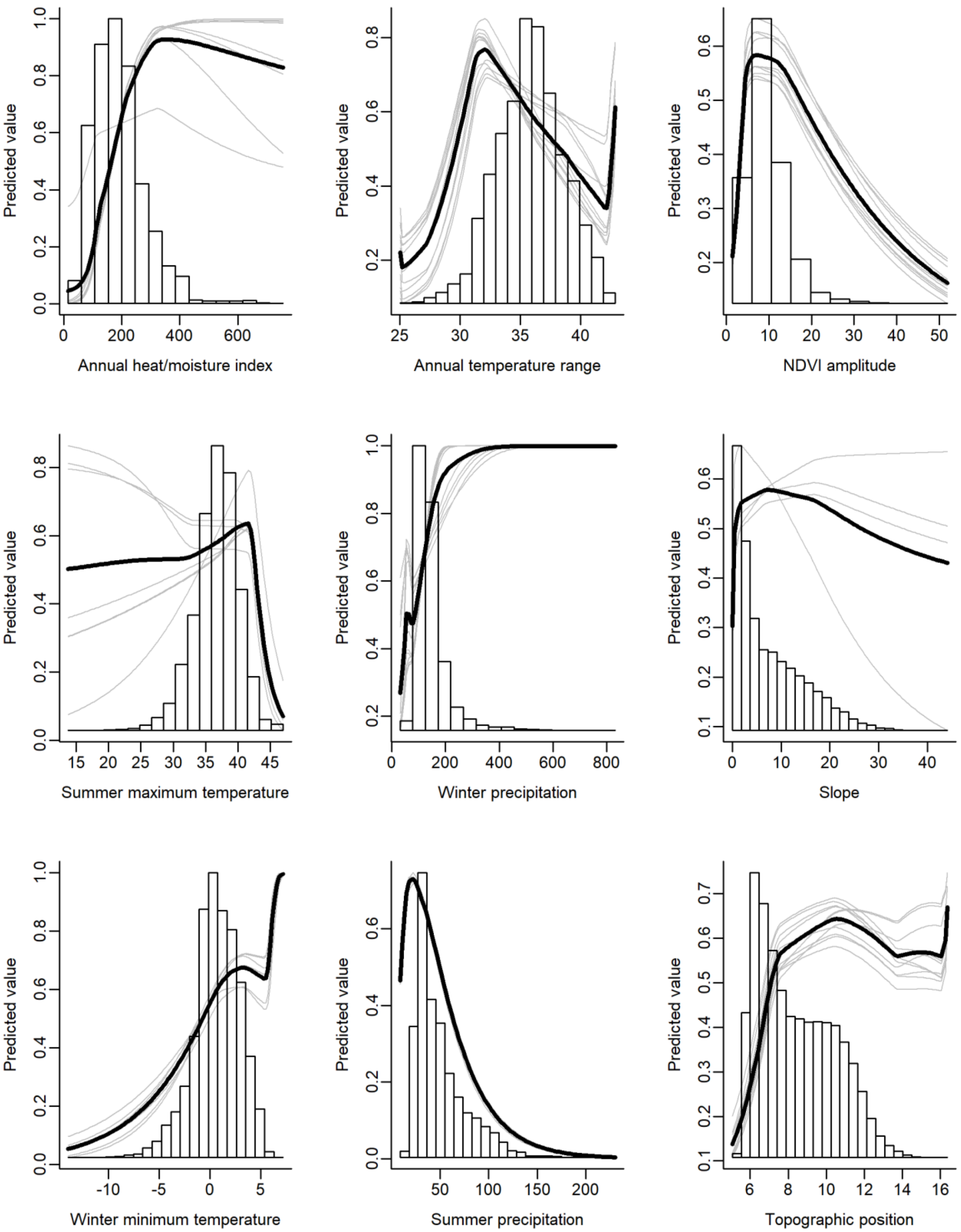

MaxEnt response curves for *Malacothrix glabrata*

34. *Mirabilis laevis* SDM and response curves (GAM, Random Forest, Maxent)

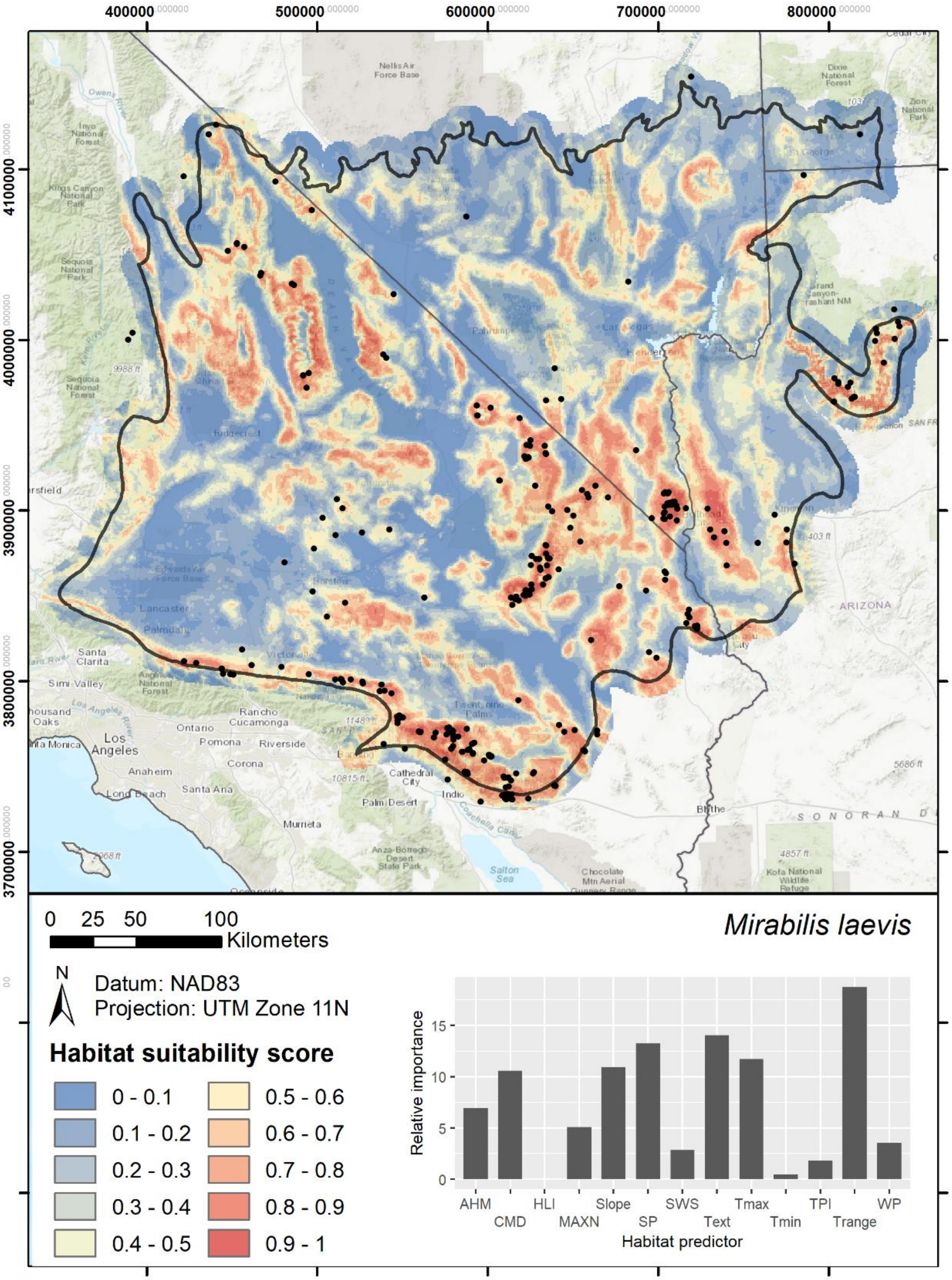

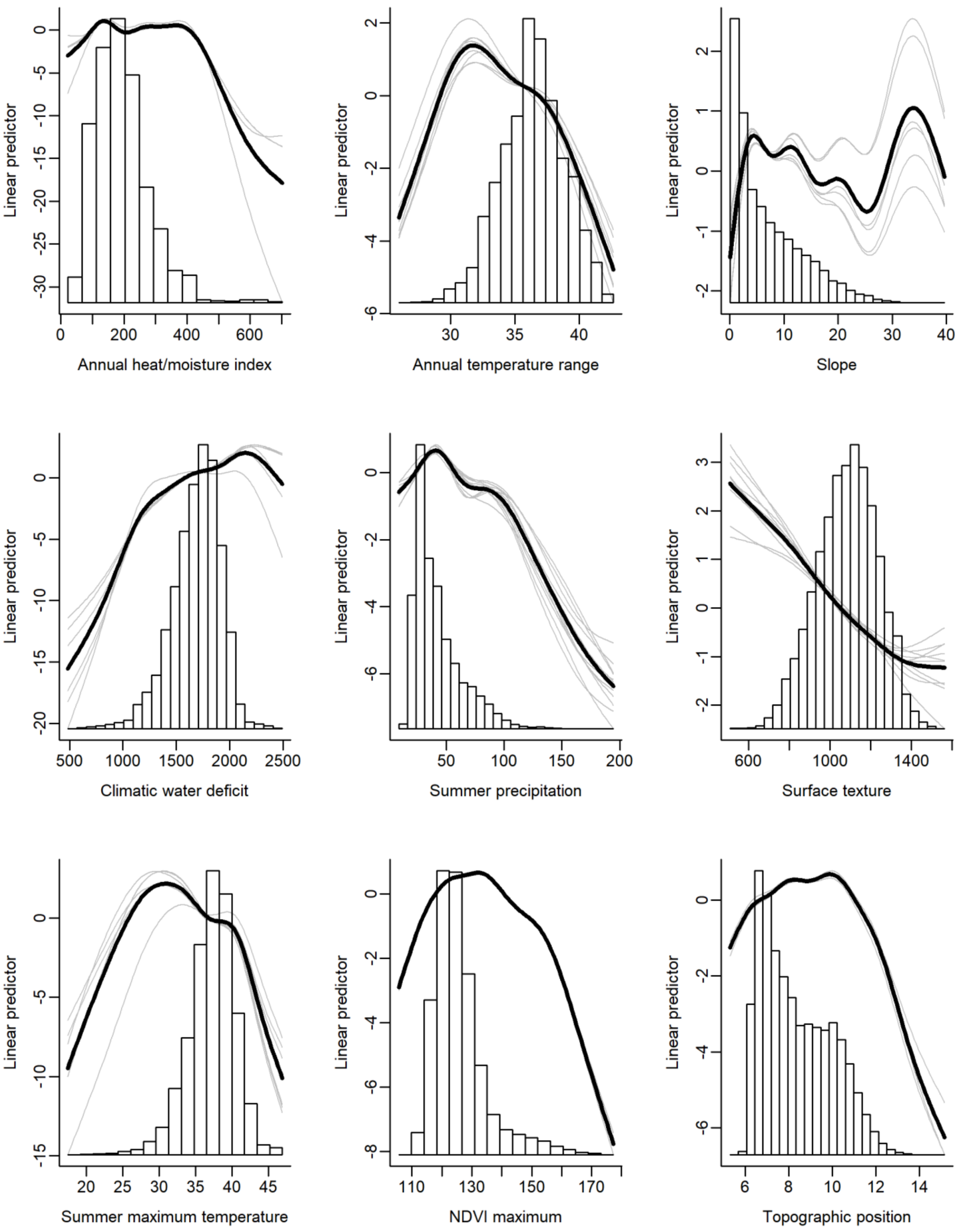

GAM response curves for *Mirabilis laevis*

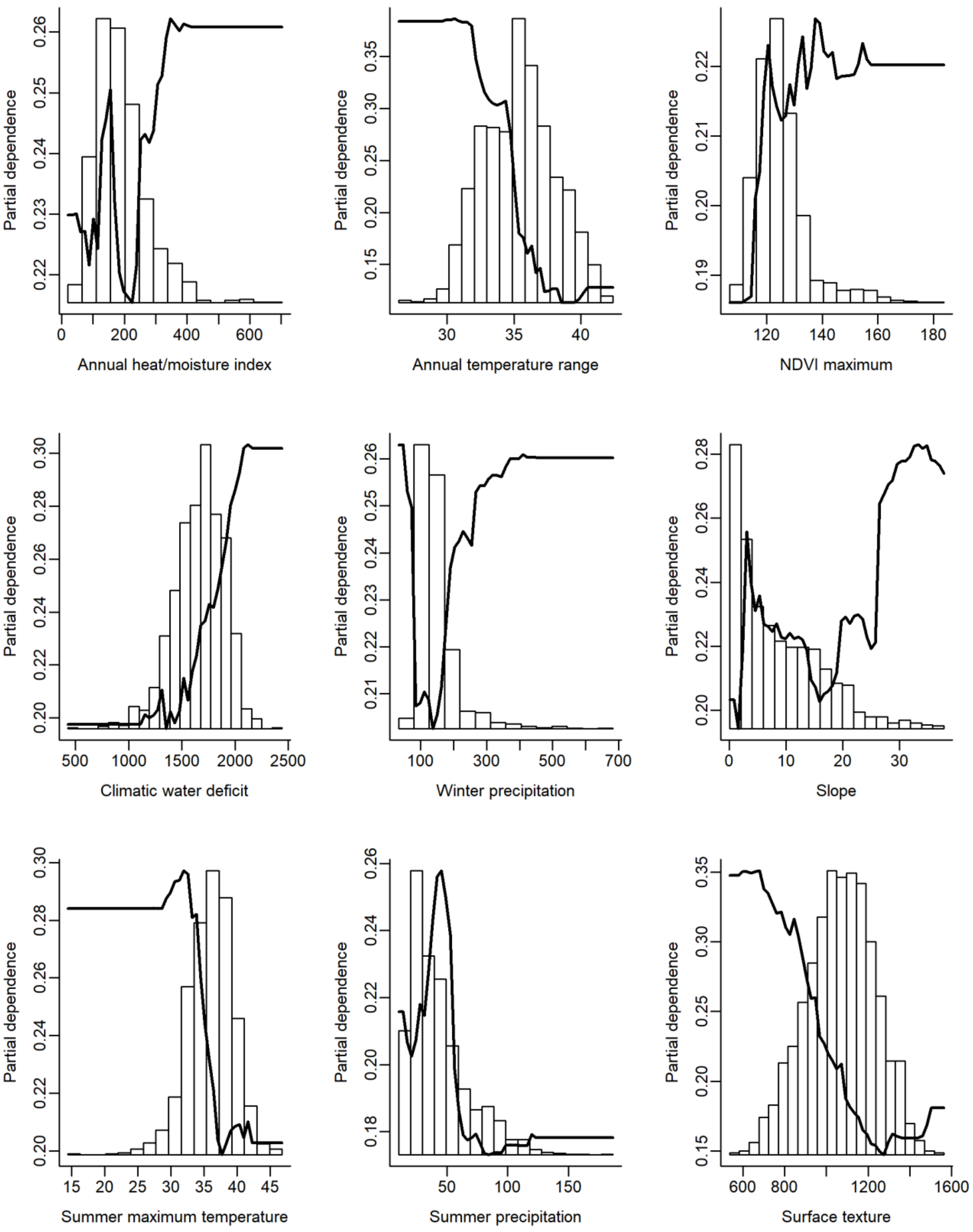

Random Forest response curves for *Mirabilis laevis*

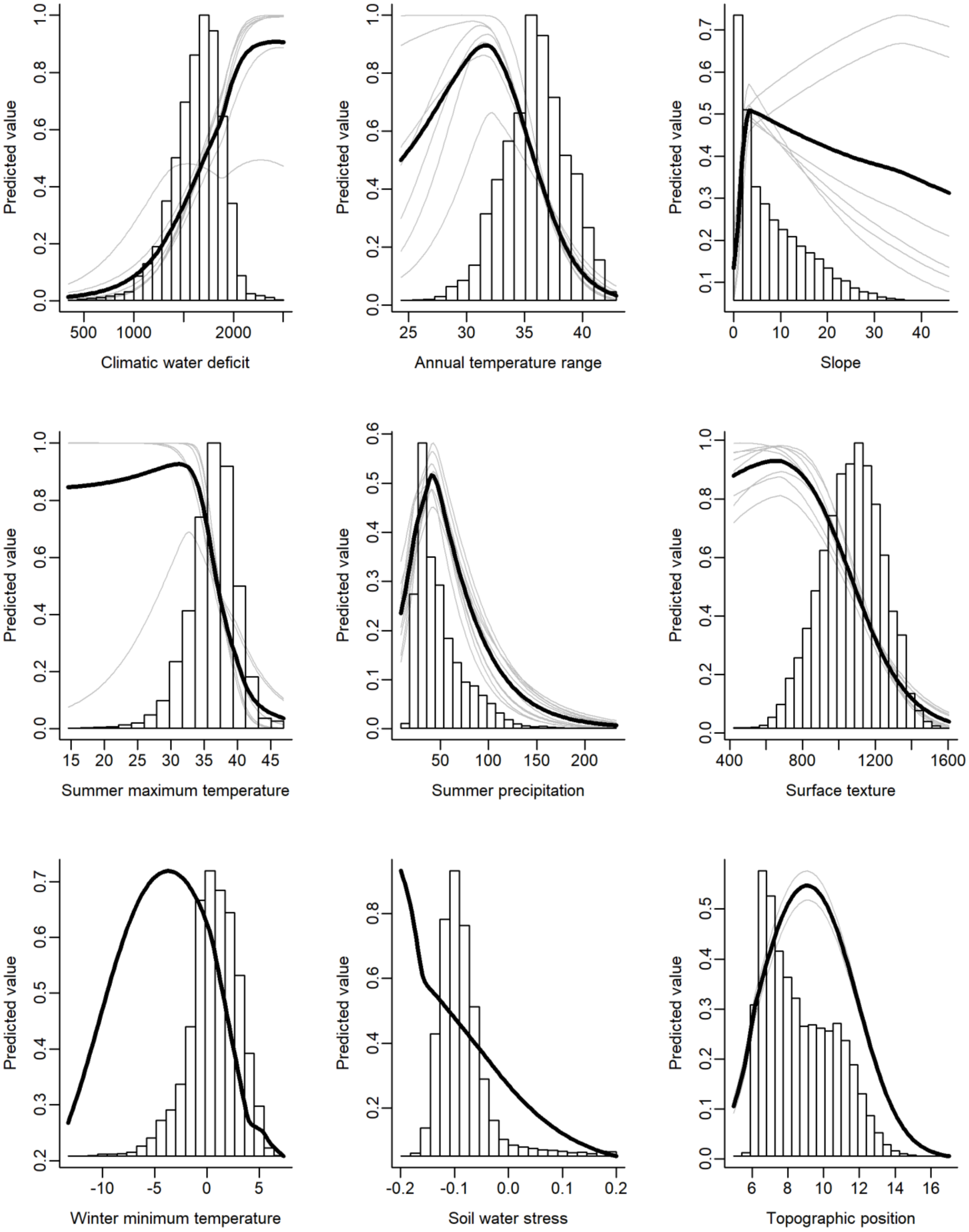

MaxEnt response curves for *Mirabilis laevis*

35. *Muhlenbergia porteri* SDM and response curves (GAM, Random Forest, Maxent)

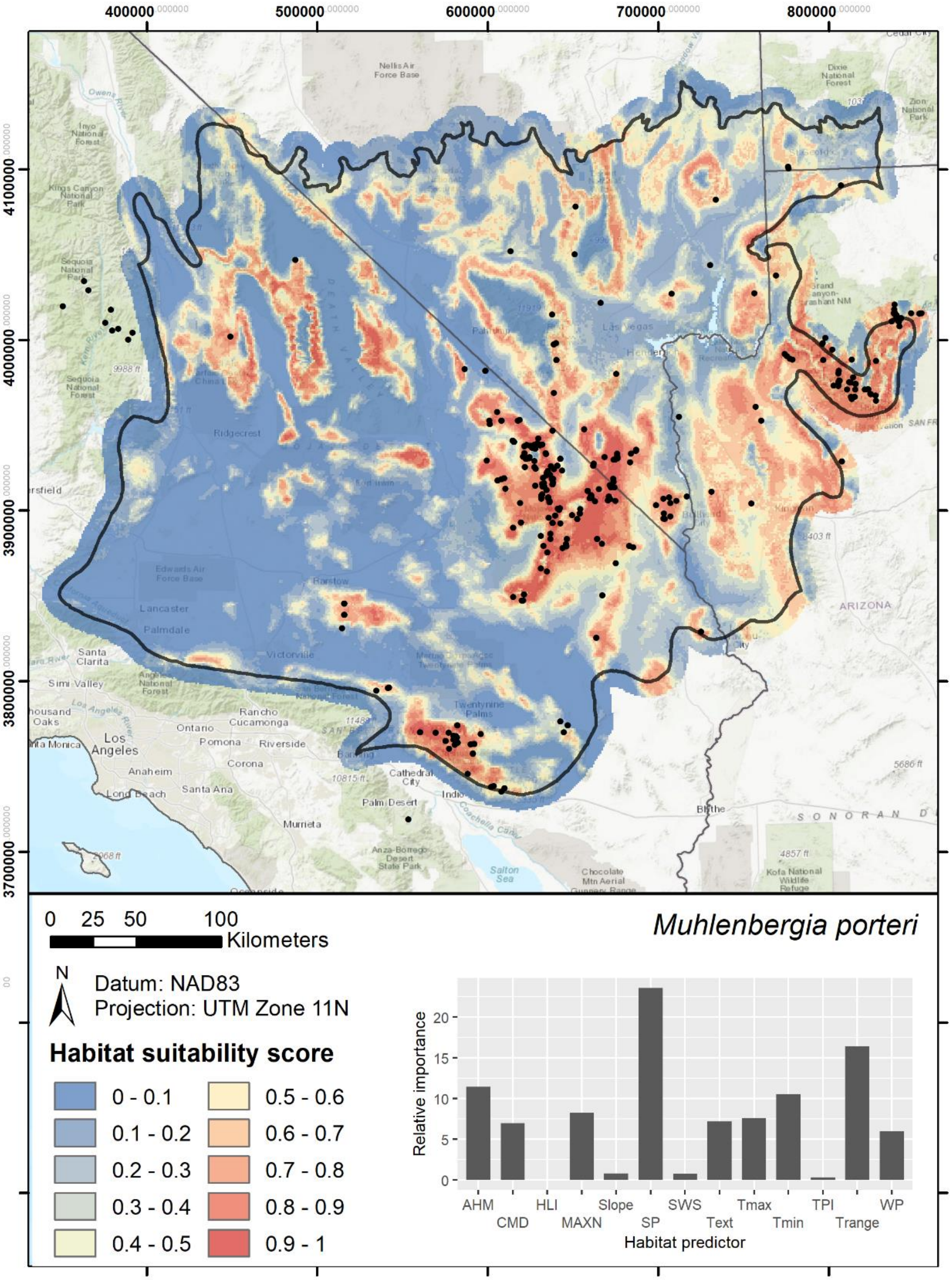

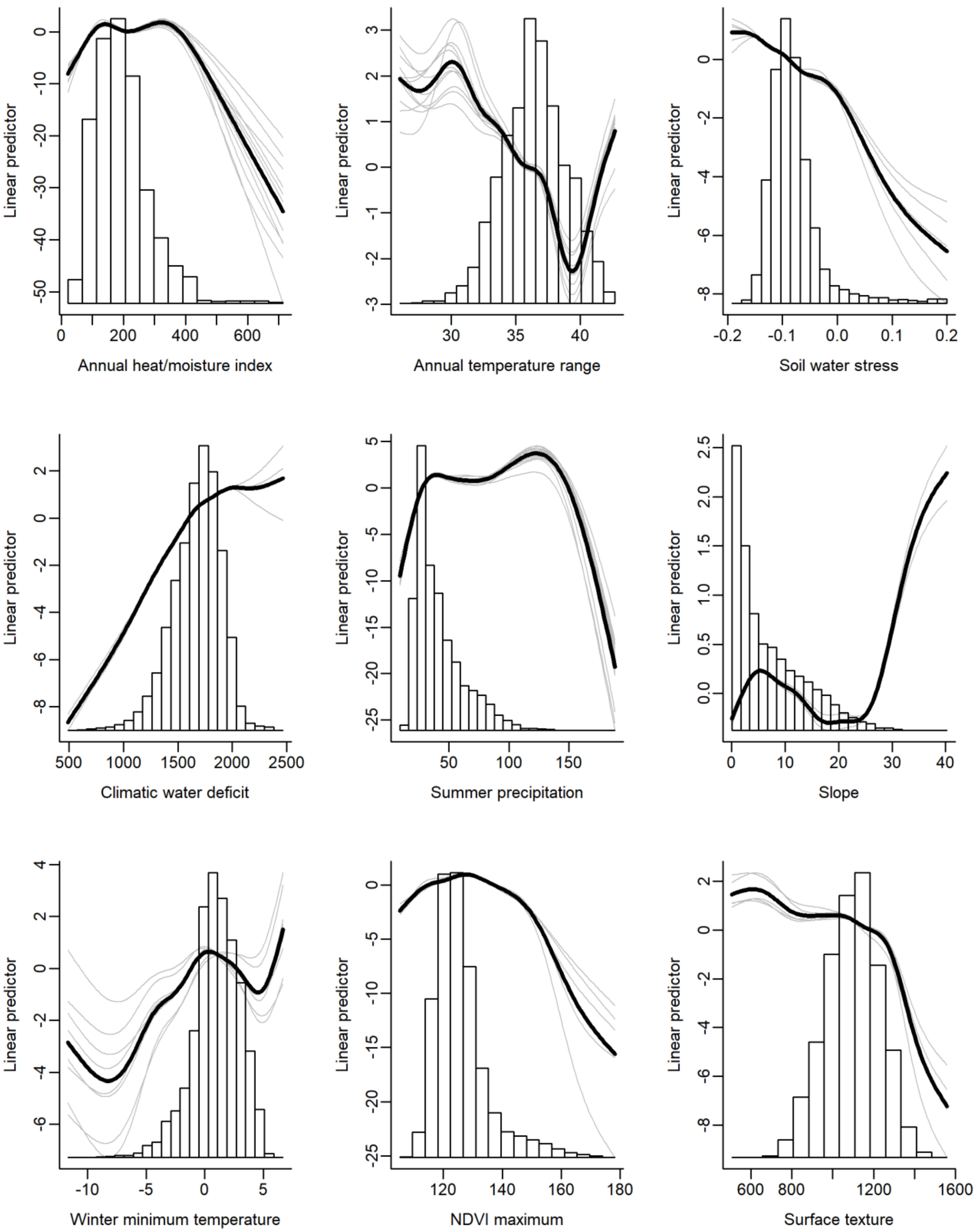

GAM response curves for *Muhlenbergia porteri*

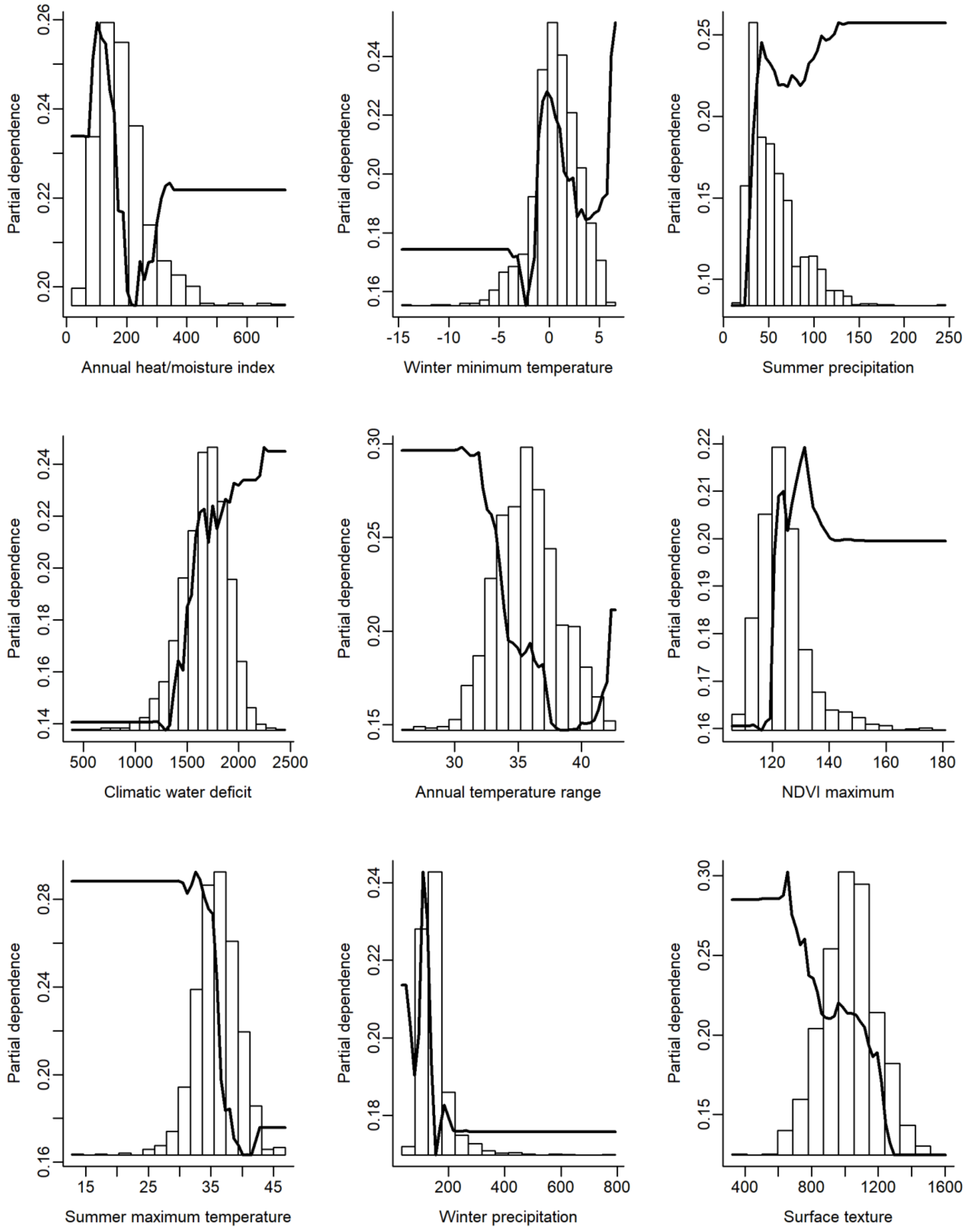

Random Forest response curves for *Muhlenbergia porteri*

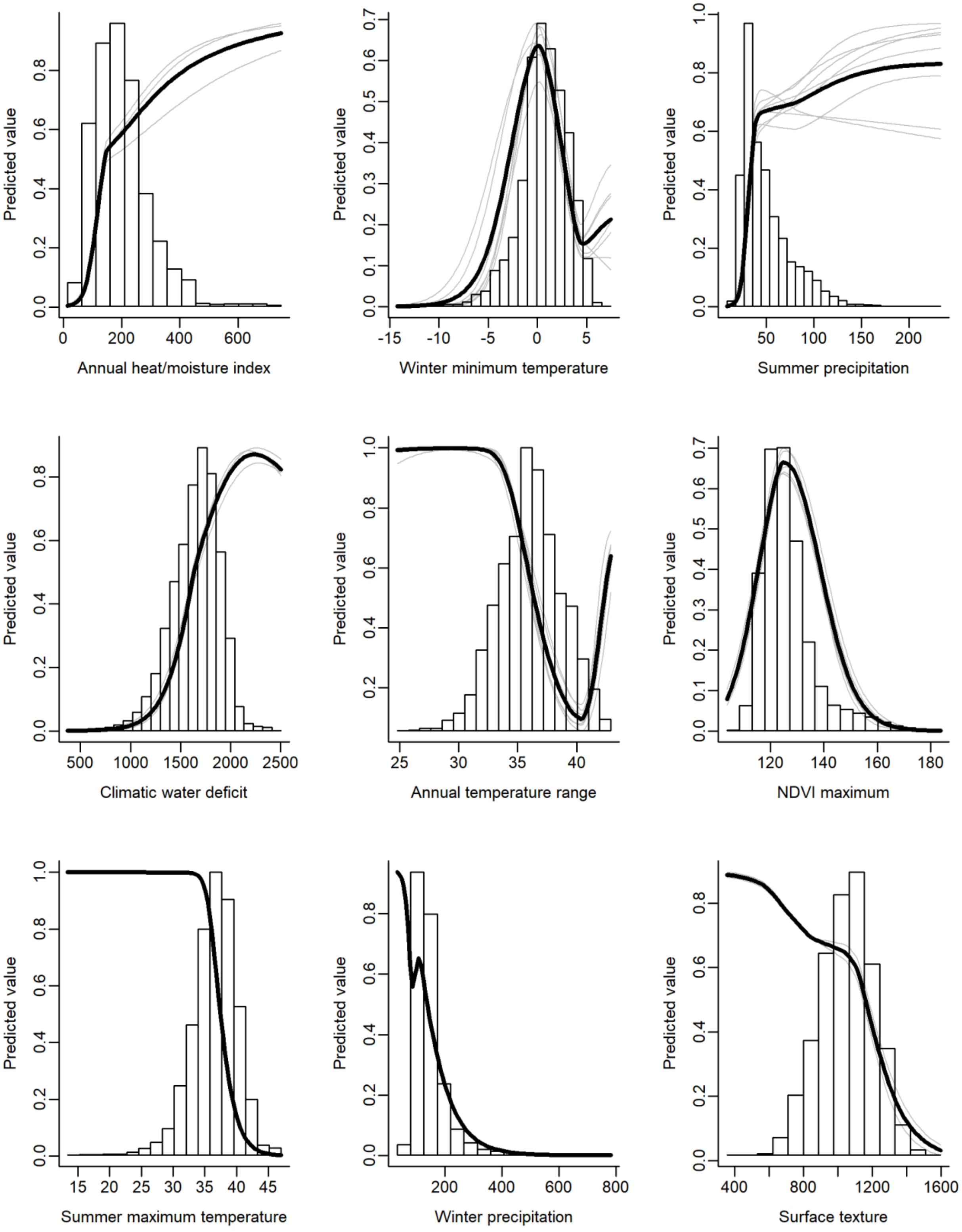

MaxEnt response curves for *Muhlenbergia porteri*

36. *Oenothera cespitosa* SDM and response curves (GAM, Random Forest, Maxent)

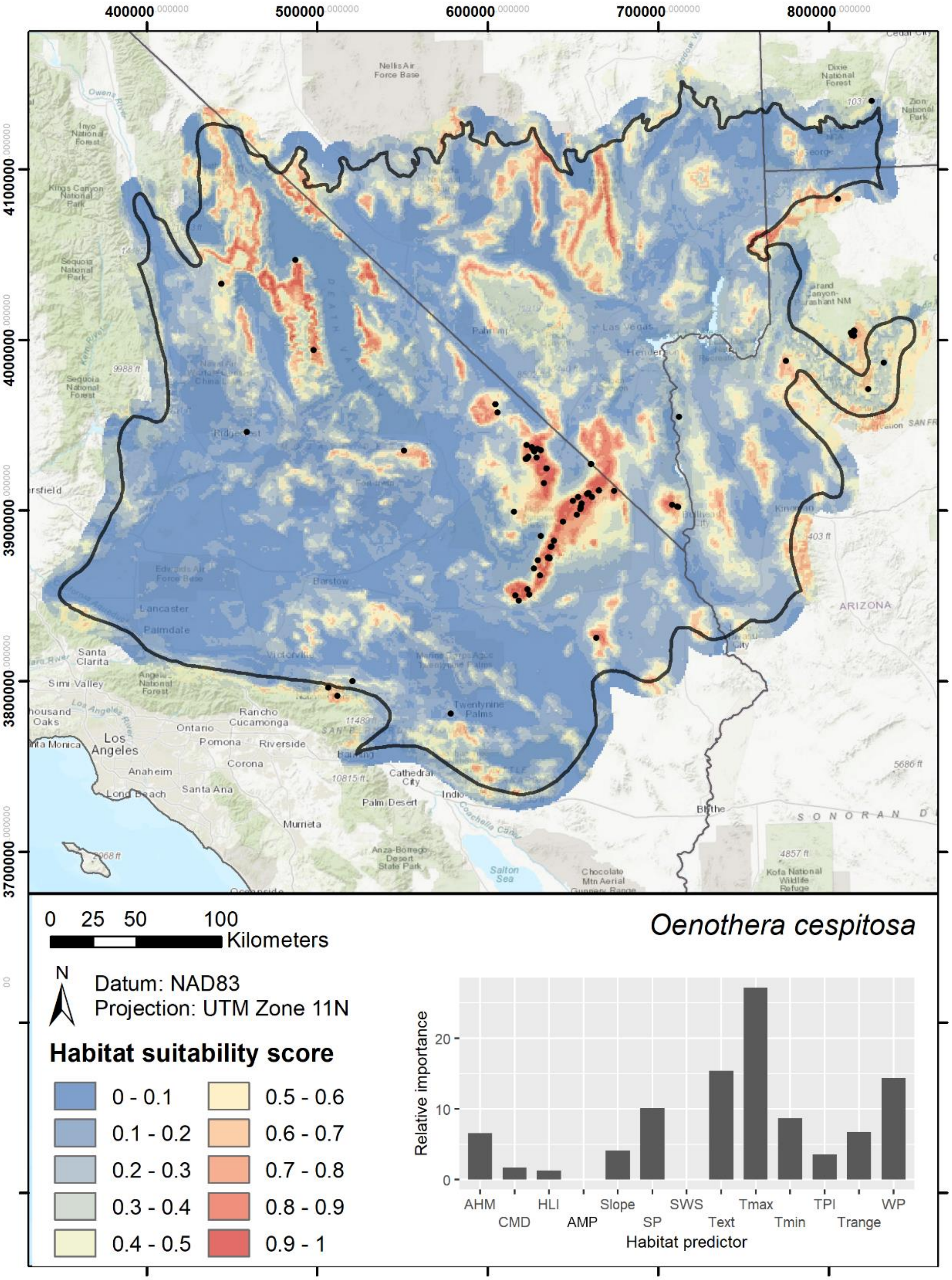

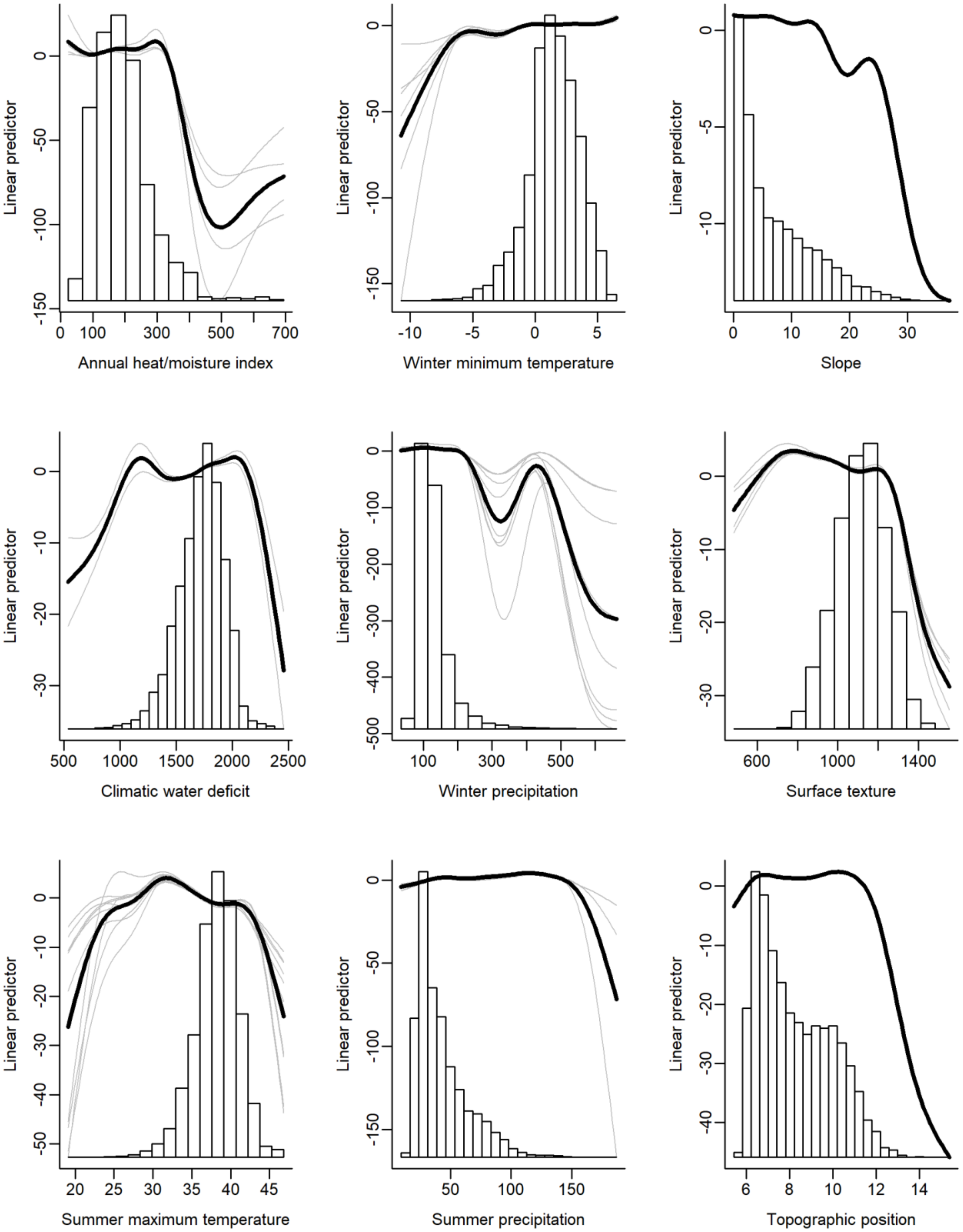

GAM response curves for *Oenothera cespitosa*

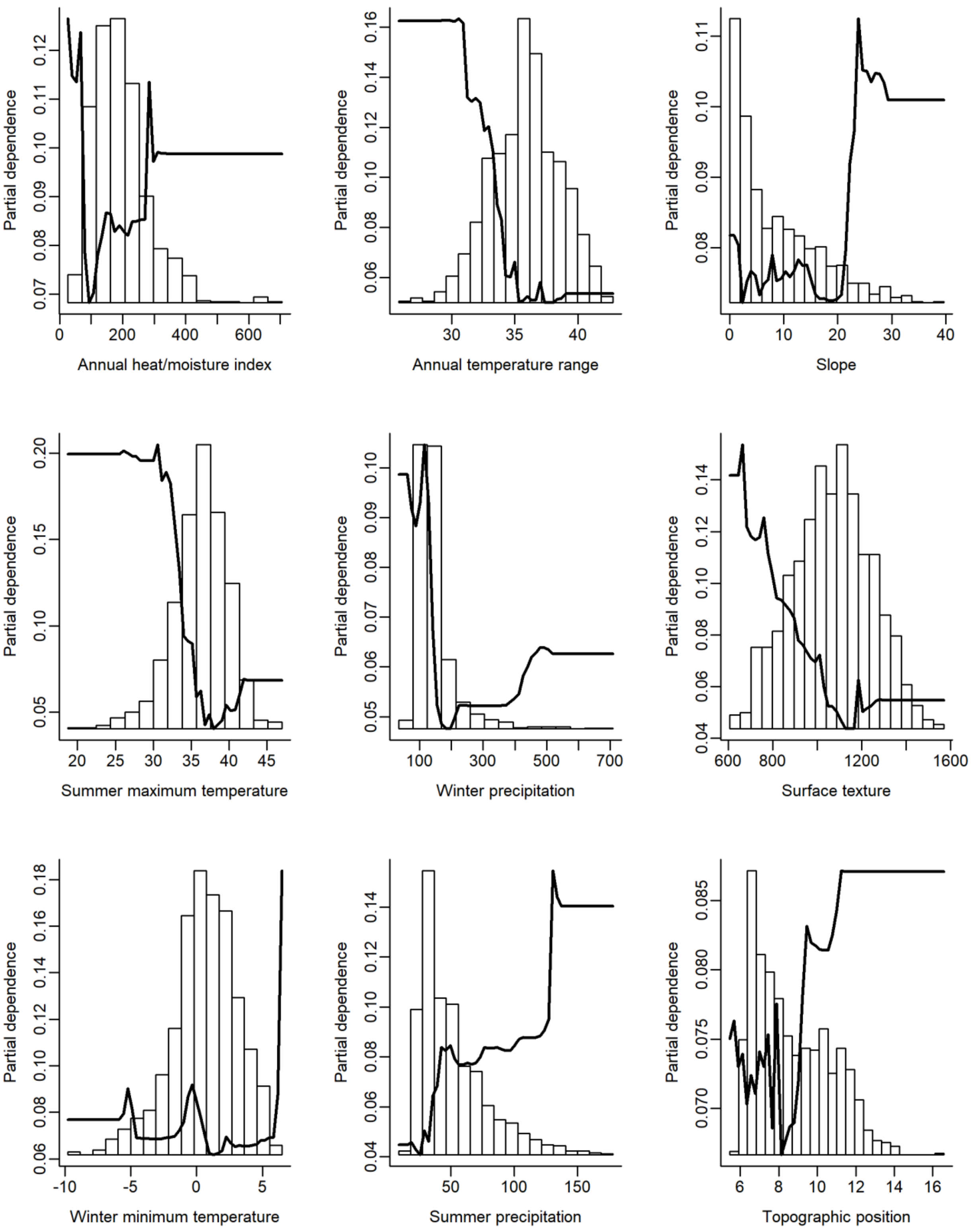

Random Forest response curves for *Oenothera cespitosa*

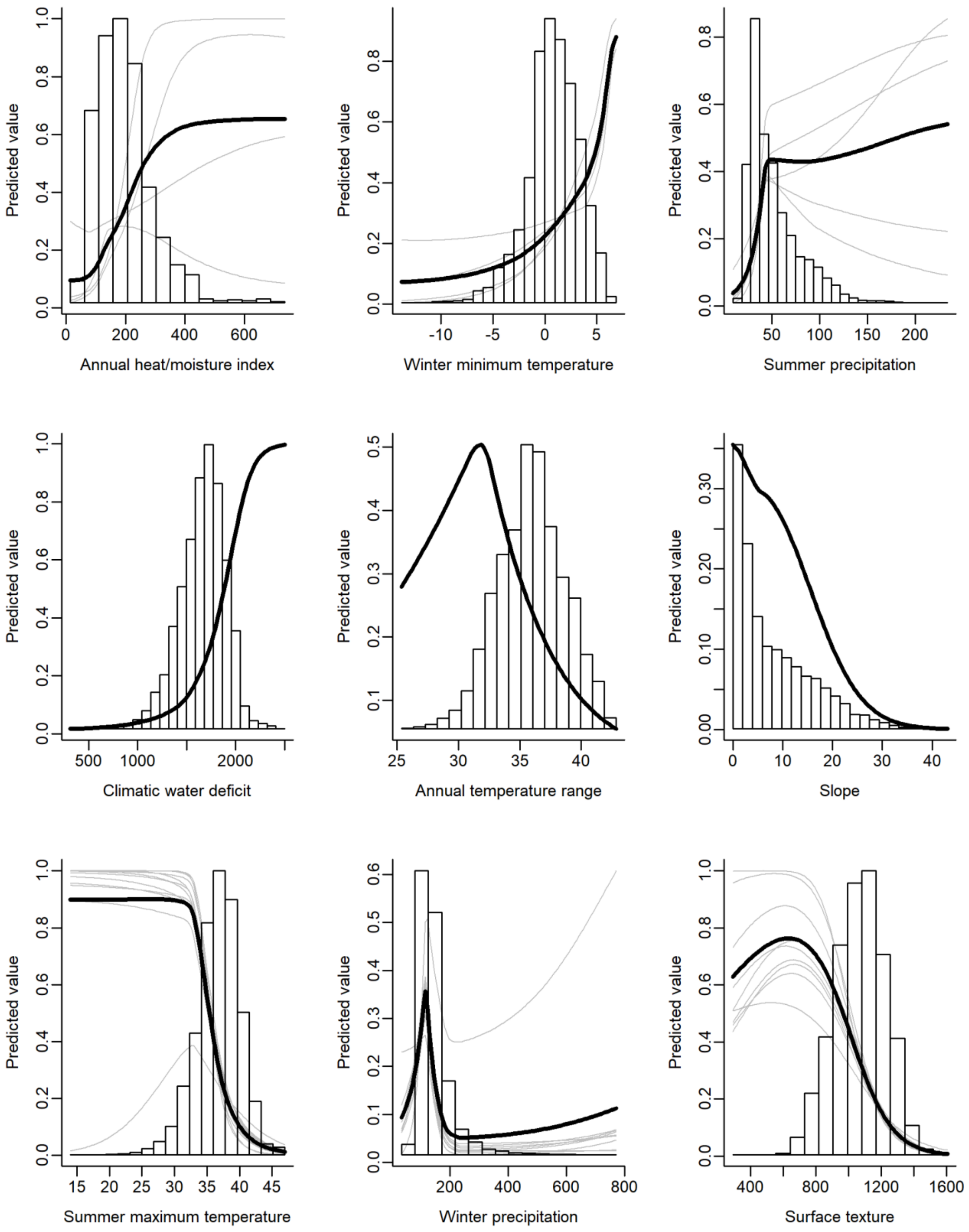

MaxEnt response curves for *Oenothera cespitosa*

37. *Oenothera deltoides* SDM and response curves (GAM, Random Forest, Maxent)

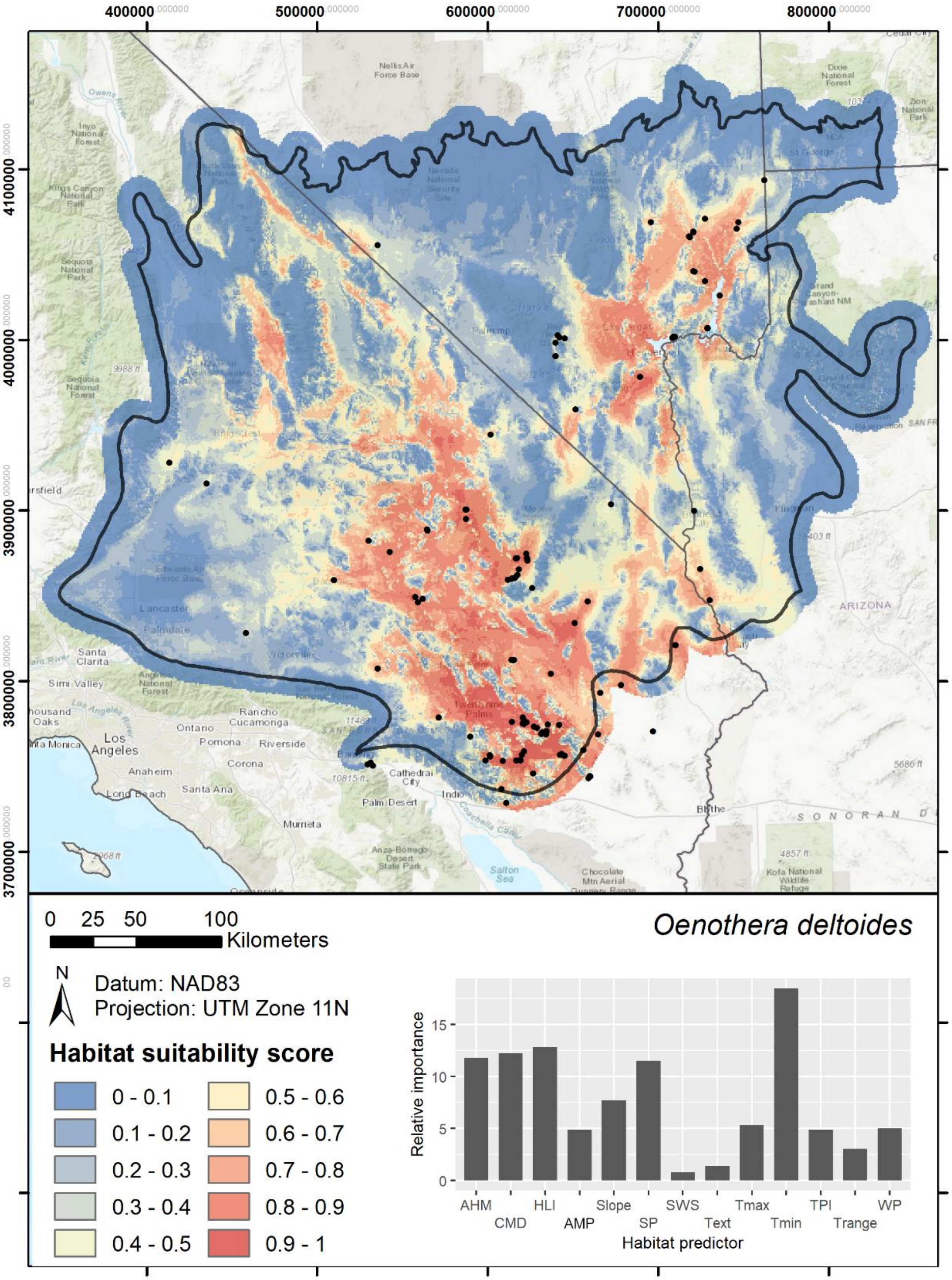

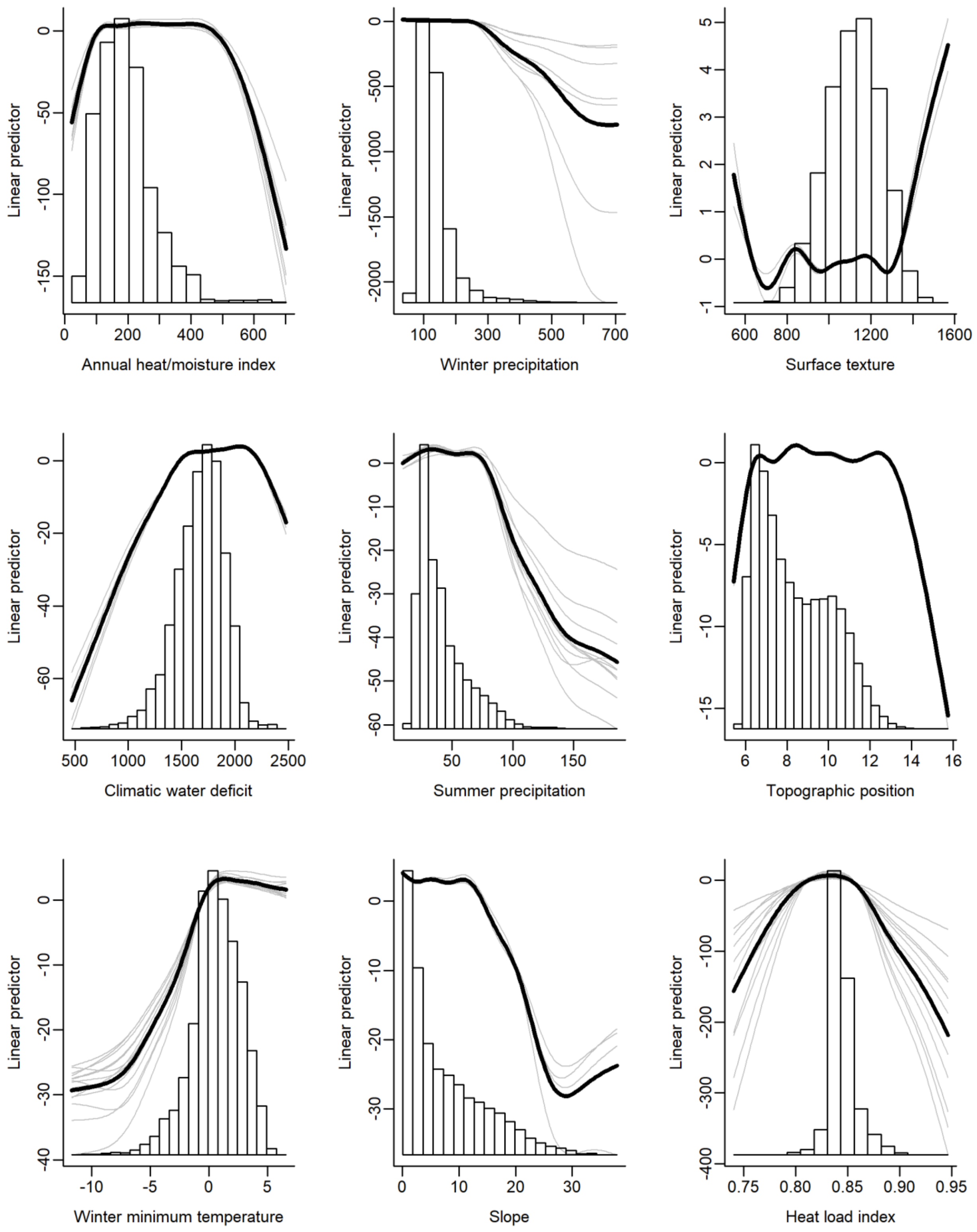

GAM response curves for *Oenothera deltoides*

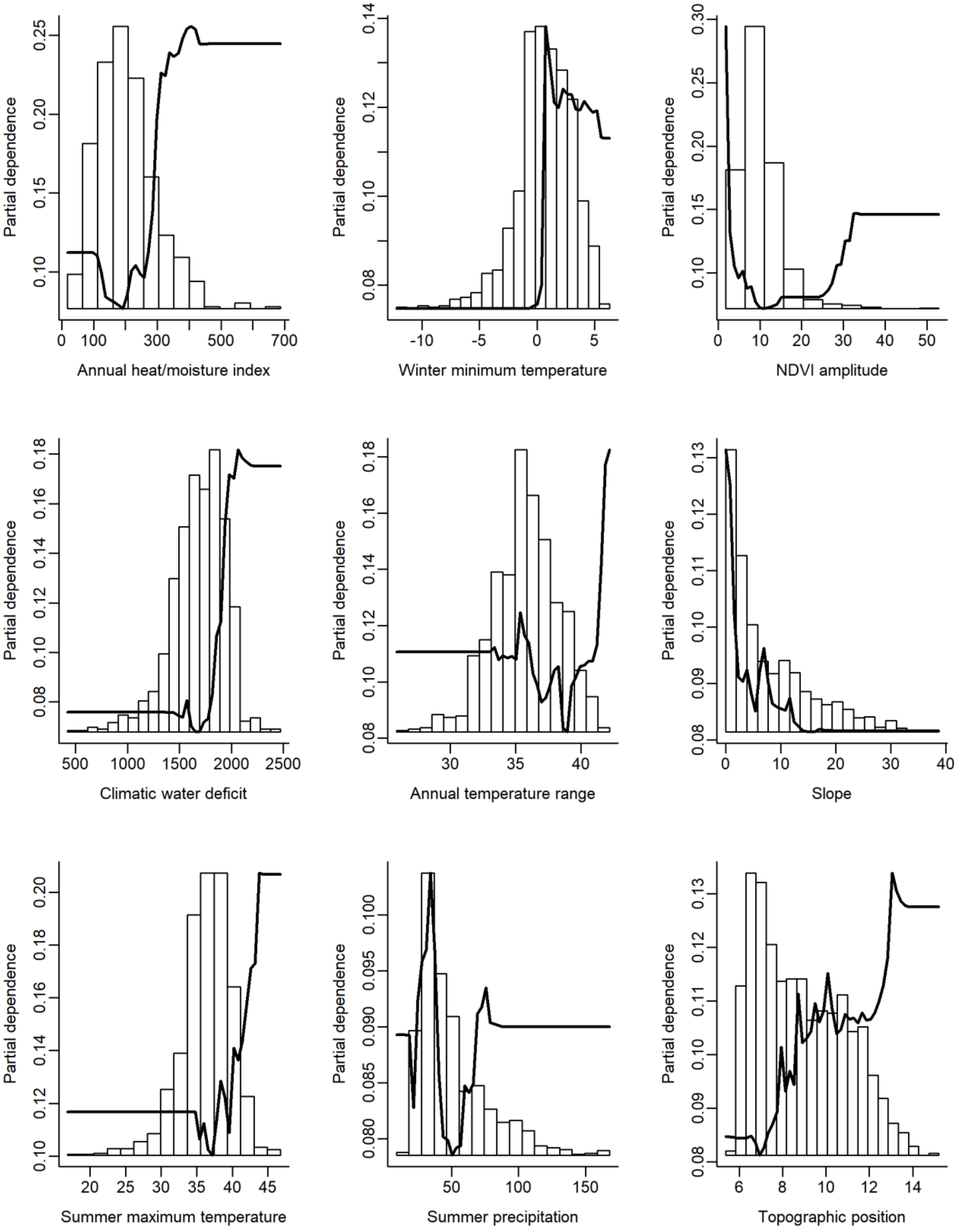

Random Forest response curves for *Oenothera deltoides*

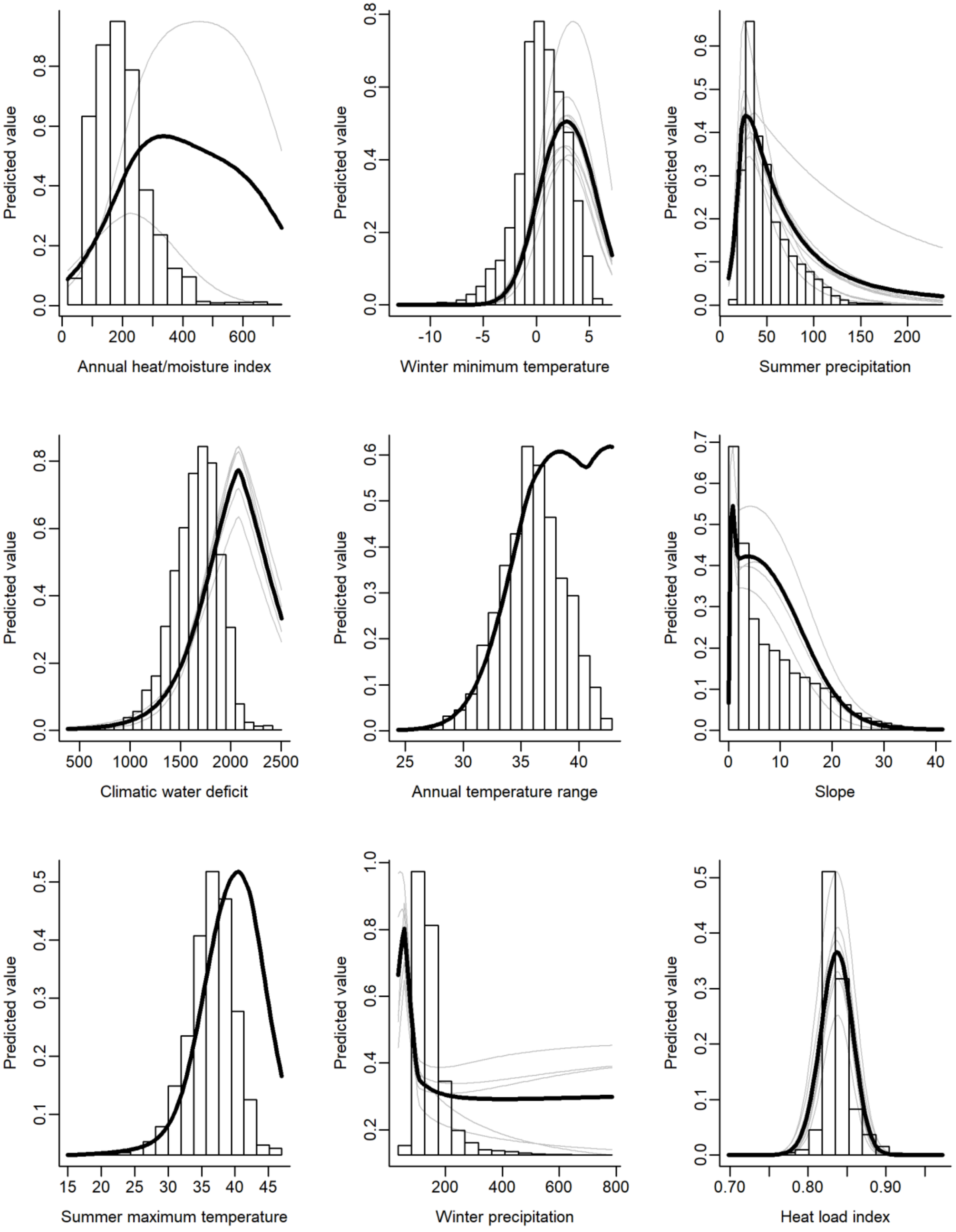

MaxEnt response curves for *Oenothera deltoides*

38. *Oenothera primiveris* SDM and response curves (GAM, Random Forest, Maxent)

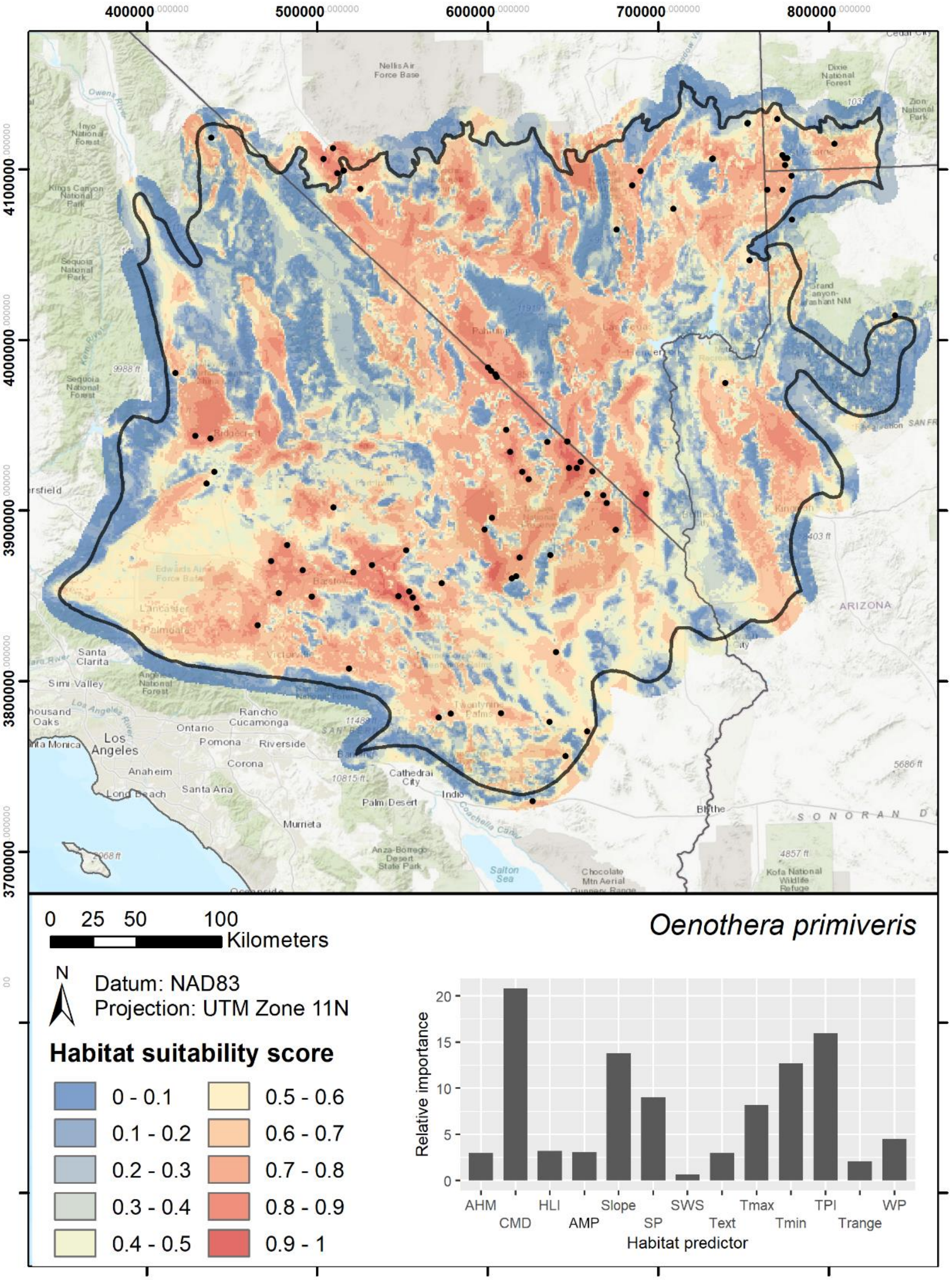

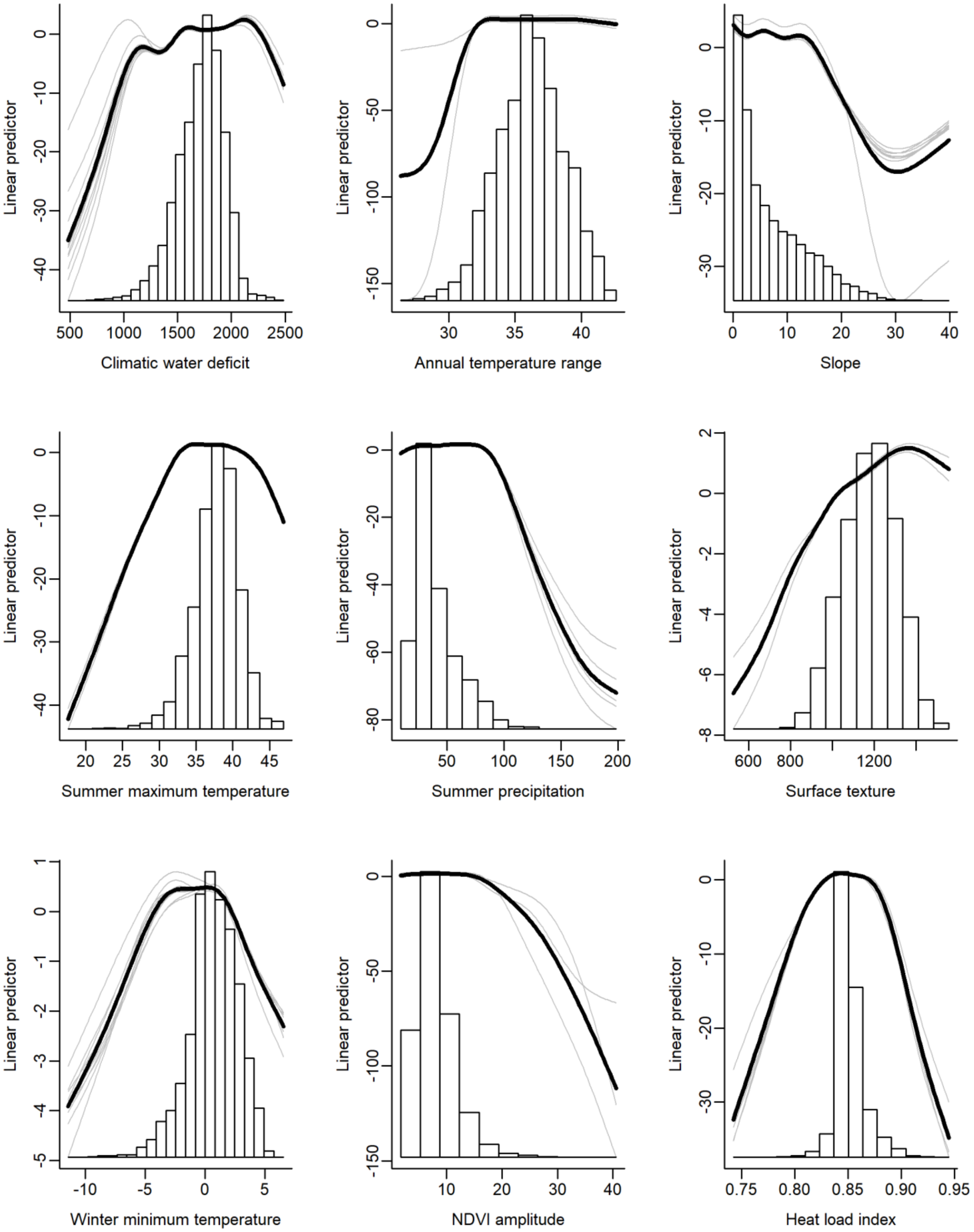

GAM response curves for *Oenothera primiveris*

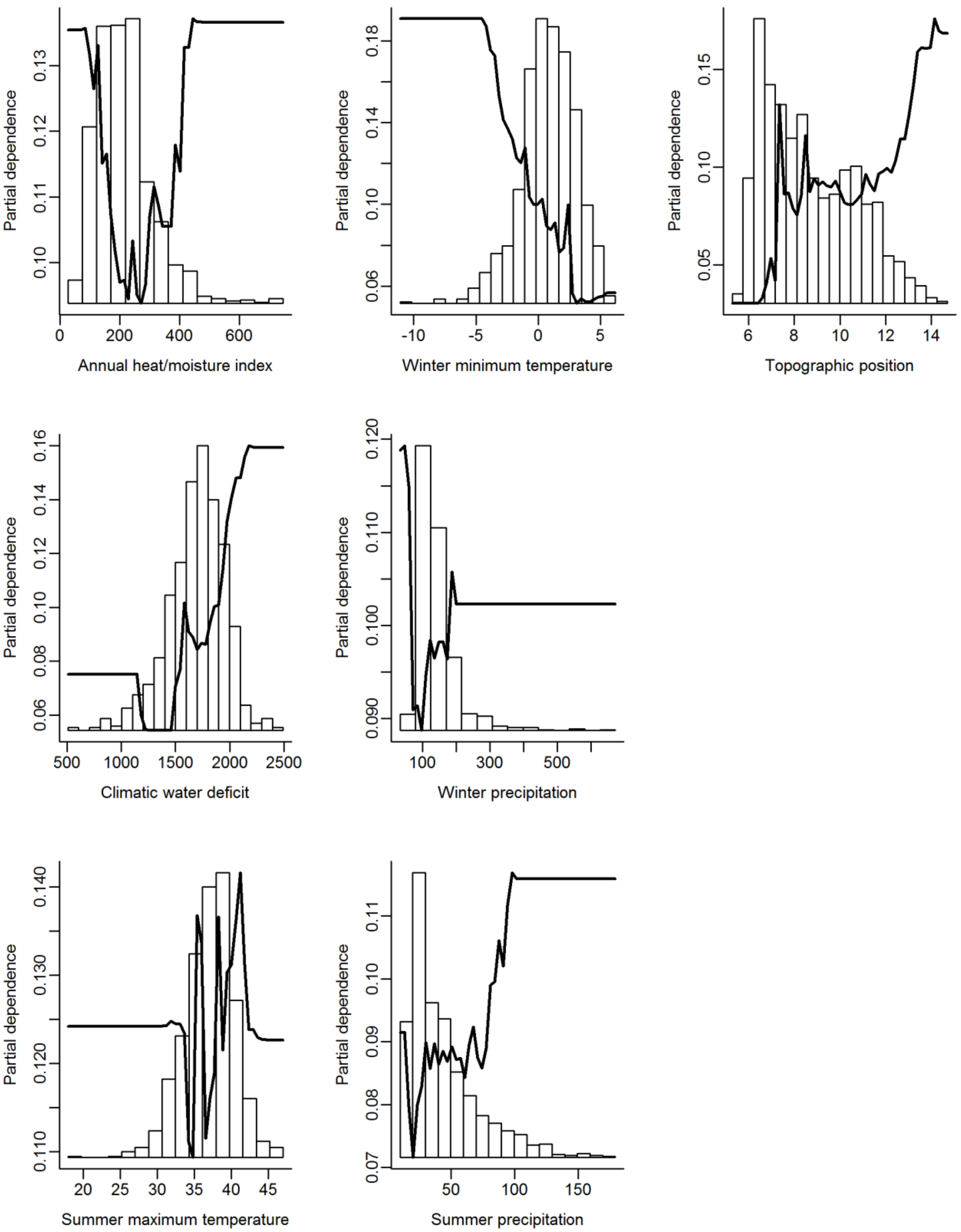

Random Forest response curves for *Oenothera primiveris*

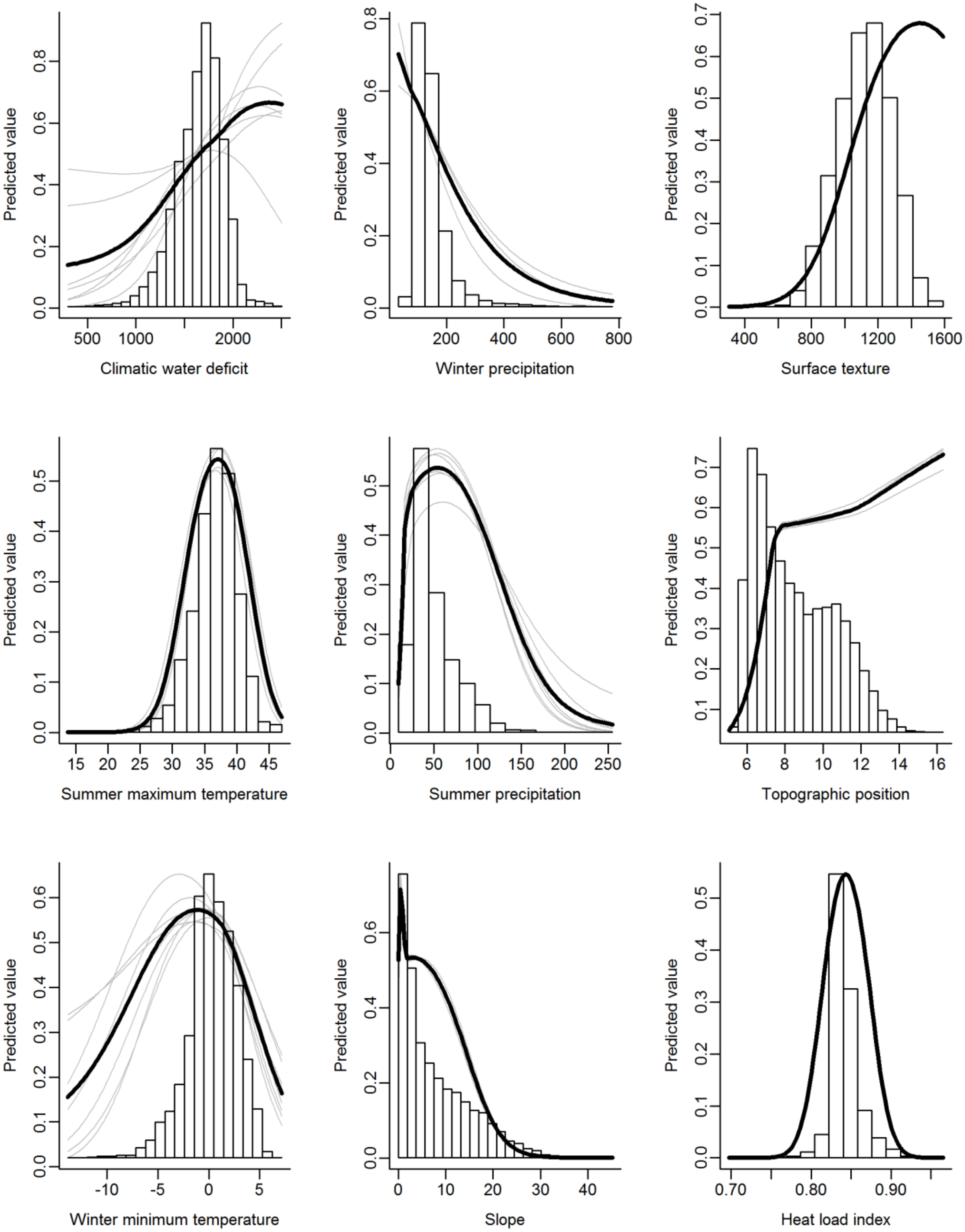

MaxEnt response curves for *Oenothera primiveris*

39. *Opuntia basilaris* SDM and response curves (GAM, Random Forest, Maxent)

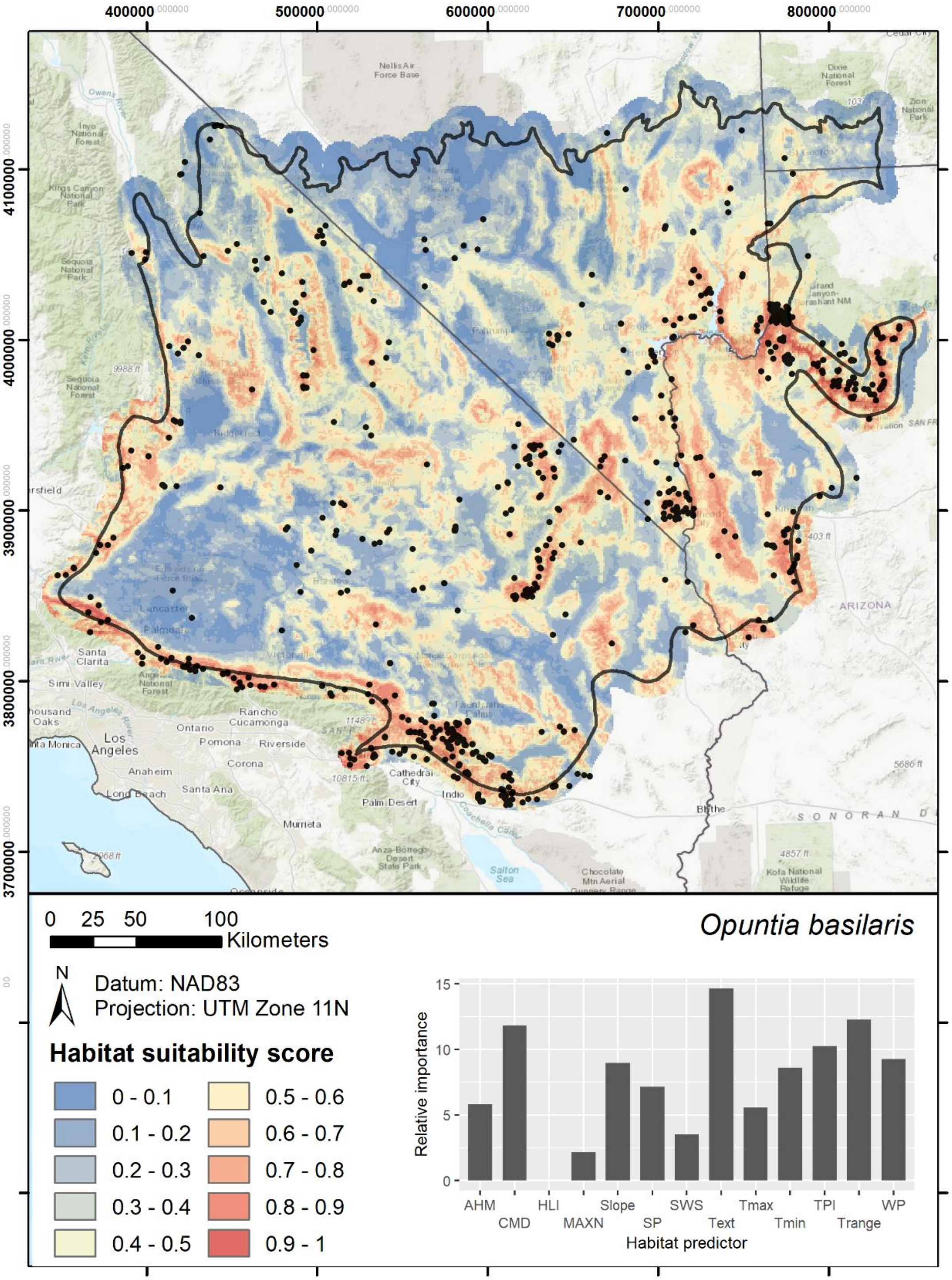

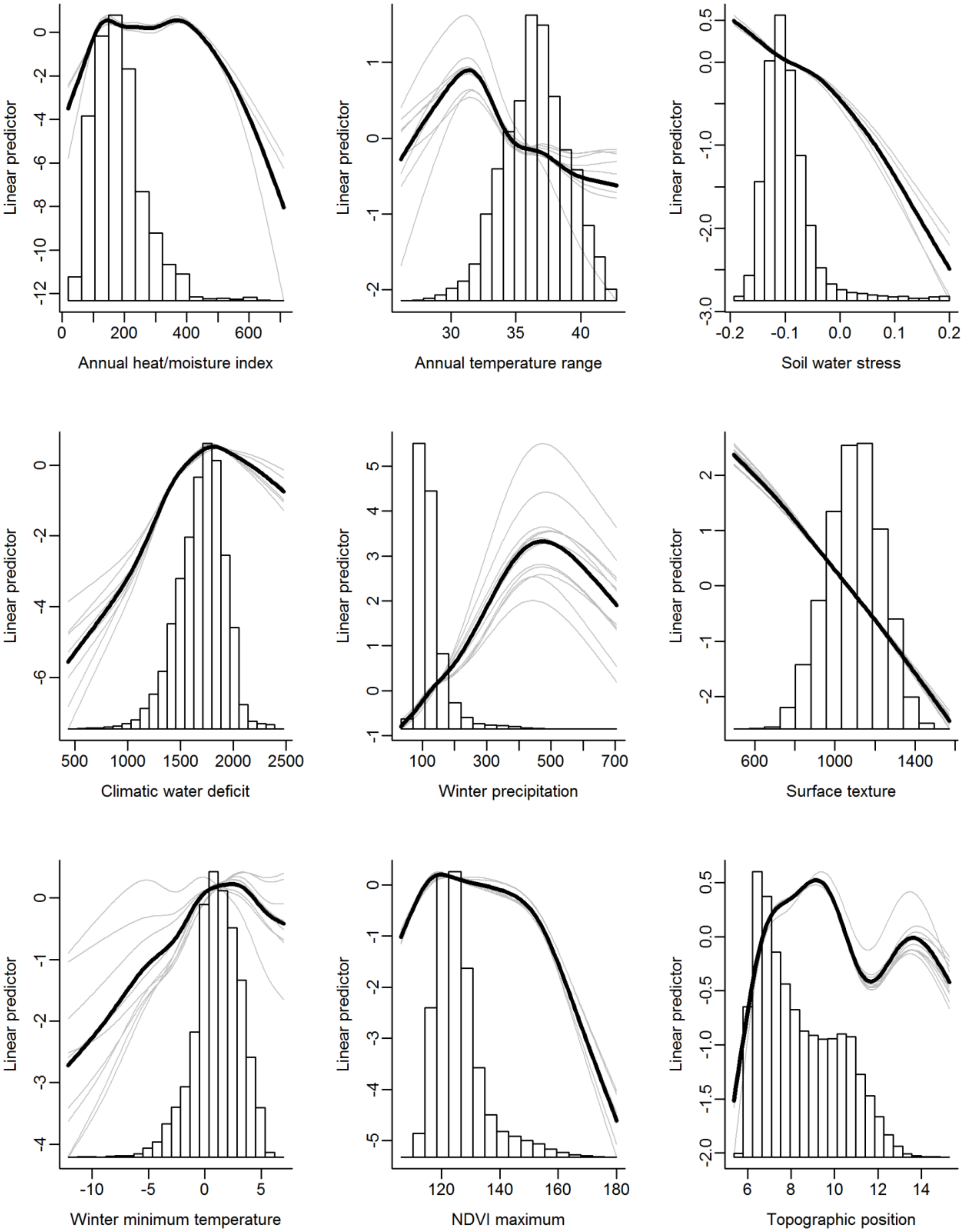

GAM response curves for *Opuntia basilaris*

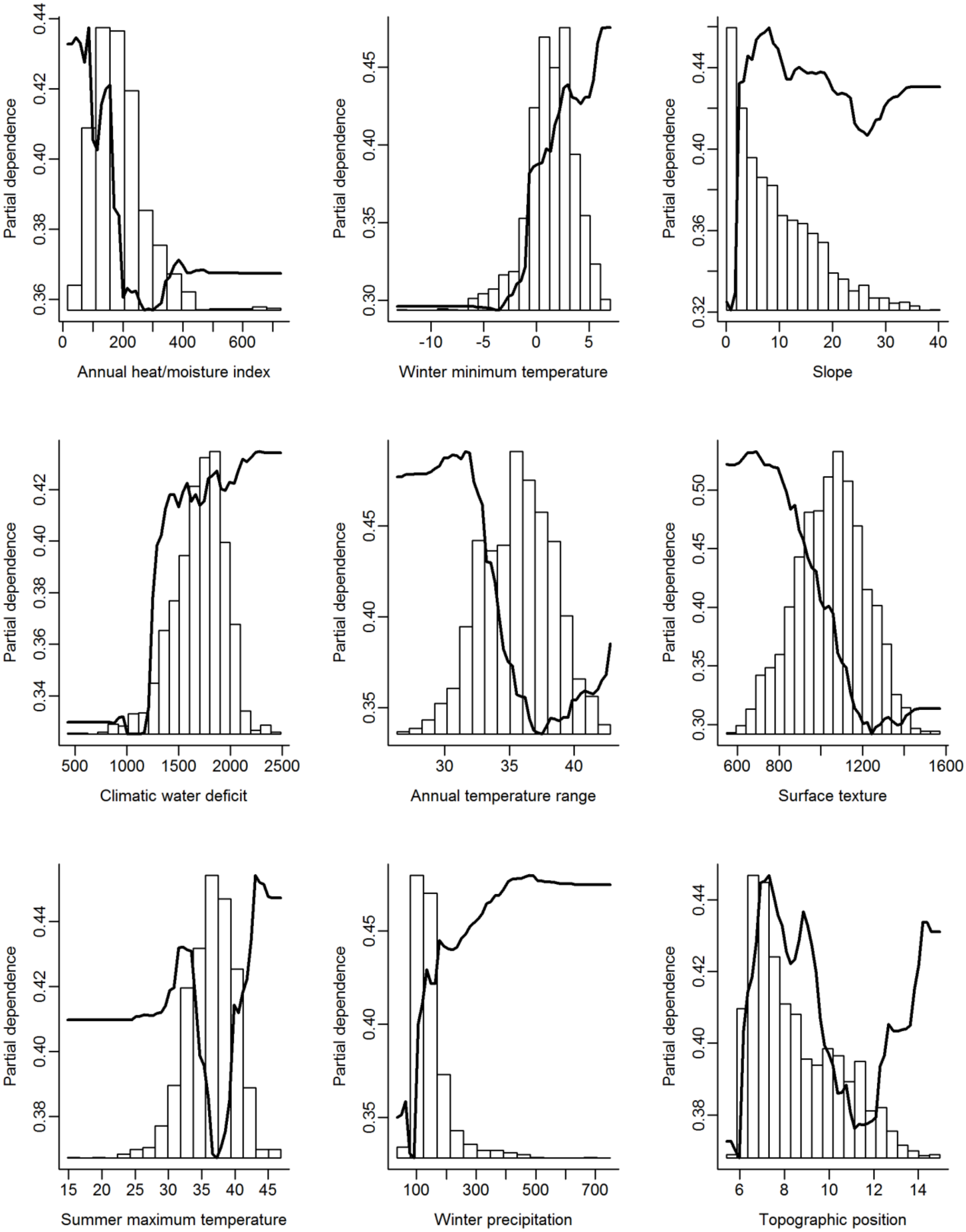

Random Forest response curves for *Opuntia basilaris*

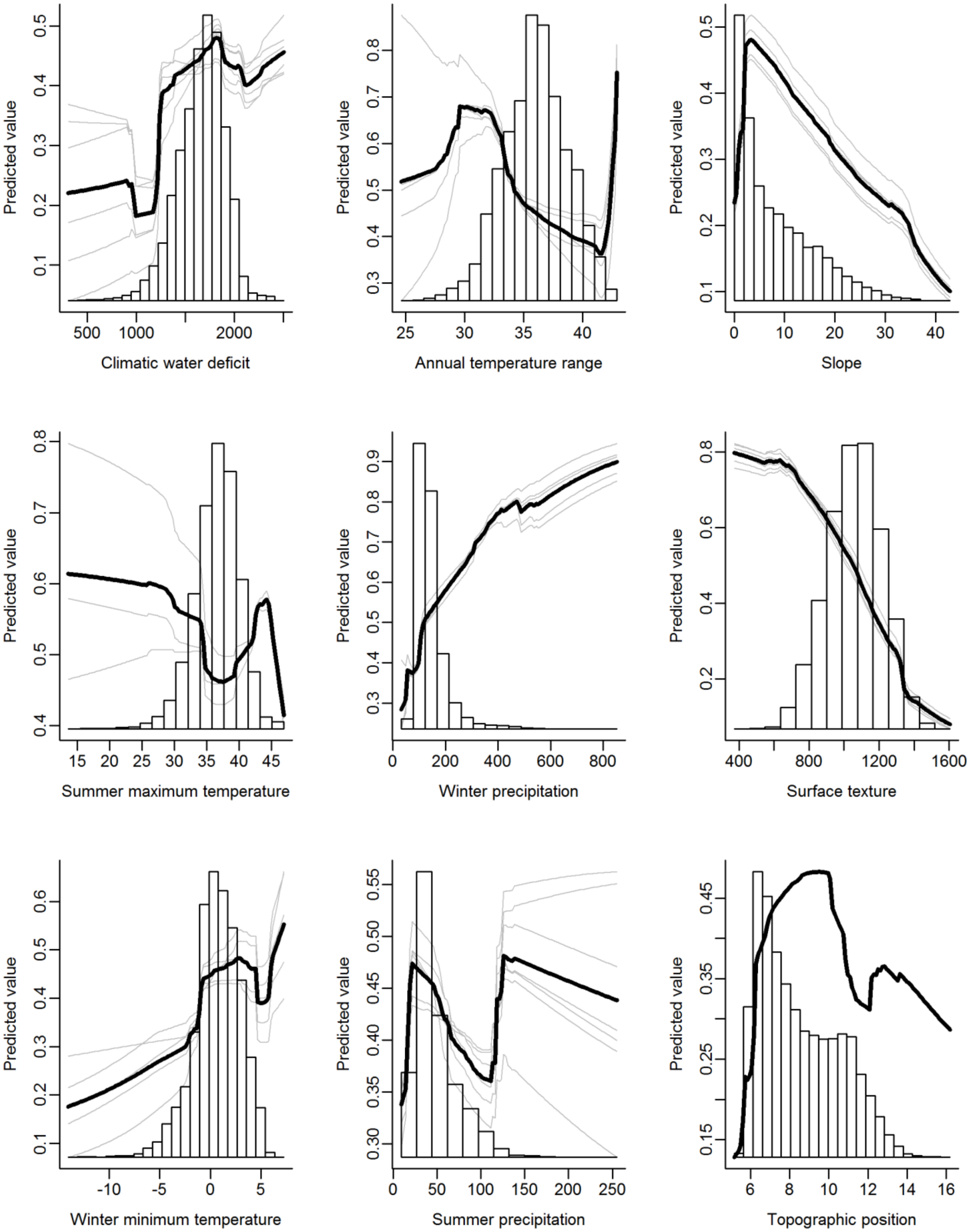

MaxEnt response curves for *Opuntia basilaris*

40. *Penstemon palmeri* SDM and response curves (GAM, Random Forest, Maxent)

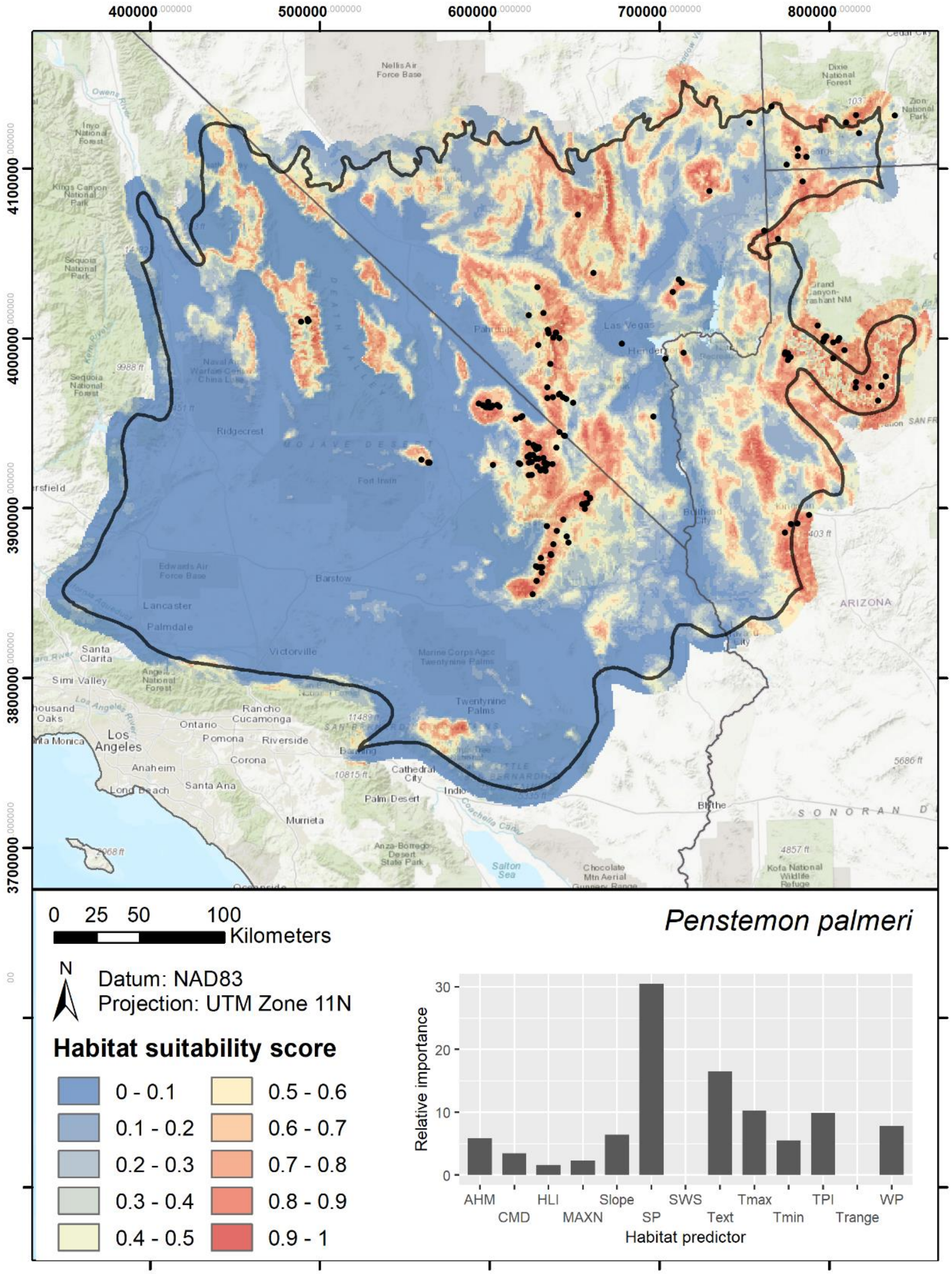

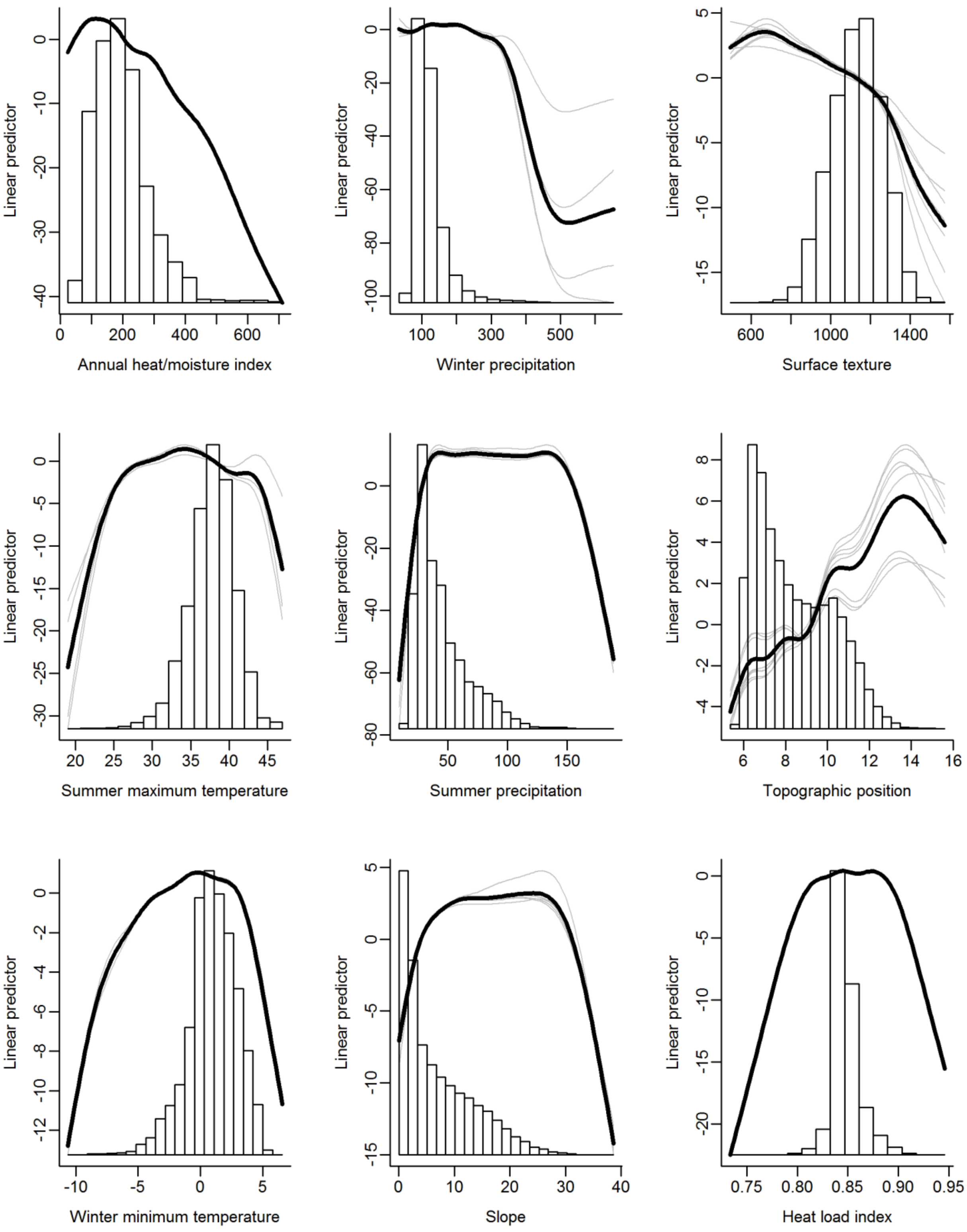

GAM response curves for *Penstemon palmeri*

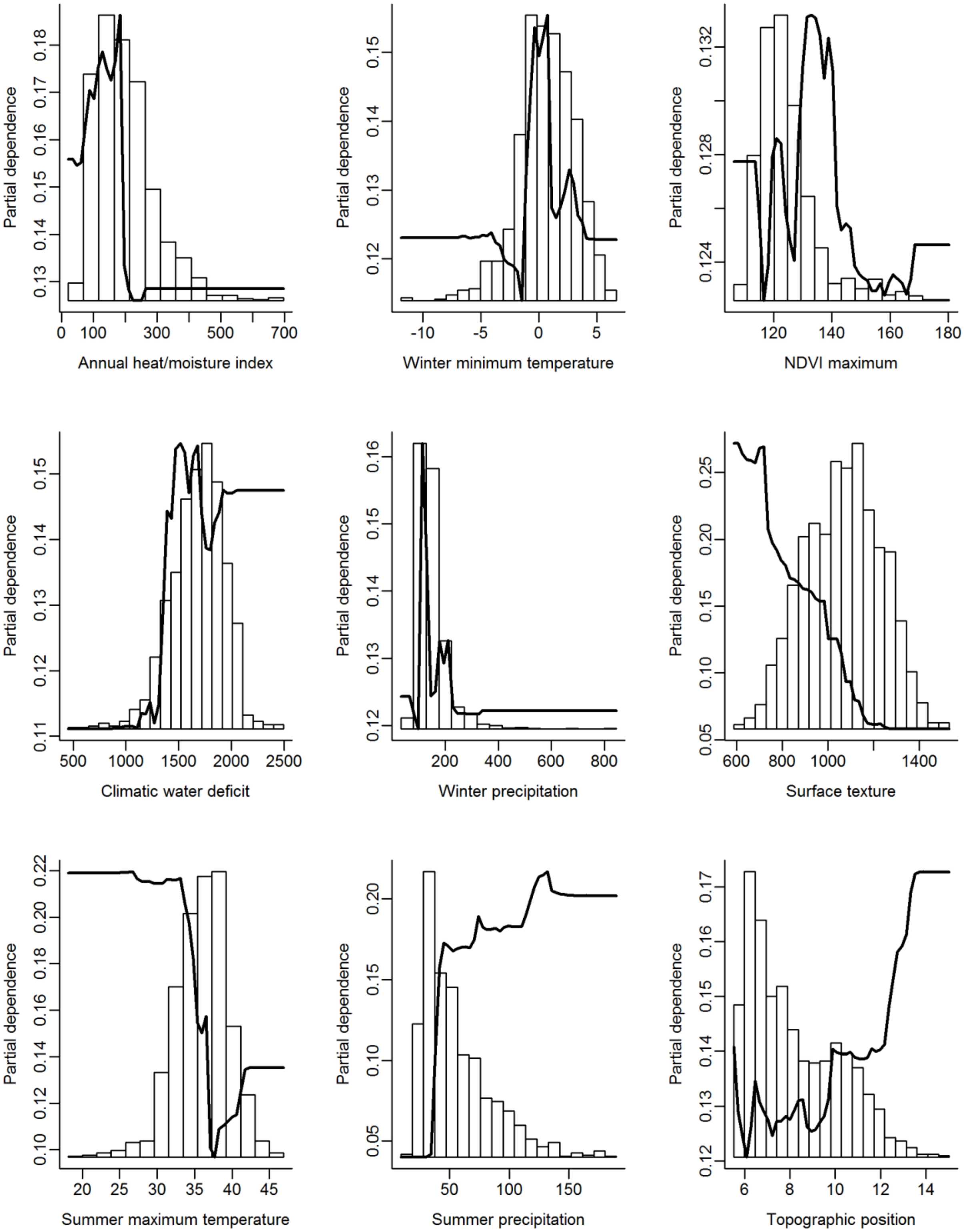

Random Forest response curves for *Penstemon palmeri*

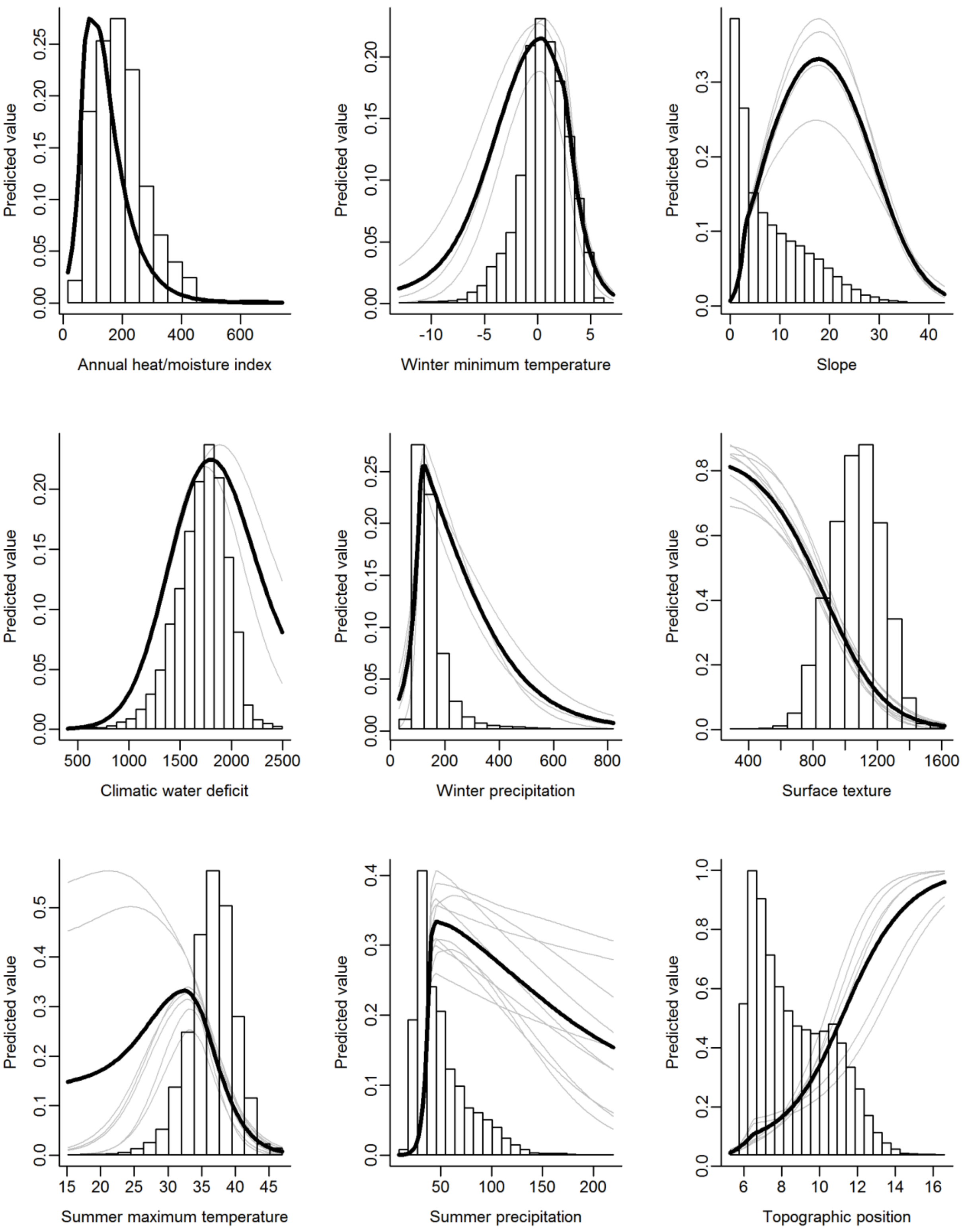

MaxEnt response curves for *Penstemon palmeri*

41. *Plantago ovata* SDM and response curves (GAM, Random Forest, Maxent)

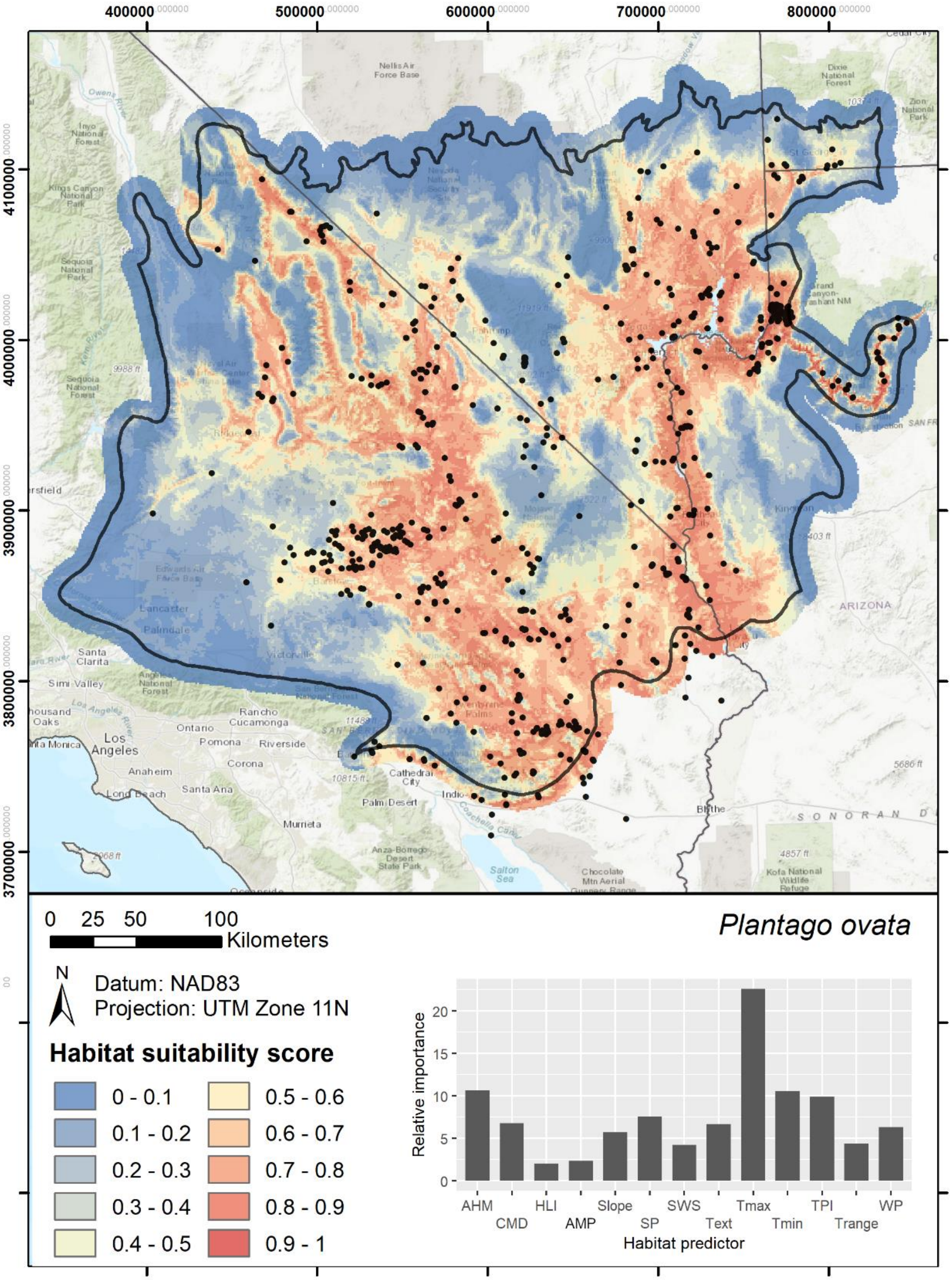

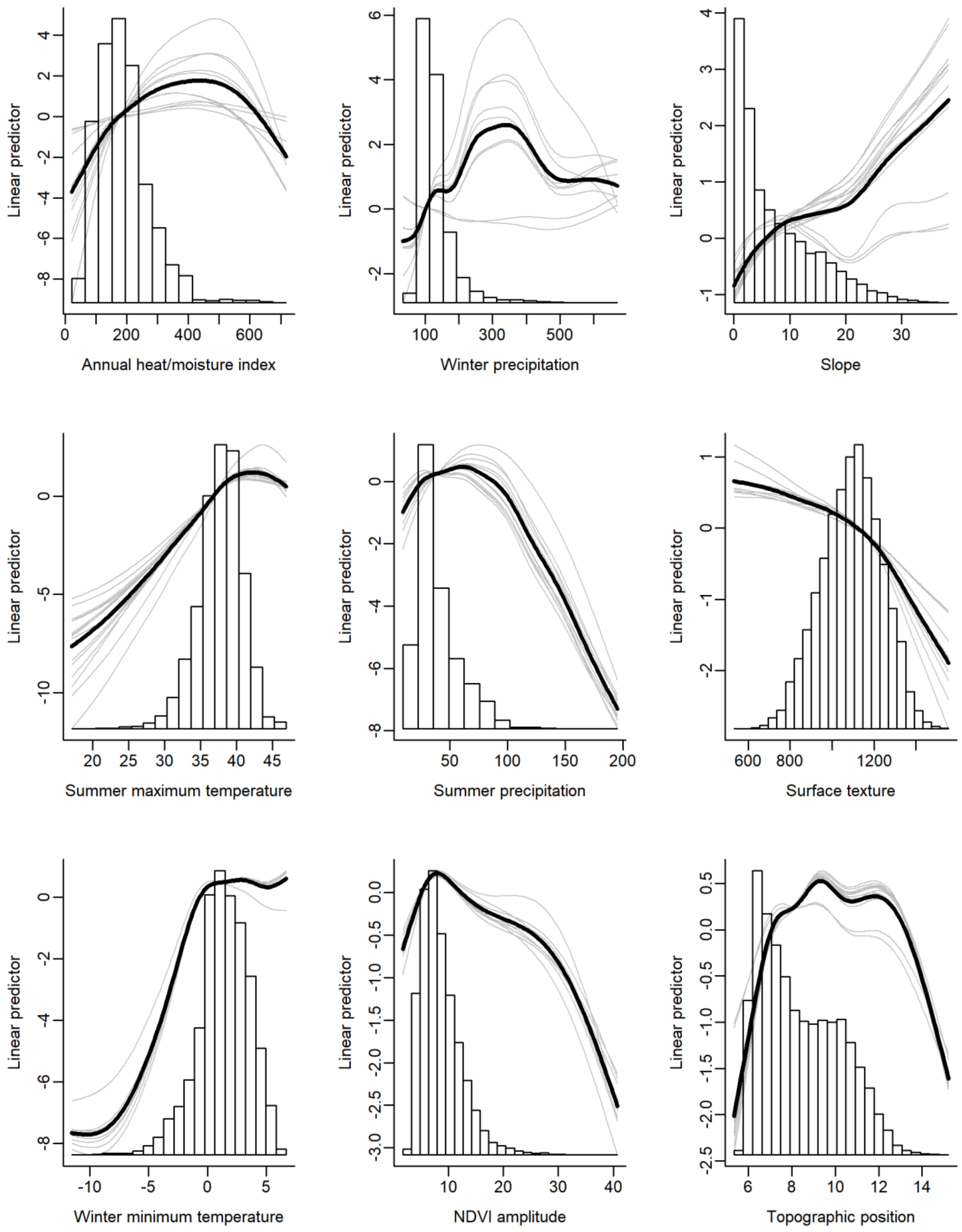

GAM response curves for *Plantago ovata*

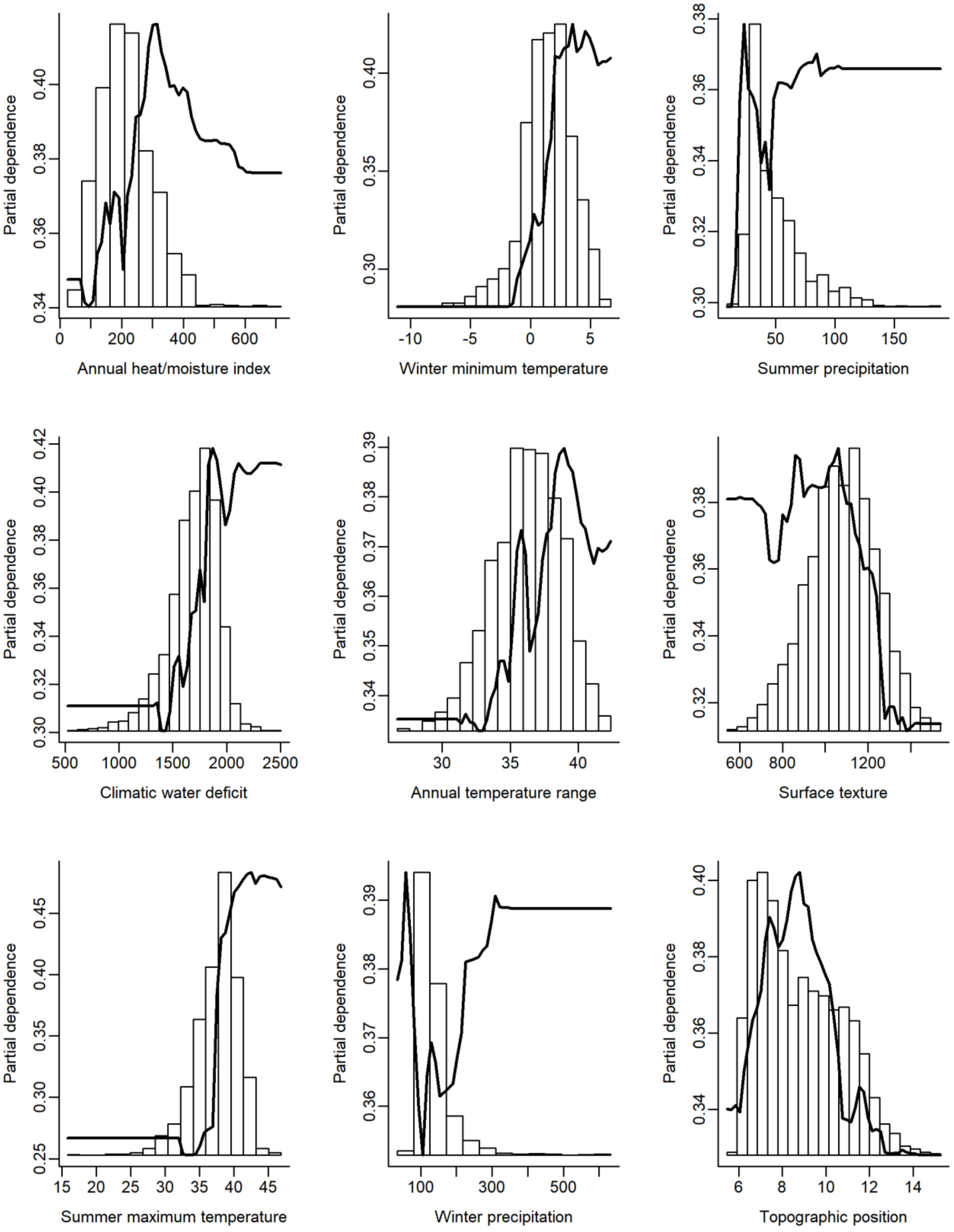

Random Forest response curves for *Plantago ovata*

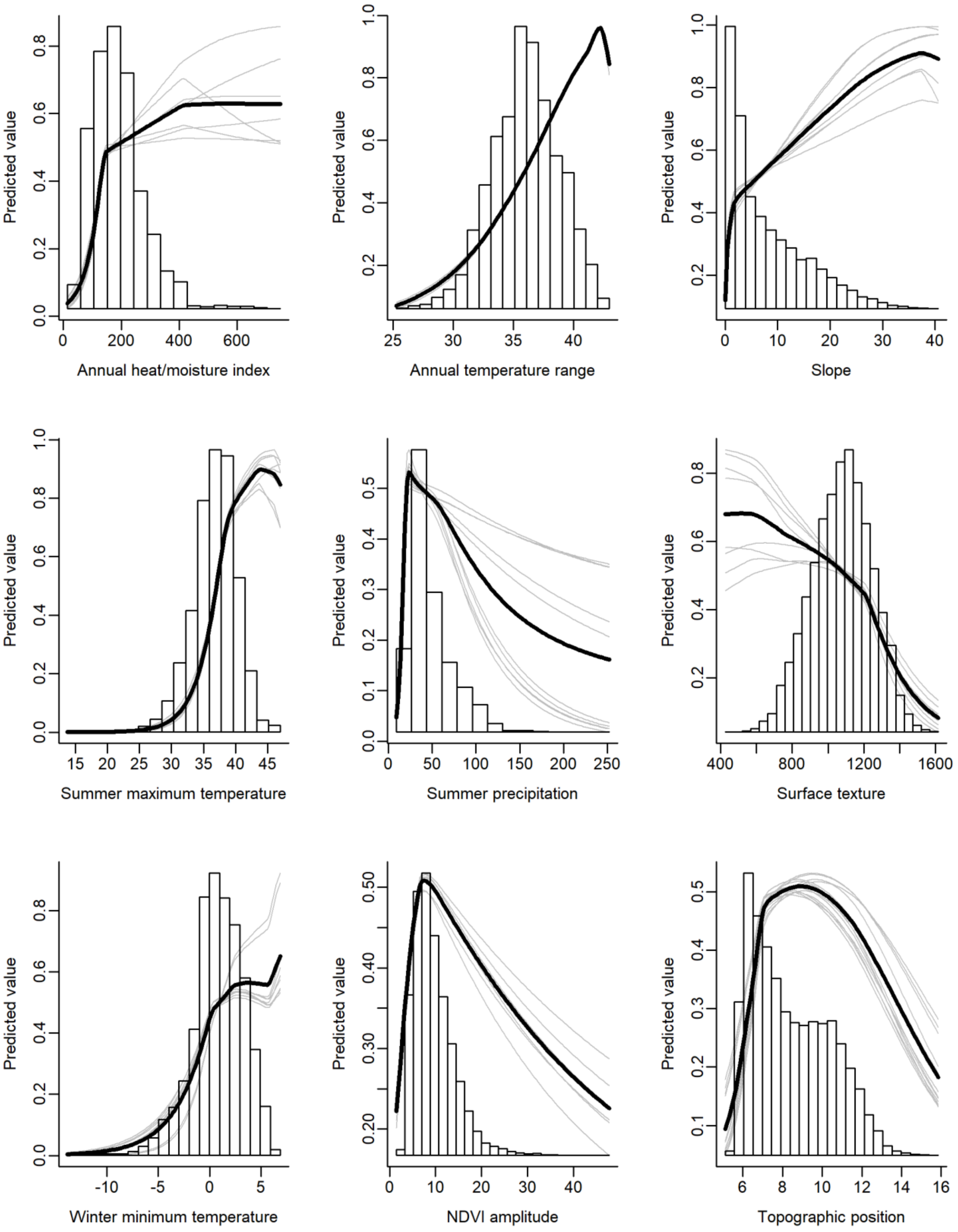

MaxEnt response curves for *Plantago ovata*

42. *Psorothamnus fremontii* SDM and response curves (GAM, Random Forest, Maxent)

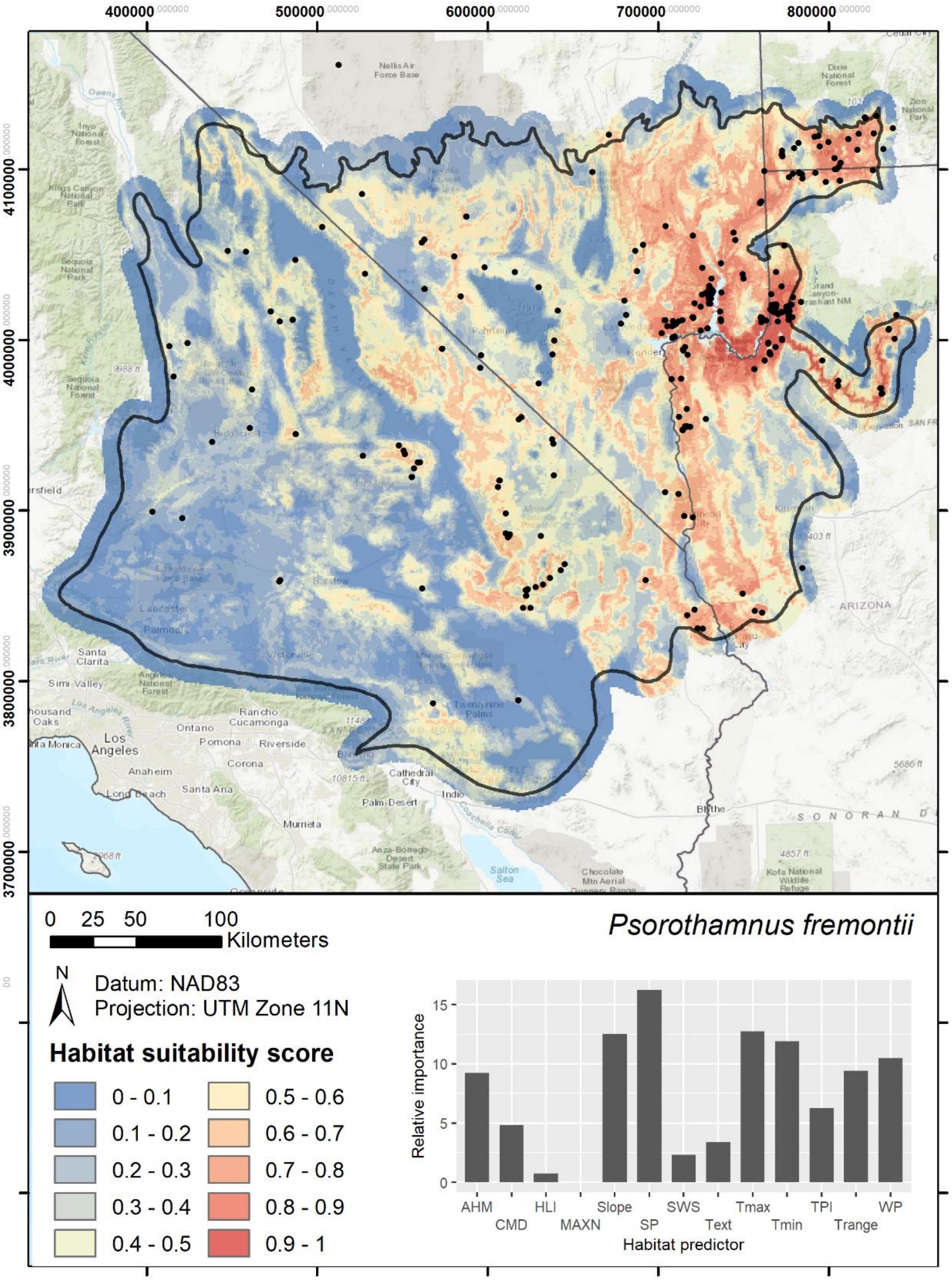

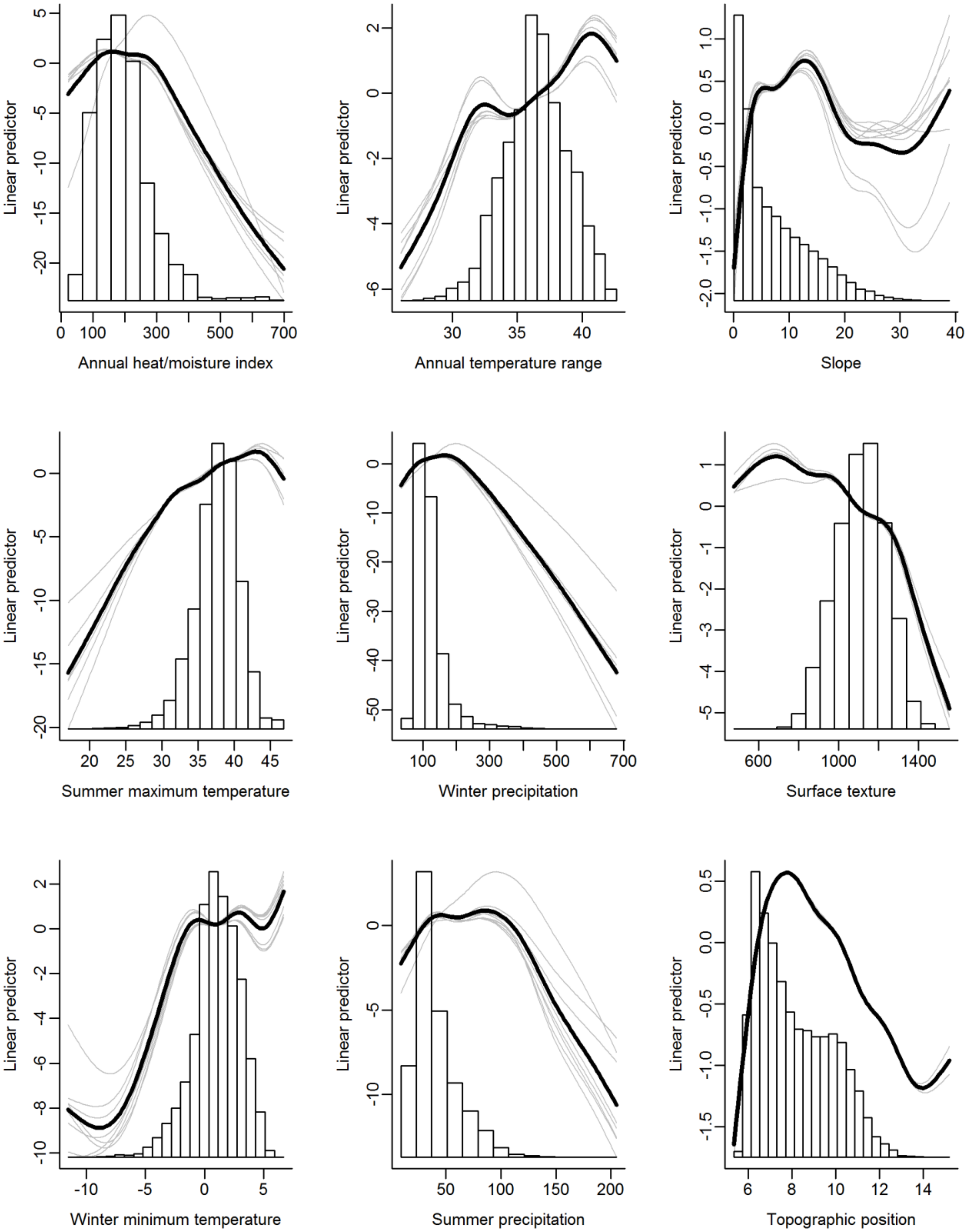

GAM response curves for *Psorothamnus fremontii*

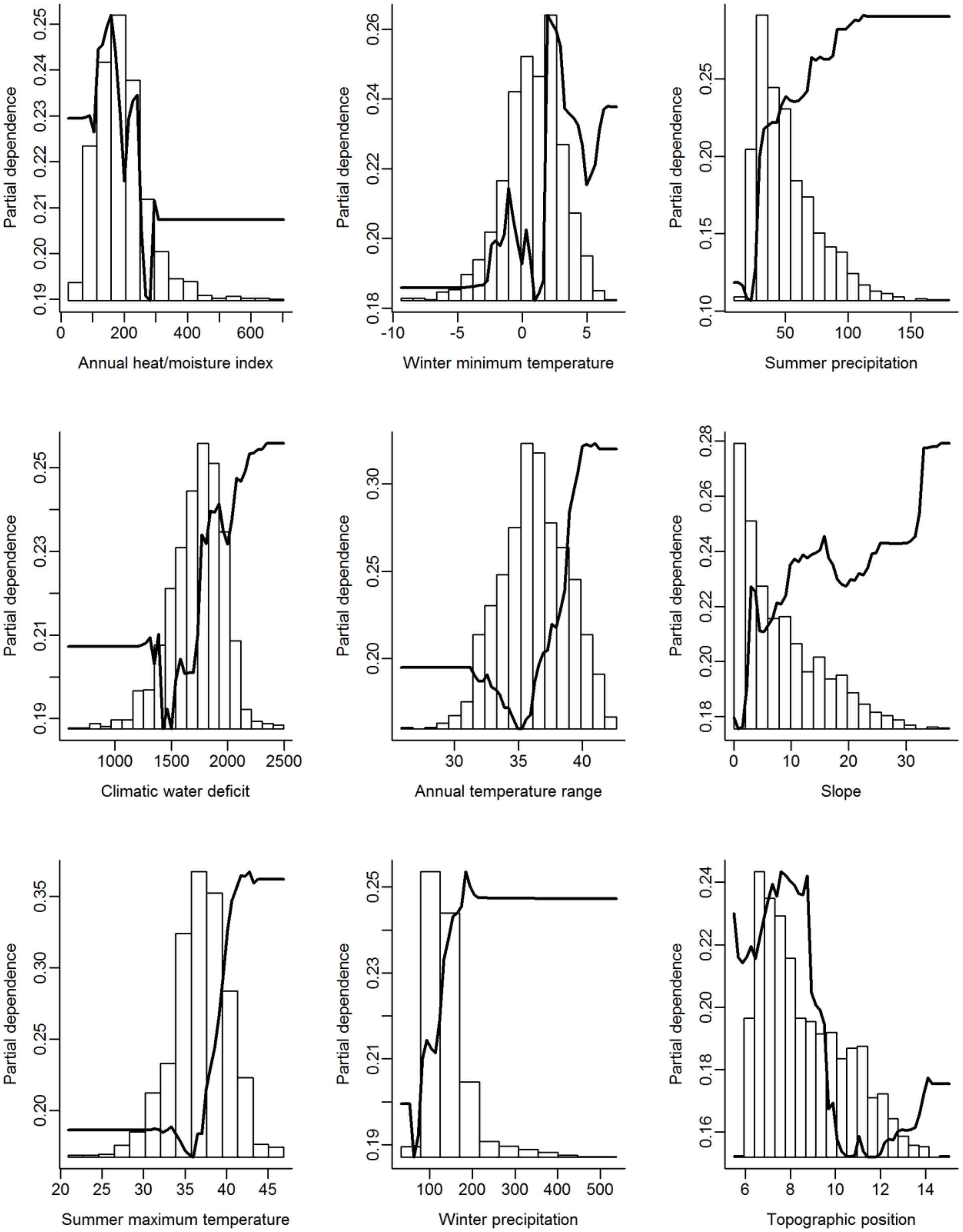

Random Forest response curves for *Psorothamnus fremontii*

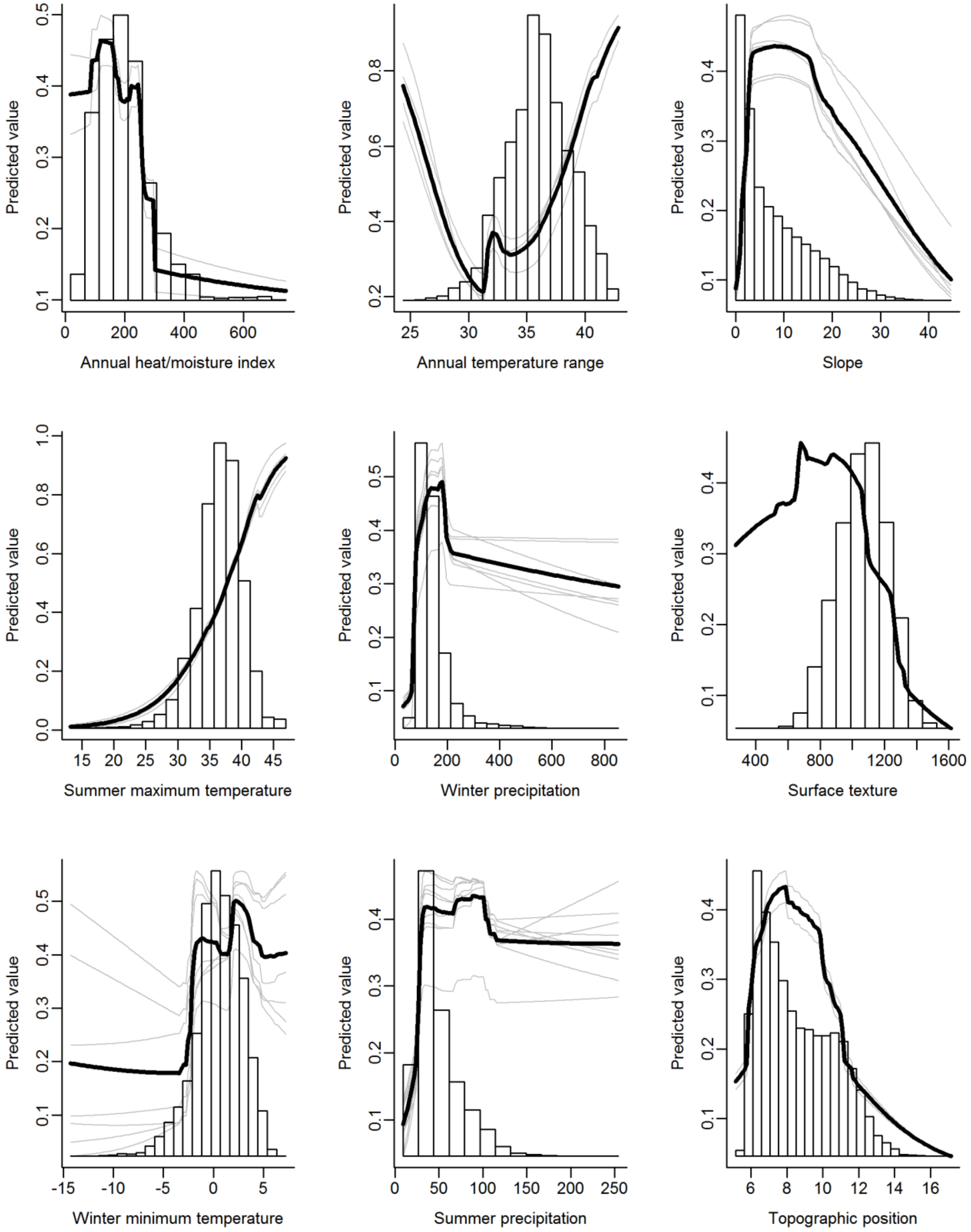

MaxEnt response curves for *Psorothamnus fremontii*

43. *Salvia columbariae* SDM and response curves (GAM, Random Forest, Maxent)

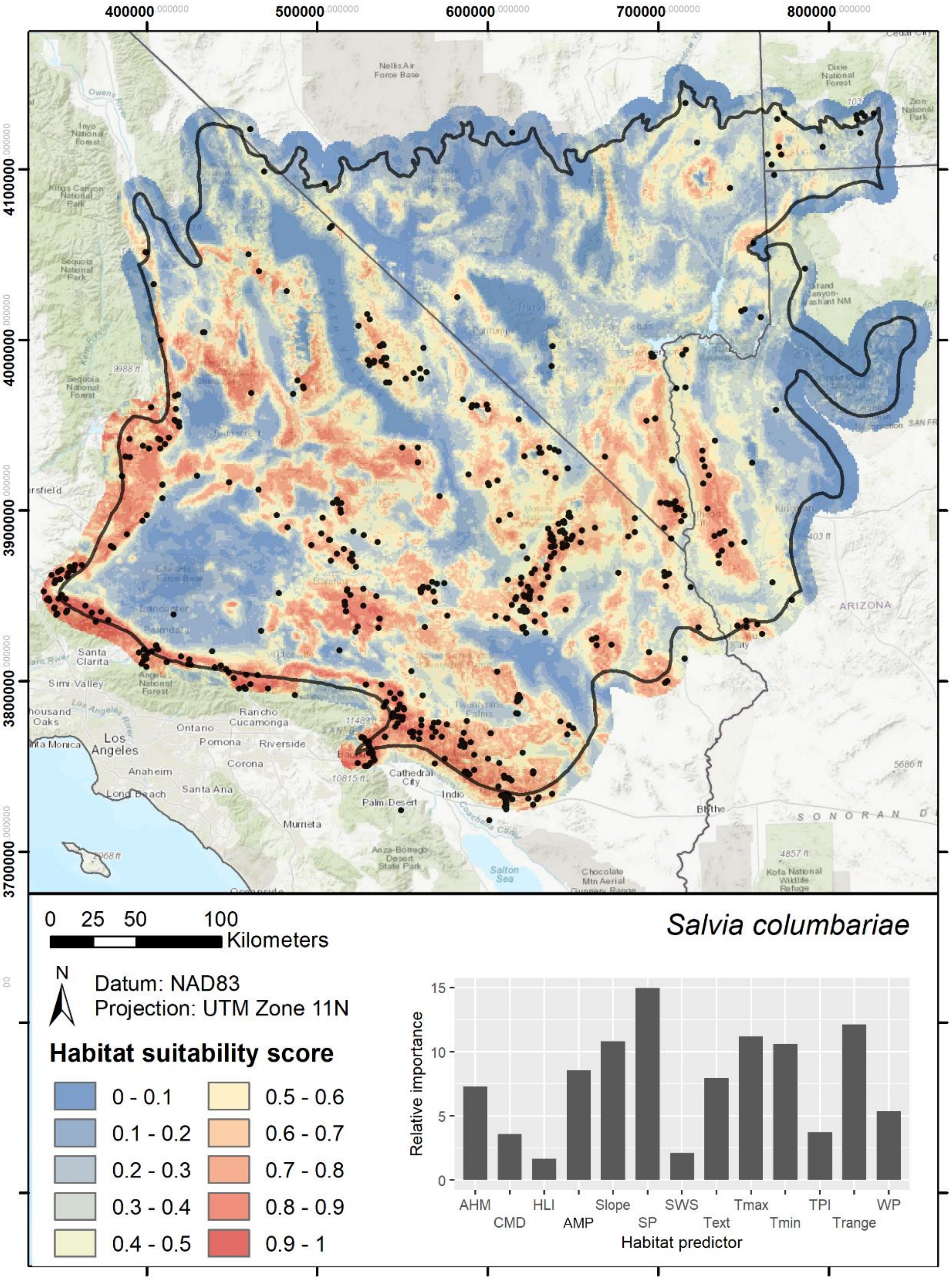

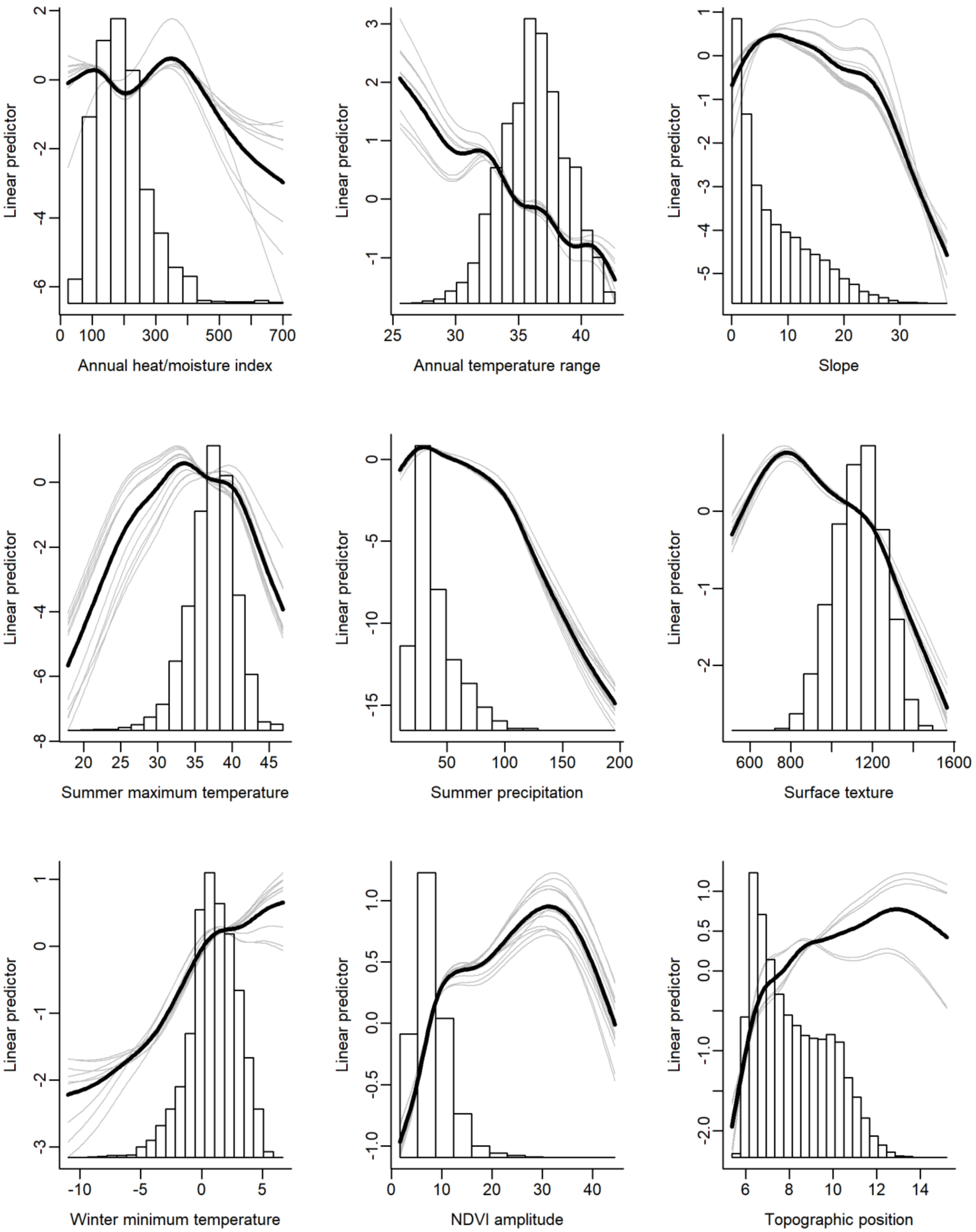

GAM response curves for *Salvia columbariae*

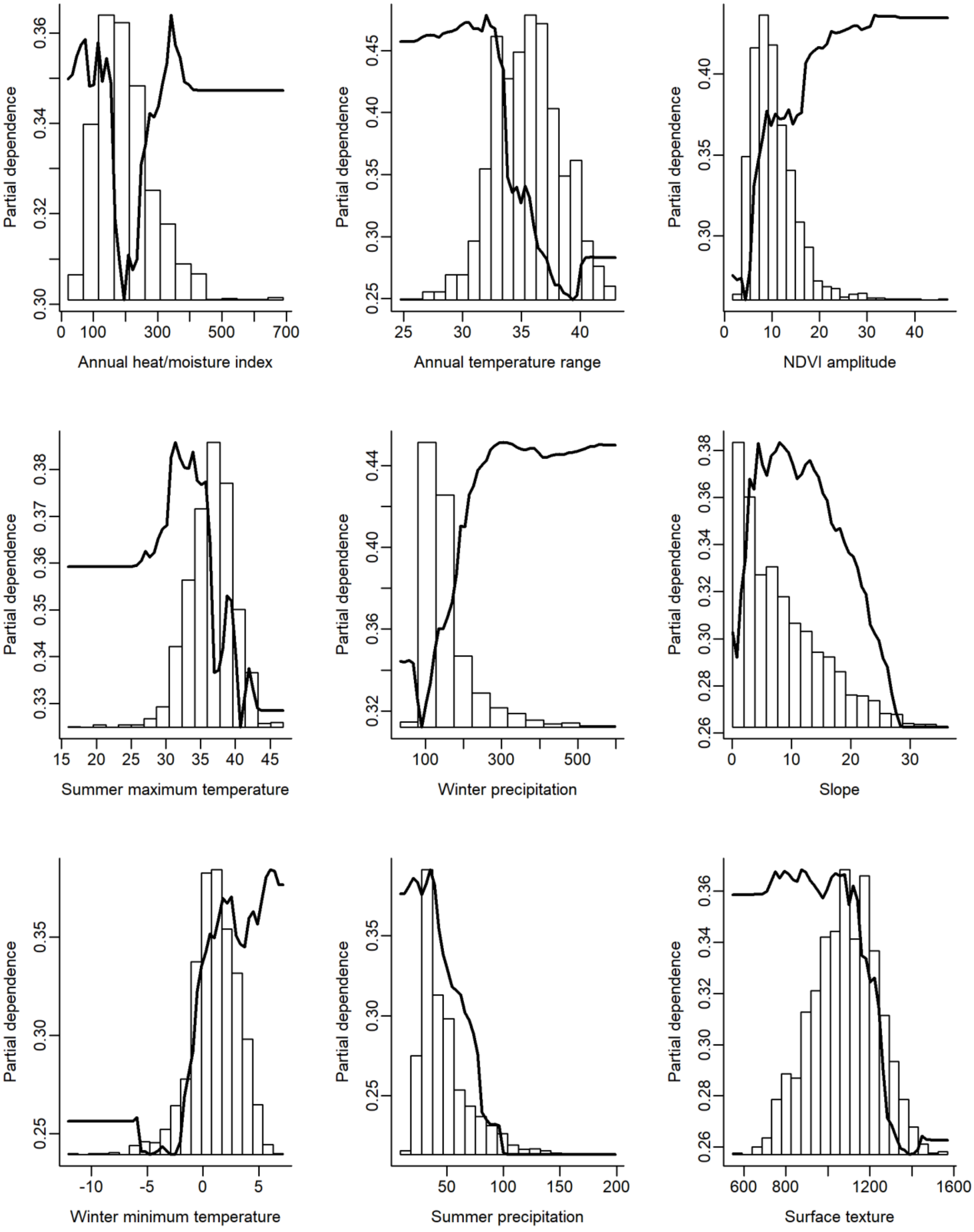

Random Forest response curves for *Salvia columbariae*

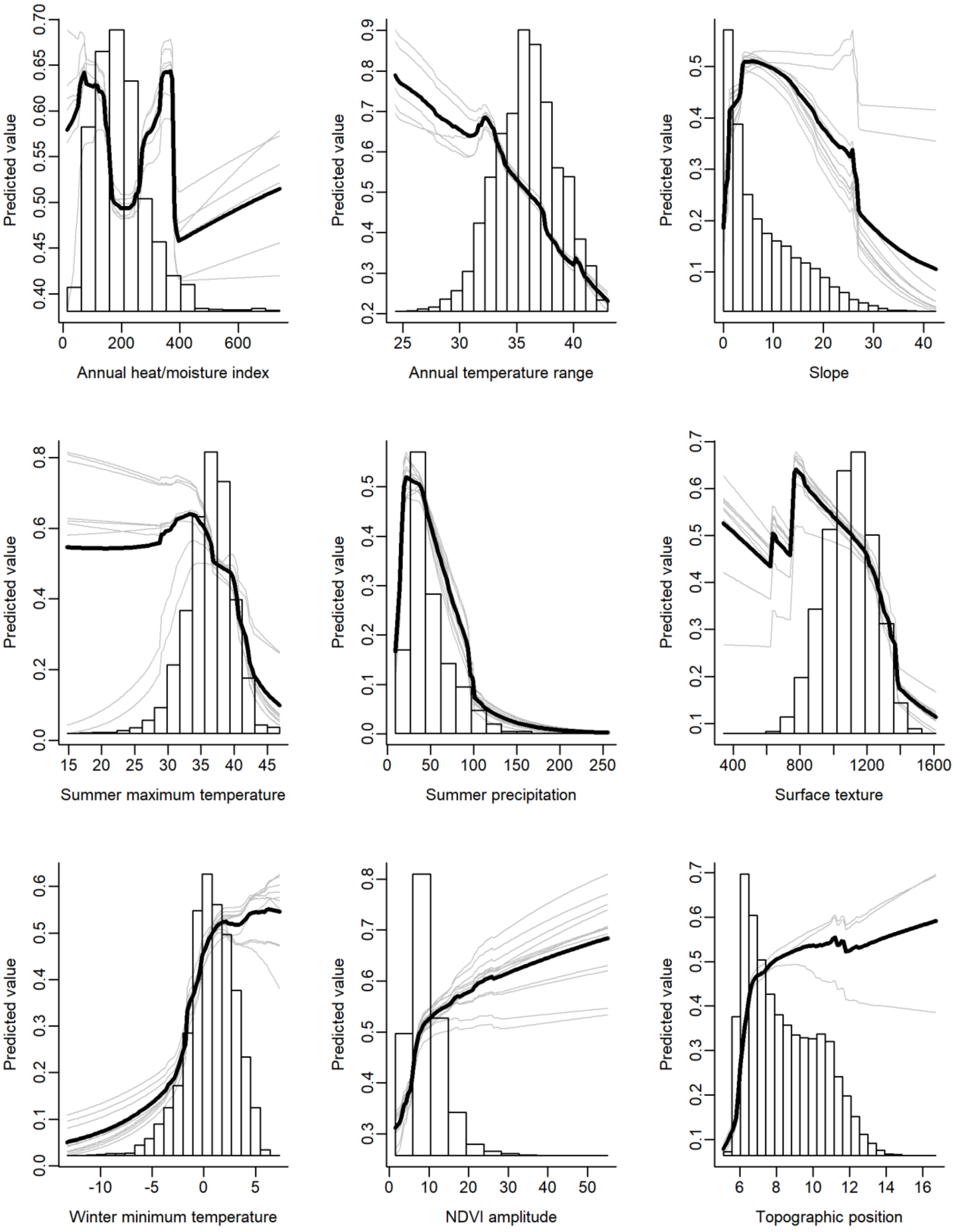

MaxEnt response curves for *Salvia columbariae*

44. *Sphaeralcea ambigua* SDM and response curves (GAM, Random Forest, Maxent)

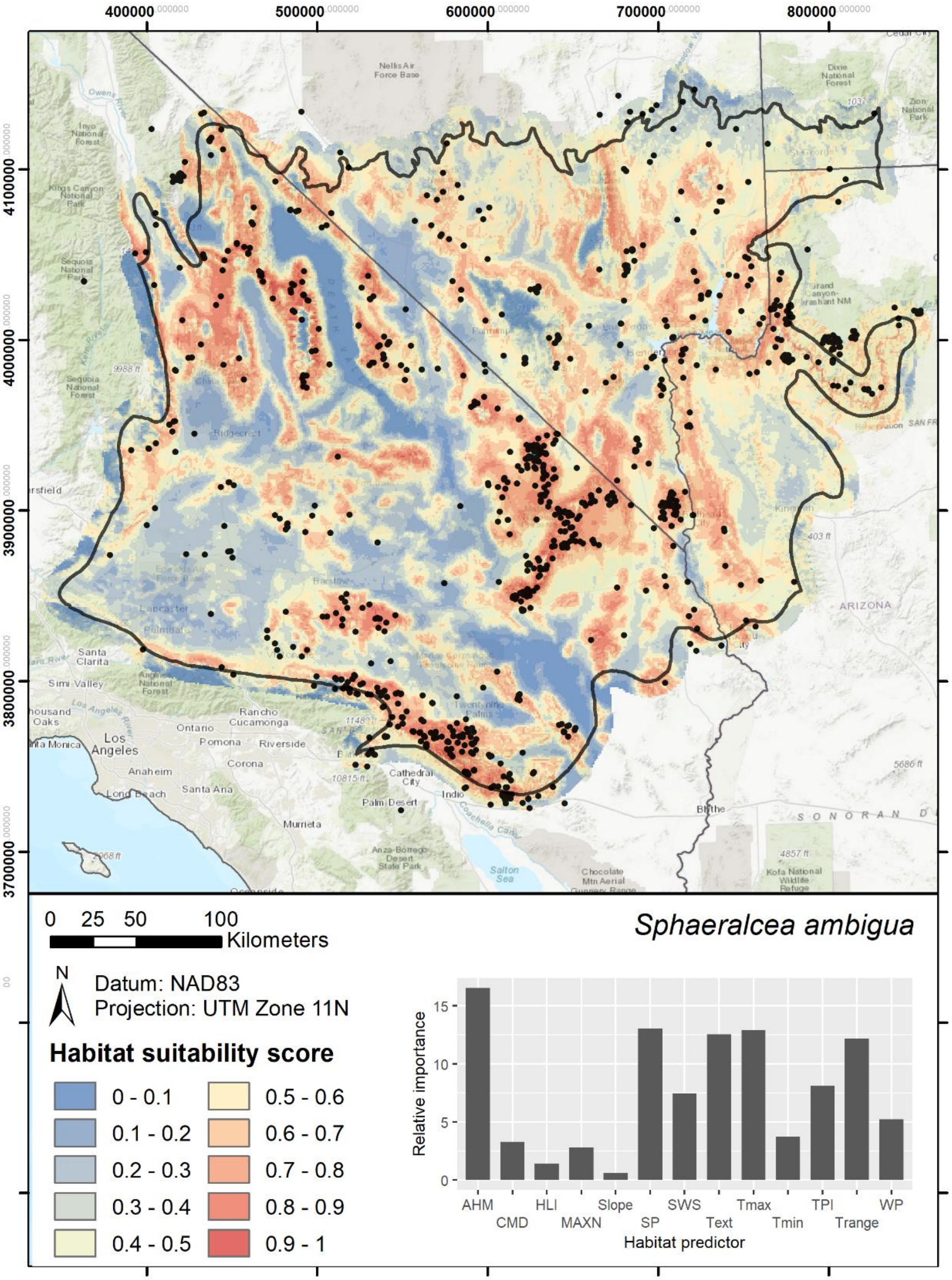

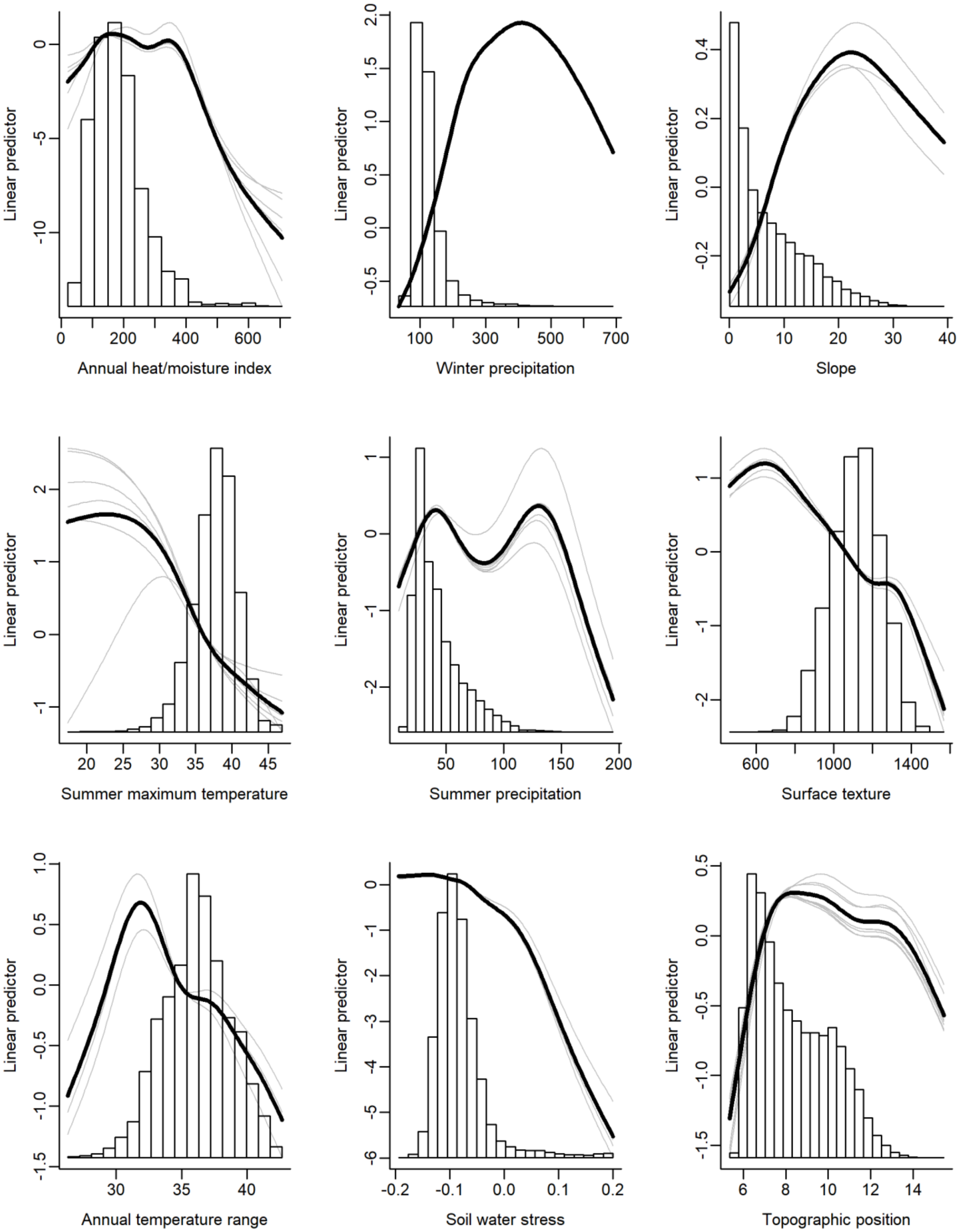

GAM response curves for *Sphaeralcea ambigua*

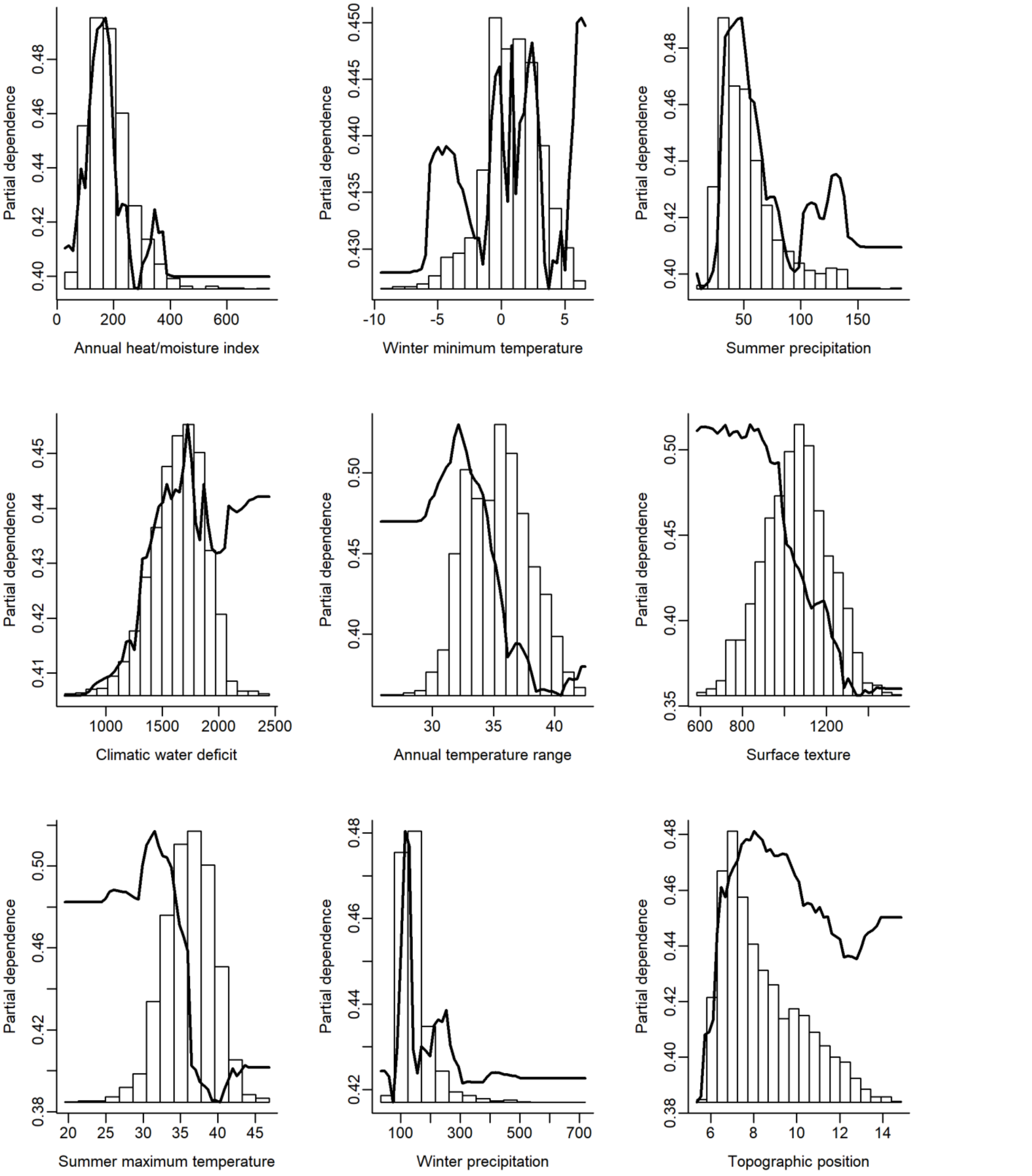

Random Forest response curves for *Sphaeralcea ambigua*

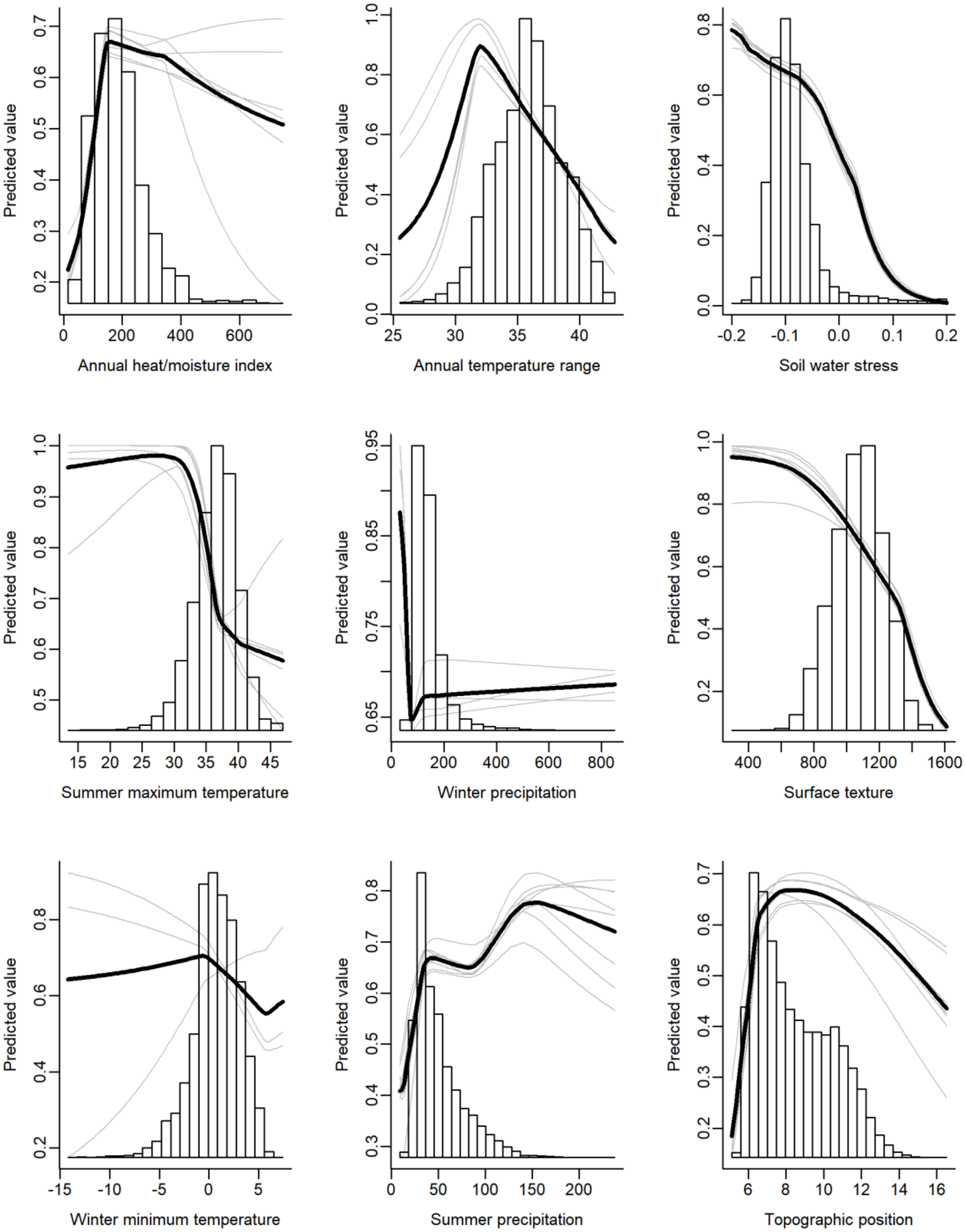

MaxEnt response curves for *Sphaeralcea ambigua*

45. *Stephanomeria exigua* SDM and response curves (GAM, Random Forest, Maxent)

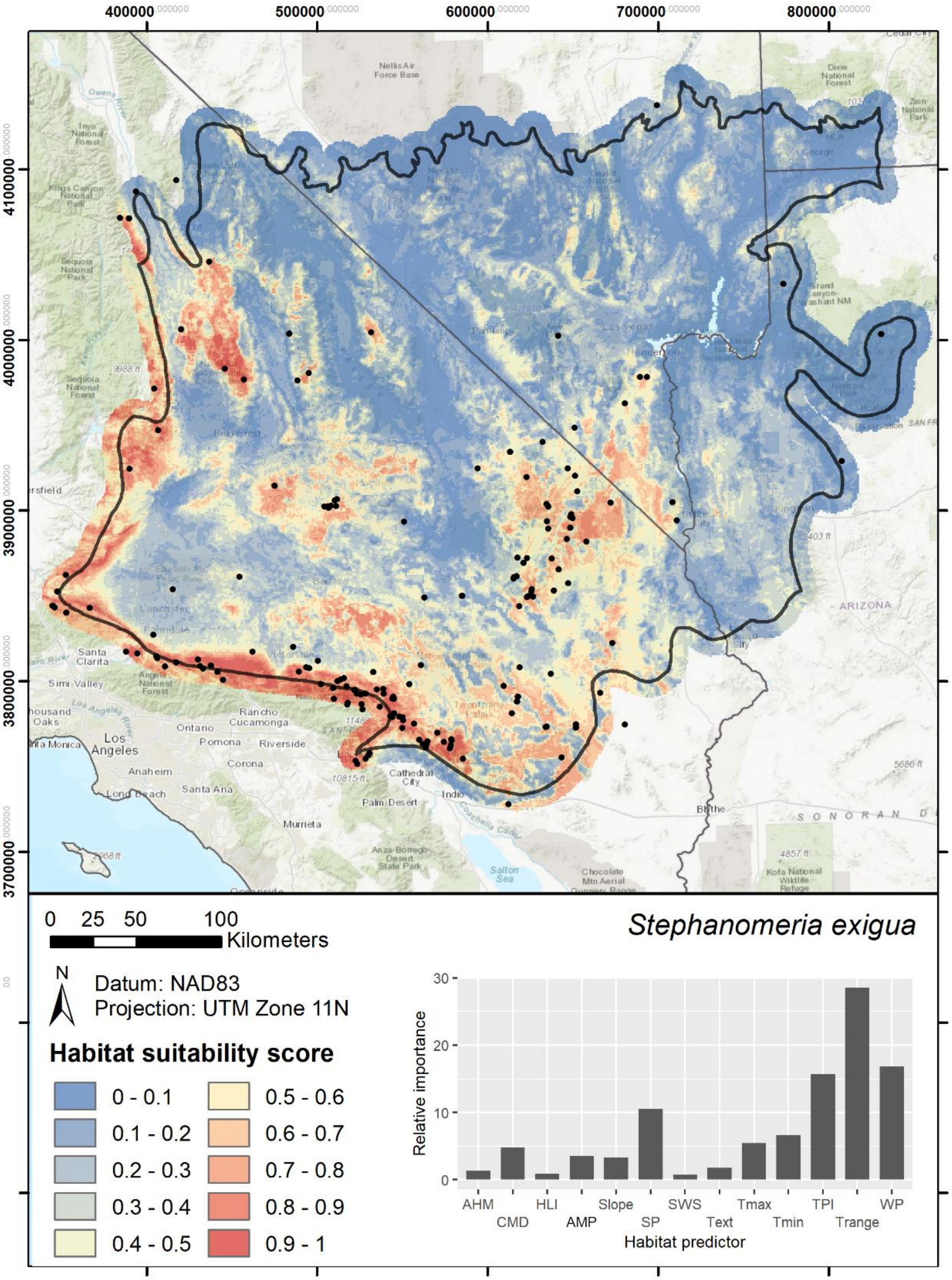

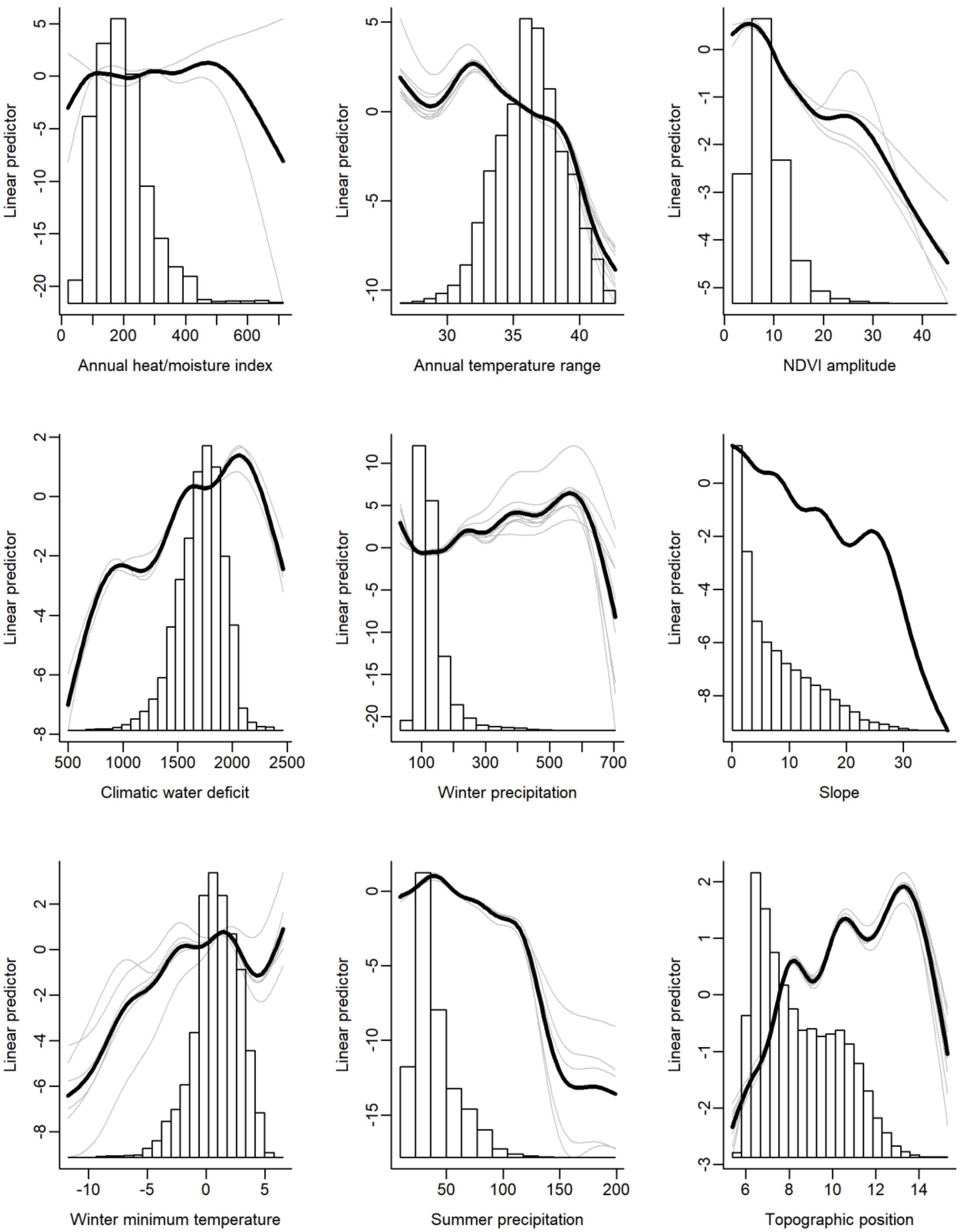

GAM response curves for *Stephanomeria exigua*

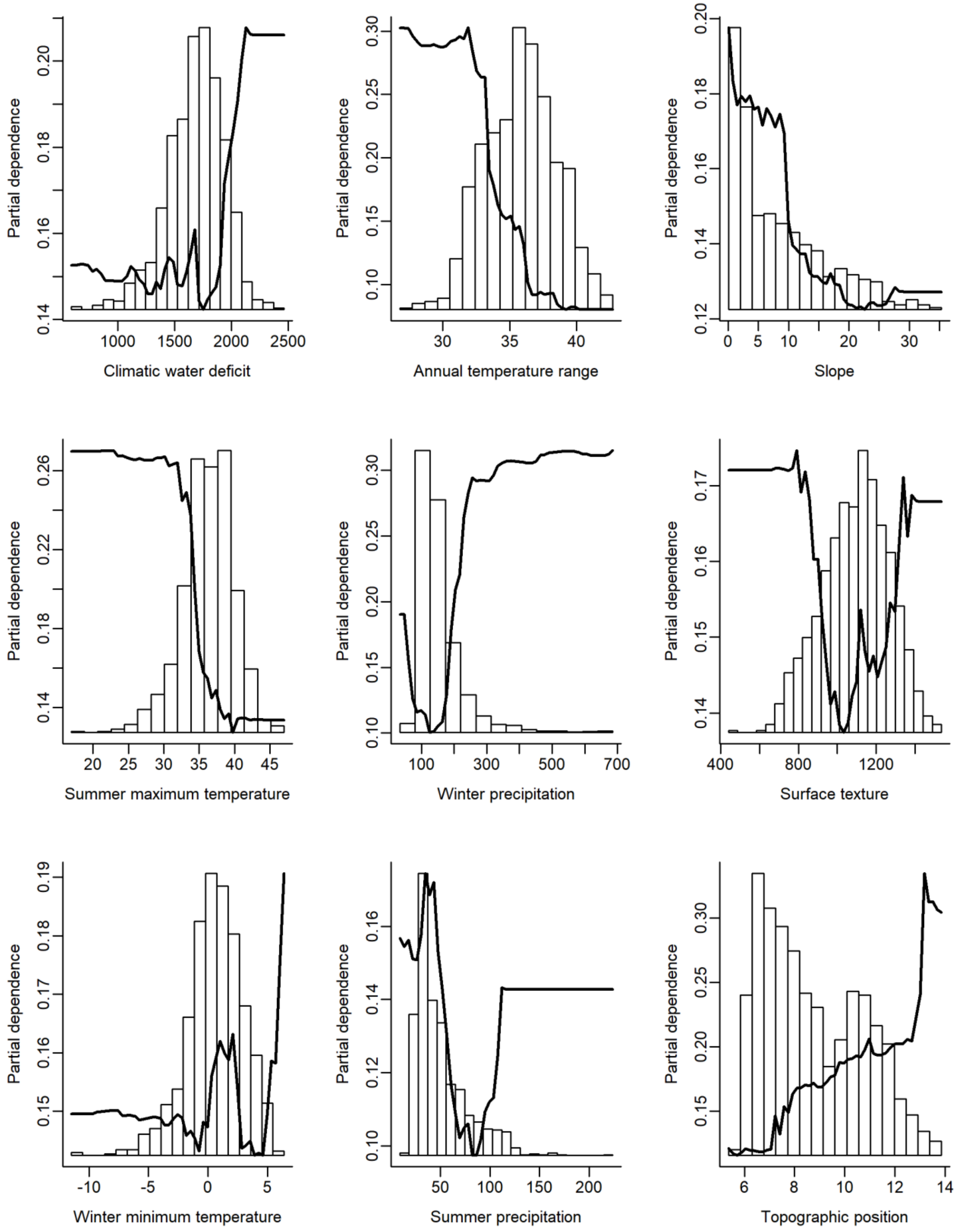

Random Forest response curves for *Stephanomeria exigua*

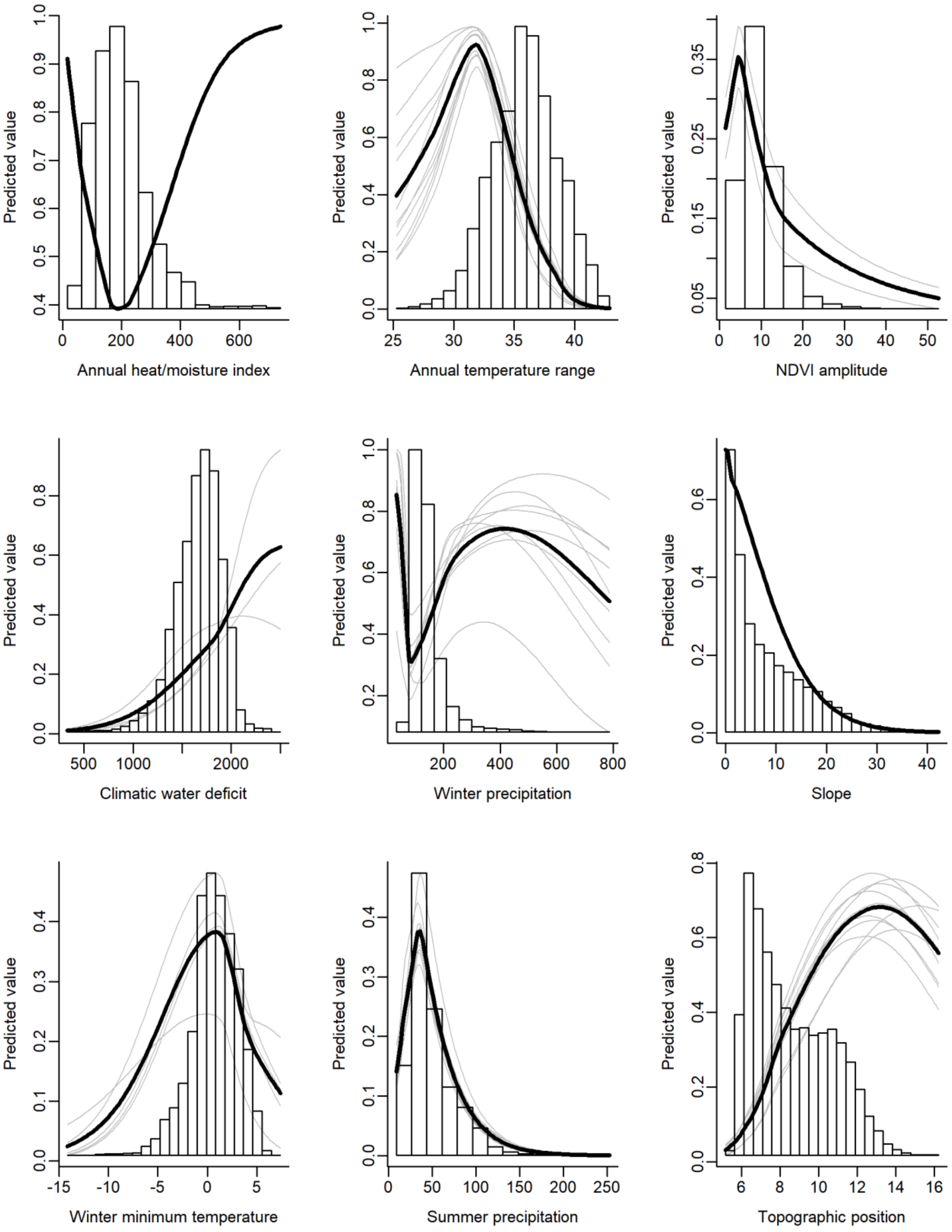

MaxEnt response curves for *Stephanomeria exigua*

46. *Stephanomeria parryi* SDM and response curves (GAM, Random Forest, Maxent)

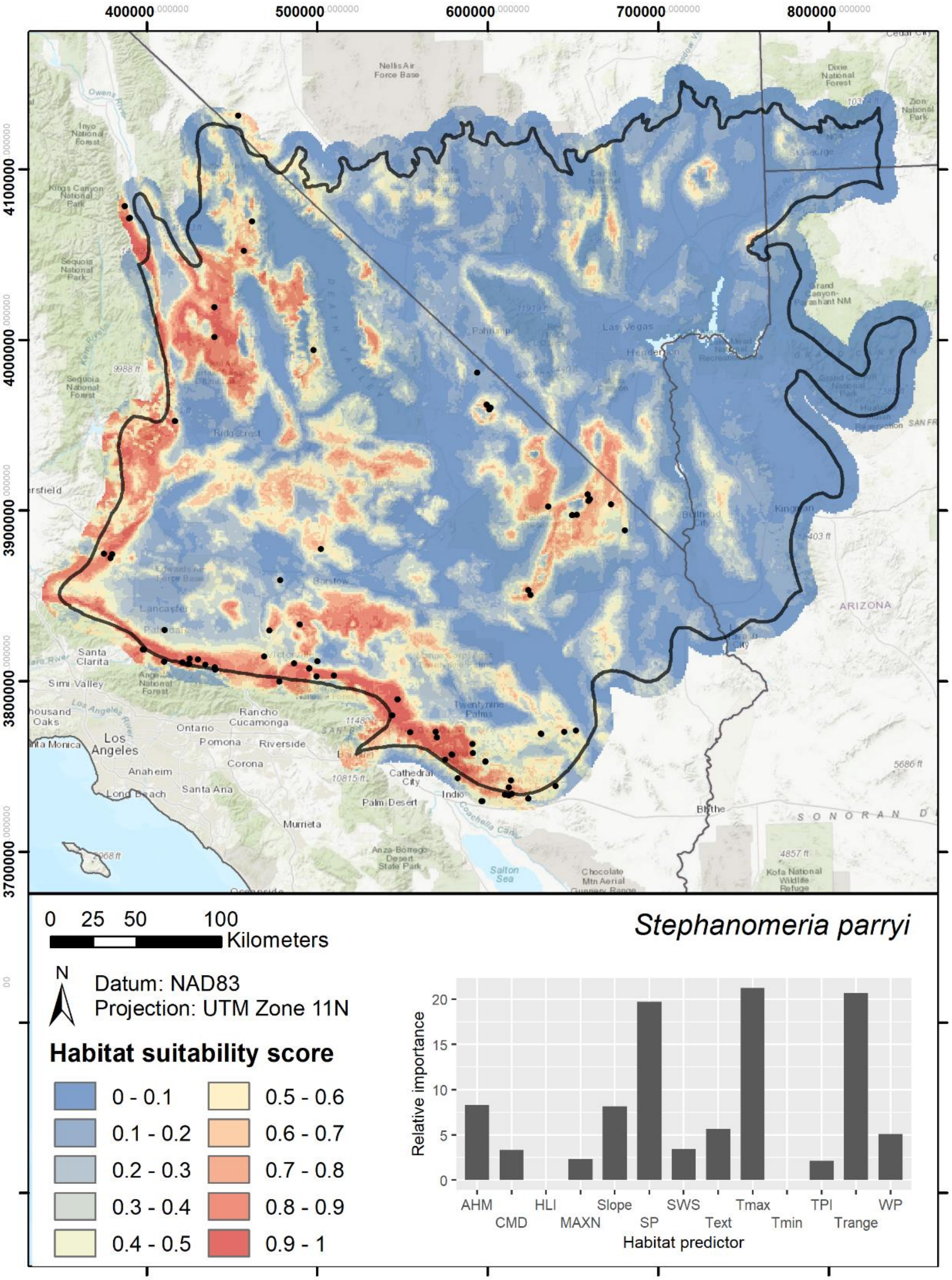

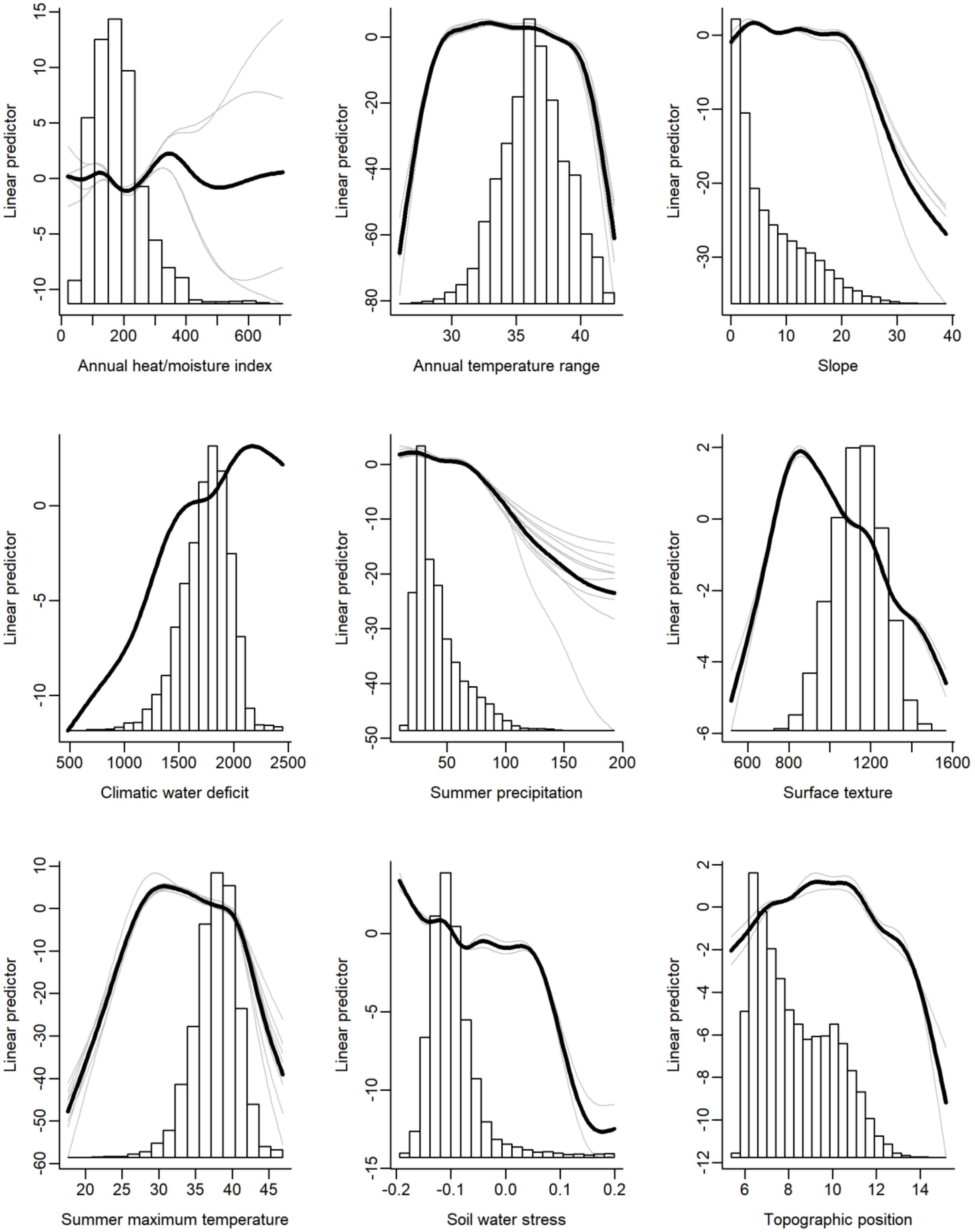

GAM response curves for *Stephanomeria parryi*

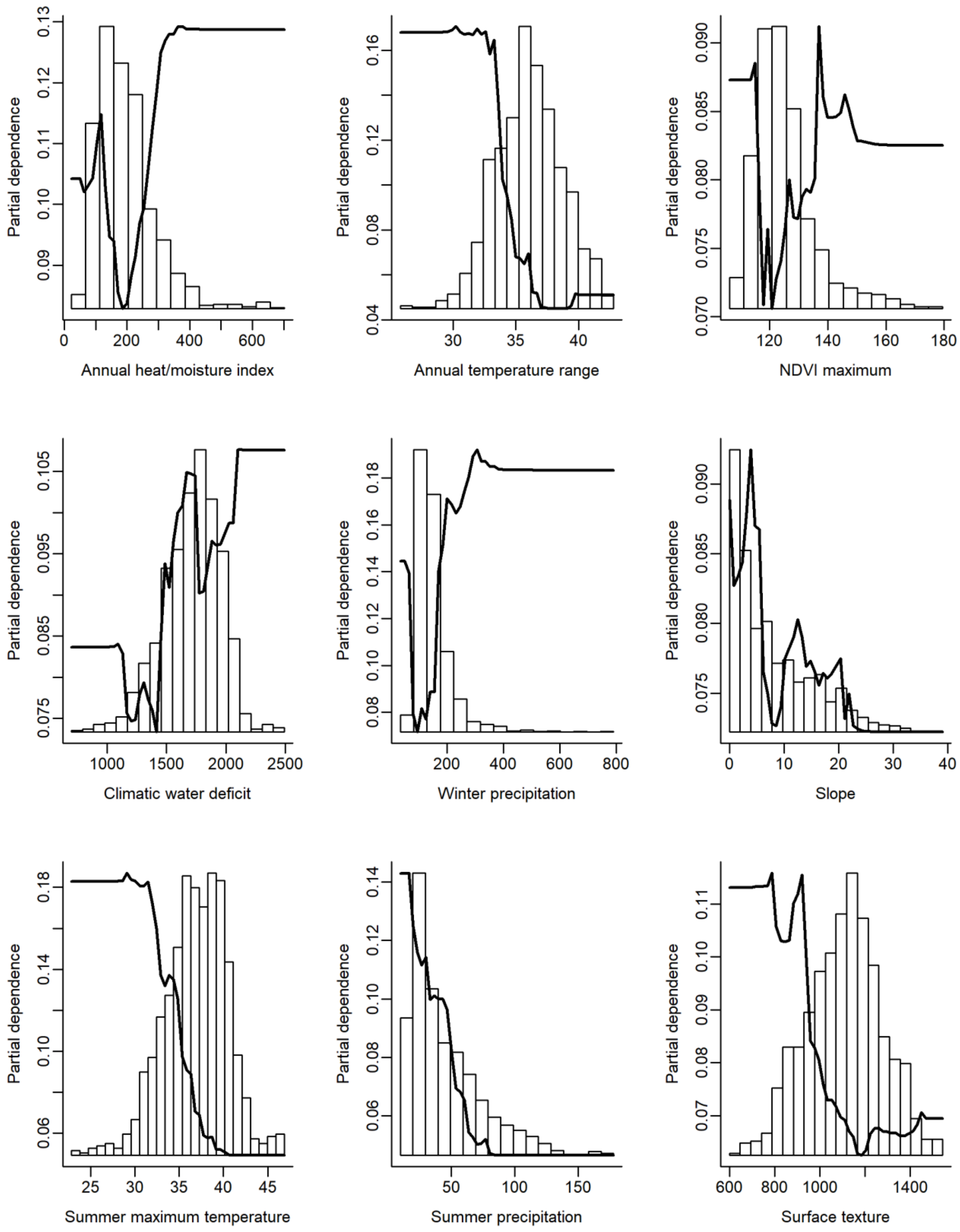

Random Forest response curves for *Stephanomeria parryi*

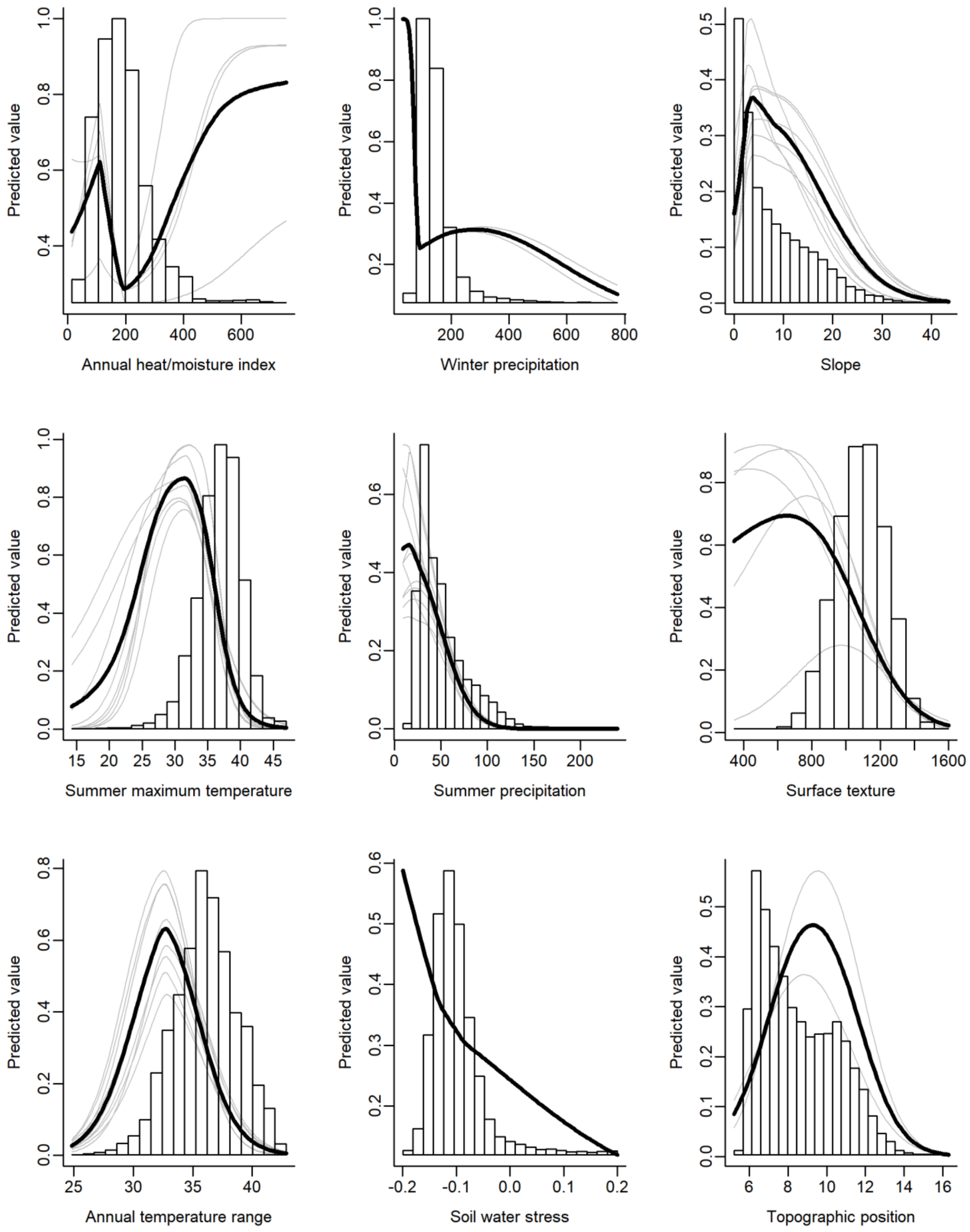

MaxEnt response curves for *Stephanomeria parryi*

47. *Vulpia octoflora* SDM and response curves (GAM, Random Forest, Maxent)

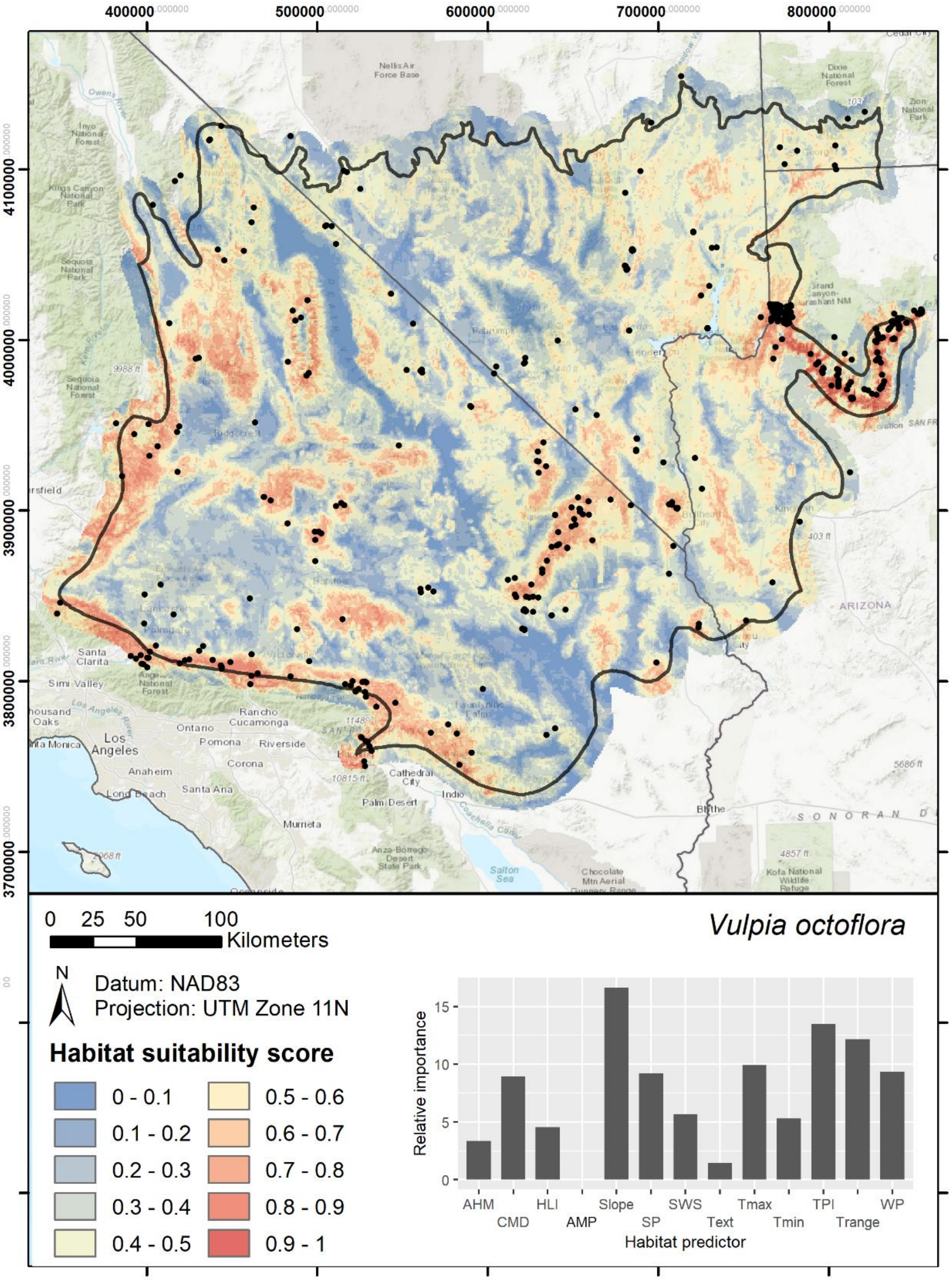

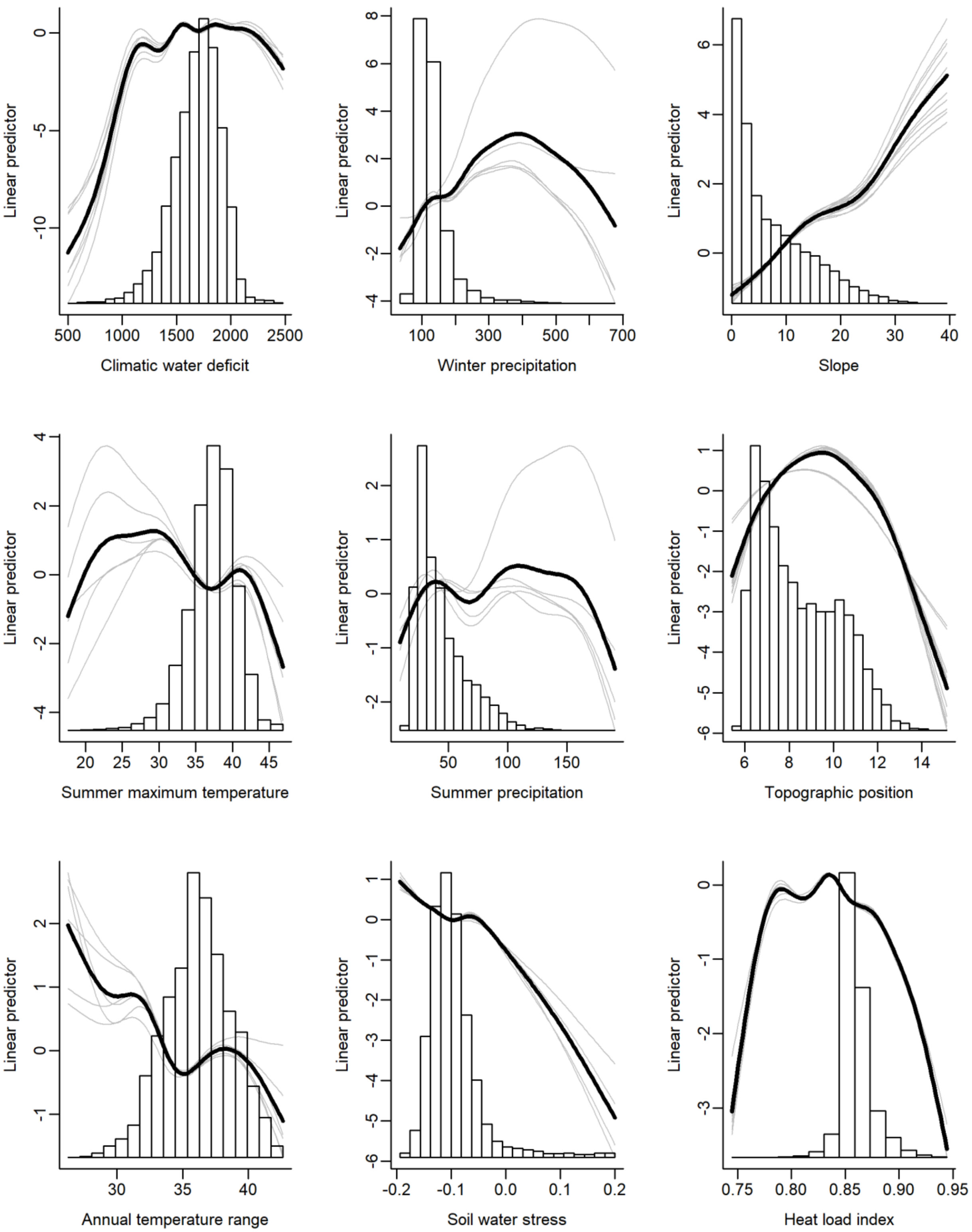

GAM response curves for *Vulpia octoflora*

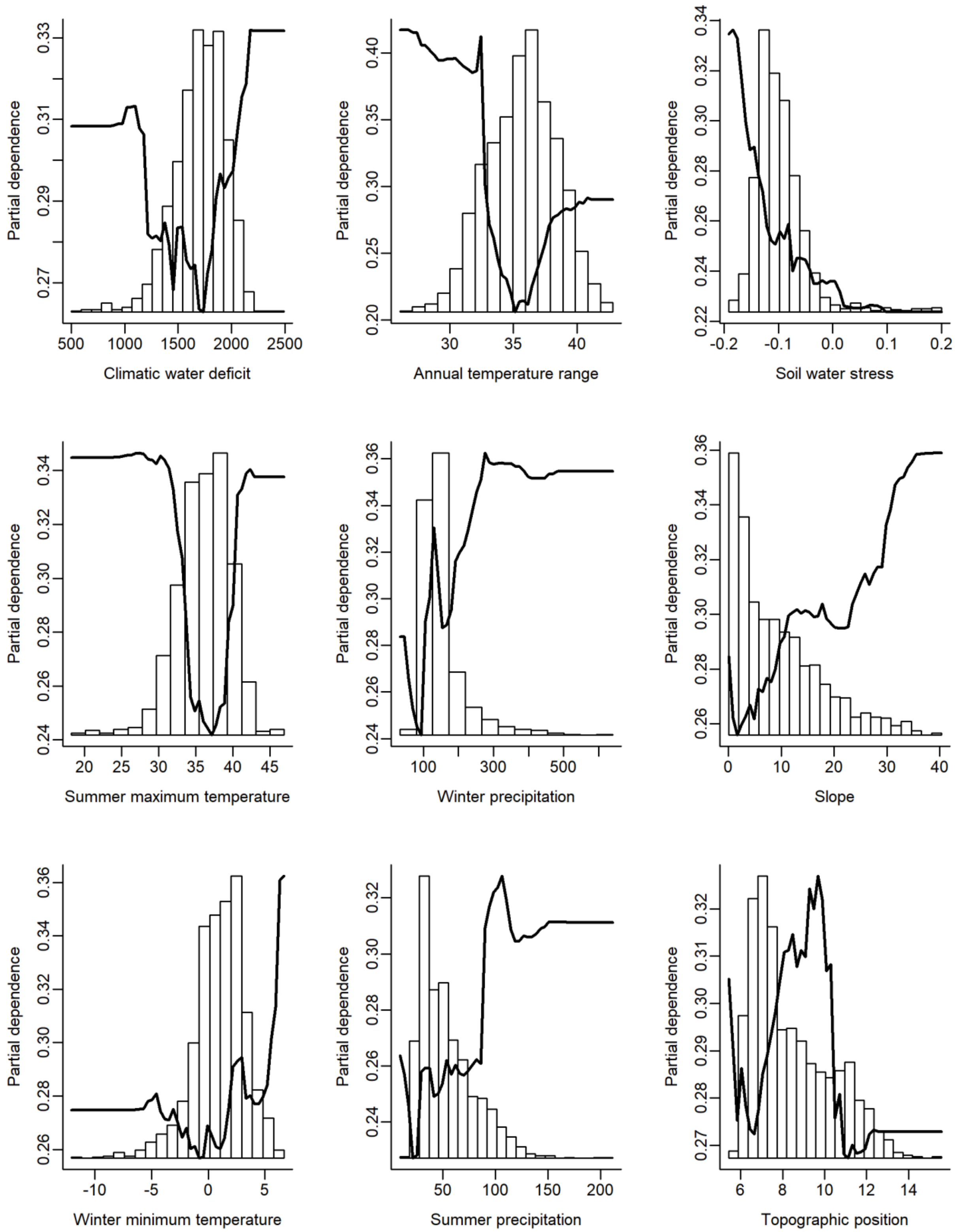

Random Forest response curves for *Vulpia octoflora*

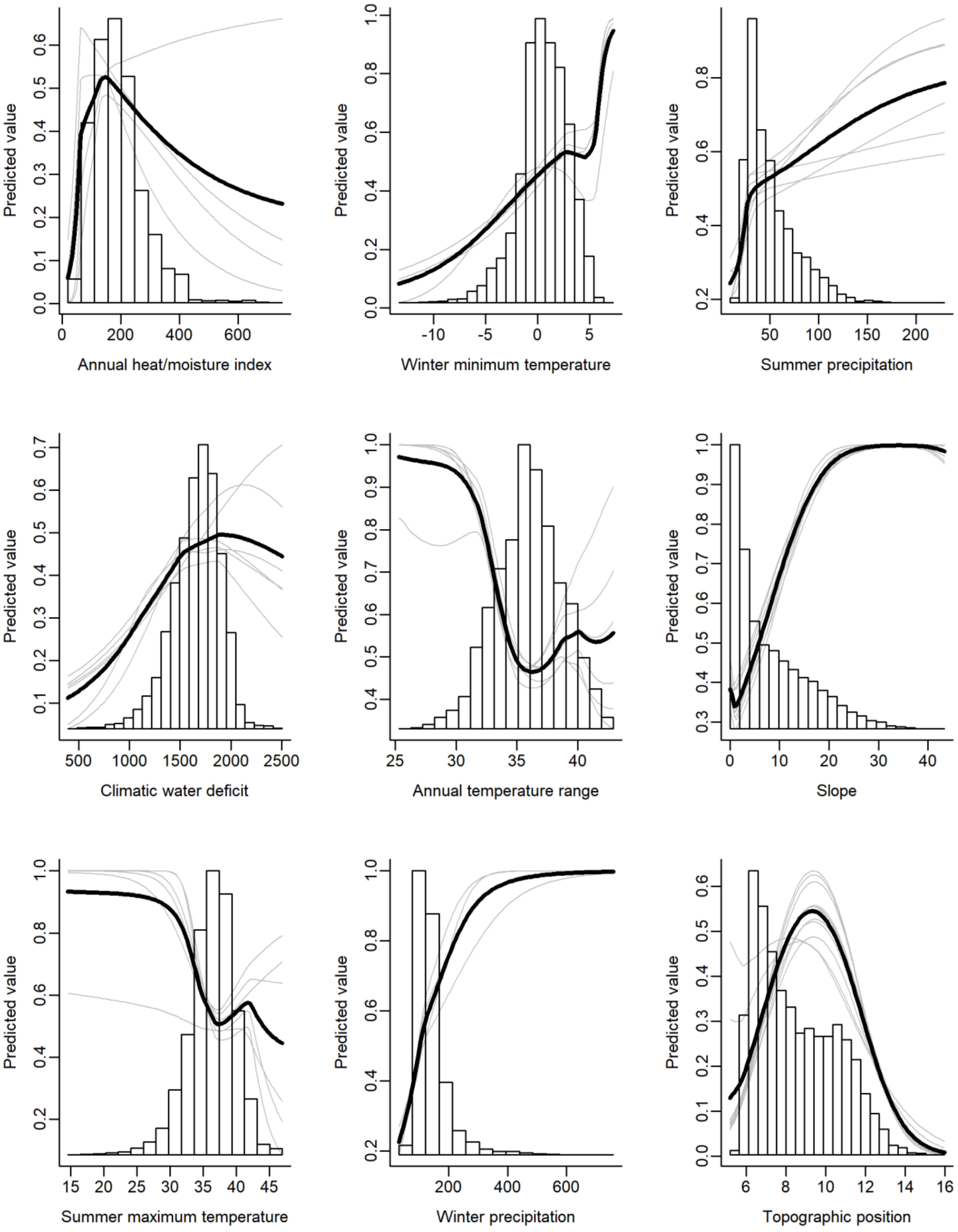

MaxEnt response curves for *Vulpia octoflora*

48. *Yucca brevifolia* SDM and response curves (GAM, Random Forest, Maxent)

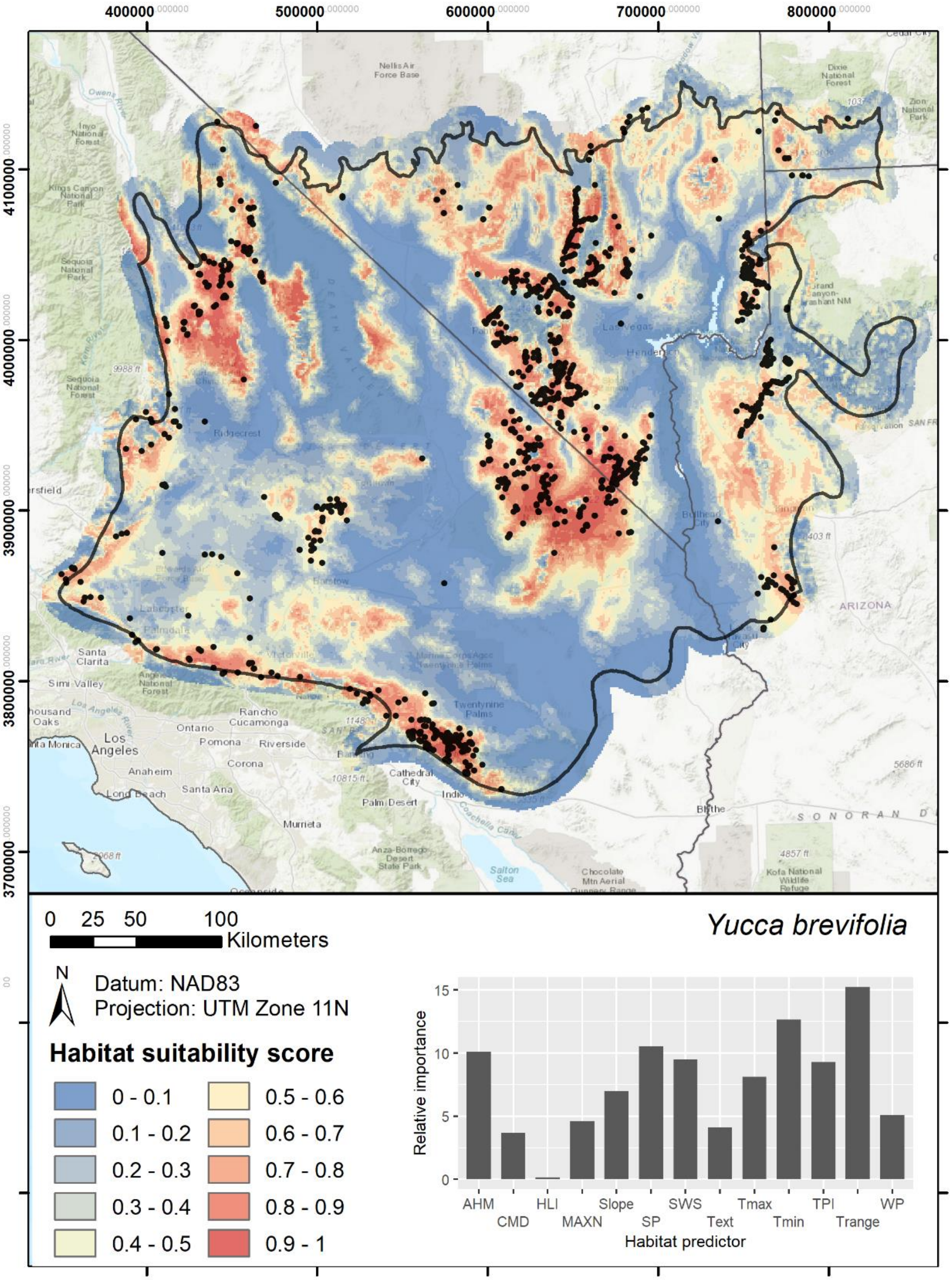

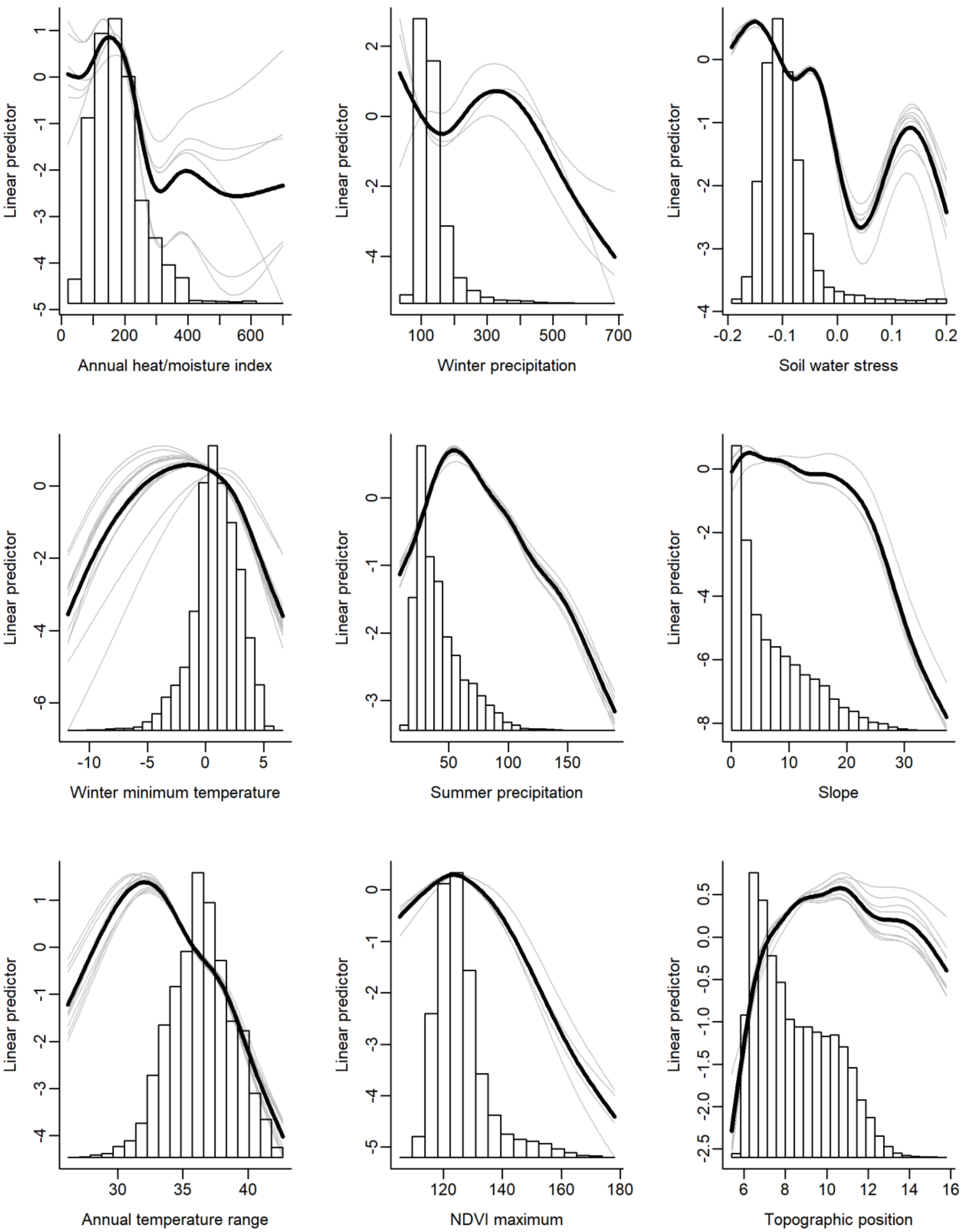

GAM response curves for *Yucca brevifolia*

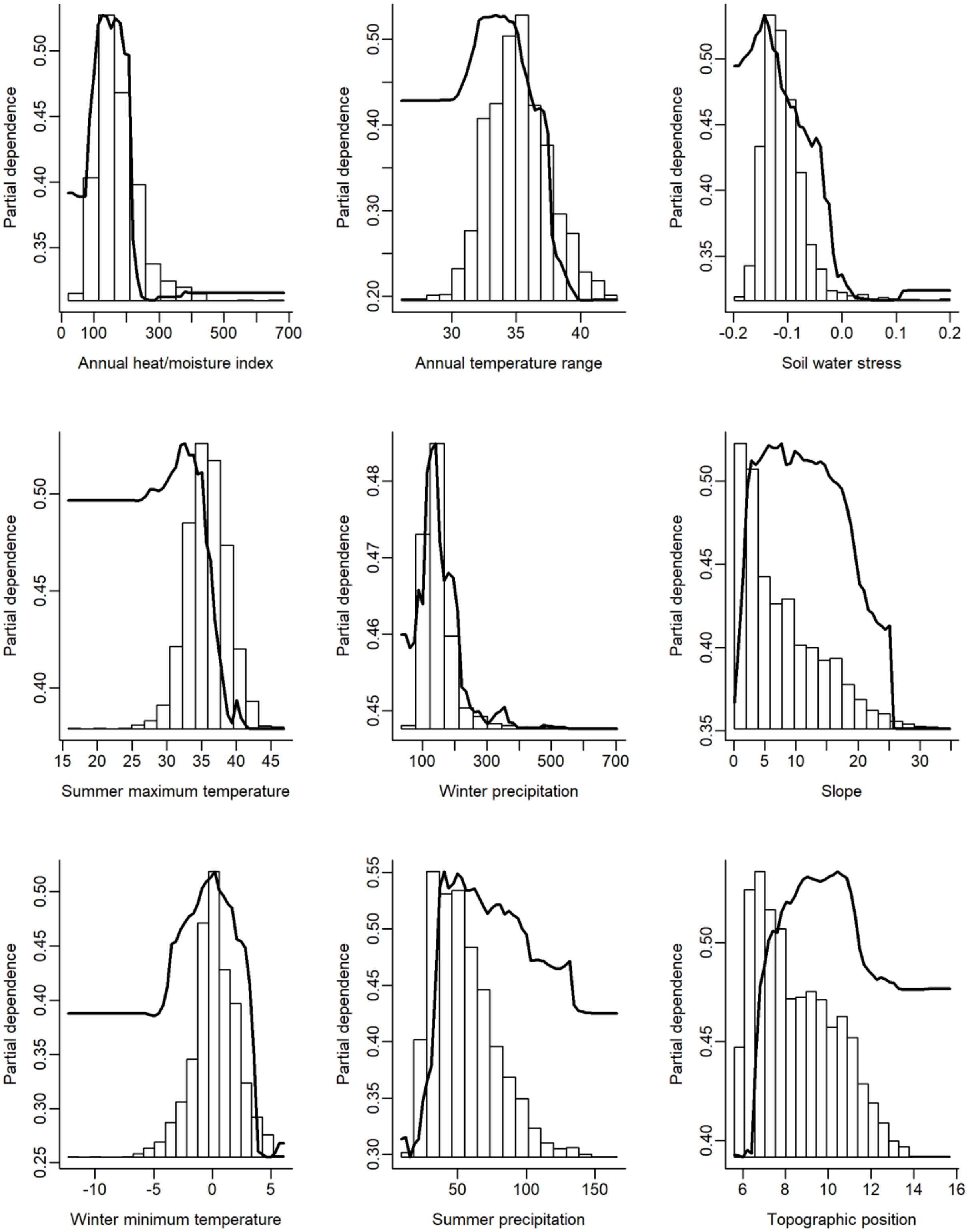

Random Forest response curves for *Yucca brevifolia*

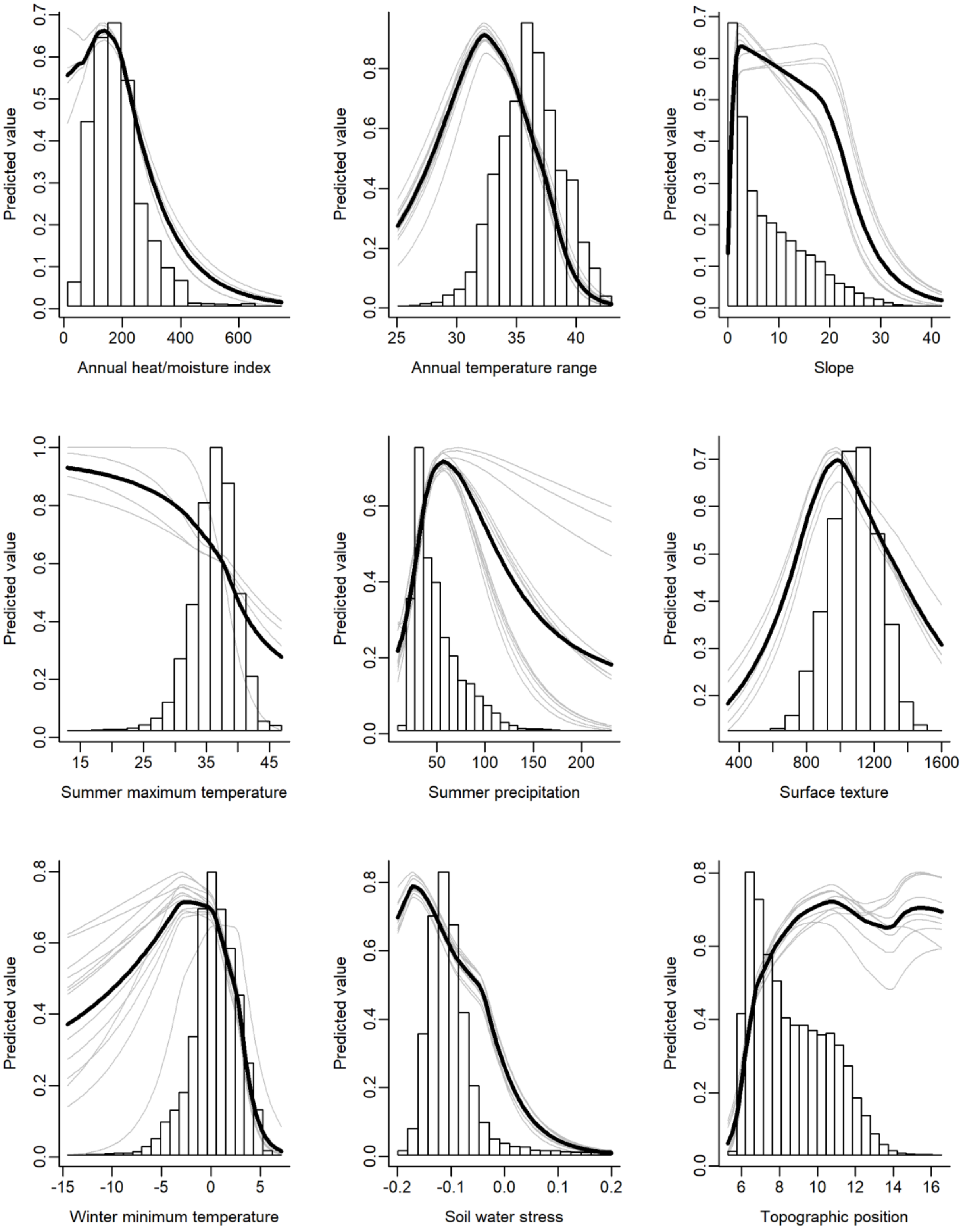

MaxEnt response curves for *Yucca brevifolia*

49. *Yucca schidigera* SDM and response curves (GAM, Random Forest, Maxent)

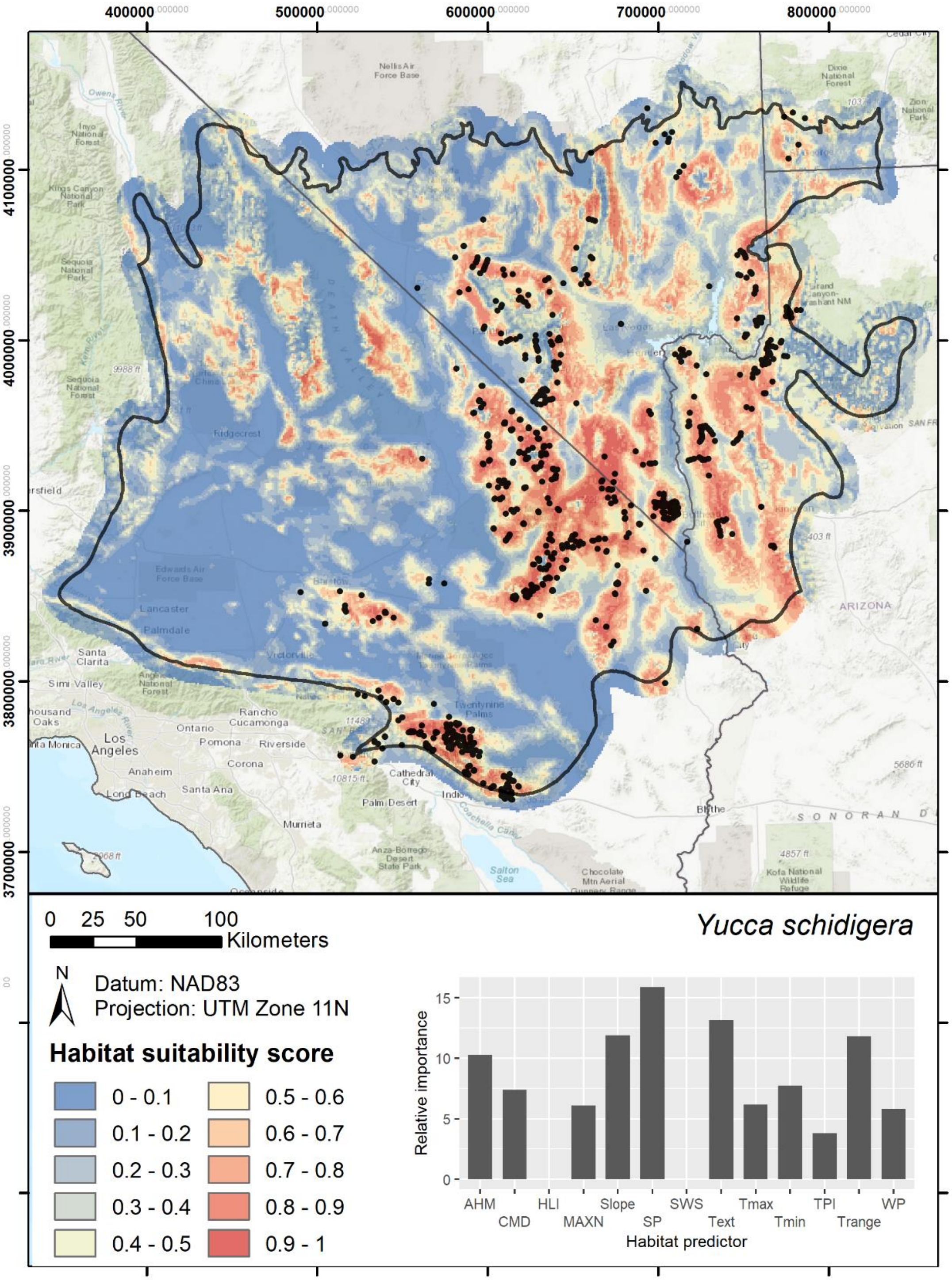

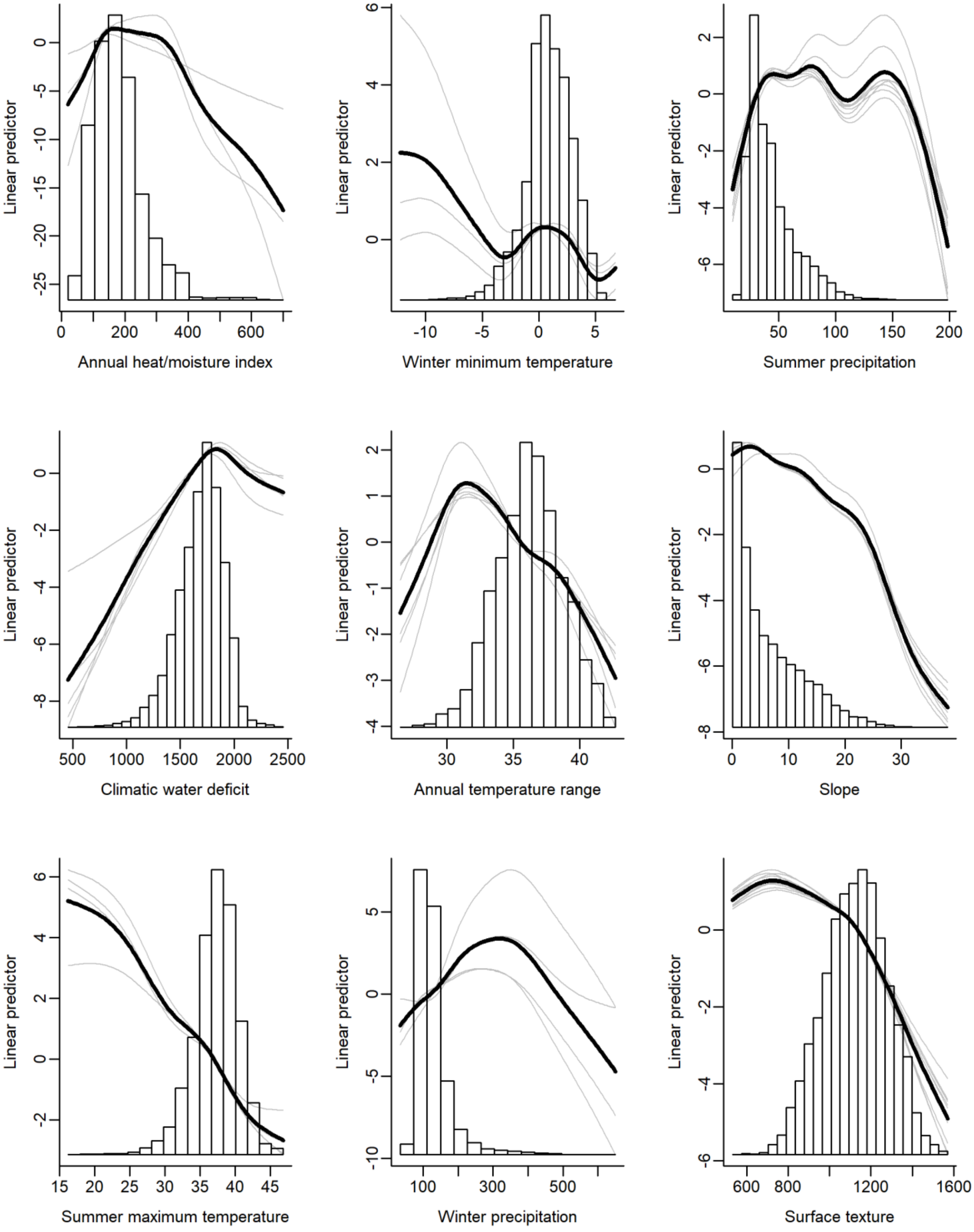

GAM response curves for *Yucca schidigera*

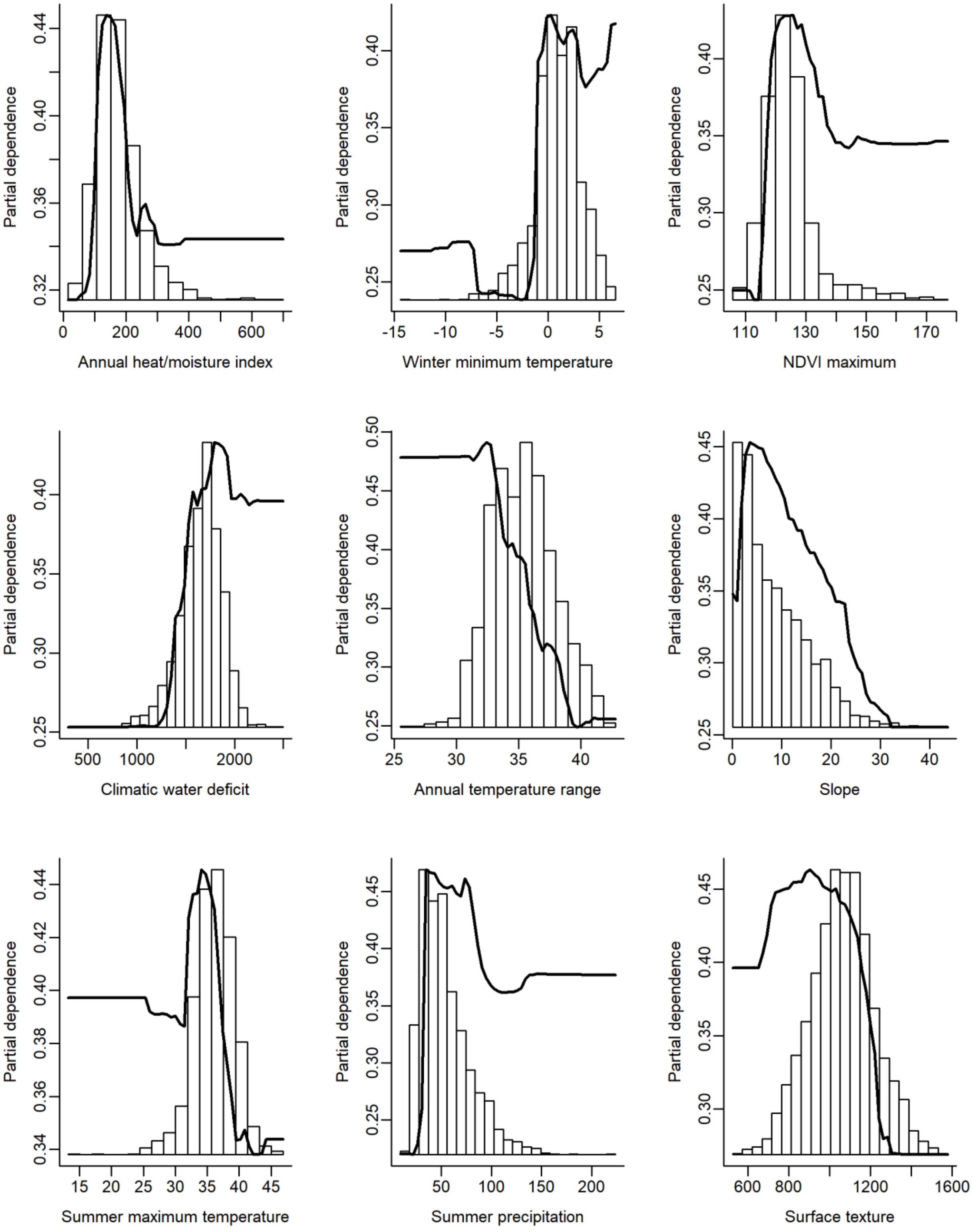

Random Forest response curves for *Yucca schidigera*

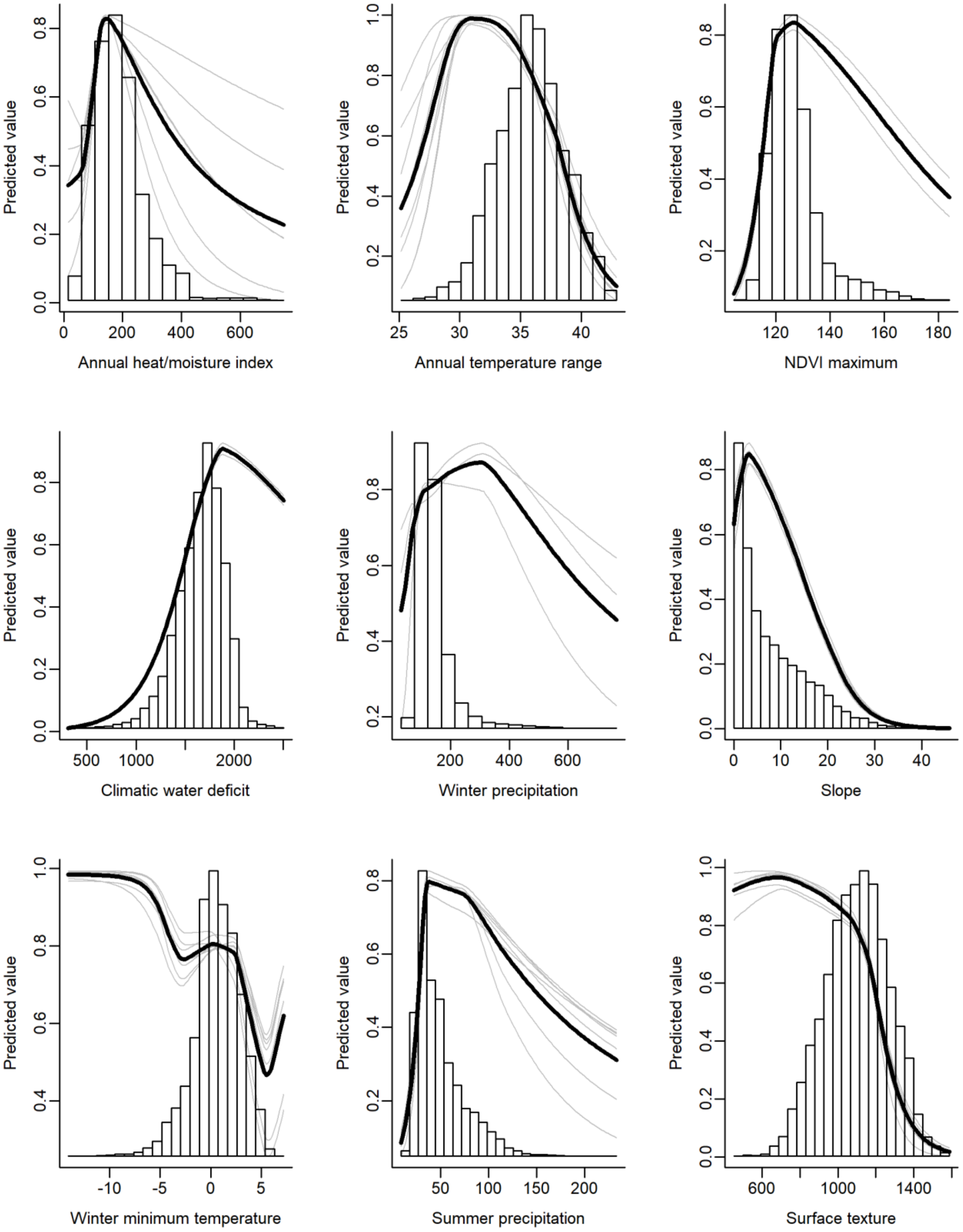

MaxEnt response curves for *Yucca schidigera*

Disclaimer

Any use of trade, firm, or product names is for descriptive purposes only and does not imply endorsement by the U.S. Government.
